# PANDORA: A Fast, Anchor-Restrained Modelling Protocol for Peptide: MHC Complexes

**DOI:** 10.3389/fimmu.2022.878762

**Published:** 2022-05-10

**Authors:** Dario F. Marzella, Farzaneh M. Parizi, Derek van Tilborg, Nicolas Renaud, Daan Sybrandi, Rafaella Buzatu, Daniel T. Rademaker, Peter A. C. ‘t Hoen, Li C. Xue

**Affiliations:** ^1^Center for Molecular and Biomolecular Informatics, Radboud Institute for Molecular Life Sciences, Radboudumc, Nijmegen, Netherlands; ^2^Institute of Biochemistry and Biophysics, University of Tehran, Tehran, Iran; ^3^Department of Biomedical Engineering, Institute for Complex Molecular Systems, Eindhoven University of Technology, Eindhoven, Netherlands; ^4^Natural Sciences and Engineering section, Netherlands eScience Center, Amsterdam, Netherlands; ^5^Bijvoet Centre for Biomolecular Research, Faculty of Science - Chemistry, Utrecht University, Utrecht, Netherlands

**Keywords:** peptide:MHC, integrative modelling, computational structural biology, large-scale 3D-modelling, computational immunology

## Abstract

Deeper understanding of T-cell-mediated adaptive immune responses is important for the design of cancer immunotherapies and antiviral vaccines against pandemic outbreaks. T-cells are activated when they recognize foreign peptides that are presented on the cell surface by Major Histocompatibility Complexes (MHC), forming peptide:MHC (pMHC) complexes. 3D structures of pMHC complexes provide fundamental insight into T-cell recognition mechanism and aids immunotherapy design. High MHC and peptide diversities necessitate efficient computational modelling to enable whole proteome structural analysis. We developed PANDORA, a generic modelling pipeline for pMHC class I and II (pMHC-I and pMHC-II), and present its performance on pMHC-I here. Given a query, PANDORA searches for structural templates in its extensive database and then applies anchor restraints to the modelling process. This restrained energy minimization ensures one of the fastest pMHC modelling pipelines so far. On a set of 835 pMHC-I complexes over 78 MHC types, PANDORA generated models with a median RMSD of 0.70 Å and achieved a 93% success rate in top 10 models. PANDORA performs competitively with three pMHC-I modelling state-of-the-art approaches and outperforms AlphaFold2 in terms of accuracy while being superior to it in speed. PANDORA is a modularized and user-configurable python package with easy installation. We envision PANDORA to fuel deep learning algorithms with large-scale high-quality 3D models to tackle long-standing immunology challenges.

## 1 Introduction

Major Histocompatibility Complex (MHC) was discovered *via* the study of transplantation compatibility (1) (hence the name). MHC proteins play a central role in immune surveillance systems and T-cell mediated immune attacks [see a comprehensive review (2)]. Cells constantly break down proteins into peptides. MHC proteins present some of these peptides on the cell surface. T-cells are fired up when their T-cell receptor (TCR) recognizes pathogenic peptides or tumor-specific presented on the cell surface by MHC proteins. MHC-I is presented on the surface of every cell, while MHC-II is presented only on specific immune cells, e.g., antigen presenting cells (APCs). Foreign peptides presented by MHC-I can activate CD8+ T-cells, which can directly kill infected cells that present the peptides on their surface. Peptides presented by MHC-II can activate CD4+ T-cells, which stimulate the production of antibodies and can provide help to CD8+ T-cells (3).

Investigations of pMHC structures are important in several ways. 3D structures can provided fundamental knowledge of MHC antigen-display mechanisms and T cell functions (4). Such knowledge can aid the design of new therapies for cancer (5), viral infections (6, 7), autoimmune disorders (8, 9), and aid the understanding immune control of immunodeficiency virus replication (10). Further, MHC structures, which are highly conserved over different species, may provide important knowledge about evolution relationships (11–15).

**Figure 1:**
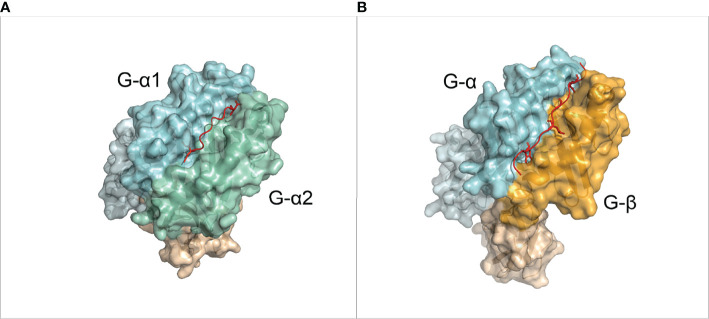
Overview of the MHC molecules. **(A)** 3D structure of a pMHC-I complex (PDB ID: 1DUZ). The α chain is divided in IMGT defined domains by shades of light blue. The *β*-2 Microglobulin chain is shown in light orange. The peptide is shown in red. **(B)** 3D structure of a pMHC-II complex (PDB ID: 1AQD). The alpha chain is divided in IMGT defined domains by shades of light blue. The *β* chain is divided in IMGT defined domains by shades of orange. The peptide is shown in red.

MHC is the most polymorphic protein known to date in humans (>32,000 identified alleles) (16). Each of these alleles has a specific binding preference for different peptides. Regardless of the highly polymorphic nature of MHC sequence, the MHC structure has an “Ultra-conserved” fold (17), which is present in nearly all jawed vertebrate species (12, 14). In MHC-I molecules, the peptide binding groove is formed by an *α*-chain, which has two domains denoted as G-ALPHA1 and G-ALPHA2 in IMGT nomenclature (18) (**Figure 1A**). This binding groove is closed on both N- and C-terminal side respective to the peptide binding and contains six binding pockets (A to F pockets) (19). Short peptides of around 8 to 11 residue lengths bind to two main deep pockets B and F with their second (P2) and last (PΩ) residues, respectively (20, 21). Secondary anchor residues (usually P4 and P7 in a 9-mer) can bind to C-E pockets and affect the binding affinity and peptide conformation (19). The peptide-binding groove of MHC-II is formed by two domains from *α*- and *β*-chain (corresponding to the G-ALPHA and G-BETA domains in IMGT nomenclature). This groove is open in both ends, and can therefore accommodate longer peptides (**Figure 1B**). Usually, 9 residues of the peptide, referred to as the binding core, bind to MHC binding groove, and the rest of the peptide protrude out of the groove. The peptide is mainly anchored at the P1, P4, P6 and P9 pockets of MHC-II (19, 21). Examples of non-canonical peptide anchor positions have been reported both for human pMHC-I (22) and pMHC-II (23), and for some other species’ pMHC-I (24).

Complementary to atomic-resolution 3D structure determination experiments (such as X-ray and NMR), the recent advances of large-scale mass spectrometry provide valuable tools to detect MHC binding peptides (25–27). However, a nearly infinite number of potential peptides could be derived from host cells and diverse pathogens. The high diversity of MHC and peptide sequences call for the development of effective computational methods for modelling pMHC structures.

In the past decades, many efforts have been devoted to design reliable modelling approaches to model 3D structures of pMHC complexes. There are three basic approaches for modelling 3D pMHC structures: (1) molecular dynamics (MD) (28–30), (2) molecular docking (31–33), and (3) homology modelling (34) [see review (35)]. MD approaches have shown to produce accurate structures; however, they are computationally intensive. State-of-the-art methods are often hybrid methods of these three techniques, to make pMHC modelling computationally accessible and still reliable (34, 36, 37). The general design of the state-of-the-art methods is as: (i) generating peptide conformation(s) based on a template conformation; (ii) inserting peptide into fixed MHC-I backbone; and (iii) optimization of the overall conformation including side-chains.

Several state-of-the-art methods for modelling pMHC-I are available. DockTope (37) models pMHC-I complexes for 4 different MHC-I alleles. It docks the peptide to MHC-I with different initial points and then selects the best conformation. It subsequently optimizes the conformation of pMHC-I with GROMACS (38) and repeats the docking to refine the pMHC-I structure. GradDock (36) constrains the peptide ends and generates numerous peptide conformations, and subsequently uses steered gradient descent to simulate binding of the peptide to MHC-I. After topological correction, a novel Rosetta-based scoring function selects the best candidate. Later APE-Gen (34) was proposed, adding the receptor modelling with MODELLER (39) before the three main steps mentioned above. APE-Gen also anchors the peptide termini and utilizes Random Coordinate Descent (RCD) (40) loop closure to generate peptide conformations. For energy optimization, it utilizes a molecular docking tool. In APE-Gen, all the main steps are run iteratively.

More recently, AlphaFold2 (41) and RoseTTAfold (42) have demonstrated outstanding performance in single-chain protein structure prediction. There have been few attempts modelling peptides using AlphaFold2, either using AlphaFold2 multimer (43) or by linking the peptide to the protein using peptide linkers (44, 45). However, pMHC interactions present unique challenges and have not been solved yet. This is mainly due to two factors: 1) peptides in pMHC databases are often synthetic peptides or originate from frameshift events, and therefore do not possess enough evolutionary information to generate an MSA (Multiple Sequence Alignment), which is the main piece of knowledge used as input from these DL-based prediction methods (41); 2) peptides are highly flexible, which necessitates the use of specific domain knowledge to reduce the large conformational search space, e.g. by guiding the anchors when docking the peptide. General purpose AI software is often slower than integrative modelling, thus not fitting to model millions of pMHC interactions.

To overcome the limitations of existing software, we developed PANDORA (Peptide ANchoreD mOdelling fRAmework), an anchor-restrained homology modelling software for pMHC complexes. PANDORA integrates two key structural concepts of pMHC binding: First, MHC molecules have a highly conserved overall structure; Second, MHCs use anchor pockets to dock peptides. PANDORA first searches a template structure from its large structure database and aligns the target MHC and peptide against the template. Then it performs an anchor restrained loop modelling to produce an accurate model of the peptide conformation. By using a restrained energy minimization step, the modelling phase is kept short, resulting in one of the fastest pMHC modelling pipelines so far. This enables large-scale proteome modelling of pMHCs for training subsequent ML algorithms. PANDORA allows users to specify anchor residues. This feature makes it the first software that is applicable to both pMHC class I and II. PANDORA also allows for multiple types of input from the user, such as non-canonical anchor position or predicted secondary structures of the peptide. Here we present PANDORA’s performance on pMHC-I.

We first demonstrate PANDORA’s performances on a cross-validation set of 835 pMHC-I structures. We then compare it with three pMHC-I modelling softwares (APE-Gen, DockTope and GradDock) on several pMHC-I sets with experimental structures. Finally, we performed a qualitative evaluation of different AlphaFold2 approaches against PANDORA on modelling 4 pMHC-I structures. PANDORA performs competitively, or better than these pMHC-I modelling softwares in terms of accuracy and computational time, while providing an easier installation and flexible user experience. PANDORA is 6 (APE-Gen) to ~72 (DockTope) times faster than state-of-the-art methods, a crucial factor for whole proteome analysis in the deep learning era.

## 2 Results

### 2.1 Description of PANDORA

**Figure 2:**
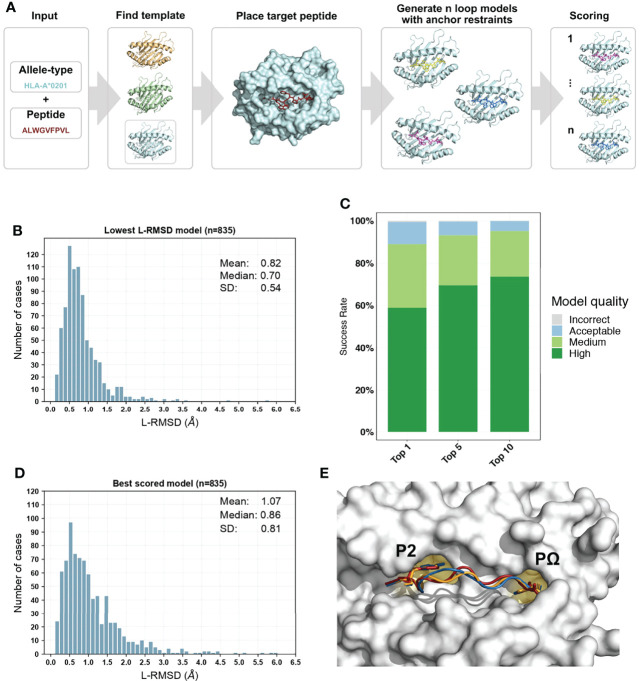
Overview of PANDORA pMHC-I protocol and its performance on 835 pMHC-I complexes with X-ray structures. **(A)** PANDORA schematic flowchart. An allele type and peptide sequence of a target pMHC-I case are given as input. This information is used to identify the best matching template structure from a local database of pMHC-I structures. The target MHC is then modelled on top of the template and its peptide (red) is superposed on the template peptide. The anchor positions (specified by the user or by other tools, see section 2.4) are then specified as fixed. MODELLER then generates 20 loop models maintaining the anchor restrained. Finally, all models are scored with MODELLER internal molpdf scoring function. **(B)** Sampling performance of PANDORA in our cross-docking benchmark experiment. Histogram of the lowest backbone L-RMSD models is shown. **(C)** Success rate of Backbone L-RMSD at different thresholds according to CAPRI criteria: High-quality (L-RMSD <1 Å), Medium, (<2 Å), Acceptable (<5 Å), and Incorrect (<10 Å) (Lensink et al., 2020). **(D)** Complete performance of PANDORA (modelling + scoring). Histogram of the backbone L-RMSD of the best molpdf models is shown. **(E)** Example of an average-quality 3D model generated with PANDORA. The target peptide (PDB ID: 3I6L) is marked in red; the template structure (PDB ID: 3WL9) is marked in blue; the model structure is marked in orange.

Our information-driven homology modelling framework PANDORA takes a few crucial steps (**Figure 2A**) to provide core domain knowledge to MODELLER (39). MHC’s high structural homology and anchoring positions for bound peptides are used to constrain the conformational search space to effectively produce an ensemble of 3D models.

First, PANDORA builds a large template database, which consists of all valid peptide-MHC-I structures from IMGT/3Dstructure-DB. All structures in the template database are renumbered starting from 1. The renamed chain ID of peptides is P; that of MHC is M. As allele names, PANDORA relies on G-domain allele names from IMGT, which are assigned based on Multiple Sequence Alignments of only G-domains, because this is the domain responsible for the peptide binding (18). One structure can have more than one allele name since the same G-domain can be shared by multiple MHC alleles. For this reason, any reference to MHC alleles in this paper is to G-domain alleles.

During a modelling run, PANDORA selects a suitable structural template for the given target from our parsed database. It then uses MODELLER to build an initial 3D structure, keeps the anchors restrained and performs a loop modelling on the central region of the peptide to output the final structures. Output models are ranked to indicate to the user which are the best ones.

### 2.2 PANDORA Produces Near-Native Models on a Large Benchmark Set

We benchmarked PANDORA on all pMHC-I complexes with experimentally determined structures in the IMGT/3Dstructure-DB database (46) (as of 28/06/2021) that could be parsed by our protocol (see Materials and Methods): 835 complexes over 78 MHC-I allele types (PDB IDs reported in **Supplementary Table 1**). We removed one structure from this dataset and used it as the test case. This process was repeated for every structure in the dataset. We used MHC allele name, actual peptide anchor position and peptide sequence as inputs for PANDORA, and asked PANDORA to generate 20 model structures.

When evaluated on the lowest backbone Ligand Root Mean Square Deviation (L-RMSD) (47), PANDORA produces at least one near-native model (L-RMSD < 2Å) in 96.6% of the cases and an overall mean deviation of 0.82 ± 0.54 Å (**Figure 2B**). Also when evaluated on lowest full-atom L-RMSD, PANDORA produces high-quality models, with an overall mean deviation of 1.53 ± 0.73 Å (**Supplementary Figure 1**). L-RMSD values used in **Figure 2** can be found in **Supplementary Table 2**. MODELLER’s internal molpdf function provides high-quality ranking for the models produced by PANDORA. To select which models should be provided to the user as output, we evaluated MODELLER’s scoring functions molpdf and DOPE with compared hit rate and success rate plots (**Supplementary Figure 2**), obtaining the best results from molpdf. **Figure 2D** shows this scoring function reaching a success rate of 93% in the top 10 models (see Methods section 5.3). The resulting L-RMSD median of top scored models with molpdf (the final best output provided to the user) is 0.86 Å. Therefore, PANDORA’s scoring method, together with the sampling procedure, allows us to deliver reliable predictions (**Figure 2C, D**).

To obtain an *a priori* estimate of PANDORA’s performance on a given MHC allele – peptide combination, we checked the performance of PANDORA with respect to different peptide lengths (**Supplementary Figures 3A, B**), sequence identities between query and template peptides (**Supplementary Figures 3C, D**) and MHC allele difference between target and template (**Supplementary Figures 3E, F**). PANDORA gives the best performance on 8-9 mer peptides with an average L-RMSD of 0.69 Å. PANDORA models generated with 100% peptide identity are slightly better than other peptide similarities (0.57 Å in median RMSD), but no clear trend is observed with respect to the peptide sequence identity (**Supplementary Figures 3C,D**). Reasonable performances were also reached in the rare cases (16 out of 835) in which no template from the same gene as the target were available (**Supplementary Figures 3E,F**). This shows how PANDORA can be used to build model cases of well-known as well as rare alleles.

### 2.3 PANDORA Performs Competitively With State-of-the-Art Methods

We compared PANDORA with three existing methods for pMHC-I 3D modelling: DockTope (37), GradDock (36) and APE-Gen (34) on datasets used in their publications. As not all methods used scoring functions to select best models for their experiments, we compared with each method’s best scenario. Specifically, we used our top molpdf model (PANDORA’s default user output) to compare with the pipelines that used scoring functions (GradDock and DockTope), and our models with best L-RMSDs to compare with the pipeline that reported the best L-RMSD models (APE-Gen).

**Figure 3:**
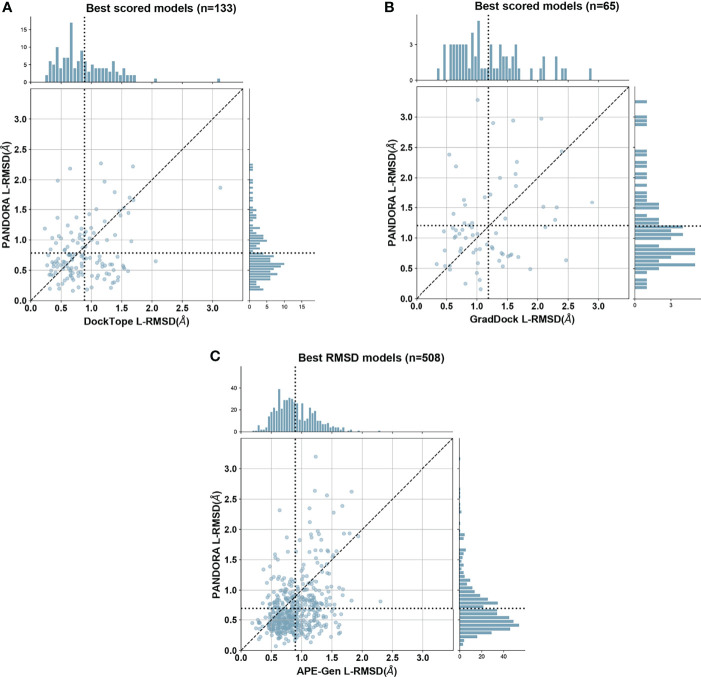
PANDORA comparison with the state-of-the-art methods. Y axes represent PANDORA L-RMSD per case while X axes represent the L-RMSD of the same case modelled with the reported method. The dotted line indicates the average L-RMSD for each method. **(A)** Difference between PANDORA best molpdf model Cα L-RMSD and DockTope reported Cα L-RMSD on 133 cases (PANDORA cross-docking against DockTope cross-docking); **(B)** Difference between PANDORA best molpdf model backbone + C*β* L-RMSD and GradDock reported backbone + C*β* L-RMSD on 65 cases (PANDORA cross-docking against GradDock cross-docking); **(C)** Difference between PANDORA best L-RMSD model Cα L-RMSD and APE-Gen reported Cα L-RMSD on 509 cases (PANDORA cross-docking against APE-Gen self-docking).

As shown in **Figure 3**, PANDORA is competitive with the state-of-the-art methods in terms of best-generated and top-selected models: both show, on average, lower L-RMSD obtained by the published methods. PANDORA L-RMSD values used in **Figure 3** are listed in **Supplementary Table 2**. The comparison between PANDORA and GradDock (**Figure 3A**) and DockTope (**Figure 3B**) are based on cross-docking. The comparison in **Figure 3C** reflects the results from the cross-docking experiments from PANDORA and the self-docking experiments from APE-GEN. Whereas self-docking uses the original bound conformations of target MHC and peptide as input to their modelling protocol, cross-docking inputs instead consist of conformations of MHC and peptide that are not the target one. Therefore, self-docking experiments are a simpler scenario than cross-docking experiments, and tend to give better results (36). PANDORA full-atom comparison with DockTope and APE-Gen can also be found in **Supplementary Figures 1B, C**.

PANDORA is computationally efficient. After downloading or building the templates dataset (both options have to be done only once, but building can require up to 1.5 hours) PANDORA takes an average of ~2.6 minutes (156 seconds) to build 20 models per case on one thread on a Intel(R) Xeon(R) Gold 6142 CPU @ 2.60GHz. According to their publications, DockTope takes “less than 6 hours”, and GradDock takes about “107.79” seconds to model one case, but lacking the exact hardware information a fair comparison is not possible. Given the availability and installation conditions of the softwares we compared with, a direct comparison of the running times can in fact be done only with APE-Gen. APE-Gen takes 3 minutes to prepare the MHC 3D structure plus 2 minutes per each pMHC-I complex using 6 or 8 threads (48). With roughly the same computational time and number of cores (i.e., 5 minutes on 6 to 8 cores), PANDORA can model up to 11-15 cases.

To have a qualitative evaluation of PANDORA against AlphaFold2, we tested multiple published strategies for protein-peptide interaction modelling. We tried the multimer-approach (43) and linker-approach (44, 45) using template-based and template-independent AlphaFold2 publicly available colabs (41, 49). As reported in **Supplementary Figure 4**, PANDORA always generated models with a considerably lower backbone L-RMSD than AlphaFold2 on the four randomly selected pMHC complexes. Also, PANDORA’s cost in terms of computational resources previously discussed is much lower than that of AlphaFold2, which can take up to 18 GBs of GPU power for 20 minutes to model one single pMHC case, making modelling of millions of pMHC very expensive with such a tool.

### 2.4 Correct Anchor Positions Play a Key Role

**Figure 4:**
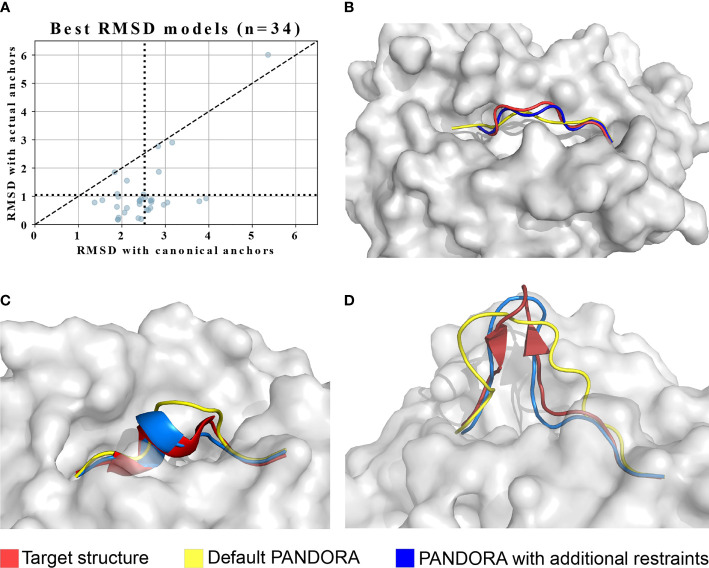
PANDORA’s case studies on with non-canonical cases. The images are oriented to present the most representative view of the difference between models and target. **(A)** PANDORA produced better models than using canonical anchor positions in terms of backbone L-RMSD of cases. **(B)** A typical case (target PDB ID: 1DUY, template PDB ID: 1AO7 in Red, peptide=LFGYPVYV) with non-canonical anchor positions Blue with (actual anchors: P1 and P9(Ω). **(C)** Case study on the 10-mer from PDB structure 3BEW (Red). L-RMSD with default settings: 2.02 Å (Yellow); L-RMSD using secondary structure restraints: 0.80 Å (Blue). **(D)** Case study on the 15-mer from PDB structure 4U6Y (Red). L-RMSD with default settings: 3.32 Å (Yellow); L-RMSD using secondary structure restraints: 1.50 Å (Blue).

As mentioned in section 2.1, the input we provided PANDORA with was the actual target peptide anchors that were calculated directly from the target structure. We did so to avoid biases derived from wrong anchor prediction in our benchmark performance. The anchor information is crucial for our modelling pipeline. The majority of 9- to 12-mer MHC-I peptides have canonical anchoring positions at P2 and PΩ (20, 21). To study the effect of non-canonical peptide anchoring in the 3D modelling process of PANDORA, we listed which peptides from our benchmark dataset used non-canonical anchor positions to bind to the MHC, resulting in a total of 34 cases. We modelled them as in the previous benchmark experiment, with the only difference that canonical anchor positions were used as input of PANDORA. The models with the lowest L-RMSD are reported in **Figure 4A**, where the average L-RMSD improvement achieved using real anchors over canonical anchors is 1.46 Å. This result is better exemplified in **Figure 4B**, where it can be observed how defining incorrect (canonical, in this case) anchors causes PANDORA to fix the wrong residues inside the anchor pockets, stretching (as shown) or elongating the peptide central loop, thus worsening the L-RMSD with the x-ray structure.

Since the real anchor position is hardly available with peptide-MHC binding data, we evaluated the reliability of predicting anchor positions using prediction tools. NetMHCpan4.1 is a binding affinity and core prediction tool for pMHC-I complexes (50). An overview of NetMHCpan4.1 performance on predicting the right anchors over our whole dataset can be found in **Supplementary Figure 5**, showing that NetMHCpan4.1 provides reliable anchor predictions for most of the cases: correct anchor prediction in 96.5% of the tested cases, one residue shift for 3.2% cases, and position shifts on both anchors in 0.2% cases. Based on these observations, we implemented the following options in PANDORA: to use either canonical anchors, NetMHCpan4.1 predicted anchors, or anchor points directly provided by the user, who may exploit different tools or gather integrative information from experimental data.

### 2.5 Long Peptides Are Challenging to be Modelled Reliably

Long peptides (11-15 mers) present a challenge to be modelled reliably (**Supplementary Figure 3A**). This is because long peptides are able to fold into small elements of secondary structure in their central part. This problem, although rare (17 cases out of 835 structures in our benchmark dataset presented elements of secondary structure), presents a modelling challenge (model L-RMSD up to 3.03 Å).

To address this challenge, we enabled PANDORA to include secondary structure restraints and tested its performance on a 10-mer (PDB ID: 3BEW) and a 15-mer case (PDB ID: 4U6Y). In these structures the peptide presents in its center region a small alpha helix (16 structures out of 835) and a small beta-sheet hairpin (1 structure out of 835), respectively. We manually defined secondary structure restraints for the peptide based on the bound conformation found in the PDB structure. Secondary-structure restraints improved model qualities for both cases (**Figures 4C, D**). This indicates that a correct secondary structure prediction can be provided to PANDORA to guide its modelling, increasing the quality of the models for similar challenging cases.

### 2.6 Software Information

PANDORA is designed to be a robust and user-friendly python package, which can be integrated into other python pipelines. It is highly modularized (see **Supplementary Figure 6** for the object relation diagram) and supports continuous integration, facilitating automatic integration of code development from multiple developers.

PANDORA builds its template database through a robust, automated and yet adjustable module. This module takes care of downloading, parsing and homogenizing the queried types of structures and it summarizes their information (e.g., sequences, allele information, anchor positions, biopython structure object) in an easily accessible python object, providing the base for other methods that might use these data for different purposes. The same module also downloads and parses reference sequences from the manually curated sequence database https://www.ebi.ac.uk/ipd/mhc/ (51, 52) to build a local, reliable MHC-I sequence database of multiple species. Both the structural and the sequence database can be rebuilt or updated at any moment with ease by the user, and multiple databases with different parsing criteria can be saved at the same time.

PANDORA takes as input: 1) peptide sequence and 2) MHC allele name. PANDORA by default assumes the canonical anchor positions. Users may easily personalize their runs by adding anchor information or secondary structure predictions, increasing the number of generated loops, changing refinement mode or providing personalized MHC sequences. Expert users of MODELLER may further personalize the main MODELLER modelling scripts to adapt the pipeline to their specific needs.

PANDORA is computationally efficient (see Section 2.1 for average running times) and copes well with large-scale modelling tasks on HPC (High Performance Computing) facilities. PANDORA supports parallelization at multiple levels (per-case or per model). A short tutorial with six different examples showing the ease of setting up different types of PANDORA run can be found in our GitHub repository (https://github.com/X-lab-3D/PANDORA). Users can report problems, ask for assistance or request specific features to be added from the GitHub issue section.

## 3 Discussion

In this study we present PANDORA, a user-friendly and modularized pMHC modelling pipeline. PANDORA takes anchor positions as restraints, making it generally applicable to both pMHC-I and II. Here we present PANDORA’s performance on pMHC-I. We demonstrate that PANDORA performs reliably on the largest pMHC-I dataset obtained from IMGT/3Dstructure-DB (46). PANDORA also shows competitive performance compared with three state-of-the-art pMHC-I modelling softwares while PANDORA is superior in computational efficiency (6 to 72 times faster) and ease of installation.

PANDORA distinguishes itself from existing methods in several ways. PANDORA is the first software in the field that provides a comprehensive cleaned template dataset which can be easily updated (see “Template Set Building” in section 4.1). Moreover, PANDORA dynamically chooses the most appropriate template per case, instead of simply relying on one template per peptide length group, as APE-Gen does. PANDORA is marked by its simplicity and modularity. It relies only on one core software, MODELLER, instead of multiple dependencies as Ape-Gen does. As demonstrated by its class diagram (**Supplementary Figure 6**), PANDORA is highly modular making it easy to maintain and extend. Being a modular python package, our method (or single sub-modules of it) can be integrated in other pipelines with ease, which is considerably harder for the state-of-the-art softwares.

Although specific software for pMHC modelling are available, we could not avoid to compare our software with the groundbreaking, general-purpose 3D-modelling software AlphaFold2 (41), that has recently been used to accurately model tens of thousands of protein structures (53) including MHCs. PANDORA outperforms AlphaFold2 on the pMHC modelling task in terms of accuracy and computational time. To the best of our knowledge, it is not possible yet to provide distance restraints or secondary structure restraints to AlphaFold2, but its models can be biased by template structures (automatically identified by AlphaFold2). To evaluate the template influence on AlphaFold2 generated pMHC-I models, we tested two cases in which a template could be selected (**Supplementary Figures 4A, D**) and two cases in which a template could not be selected (**Supplementary Figures 4B, C**). Overall, our evaluation of AlphaFold2 on modelling pMHC-I complexes revealed that AlphaFold2 is often misplacing the P2 anchor residue outside its pocket, causing a high backbone L-RMSD compared to the X-ray structure (**Supplementary Figures 4A-C**). Furthermore, in presence of secondary structures, AlphaFold2 can generate even higher L-RMSD models (**Supplementary Figure 4D**).

When run with default settings (i.e., using P2 and PΩ as anchors for pMHC-I), our method achieves a median L-RMSD of 0.86 Å on our large benchmark dataset (**Supplementary Figure 7**), but it fails in delivering high-quality models for some, mainly non-canonical cases. Most of these outliers are caused by peptides with non-canonical anchor positions. To overcome this issue, users may opt for binding core prediction tools [such as NetMHCPan4.1 (50) or MHCflurry (54)] to guide PANDORA’s prediction or model peptides with multiple anchor positions and choose the ones with best molpdf scores. Also, high L-RMSD can be caused by long peptides able to fold into secondary structures. In these cases, users may decide on secondary structure- or folding-prediction tools such as AGADIR (55) and PEPFOLD3 (56) to elicit secondary structures restraints to input into PANDORA. For long peptides that do not form secondary structures, users might just use a much larger sampling step, increasing the number of generated models to hundreds or thousands. We report in **Supplementary Figure 8** how a larger sampling results in slightly increased models’ quality.

While we evaluated PANDORA on MHC-I cases only here, PANDORA is designed to be applied to MHC-II as well. We systematically investigated PANDORA’s performance on pMHC-II (manuscript under preparation). PANDORA with support for both MHC-I and MHC-II is freely accessible for academic usages (see Code Availability).

PANDORA supports multi-level parallelization and multiple user-configurable options (see GitHub README at: https://github.com/X-lab-3D/PANDORA). These features make it suitable for high-throughput purposes as well as to explore the modelling of particularly challenging peptides (e.g., peptides of non-canonical length). In fact, its computational efficiency allows users to quickly run thousands of cases or to increase the models’ sampling (from the default of 20 to hundreds or thousands) to explore a higher variety of conformations. This computational efficiency is combined with easy installation, flexibility, robust template data collection and high quality of the produced models. PANDORA thus makes a reliable tool for research groups that might need either fine-tuned, accurate 3D models of single pMHC cases, or large-scale modellings (both on HPC facilities or modest desktops).

Lastly, PANDORA is able to enrich the large amount of existing sequence-based binding data with high-quality 3D models, providing 3D enriched data to subsequent ML algorithms. PANDORA’s accuracy and computational requirements makes it affordable to generate millions of 3D models. 3D-based AI frameworks like DeepRank (57) and DeepRank-GNN (58) can then exploit these to tackle long standing challenges in pMHC-based vaccine design (work in progress).

## 4 Materials and Methods

### 4.1 PANDORA Protocol

The PANDORA package generates pMHC 3D models through restraint-guided homology modelling. PANDORA can take as input one or multiple peptide sequences and an MHC-I IMGT allele name (46) for each peptide. It returns by default 20 model structures (adjustable) in PDB format, ranked by MODELLER’s internal scoring function molpdf (39). To build the pMHC models, PANDORA covers three main steps (shown in **Figure 2A**): i) template set building, ii) input preparation and iii) 3D modelling, described below. Although PANDORA is designed to work for both MHC class I and II, below we focus on the protocol for pMHC-I as our experiments presented in this paper are on MHC-I (MHC-II manuscript under preparation).

#### 4.1.1 Template Set Building

PANDORA automatically builds an extensive cleaned template set. The whole compressed IMGT/3D-structureDB (46) is downloaded and a list of all the MHC-I PDB IDs is queried from the IMGT webserver. For our cross-validation benchmark experiment, such list consisted of a total of 1188 pMHC-I PDB IDs (downloaded on March 23rd of 2021). Template pMHC-I structures from such list with peptide length spanning from 7 to 15 (adjustable) residues are then extracted. From each of these PDB file only one Alpha chain (if multiple copies are available) and its bound peptide are extracted (β2-microglobuline is neither saved in the template object, nor modelled). If present, non-canonical residues are changed into canonical residues when no coordinate modifications are required (e.g., changing phosphoserines in serines by removing the phospho group) (see **Supplementary Table 3** for the list of tolerated non-canonical residues). However, the template is removed from the dataset when: 1) other non-canonical residues are present; 2) a small, non-amino acid molecule besides the peptide is present inside the MHC binding groove; 3) the PDB structure cannot be parsed in Biopython (59) for additional reasons; or 4) the file is lacking allele information from IMGT. The final parsed dataset we used in our experiments consisted of 835 PDB structures over 78 MHC-I G-domain alleles (the case 3RGV had to be manually removed due to unexpected issues in the parsing).

#### 4.1.2 Input Preparation

##### 4.1.2.1 Template Selection

For each pair of MHC allele type and peptide sequence provided by the user, PANDORA searches the template database, computes a list of putative templates and selects the first of the list as template. First, it searches for templates that share the same MHC allele type (e.g., HLA-A*02:01) as the target. If no such templates are available, the putative templates list is compiled with structures from the same allele group (e.g., HLA-A*02) as the target. If these do not yield any putative templates either, PANDORA extends the search for structures from the same gene (e.g., HLA-A) as the target. Once PANDORA has compiled such list of putative templates, the final template is selected from them based on the peptide sequence similarity. Specifically, the sequences from putative templates’ peptides are then aligned with the target peptide sequence as follows. First, the anchor positions of the putative templates and target sequences are aligned, then gaps are added in the exact centre of the binding core (or in the flanking regions) if needed to match different lengths. These peptide anchor-position-driven alignments are ranked with a PAM30 matrix to select the best template.

##### 4.1.2.2 Alignment File Generation

Once a template is selected based on MHC allele name and peptide sequence, its MHC sequence is aligned with the target by using MUSCLE (60), while for the peptides the anchor-position-driven alignment generated for the template selection step is maintained. For the benchmark experiment, the MHC sequence for each case was retrieved from the target structure to be modelled. Besides MHC types, users may also provide MHC sequences. If a user does not provide MHC sequences, PANDORA will automatically retrieve it from the reference MHC allele sequence [retrieved from https://www.ebi.ac.uk/ipd/mhc/ (51)] according to the provided allele name.

#### 4.1.3 3D Modelling

PANDORA is built on top of MODELLER (39). The template structure file, anchor positions and the template-target alignment file are fed into MODELLER to generate target pMHC models. First, the MHC structure is generated with a simple homology model over the template structure. Then, anchor positions are provided to MODELLER to indicate which part of the peptide should be kept restrained. Finally, twenty models (adjustable) of the peptide are produced by using MODELLER loop modelling method. Specifically, MODELLER takes the initial loop model (build on top of the template structure) and randomizes its structure by ± 5Å to generate 20 initial models. Each of these models undergoes a short, two-phase energy minimization to produce one final loop model each. Generated models are then ranked using MODELLER’s built-in molpdf function for selection of near-native decoys. In case the target peptide sequence and MHC allele are identical between target and template, the initial loop model generated by MODELLER (which has the same structure as the template) is scored as top model (by applying a fictional molpdf score lower equal to the lowest molpdf achieved by any model for the same run minus one) and provided as best output. Also, the user is informed of this sequence identity and pointed to the deposited X-ray structure from PANDORA’s log.

### 4.2 Comparisons With State of the Art

Datasets for comparisons with state-of-the-art methods were retrieved from each software’s paper or kindly provided by the authors. Some structures could not be processed by PANDORA according to the criteria listed in section 5.1 or were not found in IMGT/3Dstructure-DB, resulting in smaller comparison datasets than the exact ones provided in literature. We used 133 out of 135 structures for **Figure 3A** and **Supplementary Figure 1C** (DockTope), with a peptide length span from 8 to 10 over 5 MHC alleles; 65 out of 69 for **Figure 3B** (GradDock), with a peptide length span from 8 to 10 over 21 MHC alleles; and 508 out of 535 for **Figure 3C** and **Supplementary Figure 1B** (APE-Gen), with a peptide length span from 8 to 11 over 59 MHC alleles.

### 4.3 Evaluations

#### 4.3.1 Ligand Root Mean Squared Deviation (L-RMSD)

The models’ quality was evaluated in terms of L-RMSD (47). All the L-RMSDs provided, unless otherwise specified, refer to backbone (i.e. Cα, N, C, O) L-RMSD. This calculation provides information on the full backbone position together with the side chain orientation (since the Carbon *β* position is mainly fixed, given the backbone orientation). Specifically, the G-domains (positions 1-180) of models and target structures were superposed and the L-RMSD was calculated as the RMSD between the atoms of the experimentally determined peptide conformation and the modelled peptide. L-RMSDs were calculated using ProFit (61). When directly comparing with state-of-the-art methods, we used the same sets of atoms as these works did: Carbon α L-RMSD (**Figures 3A, C** respectively) and full-atom L-RSMD (**Supplementary Figures 1C, B** respectively) for DockTope and APE-Gen and Backbone + Carbon *β* L-RMSD (**Figure 3B**) for GradDock.

#### 4.3.2 Hit Rate and Success Rate

Hit Rate and Success Rate are widely used in computational modelling for biomolecular complexes (62). A hit here is a model with an L-RMSD < 2 Å from the target structure (63). The Hit Rate is defined as the percentage of hits taken when selecting the top N ranked models, averaged over every case:


$$Hit\;Rate(K)\;=\frac{\;n_{hits}(K)}{M}$$

where *n_hits_* (*K*) is the number of hits (i.e., near-native models) among top K models and *M* the total number of near-native models for this case.The Success Rate is defined as the number of cases, taken the top N ranked models, containing at least one hit, divided by the total number of cases:


$$Success\;Rate\;=\frac{n_{successful\_cases}(K)}{N}$$

where *n_successfull_cases_* (*K*) is the number of cases with at least one hit among top K models, and N is the total number of cases.

## Data Availability Statement

The list of PDB structure IDs used in the cross-validation benchmark experiment can be found in **Supplementary Table 1**. The L-RMSDs used for **Figures 2**–**4** and **Supplementary Figures 3, 7 **and **8** can be found in **Supplementary Table 2**. The PANDORA database used for the experiments in this work is available for download at: https://github.com/X-lab-3D/PANDORA_database.

## Author Contributions

LX, DM, and FP contributed to the design of the pipeline. DM, FP, LX, and DR contributed to the design and development of the experiments. FP and DS contributed to the preliminary experiments. DM, DT, FP, RB, and NR contributed to the development of the pipeline. NR, DM, and FP contributed to the release of the python package and online documentation. LX, DM, FP, and PH contributed to the discussion. DM, FP, LX, DR, PH, and DT contributed to the writing of the manuscript. All authors contributed to the article and approved the submitted version.

## Funding

This project is supported by the Hypatia Fellowship from Radboudumc (Rv819.52706). FP acknowledges a visiting scholarship from Department of Scholarships and Students’ Affairs Abroad, Ministry of Science, Research and Technology, Iran.

## Conflict of Interest

The authors declare that the research was conducted in the absence of any commercial or financial relationships that could be construed as a potential conflict of interest.

## Publisher’s Note

All claims expressed in this article are solely those of the authors and do not necessarily represent those of their affiliated organizations, or those of the publisher, the editors and the reviewers. Any product that may be evaluated in this article, or claim that may be made by its manufacturer, is not guaranteed or endorsed by the publisher.
