# Supplementary material for: PANDORA: A Fast, Anchor-Restrained Modelling Protocol for Peptide: MHC Complexes
Source: Front Immunol. 2022 May 10;13:878762. doi: 10.3389/fimmu.2022.878762 (PMC9127323; doi:10.3389/fimmu.2022.878762)
Supplement: Supplementary file 2 [file Table_1.docx]

**Supplementary Table 1. PANDORA cross-validation dataset.** For every case, peptide, real anchor position and IMGT alleles are reported.

| PDB_ID | PEPETIDE SEQ | ANCHORS | IMGT assigned G-DOMAIN ALLELE |
| --- | --- | --- | --- |
| 1A1M | TPYDINQML | 2;9 | HLA-B*5301;HLA-B*5301 |
| 1A1N | VPLRPMTY | 2;8 | HLA-B*3501;HLA-B*3542;HLA-B*3501;HLA-B*3542 |
| 1A1O | KPIVQYDNF | 2;9 | HLA-B*5301;HLA-B*5301 |
| 1A9B | LPPLDITPY | 2;9 | HLA-B*3501;HLA-B*3542;HLA-B*3501;HLA-B*3542 |
| 1A9E | LPPLDITPY | 2;9 | HLA-B*3501;HLA-B*3542;HLA-B*3501;HLA-B*3542 |
| 1AGB | GGRKKYKL | 2;8 | HLA-B*0801;HLA-B*0801 |
| 1AGC | GGKKKYQL | 2;8 | HLA-B*0801;HLA-B*0801 |
| 1AGD | GGKKKYKL | 2;8 | HLA-B*0801;HLA-B*0801 |
| 1AGE | GGKKKYRL | 2;8 | HLA-B*0801;HLA-B*0801 |
| 1AGF | GGKKRYKL | 2;8 | HLA-B*0801;HLA-B*0801 |
| 1AKJ | ILKEPVHGV | 2;9 | HLA-A*0201;HLA-A*0201 |
| 1AO7 | LLFGYPVYV | 2;9 | HLA-A*0201;HLA-A*0203;HLA-A*0204;HLA-A*0207;HLA-A*0209;HLA-A*0212;HLA-A*0213;HLA-A*0216;HLA-A*0217;HLA-A*0218;HLA-A*0219;HLA-A*0222;HLA-A*0224;HLA-A*0225;HLA-A*0226;HLA-A*0227;HLA-A*0233;HLA-A*0236;HLA-A*0237;HLA-A*0238;HLA-A*0239;HLA-A*0240;HLA-A*0249;HLA-A*0252;HLA-A*0266;HLA-A*0289;HLA-A*0271;HLA-A*0268;HLA-A*0274;HLA-A*0258;HLA-A*0280;HLA-A*0265;HLA-A*0286;HLA-A*0277;HLA-A*0260;HLA-A*0273;HLA-A*0285;HLA-A*0264;HLA-A*0275;HLA-A*0267;HLA-A*0295;HLA-A*0297;HLA-A*9201;HLA-A*0296;HLA-A*0293;HLA-A*9221;HLA-A*9231;HLA-A*9232;HLA-A*9214;HLA-A*9202;HLA-A*9210;HLA-A*9205;HLA-A*9217;HLA-A*9211;HLA-A*9204;HLA-A*9230;HLA-A*9234;HLA-A*9218;HLA-A*9247;HLA-A*9249;HLA-A*9251;HLA-A*9248;HLA-A*9256;HLA-A*9235;HLA-A*9252;HLA-A*9240;HLA-A*9253;HLA-A*9245;HLA-A*9277;HLA-A*9257;HLA-A*9271;HLA-A*9294;HLA-A*9264;HLA-A*9290;HLA-A*9266;HLA-A*9291;HLA-A*9289;HLA-A*9274;HLA-A*9262;HLA-A*9259;HLA-A*9263;HLA-A*9268;HLA-A*9288;HLA-A*9293;HLA-A*9287;HLA-A*9283;HLA-A*9267;HLA-A*9265;HLA-A*9282;HLA-A*9297;HLA-A*9298;HLA-A*9299;HLA-A*0201;HLA-A*0209;HLA-A*0266;HLA-A*0289;HLA-A*0275;HLA-A*0297;HLA-A*0296;HLA-A*9221;HLA-A*9232;HLA-A*9234;HLA-A*9240;HLA-A*9299 |
| 1B0G | ALWGFFPVL | 2;9 | HLA-A*0201;HLA-A*0201 |
| 1BD2 | LLFGYPVYV | 2;9 | HLA-A*0201;HLA-A*0201 |
| 1BII | RGPGRAFVTI | 2;10 | H2-D1*02;H2-D1*02 |
| 1BQH | RGYVYQGL | 2;8 | MH1-K1b;MH1-K1b |
| 1BZ9 | FAPGVFPYM | 2;9 | H2-D1b;H2-D1b |
| 1CE6 | FAPGNYPAL | 2;9 | H2-D1b;H2-D1b |
| 1CG9 | LPPLDITPY | 2;9 | HLA-B*3501;HLA-B*3542;HLA-B*3501;HLA-B*3542 |
| 1DDH | RGPGRAFVTI | 2;10 | H2-D1*02;H2-D1*02 |
| 1DUY | LFGYPVYV | 1;8 | HLA-A*0201;HLA-A*0296;HLA-A*9221;HLA-A*0201;HLA-A*0296;HLA-A*9221 |
| 1DUZ | LLFGYPVYV | 2;9 | HLA-A*0201;HLA-A*0296;HLA-A*9221;HLA-A*0201;HLA-A*0296;HLA-A*9221 |
| 1E27 | LPPVVAKEI | 2;9 | HLA-B*5101;HLA-B*5101 |
| 1E28 | TAFTIPSI | 2;8 | HLA-B*5101;HLA-B*5101 |
| 1ED3 | ILFPSSERLISNR | 2;13 | RT1-A;RT1-A |
| 1EEY | ILSALVGIV | 2;9 | HLA-A*0201;HLA-A*0296;HLA-A*9221;HLA-A*0201;HLA-A*0296;HLA-A*9221 |
| 1EEZ | ILSALVGIL | 2;9 | HLA-A*0201;HLA-A*0296;HLA-A*9221;HLA-A*0201;HLA-A*0296;HLA-A*9221 |
| 1EFX | GAVDPLLAL | 2;9 | HLA-C*0303;HLA-C*0304;HLA-C*0303;HLA-C*0304 |
| 1FFN | KAVYNFATM | 2;9 | H2-D1b;H2-D1b |
| 1FFO | AAVYNFATM | 2;9 | H2-D1b;H2-D1b |
| 1FFP | SAVYNFATM | 2;9 | H2-D1b;H2-D1b |
| 1FG2 | KAVYNFATC | 2;9 | H2-D1b;H2-D1b |
| 1FO0 | INFDFNTI | 2;8 | MH1-K1b;MH1-K1b |
| 1FZJ | RGYVYQGL | 2;8 | MH1-K1b;MH1-K1b |
| 1FZK | FAPGNYPAL | 2;9 | MH1-K1b;MH1-K1b |
| 1FZM | RGYVYQGL | 2;8 | MH1-K1b;MH1-K1b |
| 1FZO | FAPGNYPAL | 2;9 | MH1-K1b;MH1-K1b |
| 1G6R | SIYRYYGL | 2;8 | MH1-K1b;MH1-K1b |
| 1G7P | SRDHSRTPM | 2;9 | MH1-K1b;MH1-K1b |
| 1G7Q | SAPDTRPA | 2;8 | MH1-K1b;MH1-K1b |
| 1HHG | TLTSCNTSV | 2;9 | HLA-A*0201;HLA-A*0201 |
| 1HHH | FLPSDFFPSV | 2;10 | HLA-A*0201;HLA-A*0201 |
| 1HHI | GILGFVFTL | 2;9 | HLA-A*0201;HLA-A*0201 |
| 1HHJ | ILKEPVHGV | 2;9 | HLA-A*0201;HLA-A*0201 |
| 1HHK | LLFGYPVYV | 2;9 | HLA-A*0201;HLA-A*0201 |
| 1HOC | ASNENMETM | 2;9 | H2-D1b;H2-D1b |
| 1HSA | ARAAAAAAA | 2;9 | HLA-B*2705;HLA-B*2707;HLA-B*2709;HLA-B*2710;HLA-B*2713;HLA-B*2714;HLA-B*2719;HLA-B*2728;HLA-B*2727;HLA-B*2732;HLA-B*2735;HLA-B*2734;HLA-B*2738;HLA-B*2741;HLA-B*2745;HLA-B*2743;HLA-B*2750;HLA-B*2746;HLA-B*2755;HLA-B*2756;HLA-B*2747;HLA-B*2754;HLA-B*2758;HLA-B*2760;HLA-B*2705;HLA-B*2713 |
| 1I1F | FLKEPVHGV | 2;9 | HLA-A*0201;HLA-A*0201 |
| 1I1Y | YLKEPVHGV | 2;9 | HLA-A*0201;HLA-A*0296;HLA-A*9221;HLA-A*0201;HLA-A*0296;HLA-A*9221 |
| 1I4F | GVYDGREHTV | 2;10 | HLA-A*0201;HLA-A*0203;HLA-A*0204;HLA-A*0207;HLA-A*0209;HLA-A*0212;HLA-A*0213;HLA-A*0216;HLA-A*0217;HLA-A*0218;HLA-A*0219;HLA-A*0222;HLA-A*0224;HLA-A*0225;HLA-A*0226;HLA-A*0227;HLA-A*0233;HLA-A*0236;HLA-A*0237;HLA-A*0238;HLA-A*0239;HLA-A*0240;HLA-A*0249;HLA-A*0252;HLA-A*0266;HLA-A*0289;HLA-A*0271;HLA-A*0268;HLA-A*0274;HLA-A*0258;HLA-A*0280;HLA-A*0265;HLA-A*0286;HLA-A*0277;HLA-A*0260;HLA-A*0273;HLA-A*0285;HLA-A*0264;HLA-A*0275;HLA-A*0267;HLA-A*0295;HLA-A*0297;HLA-A*9201;HLA-A*0296;HLA-A*0293;HLA-A*9221;HLA-A*9231;HLA-A*9232;HLA-A*9214;HLA-A*9202;HLA-A*9210;HLA-A*9205;HLA-A*9217;HLA-A*9211;HLA-A*9204;HLA-A*9230;HLA-A*9234;HLA-A*9218;HLA-A*9247;HLA-A*9249;HLA-A*9251;HLA-A*9248;HLA-A*9256;HLA-A*9235;HLA-A*9252;HLA-A*9240;HLA-A*9253;HLA-A*9245;HLA-A*9277;HLA-A*9257;HLA-A*9271;HLA-A*9294;HLA-A*9264;HLA-A*9290;HLA-A*9266;HLA-A*9291;HLA-A*9289;HLA-A*9274;HLA-A*9262;HLA-A*9259;HLA-A*9263;HLA-A*9268;HLA-A*9288;HLA-A*9293;HLA-A*9287;HLA-A*9283;HLA-A*9267;HLA-A*9265;HLA-A*9282;HLA-A*9297;HLA-A*9298;HLA-A*9299;HLA-A*0201;HLA-A*0209;HLA-A*0266;HLA-A*0289;HLA-A*0275;HLA-A*0297;HLA-A*0296;HLA-A*9221;HLA-A*9232;HLA-A*9234;HLA-A*9240;HLA-A*9299 |
| 1I7R | FAPGFFPYL | 2;9 | HLA-A*0201;HLA-A*0296;HLA-A*9221;HLA-A*0201;HLA-A*0296;HLA-A*9221 |
| 1I7T | ALWGVFPVL | 2;9 | HLA-A*0201;HLA-A*0201 |
| 1I7U | ALWGFVPVL | 2;9 | HLA-A*0201;HLA-A*0296;HLA-A*9221;HLA-A*0201;HLA-A*0296;HLA-A*9221 |
| 1IM3 | LLFGYPVYV | 2;9 | HLA-A*0201;HLA-A*0296;HLA-A*9221;HLA-A*0201;HLA-A*0296;HLA-A*9221 |
| 1IM9 | QYDDAVYKL | 2;9 | HLA-C*0401;HLA-C*0430;HLA-C*0433;HLA-C*0401;HLA-C*0430;HLA-C*0433 |
| 1INQ | SSVVGVWYL | 2;9 | H2-D1b;H2-D1b |
| 1JF1 | ELAGIGILTV | 2;10 | HLA-A*0201;HLA-A*0296;HLA-A*9221;HLA-A*0201;HLA-A*0296;HLA-A*9221 |
| 1JGD | RRLLRGHNQY | 2;10 | HLA-B*2709;HLA-B*2709 |
| 1JGE | GRFAAAIAK | 2;9 | HLA-B*2705;HLA-B*2713;HLA-B*2705;HLA-B*2713 |
| 1JHT | ALGIGILTV | 2;9 | HLA-A*0201;HLA-A*0296;HLA-A*9221;HLA-A*0201;HLA-A*0296;HLA-A*9221 |
| 1JPF | SGVENPGGYCL | 2;11 | H2-D1b;H2-D1b |
| 1JPG | FQPQNGQFI | 2;9 | H2-D1b;H2-D1b |
| 1JTR | EQYKFYSV | 2;8 | MH1-K1b;MH1-K1b |
| 1JUF | SSVIGVWYL | 2;9 | H2-D1b;H2-D1b |
| 1K5N | GRFAAAIAK | 2;9 | HLA-B*2709;HLA-B*2709 |
| 1K8D | ILMEHIHKL | 2;9 | H2-Q7*01;H2-Q7*01 |
| 1KJ2 | KVITFIDL | 2;8 | MH1-K1b;MH1-K1b |
| 1KJ3 | KVITFIDL | 2;8 | MH1-K1b;MH1-K1b |
| 1KJM | AQFSASASR | 2;9 | RT1-A;RT1-A |
| 1KJV | NPRAMQALL | 2;9 | RT1-A;RT1-A |
| 1KPR | VMAPRTVLL | 2;9 | HLA-E*0101;HLA-E*0103;HLA-E*0103 |
| 1KPU | RGYVYQGL | 2;8 | MH1-K1b;MH1-K1b |
| 1KPV | FAPGNYPAL | 2;9 | MH1-K1b;MH1-K1b |
| 1KTL | VTAPRTLLL | 2;9 | HLA-E*0103;HLA-E*0103 |
| 1L6Q | LYLVCGERG | 2;9 | MH1-K1*02;MH1-K1*02 |
| 1LD9 | YPNVNIHNF | 2;9 | H2-Ld-2;H2-L*03;H2-Ld-2 |
| 1LEG | EQYKFYSV | 2;8 | MH1-K1b;MH1-K1b |
| 1LEK | EQYKFYSV | 2;8 | MH1-K1b;MH1-K1b |
| 1LK2 | GNYSFYAL | 2;8 | MH1-K1b;MH1-K1b |
| 1LP9 | ALWGFFPVL | 2;9 | HLA-A*0201;HLA-A*0201 |
| 1M05 | FLRGRAYGL | 2;9 | HLA-B*0801;HLA-B*0801 |
| 1M6O | EEFGRAFSF | 2;9 | HLA-B*4402;HLA-B*4402 |
| 1MHE | VMAPRTVLL | 2;9 | HLA-E*0101;HLA-E*0101 |
| 1MI5 | FLRGRAYGL | 2;9 | HLA-B*0801;HLA-B*0807;HLA-B*0809;HLA-B*0811;HLA-B*0812;HLA-B*0813;HLA-B*0814;HLA-B*0827;HLA-B*0822;HLA-B*0820;HLA-B*0825;HLA-B*0821;HLA-B*0818;HLA-B*0828;HLA-B*0831;HLA-B*0833;HLA-B*0835;HLA-B*0837;HLA-B*0840;HLA-B*0839;HLA-B*0842;HLA-B*0843;HLA-B*0853;HLA-B*0858;HLA-B*0848;HLA-B*0849;HLA-B*0860;HLA-B*0855;HLA-B*9580;HLA-B*0801;HLA-B*0818;HLA-B*0839 |
| 1MWA | EQYKFYSV | 2;8 | MH1-K1b;MH1-K1b |
| 1N2R | EEFGRAFSF | 2;9 | HLA-B*4403;HLA-B*4403 |
| 1N3N | SALQNAASIA | 2;10 | H2-D1b;H2-D1b |
| 1N59 | AVYNFATM | 2;8 | MH1-K1b;MH1-K1b |
| 1N5A | KAVYNFATM | 2;9 | H2-D1b;H2-D1b |
| 1NAM | RGYVYQGL | 2;8 | MH1-K1b;MH1-K1b |
| 1NAN | INFDFNTI | 2;8 | MH1-K1b;MH1-K1b |
| 1OF2 | RRKWRRWHL | 2;9 | HLA-B*2705;HLA-B*2707;HLA-B*2709;HLA-B*2710;HLA-B*2713;HLA-B*2714;HLA-B*2719;HLA-B*2728;HLA-B*2727;HLA-B*2732;HLA-B*2735;HLA-B*2734;HLA-B*2738;HLA-B*2741;HLA-B*2745;HLA-B*2743;HLA-B*2750;HLA-B*2746;HLA-B*2755;HLA-B*2756;HLA-B*2747;HLA-B*2754;HLA-B*2758;HLA-B*2760;HLA-B*2709 |
| 1OGA | GILGFVFTL | 2;9 | HLA-A*0201;HLA-A*0201 |
| 1OGT | RRKWRRWHL | 2;9 | HLA-B*2705;HLA-B*2707;HLA-B*2709;HLA-B*2710;HLA-B*2713;HLA-B*2714;HLA-B*2719;HLA-B*2728;HLA-B*2727;HLA-B*2732;HLA-B*2735;HLA-B*2734;HLA-B*2738;HLA-B*2741;HLA-B*2745;HLA-B*2743;HLA-B*2750;HLA-B*2746;HLA-B*2755;HLA-B*2756;HLA-B*2747;HLA-B*2754;HLA-B*2758;HLA-B*2760;HLA-B*2705;HLA-B*2713 |
| 1OSZ | RGYLYQGL | 2;8 | MH1-K1b;MH1-K1b |
| 1P1Z | SIINFEKL | 2;8 | MH1-K1b;MH1-K1b |
| 1P4L | SIINFEKL | 2;8 | MH1-K1b;MH1-K1b |
| 1P7Q | ILKEPVHGV | 2;9 | HLA-A*0201;HLA-A*0201 |
| 1Q94 | AIFQSSMTK | 2;9 | HLA-A*1101;HLA-A*1101 |
| 1QEW | FLWGPRALV | 2;9 | HLA-A*0201;HLA-A*0296;HLA-A*9221;HLA-A*0201;HLA-A*0296;HLA-A*9221 |
| 1QLF | FAPSNYPAL | 2;9 | H2-D1b;H2-D1b |
| 1QO3 | RGPGRAFVTI | 2;10 | H2-D1*02;H2-D1*02 |
| 1QQD | QYDDAVYKL | 2;9 | HLA-C*0401;HLA-C*0430;HLA-C*0433;HLA-C*0401;HLA-C*0430;HLA-C*0433 |
| 1QR1 | IISAVVGIL | 2;9 | HLA-A*0201;HLA-A*0296;HLA-A*9221;HLA-A*0201;HLA-A*0296;HLA-A*9221 |
| 1QRN | LLFGYAVYV | 2;9 | HLA-A*0201;HLA-A*0203;HLA-A*0204;HLA-A*0207;HLA-A*0209;HLA-A*0212;HLA-A*0213;HLA-A*0216;HLA-A*0217;HLA-A*0218;HLA-A*0219;HLA-A*0222;HLA-A*0224;HLA-A*0225;HLA-A*0226;HLA-A*0227;HLA-A*0233;HLA-A*0236;HLA-A*0237;HLA-A*0238;HLA-A*0239;HLA-A*0240;HLA-A*0249;HLA-A*0252;HLA-A*0266;HLA-A*0289;HLA-A*0271;HLA-A*0268;HLA-A*0274;HLA-A*0258;HLA-A*0280;HLA-A*0265;HLA-A*0286;HLA-A*0277;HLA-A*0260;HLA-A*0273;HLA-A*0285;HLA-A*0264;HLA-A*0275;HLA-A*0267;HLA-A*0295;HLA-A*0297;HLA-A*9201;HLA-A*0296;HLA-A*0293;HLA-A*9221;HLA-A*9231;HLA-A*9232;HLA-A*9214;HLA-A*9202;HLA-A*9210;HLA-A*9205;HLA-A*9217;HLA-A*9211;HLA-A*9204;HLA-A*9230;HLA-A*9234;HLA-A*9218;HLA-A*9247;HLA-A*9249;HLA-A*9251;HLA-A*9248;HLA-A*9256;HLA-A*9235;HLA-A*9252;HLA-A*9240;HLA-A*9253;HLA-A*9245;HLA-A*9277;HLA-A*9257;HLA-A*9271;HLA-A*9294;HLA-A*9264;HLA-A*9290;HLA-A*9266;HLA-A*9291;HLA-A*9289;HLA-A*9274;HLA-A*9262;HLA-A*9259;HLA-A*9263;HLA-A*9268;HLA-A*9288;HLA-A*9293;HLA-A*9287;HLA-A*9283;HLA-A*9267;HLA-A*9265;HLA-A*9282;HLA-A*9297;HLA-A*9298;HLA-A*9299;HLA-A*0201;HLA-A*0209;HLA-A*0266;HLA-A*0289;HLA-A*0275;HLA-A*0297;HLA-A*0296;HLA-A*9221;HLA-A*9232;HLA-A*9234;HLA-A*9240;HLA-A*9299 |
| 1QSE | LLFGYPRYV | 2;9 | HLA-A*0201;HLA-A*0201 |
| 1QSF | LLFGYPVAV | 2;9 | HLA-A*0201;HLA-A*0203;HLA-A*0204;HLA-A*0207;HLA-A*0209;HLA-A*0212;HLA-A*0213;HLA-A*0216;HLA-A*0217;HLA-A*0218;HLA-A*0219;HLA-A*0222;HLA-A*0224;HLA-A*0225;HLA-A*0226;HLA-A*0227;HLA-A*0233;HLA-A*0236;HLA-A*0237;HLA-A*0238;HLA-A*0239;HLA-A*0240;HLA-A*0249;HLA-A*0252;HLA-A*0266;HLA-A*0289;HLA-A*0271;HLA-A*0268;HLA-A*0274;HLA-A*0258;HLA-A*0280;HLA-A*0265;HLA-A*0286;HLA-A*0277;HLA-A*0260;HLA-A*0273;HLA-A*0285;HLA-A*0264;HLA-A*0275;HLA-A*0267;HLA-A*0295;HLA-A*0297;HLA-A*9201;HLA-A*0296;HLA-A*0293;HLA-A*9221;HLA-A*9231;HLA-A*9232;HLA-A*9214;HLA-A*9202;HLA-A*9210;HLA-A*9205;HLA-A*9217;HLA-A*9211;HLA-A*9204;HLA-A*9230;HLA-A*9234;HLA-A*9218;HLA-A*9247;HLA-A*9249;HLA-A*9251;HLA-A*9248;HLA-A*9256;HLA-A*9235;HLA-A*9252;HLA-A*9240;HLA-A*9253;HLA-A*9245;HLA-A*9277;HLA-A*9257;HLA-A*9271;HLA-A*9294;HLA-A*9264;HLA-A*9290;HLA-A*9266;HLA-A*9291;HLA-A*9289;HLA-A*9274;HLA-A*9262;HLA-A*9259;HLA-A*9263;HLA-A*9268;HLA-A*9288;HLA-A*9293;HLA-A*9287;HLA-A*9283;HLA-A*9267;HLA-A*9265;HLA-A*9282;HLA-A*9297;HLA-A*9298;HLA-A*9299;HLA-A*0201;HLA-A*0209;HLA-A*0266;HLA-A*0289;HLA-A*0275;HLA-A*0297;HLA-A*0296;HLA-A*9221;HLA-A*9232;HLA-A*9234;HLA-A*9240;HLA-A*9299 |
| 1QVO | QVPLRPMTYK | 2;10 | HLA-A*1101;HLA-A*1101 |
| 1RJY | SSIEFARL | 2;8 | MH1-K1b;MH1-K1b |
| 1RJZ | SEIEFARL | 2;8 | MH1-K1b;MH1-K1b |
| 1RK0 | SSIEFARL | 2;8 | MH1-K1b;MH1-K1b |
| 1RK1 | SEIEFARL | 2;8 | MH1-K1b;MH1-K1b |
| 1S7Q | AVYNFATM | 2;8 | MH1-K1b;MH1-K1b |
| 1S7R | KAVYNLATM | 3;9 | MH1-K1b;MH1-K1b |
| 1S7S | ALYNFATM | 2;8 | MH1-K1b;MH1-K1b |
| 1S7T | AVFNFATM | 2;8 | MH1-K1b;MH1-K1b |
| 1S7U | KAVYNFATM | 2;9 | H2-D1b;H2-D1b |
| 1S7V | KAVYNLATM | 2;9 | H2-D1b;H2-D1b |
| 1S7W | KALYNFATM | 2;9 | H2-D1b;H2-D1b |
| 1S7X | KAVFNFATM | 2;9 | H2-D1b;H2-D1b |
| 1S8D | SLANTVATL | 2;9 | HLA-A*0201;HLA-A*0296;HLA-A*9221;HLA-A*0201;HLA-A*0296;HLA-A*9221 |
| 1S9W | SLLMWITQC | 2;9 | HLA-A*0201;HLA-A*0296;HLA-A*9221;HLA-A*0201;HLA-A*0296;HLA-A*9221 |
| 1S9X | SLLMWITQA | 2;9 | HLA-A*0201;HLA-A*0296;HLA-A*9221;HLA-A*0201;HLA-A*0296;HLA-A*9221 |
| 1S9Y | SLLMWITQS | 2;9 | HLA-A*0201;HLA-A*0296;HLA-A*9221;HLA-A*0201;HLA-A*0296;HLA-A*9221 |
| 1SYS | EEPTVIKKY | 2;9 | HLA-B*4403;HLA-B*4403 |
| 1SYV | EEFGRAFSF | 2;9 | HLA-B*4405;HLA-B*4405 |
| 1T0M | SSIEFARL | 2;8 | MH1-K1b;MH1-K1b |
| 1T0N | SSIEFARL | 2;8 | MH1-K1b;MH1-K1b |
| 1T1W | SLFNTIAVL | 2;9 | HLA-A*0201;HLA-A*0296;HLA-A*9221;HLA-A*0201;HLA-A*0296;HLA-A*9221 |
| 1T1X | SLYLTVATL | 2;9 | HLA-A*0201;HLA-A*0296;HLA-A*9221;HLA-A*0201;HLA-A*0296;HLA-A*9221 |
| 1T1Y | SLYNVVATL | 2;9 | HLA-A*0201;HLA-A*0296;HLA-A*9221;HLA-A*0201;HLA-A*0296;HLA-A*9221 |
| 1T1Z | ALYNTAAAL | 2;9 | HLA-A*0201;HLA-A*0296;HLA-A*9221;HLA-A*0201;HLA-A*0296;HLA-A*9221 |
| 1T20 | SLYNTIATL | 2;9 | HLA-A*0201;HLA-A*0296;HLA-A*9221;HLA-A*0201;HLA-A*0296;HLA-A*9221 |
| 1T21 | SLYNTVATL | 2;9 | HLA-A*0201;HLA-A*0296;HLA-A*9221;HLA-A*0201;HLA-A*0296;HLA-A*9221 |
| 1T22 | SLYNTVATL | 2;9 | HLA-A*0201;HLA-A*0296;HLA-A*9221;HLA-A*0201;HLA-A*0296;HLA-A*9221 |
| 1TVB | ITDQVPFSV | 2;9 | HLA-A*0201;HLA-A*0203;HLA-A*0204;HLA-A*0207;HLA-A*0209;HLA-A*0212;HLA-A*0213;HLA-A*0216;HLA-A*0217;HLA-A*0218;HLA-A*0219;HLA-A*0222;HLA-A*0224;HLA-A*0225;HLA-A*0226;HLA-A*0227;HLA-A*0233;HLA-A*0236;HLA-A*0237;HLA-A*0238;HLA-A*0239;HLA-A*0240;HLA-A*0249;HLA-A*0252;HLA-A*0266;HLA-A*0289;HLA-A*0271;HLA-A*0268;HLA-A*0274;HLA-A*0258;HLA-A*0280;HLA-A*0265;HLA-A*0286;HLA-A*0277;HLA-A*0260;HLA-A*0273;HLA-A*0285;HLA-A*0264;HLA-A*0275;HLA-A*0267;HLA-A*0295;HLA-A*0297;HLA-A*9201;HLA-A*0296;HLA-A*0293;HLA-A*9221;HLA-A*9231;HLA-A*9232;HLA-A*9214;HLA-A*9202;HLA-A*9210;HLA-A*9205;HLA-A*9217;HLA-A*9211;HLA-A*9204;HLA-A*9230;HLA-A*9234;HLA-A*9218;HLA-A*9247;HLA-A*9249;HLA-A*9251;HLA-A*9248;HLA-A*9256;HLA-A*9235;HLA-A*9252;HLA-A*9240;HLA-A*9253;HLA-A*9245;HLA-A*9277;HLA-A*9257;HLA-A*9271;HLA-A*9294;HLA-A*9264;HLA-A*9290;HLA-A*9266;HLA-A*9291;HLA-A*9289;HLA-A*9274;HLA-A*9262;HLA-A*9259;HLA-A*9263;HLA-A*9268;HLA-A*9288;HLA-A*9293;HLA-A*9287;HLA-A*9283;HLA-A*9267;HLA-A*9265;HLA-A*9282;HLA-A*9297;HLA-A*9298;HLA-A*9299;HLA-A*0201;HLA-A*0209;HLA-A*0266;HLA-A*0289;HLA-A*0275;HLA-A*0297;HLA-A*0296;HLA-A*9221;HLA-A*9232;HLA-A*9234;HLA-A*9240;HLA-A*9299 |
| 1TVH | IMDQVPFSV | 2;9 | HLA-A*0201;HLA-A*0203;HLA-A*0204;HLA-A*0207;HLA-A*0209;HLA-A*0212;HLA-A*0213;HLA-A*0216;HLA-A*0217;HLA-A*0218;HLA-A*0219;HLA-A*0222;HLA-A*0224;HLA-A*0225;HLA-A*0226;HLA-A*0227;HLA-A*0233;HLA-A*0236;HLA-A*0237;HLA-A*0238;HLA-A*0239;HLA-A*0240;HLA-A*0249;HLA-A*0252;HLA-A*0266;HLA-A*0289;HLA-A*0271;HLA-A*0268;HLA-A*0274;HLA-A*0258;HLA-A*0280;HLA-A*0265;HLA-A*0286;HLA-A*0277;HLA-A*0260;HLA-A*0273;HLA-A*0285;HLA-A*0264;HLA-A*0275;HLA-A*0267;HLA-A*0295;HLA-A*0297;HLA-A*9201;HLA-A*0296;HLA-A*0293;HLA-A*9221;HLA-A*9231;HLA-A*9232;HLA-A*9214;HLA-A*9202;HLA-A*9210;HLA-A*9205;HLA-A*9217;HLA-A*9211;HLA-A*9204;HLA-A*9230;HLA-A*9234;HLA-A*9218;HLA-A*9247;HLA-A*9249;HLA-A*9251;HLA-A*9248;HLA-A*9256;HLA-A*9235;HLA-A*9252;HLA-A*9240;HLA-A*9253;HLA-A*9245;HLA-A*9277;HLA-A*9257;HLA-A*9271;HLA-A*9294;HLA-A*9264;HLA-A*9290;HLA-A*9266;HLA-A*9291;HLA-A*9289;HLA-A*9274;HLA-A*9262;HLA-A*9259;HLA-A*9263;HLA-A*9268;HLA-A*9288;HLA-A*9293;HLA-A*9287;HLA-A*9283;HLA-A*9267;HLA-A*9265;HLA-A*9282;HLA-A*9297;HLA-A*9298;HLA-A*9299;HLA-A*0201;HLA-A*0209;HLA-A*0266;HLA-A*0289;HLA-A*0275;HLA-A*0297;HLA-A*0296;HLA-A*9221;HLA-A*9232;HLA-A*9234;HLA-A*9240;HLA-A*9299 |
| 1UXS | RRRWRRLTV | 2;9 | HLA-B*2705;HLA-B*2707;HLA-B*2709;HLA-B*2710;HLA-B*2713;HLA-B*2714;HLA-B*2719;HLA-B*2728;HLA-B*2727;HLA-B*2732;HLA-B*2735;HLA-B*2734;HLA-B*2738;HLA-B*2741;HLA-B*2745;HLA-B*2743;HLA-B*2750;HLA-B*2746;HLA-B*2755;HLA-B*2756;HLA-B*2747;HLA-B*2754;HLA-B*2758;HLA-B*2760;HLA-B*2705;HLA-B*2713 |
| 1VAC | SIINFEKL | 2;8 | MH1-K1b;MH1-K1b |
| 1VAD | SRDHSRTPM | 2;9 | MH1-K1b;MH1-K1b |
| 1VGK | SYVNTNMGL | 2;9 | MH1-K1*02;MH1-K1*02 |
| 1W0V | RRLPIFSRL | 2;9 | HLA-B*2705;HLA-B*2713;HLA-B*2705;HLA-B*2713 |
| 1W0W | RRLPIFSRL | 2;9 | HLA-B*2709;HLA-B*2709 |
| 1W72 | EADPTGHSY | 2;9 | HLA-A*0101;HLA-A*0103;HLA-A*0106;HLA-A*0108;HLA-A*3601;HLA-A*3602;HLA-A*3603;HLA-A*0110;HLA-A*0112;HLA-A*0114;HLA-A*3604;HLA-A*0119;HLA-A*0125;HLA-A*0121;HLA-A*0126;HLA-A*0130;HLA-A*0132;HLA-A*0137;HLA-A*0135;HLA-A*0138;HLA-A*0136;HLA-A*0142;HLA-A*0144;HLA-A*0145;HLA-A*0140;HLA-A*0139;HLA-A*0141;HLA-A*0155;HLA-A*0146;HLA-A*0154;HLA-A*0148;HLA-A*3605;HLA-A*0150;HLA-A*0149;HLA-A*0101;HLA-A*0132;HLA-A*0137;HLA-A*0145 |
| 1WBX | SQLKNNAKEI | 2;10 | H2-D1b;H2-D1b |
| 1WBY | SSLENFRAYV | 2;10 | H2-D1b;H2-D1b |
| 1WBZ | SSYRRPVGI | 2;9 | MH1-K1b;MH1-K1b |
| 1X7Q | KTFPPTEPK | 2;9 | HLA-A*1101;HLA-A*1103;HLA-A*1104;HLA-A*1105;HLA-A*1107;HLA-A*1108;HLA-A*1109;HLA-A*1112;HLA-A*1113;HLA-A*1120;HLA-A*0312;HLA-A*1122;HLA-A*1115;HLA-A*1123;HLA-A*0278;HLA-A*1126;HLA-A*1127;HLA-A*1125;HLA-A*1124;HLA-A*1132;HLA-A*1129;HLA-A*1130;HLA-A*1131;HLA-A*1133;HLA-A*1139;HLA-A*1135;HLA-A*1136;HLA-A*1134;HLA-A*1141;HLA-A*1144;HLA-A*1148;HLA-A*1150;HLA-A*1159;HLA-A*1154;HLA-A*1101;HLA-A*1112;HLA-A*1132 |
| 1XH3 | LPAVVGLSPGEQEY | 2;14 | HLA-B*3501;HLA-B*3542;HLA-B*3501;HLA-B*3542 |
| 1XR9 | ILGPPGSVY | 2;9 | HLA-B*1501;HLA-B*1501 |
| 1YDP | RIIPRHLQL | 2;9 | HLA-G*0104;HLA-G*0104 |
| 1YN6 | SSLENFRAYV | 2;10 | H2-D1b;H2-D1b |
| 1YN7 | SSLENFAAYV | 2;10 | H2-D1b;H2-D1b |
| 1ZHB | KALYNYAPI | 2;9 | H2-D1b;H2-D1b |
| 1ZHK | LPEPLPQGQLTAY | 2;13 | HLA-B*3501;HLA-B*3542;HLA-B*3501;HLA-B*3542 |
| 1ZHL | LPEPLPQGQLTAY | 2;13 | HLA-B*3508;HLA-B*3508 |
| 1ZSD | EPLPQGQLTAY | 2;11 | HLA-B*3501;HLA-B*3542;HLA-B*3501;HLA-B*3542 |
| 1ZT1 | FEANGNLI | 2;8 | MH1-K1k;MH1-K1k |
| 1ZT7 | SEFLLEKRI | 2;9 | MH1-K1k;MH1-K1k |
| 1ZVS | TTPESANL | 2;8 | MH1-A*01;MH1-A*01 |
| 2A83 | RRRWHRWRL | 2;9 | HLA-B*2705;HLA-B*2707;HLA-B*2709;HLA-B*2710;HLA-B*2713;HLA-B*2714;HLA-B*2719;HLA-B*2728;HLA-B*2727;HLA-B*2732;HLA-B*2735;HLA-B*2734;HLA-B*2738;HLA-B*2741;HLA-B*2745;HLA-B*2743;HLA-B*2750;HLA-B*2746;HLA-B*2755;HLA-B*2756;HLA-B*2747;HLA-B*2754;HLA-B*2758;HLA-B*2760;HLA-B*2705;HLA-B*2713 |
| 2AK4 | LPEPLPQGQLTAY | 2;13 | HLA-B*3501;HLA-B*3502;HLA-B*3503;HLA-B*3504;HLA-B*3505;HLA-B*3506;HLA-B*3508;HLA-B*3509;HLA-B*3512;HLA-B*3514;HLA-B*3515;HLA-B*3517;HLA-B*3518;HLA-B*3521;HLA-B*3522;HLA-B*3523;HLA-B*3524;HLA-B*3530;HLA-B*3531;HLA-B*3532;HLA-B*3533;HLA-B*3534;HLA-B*3535;HLA-B*3537;HLA-B*3538;HLA-B*3539;HLA-B*3541;HLA-B*7802;HLA-B*7804;HLA-B*3542;HLA-B*3565;HLA-B*3555;HLA-B*3560;HLA-B*3548;HLA-B*3559;HLA-B*3544;HLA-B*3558;HLA-B*3561;HLA-B*3551;HLA-B*3557;HLA-B*3545;HLA-B*3564;HLA-B*3567;HLA-B*3566;HLA-B*3562;HLA-B*3579;HLA-B*3575;HLA-B*3571;HLA-B*3570;HLA-B*3568;HLA-B*3583;HLA-B*3593;HLA-B*3587;HLA-B*3586;HLA-B*3581;HLA-B*3589;HLA-B*3591;HLA-B*3594;HLA-B*3588;HLA-B*3599;HLA-B*3596;HLA-B*3598;HLA-B*3508 |
| 2AV1 | LLFGYPVYV | 2;9 | HLA-A*0201;HLA-A*0203;HLA-A*0204;HLA-A*0207;HLA-A*0209;HLA-A*0212;HLA-A*0213;HLA-A*0216;HLA-A*0217;HLA-A*0218;HLA-A*0219;HLA-A*0220;HLA-A*0222;HLA-A*0224;HLA-A*0225;HLA-A*0226;HLA-A*0227;HLA-A*0229;HLA-A*0233;HLA-A*0236;HLA-A*0237;HLA-A*0238;HLA-A*0239;HLA-A*0240;HLA-A*0249;HLA-A*0252;HLA-A*0266;HLA-A*0289;HLA-A*0271;HLA-A*0268;HLA-A*0274;HLA-A*0258;HLA-A*0280;HLA-A*0265;HLA-A*0286;HLA-A*0277;HLA-A*0260;HLA-A*0273;HLA-A*0285;HLA-A*0264;HLA-A*0275;HLA-A*0267;HLA-A*0295;HLA-A*0297;HLA-A*9201;HLA-A*0296;HLA-A*0293;HLA-A*9221;HLA-A*9231;HLA-A*9232;HLA-A*9214;HLA-A*9202;HLA-A*9210;HLA-A*9205;HLA-A*9217;HLA-A*9211;HLA-A*9204;HLA-A*9230;HLA-A*9234;HLA-A*9218;HLA-A*9247;HLA-A*9249;HLA-A*9251;HLA-A*9248;HLA-A*9256;HLA-A*9235;HLA-A*9252;HLA-A*9240;HLA-A*9253;HLA-A*9245;HLA-A*9277;HLA-A*9257;HLA-A*9271;HLA-A*9294;HLA-A*9264;HLA-A*9290;HLA-A*9266;HLA-A*9291;HLA-A*9289;HLA-A*9274;HLA-A*9262;HLA-A*9259;HLA-A*9263;HLA-A*9268;HLA-A*9288;HLA-A*9293;HLA-A*9287;HLA-A*9283;HLA-A*9267;HLA-A*9265;HLA-A*9282;HLA-A*9297;HLA-A*9298;HLA-A*0201;HLA-A*0209;HLA-A*0220;HLA-A*0229;HLA-A*0266;HLA-A*0289;HLA-A*0275;HLA-A*0297;HLA-A*0296;HLA-A*9221;HLA-A*9232;HLA-A*9234;HLA-A*9240 |
| 2AV7 | LLFGYPVYV | 2;9 | HLA-A*0201;HLA-A*0203;HLA-A*0204;HLA-A*0207;HLA-A*0209;HLA-A*0212;HLA-A*0213;HLA-A*0216;HLA-A*0217;HLA-A*0218;HLA-A*0219;HLA-A*0220;HLA-A*0222;HLA-A*0224;HLA-A*0225;HLA-A*0226;HLA-A*0227;HLA-A*0229;HLA-A*0233;HLA-A*0236;HLA-A*0237;HLA-A*0238;HLA-A*0239;HLA-A*0240;HLA-A*0249;HLA-A*0252;HLA-A*0266;HLA-A*0289;HLA-A*0271;HLA-A*0268;HLA-A*0274;HLA-A*0258;HLA-A*0280;HLA-A*0265;HLA-A*0286;HLA-A*0277;HLA-A*0260;HLA-A*0273;HLA-A*0285;HLA-A*0264;HLA-A*0275;HLA-A*0267;HLA-A*0295;HLA-A*0297;HLA-A*9201;HLA-A*0296;HLA-A*0293;HLA-A*9221;HLA-A*9231;HLA-A*9232;HLA-A*9214;HLA-A*9202;HLA-A*9210;HLA-A*9205;HLA-A*9217;HLA-A*9211;HLA-A*9204;HLA-A*9230;HLA-A*9234;HLA-A*9218;HLA-A*9247;HLA-A*9249;HLA-A*9251;HLA-A*9248;HLA-A*9256;HLA-A*9235;HLA-A*9252;HLA-A*9240;HLA-A*9253;HLA-A*9245;HLA-A*9277;HLA-A*9257;HLA-A*9271;HLA-A*9294;HLA-A*9264;HLA-A*9290;HLA-A*9266;HLA-A*9291;HLA-A*9289;HLA-A*9274;HLA-A*9262;HLA-A*9259;HLA-A*9263;HLA-A*9268;HLA-A*9288;HLA-A*9293;HLA-A*9287;HLA-A*9283;HLA-A*9267;HLA-A*9265;HLA-A*9282;HLA-A*9297;HLA-A*9298;HLA-A*9299;HLA-A*0201;HLA-A*0209;HLA-A*0220;HLA-A*0229;HLA-A*0266;HLA-A*0289;HLA-A*0275;HLA-A*0297;HLA-A*0296;HLA-A*9221;HLA-A*9232;HLA-A*9234;HLA-A*9240;HLA-A*9299 |
| 2AXF | APQPAPENAY | 2;10 | HLA-B*3508;HLA-B*3508 |
| 2AXG | APQPAPENAY | 2;10 | HLA-B*3501;HLA-B*3542;HLA-B*3501;HLA-B*3542 |
| 2BCK | VYGFVRACL | 2;9 | HLA-A*2402;HLA-A*2402 |
| 2BNQ | SLLMWITQV | 2;9 | HLA-A*0201;HLA-A*0296;HLA-A*9221;HLA-A*0201;HLA-A*0296;HLA-A*9221 |
| 2BNR | SLLMWITQC | 2;9 | HLA-A*0201;HLA-A*0296;HLA-A*9221;HLA-A*0201;HLA-A*0296;HLA-A*9221 |
| 2BSR | RRIYDLIEL | 2;9 | HLA-B*2705;HLA-B*2713;HLA-B*2705;HLA-B*2713 |
| 2BSS | KRWIILGLNK | 2;10 | HLA-B*2705;HLA-B*2713;HLA-B*2705;HLA-B*2713 |
| 2BST | SRYWAIRTR | 2;9 | HLA-B*2705;HLA-B*2713;HLA-B*2705;HLA-B*2713 |
| 2BVO | KAFSPEVIPMF | 2;11 | HLA-B*5703;HLA-B*5703 |
| 2BVP | ISPRTLDAW | 2;9 | HLA-B*5701;HLA-B*5702;HLA-B*5703;HLA-B*5704;HLA-B*5705;HLA-B*5706;HLA-B*5707;HLA-B*5709;HLA-B*5711;HLA-B*5714;HLA-B*5713;HLA-B*5718;HLA-B*5717;HLA-B*5719;HLA-B*5725;HLA-B*5722;HLA-B*5721;HLA-B*5726;HLA-B*5724;HLA-B*5729;HLA-B*5730;HLA-B*5731;HLA-B*5727;HLA-B*5703 |
| 2BVQ | KAFSPEVIP | 2;8 | HLA-B*5701;HLA-B*5702;HLA-B*5703;HLA-B*5706;HLA-B*5711;HLA-B*5703 |
| 2C7U | SLFNTIAVL | 2;9 | HLA-A*0201;HLA-A*0296;HLA-A*9221;HLA-A*0201;HLA-A*0296;HLA-A*9221 |
| 2CIK | KPIVVLHGY | 2;9 | HLA-B*3501;HLA-B*3542;HLA-B*3501;HLA-B*3542 |
| 2CKB | EQYKFYSV | 2;8 | MH1-K1b;MH1-K1b |
| 2CLR | MLLSVPLLLG | 2;9 | HLA-A*0201;HLA-A*0201 |
| 2CLV | SQYYYNSL | 2;8 | MH1-K1b;MH1-K1b |
| 2CLZ | INFDFNTI | 2;8 | MH1-K1b;MH1-K1b |
| 2D31 | RIIPRHLQL | 2;9 | HLA-G*0101;HLA-G*0101 |
| 2DYP | RIIPRHLQL | 2;9 | HLA-G*0101;HLA-G*0101 |
| 2ESV | VMAPRTLIL | 2;9 | HLA-E*0101;HLA-E*0103;HLA-E*0101 |
| 2F53 | SLLMWITQC | 2;9 | HLA-A*0201;HLA-A*0201 |
| 2F54 | SLLMWITQC | 2;9 | HLA-A*0201;HLA-A*0296;HLA-A*9221;HLA-A*0201;HLA-A*0296;HLA-A*9221 |
| 2F74 | KAVYNFATM | 2;9 | H2-D1b;H2-D1b |
| 2FO4 | SAPDFRPL | 2;8 | MH1-K1b;MH1-K1b |
| 2FWO | TYQRTRALV | 2;9 | MH1-K1*02;MH1-K1*02 |
| 2FZ3 | HPVGEADYFEY | 2;11 | HLA-B*3508;HLA-B*3508 |
| 2GIT | LLFGKPVYV | 2;9 | HLA-A*0201;HLA-A*0296;HLA-A*9221;HLA-A*0201;HLA-A*0296;HLA-A*9221 |
| 2GJ6 | LLFGKPVYV | 2;9 | HLA-A*0201;HLA-A*0203;HLA-A*0204;HLA-A*0207;HLA-A*0209;HLA-A*0212;HLA-A*0213;HLA-A*0216;HLA-A*0217;HLA-A*0218;HLA-A*0219;HLA-A*0222;HLA-A*0224;HLA-A*0225;HLA-A*0226;HLA-A*0227;HLA-A*0233;HLA-A*0236;HLA-A*0237;HLA-A*0238;HLA-A*0239;HLA-A*0240;HLA-A*0249;HLA-A*0252;HLA-A*0266;HLA-A*0289;HLA-A*0271;HLA-A*0268;HLA-A*0274;HLA-A*0258;HLA-A*0280;HLA-A*0265;HLA-A*0286;HLA-A*0277;HLA-A*0260;HLA-A*0273;HLA-A*0285;HLA-A*0264;HLA-A*0275;HLA-A*0267;HLA-A*0295;HLA-A*0297;HLA-A*9201;HLA-A*0296;HLA-A*0293;HLA-A*9221;HLA-A*9231;HLA-A*9232;HLA-A*9214;HLA-A*9202;HLA-A*9210;HLA-A*9205;HLA-A*9217;HLA-A*9211;HLA-A*9204;HLA-A*9230;HLA-A*9234;HLA-A*9218;HLA-A*9247;HLA-A*9249;HLA-A*9251;HLA-A*9248;HLA-A*9256;HLA-A*9235;HLA-A*9252;HLA-A*9240;HLA-A*9253;HLA-A*9245;HLA-A*9277;HLA-A*9257;HLA-A*9271;HLA-A*9294;HLA-A*9264;HLA-A*9290;HLA-A*9266;HLA-A*9291;HLA-A*9289;HLA-A*9274;HLA-A*9262;HLA-A*9259;HLA-A*9263;HLA-A*9268;HLA-A*9288;HLA-A*9293;HLA-A*9287;HLA-A*9283;HLA-A*9267;HLA-A*9265;HLA-A*9282;HLA-A*9297;HLA-A*9298;HLA-A*9299;HLA-A*0201;HLA-A*0209;HLA-A*0266;HLA-A*0289;HLA-A*0275;HLA-A*0297;HLA-A*0296;HLA-A*9221;HLA-A*9232;HLA-A*9234;HLA-A*9240;HLA-A*9299 |
| 2GT9 | EAAGIGILTV | 2;10 | HLA-A*0201;HLA-A*0296;HLA-A*9221;HLA-A*0201;HLA-A*0296;HLA-A*9221 |
| 2GTW | LAGIGILTV | 1;9 | HLA-A*0201;HLA-A*0296;HLA-A*9221;HLA-A*0201;HLA-A*0296;HLA-A*9221 |
| 2GTZ | ALGIGILTV | 2;9 | HLA-A*0201;HLA-A*0203;HLA-A*0204;HLA-A*0207;HLA-A*0209;HLA-A*0212;HLA-A*0213;HLA-A*0216;HLA-A*0217;HLA-A*0218;HLA-A*0219;HLA-A*0222;HLA-A*0224;HLA-A*0225;HLA-A*0226;HLA-A*0227;HLA-A*0233;HLA-A*0236;HLA-A*0237;HLA-A*0238;HLA-A*0239;HLA-A*0240;HLA-A*0249;HLA-A*0252;HLA-A*0266;HLA-A*0289;HLA-A*0271;HLA-A*0268;HLA-A*0274;HLA-A*0258;HLA-A*0280;HLA-A*0265;HLA-A*0286;HLA-A*0277;HLA-A*0260;HLA-A*0273;HLA-A*0285;HLA-A*0264;HLA-A*0275;HLA-A*0267;HLA-A*0295;HLA-A*0297;HLA-A*9201;HLA-A*0296;HLA-A*0293;HLA-A*9221;HLA-A*9231;HLA-A*9232;HLA-A*9214;HLA-A*9202;HLA-A*9210;HLA-A*9205;HLA-A*9217;HLA-A*9211;HLA-A*9204;HLA-A*9230;HLA-A*9234;HLA-A*9218;HLA-A*9247;HLA-A*9249;HLA-A*9251;HLA-A*9248;HLA-A*9256;HLA-A*9235;HLA-A*9252;HLA-A*9240;HLA-A*9253;HLA-A*9245;HLA-A*9277;HLA-A*9257;HLA-A*9271;HLA-A*9294;HLA-A*9264;HLA-A*9290;HLA-A*9266;HLA-A*9291;HLA-A*9289;HLA-A*9274;HLA-A*9262;HLA-A*9259;HLA-A*9263;HLA-A*9268;HLA-A*9288;HLA-A*9293;HLA-A*9287;HLA-A*9283;HLA-A*9267;HLA-A*9265;HLA-A*9282;HLA-A*9297;HLA-A*9298;HLA-A*9299;HLA-A*0201;HLA-A*0209;HLA-A*0266;HLA-A*0289;HLA-A*0275;HLA-A*0297;HLA-A*0296;HLA-A*9221;HLA-A*9232;HLA-A*9234;HLA-A*9240;HLA-A*9299 |
| 2GUO | AAGIGILTV | 2;9 | HLA-A*0201;HLA-A*0203;HLA-A*0204;HLA-A*0207;HLA-A*0209;HLA-A*0212;HLA-A*0213;HLA-A*0216;HLA-A*0217;HLA-A*0218;HLA-A*0219;HLA-A*0222;HLA-A*0224;HLA-A*0225;HLA-A*0226;HLA-A*0227;HLA-A*0233;HLA-A*0236;HLA-A*0237;HLA-A*0238;HLA-A*0239;HLA-A*0240;HLA-A*0249;HLA-A*0252;HLA-A*0266;HLA-A*0289;HLA-A*0271;HLA-A*0268;HLA-A*0274;HLA-A*0258;HLA-A*0280;HLA-A*0265;HLA-A*0286;HLA-A*0277;HLA-A*0260;HLA-A*0273;HLA-A*0285;HLA-A*0264;HLA-A*0275;HLA-A*0267;HLA-A*0295;HLA-A*0297;HLA-A*9201;HLA-A*0296;HLA-A*0293;HLA-A*9221;HLA-A*9231;HLA-A*9232;HLA-A*9214;HLA-A*9202;HLA-A*9210;HLA-A*9205;HLA-A*9217;HLA-A*9211;HLA-A*9204;HLA-A*9230;HLA-A*9234;HLA-A*9218;HLA-A*9247;HLA-A*9249;HLA-A*9251;HLA-A*9248;HLA-A*9256;HLA-A*9235;HLA-A*9252;HLA-A*9240;HLA-A*9253;HLA-A*9245;HLA-A*9277;HLA-A*9257;HLA-A*9271;HLA-A*9294;HLA-A*9264;HLA-A*9290;HLA-A*9266;HLA-A*9291;HLA-A*9289;HLA-A*9274;HLA-A*9262;HLA-A*9259;HLA-A*9263;HLA-A*9268;HLA-A*9288;HLA-A*9293;HLA-A*9287;HLA-A*9283;HLA-A*9267;HLA-A*9265;HLA-A*9282;HLA-A*9297;HLA-A*9298;HLA-A*9299;HLA-A*0201;HLA-A*0209;HLA-A*0266;HLA-A*0289;HLA-A*0275;HLA-A*0297;HLA-A*0296;HLA-A*9221;HLA-A*9232;HLA-A*9234;HLA-A*9240;HLA-A*9299 |
| 2H6P | KPIVVLHGY | 2;9 | HLA-B*3501;HLA-B*3542;HLA-B*3501;HLA-B*3542 |
| 2HJK | KGFNPEVIPMF | 2;11 | HLA-B*5706;HLA-B*5706 |
| 2HJL | KAFNPEIIPMF | 2;11 | HLA-B*5706;HLA-B*5706 |
| 2HN7 | AIMPARFYPK | 2;10 | HLA-A*1101;HLA-A*1101 |
| 2J8U | ALWGFFPVL | 2;9 | HLA-A*0201;HLA-A*0201 |
| 2JCC | ALWGFFPVL | 2;9 | HLA-A*0201;HLA-A*0201 |
| 2MHA | RGYVYQGL | 2;8 | MH1-K1b;MH1-K1b |
| 2NW3 | EPLPQGQLTAY | 2;11 | HLA-B*3508;HLA-B*3508 |
| 2NX5 | EPLPQGQLTAY | 2;11 | HLA-B*3501;HLA-B*3542;HLA-B*3501;HLA-B*3542 |
| 2OL3 | SQYYYNSL | 2;8 | MH1-K1b;MH1-K1b |
| 2P5E | SLLMWITQC | 2;9 | HLA-A*0201;HLA-A*0203;HLA-A*0204;HLA-A*0207;HLA-A*0209;HLA-A*0212;HLA-A*0213;HLA-A*0216;HLA-A*0217;HLA-A*0218;HLA-A*0219;HLA-A*0222;HLA-A*0224;HLA-A*0225;HLA-A*0226;HLA-A*0227;HLA-A*0233;HLA-A*0236;HLA-A*0237;HLA-A*0238;HLA-A*0239;HLA-A*0240;HLA-A*0249;HLA-A*0252;HLA-A*0266;HLA-A*0289;HLA-A*0271;HLA-A*0268;HLA-A*0274;HLA-A*0258;HLA-A*0280;HLA-A*0265;HLA-A*0286;HLA-A*0277;HLA-A*0260;HLA-A*0273;HLA-A*0285;HLA-A*0264;HLA-A*0275;HLA-A*0267;HLA-A*0295;HLA-A*0297;HLA-A*9201;HLA-A*0296;HLA-A*0293;HLA-A*9221;HLA-A*9231;HLA-A*9232;HLA-A*9214;HLA-A*9202;HLA-A*9210;HLA-A*9205;HLA-A*9217;HLA-A*9211;HLA-A*9204;HLA-A*9230;HLA-A*9234;HLA-A*9218;HLA-A*9247;HLA-A*9249;HLA-A*9251;HLA-A*9248;HLA-A*9256;HLA-A*9235;HLA-A*9252;HLA-A*9240;HLA-A*9253;HLA-A*9245;HLA-A*9277;HLA-A*9257;HLA-A*9271;HLA-A*9294;HLA-A*9264;HLA-A*9290;HLA-A*9266;HLA-A*9291;HLA-A*9289;HLA-A*9274;HLA-A*9262;HLA-A*9259;HLA-A*9263;HLA-A*9268;HLA-A*9288;HLA-A*9293;HLA-A*9287;HLA-A*9283;HLA-A*9267;HLA-A*9265;HLA-A*9282;HLA-A*9297;HLA-A*9298;HLA-A*9299;HLA-A*0201;HLA-A*0209;HLA-A*0266;HLA-A*0289;HLA-A*0275;HLA-A*0297;HLA-A*0296;HLA-A*9221;HLA-A*9232;HLA-A*9234;HLA-A*9240;HLA-A*9299 |
| 2P5W | SLLMWITQC | 2;9 | HLA-A*0201;HLA-A*0203;HLA-A*0204;HLA-A*0207;HLA-A*0209;HLA-A*0212;HLA-A*0213;HLA-A*0216;HLA-A*0217;HLA-A*0218;HLA-A*0219;HLA-A*0222;HLA-A*0224;HLA-A*0225;HLA-A*0226;HLA-A*0227;HLA-A*0233;HLA-A*0236;HLA-A*0237;HLA-A*0238;HLA-A*0239;HLA-A*0240;HLA-A*0249;HLA-A*0252;HLA-A*0266;HLA-A*0289;HLA-A*0271;HLA-A*0268;HLA-A*0274;HLA-A*0258;HLA-A*0280;HLA-A*0265;HLA-A*0286;HLA-A*0277;HLA-A*0260;HLA-A*0273;HLA-A*0285;HLA-A*0264;HLA-A*0275;HLA-A*0267;HLA-A*0295;HLA-A*0297;HLA-A*9201;HLA-A*0296;HLA-A*0293;HLA-A*9221;HLA-A*9231;HLA-A*9232;HLA-A*9214;HLA-A*9202;HLA-A*9210;HLA-A*9205;HLA-A*9217;HLA-A*9211;HLA-A*9204;HLA-A*9230;HLA-A*9234;HLA-A*9218;HLA-A*9247;HLA-A*9249;HLA-A*9251;HLA-A*9248;HLA-A*9256;HLA-A*9235;HLA-A*9252;HLA-A*9240;HLA-A*9253;HLA-A*9245;HLA-A*9277;HLA-A*9257;HLA-A*9271;HLA-A*9294;HLA-A*9264;HLA-A*9290;HLA-A*9266;HLA-A*9291;HLA-A*9289;HLA-A*9274;HLA-A*9262;HLA-A*9259;HLA-A*9263;HLA-A*9268;HLA-A*9288;HLA-A*9293;HLA-A*9287;HLA-A*9283;HLA-A*9267;HLA-A*9265;HLA-A*9282;HLA-A*9297;HLA-A*9298;HLA-A*9299;HLA-A*0201;HLA-A*0209;HLA-A*0266;HLA-A*0289;HLA-A*0275;HLA-A*0297;HLA-A*0296;HLA-A*9221;HLA-A*9232;HLA-A*9234;HLA-A*9240;HLA-A*9299 |
| 2PYE | SLLMWITQC | 2;9 | HLA-A*0201;HLA-A*0203;HLA-A*0204;HLA-A*0207;HLA-A*0209;HLA-A*0212;HLA-A*0213;HLA-A*0216;HLA-A*0217;HLA-A*0218;HLA-A*0219;HLA-A*0222;HLA-A*0224;HLA-A*0225;HLA-A*0226;HLA-A*0227;HLA-A*0233;HLA-A*0236;HLA-A*0237;HLA-A*0238;HLA-A*0239;HLA-A*0240;HLA-A*0249;HLA-A*0252;HLA-A*0266;HLA-A*0289;HLA-A*0271;HLA-A*0268;HLA-A*0274;HLA-A*0258;HLA-A*0280;HLA-A*0265;HLA-A*0286;HLA-A*0277;HLA-A*0260;HLA-A*0273;HLA-A*0285;HLA-A*0264;HLA-A*0275;HLA-A*0267;HLA-A*0295;HLA-A*0297;HLA-A*9201;HLA-A*0296;HLA-A*0293;HLA-A*9221;HLA-A*9231;HLA-A*9232;HLA-A*9214;HLA-A*9202;HLA-A*9210;HLA-A*9205;HLA-A*9217;HLA-A*9211;HLA-A*9204;HLA-A*9230;HLA-A*9234;HLA-A*9218;HLA-A*9247;HLA-A*9249;HLA-A*9251;HLA-A*9248;HLA-A*9256;HLA-A*9235;HLA-A*9252;HLA-A*9240;HLA-A*9253;HLA-A*9245;HLA-A*9277;HLA-A*9257;HLA-A*9271;HLA-A*9294;HLA-A*9264;HLA-A*9290;HLA-A*9266;HLA-A*9291;HLA-A*9289;HLA-A*9274;HLA-A*9262;HLA-A*9259;HLA-A*9263;HLA-A*9268;HLA-A*9288;HLA-A*9293;HLA-A*9287;HLA-A*9283;HLA-A*9267;HLA-A*9265;HLA-A*9282;HLA-A*9297;HLA-A*9298;HLA-A*9299;HLA-A*0201;HLA-A*0209;HLA-A*0266;HLA-A*0289;HLA-A*0275;HLA-A*0297;HLA-A*0296;HLA-A*9221;HLA-A*9232;HLA-A*9234;HLA-A*9240;HLA-A*9299 |
| 2RFX | LSSPVTKSF | 2;9 | HLA-B*5701;HLA-B*5701 |
| 2UWE | ALWGFFPVL | 2;9 | HLA-A*0201;HLA-A*0201 |
| 2V2W | SLYNTVATL | 2;9 | HLA-A*0201;HLA-A*0201 |
| 2V2X | SLFNTVATL | 2;9 | HLA-A*0201;HLA-A*0201 |
| 2VAA | RGYVYQGL | 2;8 | MH1-K1b;MH1-K1b |
| 2VAB | FAPGNYPAL | 2;9 | MH1-K1b;MH1-K1b |
| 2VLJ | GILGFVFTL | 2;9 | HLA-A*0201;HLA-A*0203;HLA-A*0204;HLA-A*0207;HLA-A*0209;HLA-A*0212;HLA-A*0213;HLA-A*0216;HLA-A*0217;HLA-A*0218;HLA-A*0219;HLA-A*0222;HLA-A*0224;HLA-A*0225;HLA-A*0226;HLA-A*0227;HLA-A*0233;HLA-A*0236;HLA-A*0237;HLA-A*0238;HLA-A*0239;HLA-A*0240;HLA-A*0249;HLA-A*0252;HLA-A*0266;HLA-A*0289;HLA-A*0271;HLA-A*0268;HLA-A*0274;HLA-A*0258;HLA-A*0280;HLA-A*0265;HLA-A*0286;HLA-A*0277;HLA-A*0260;HLA-A*0273;HLA-A*0285;HLA-A*0264;HLA-A*0275;HLA-A*0267;HLA-A*0295;HLA-A*0297;HLA-A*9201;HLA-A*0296;HLA-A*0293;HLA-A*9221;HLA-A*9231;HLA-A*9232;HLA-A*9214;HLA-A*9202;HLA-A*9210;HLA-A*9205;HLA-A*9217;HLA-A*9211;HLA-A*9204;HLA-A*9230;HLA-A*9234;HLA-A*9218;HLA-A*9247;HLA-A*9249;HLA-A*9251;HLA-A*9248;HLA-A*9256;HLA-A*9235;HLA-A*9252;HLA-A*9240;HLA-A*9253;HLA-A*9245;HLA-A*9277;HLA-A*9257;HLA-A*9271;HLA-A*9294;HLA-A*9264;HLA-A*9290;HLA-A*9266;HLA-A*9291;HLA-A*9289;HLA-A*9274;HLA-A*9262;HLA-A*9259;HLA-A*9263;HLA-A*9268;HLA-A*9288;HLA-A*9293;HLA-A*9287;HLA-A*9283;HLA-A*9267;HLA-A*9265;HLA-A*9282;HLA-A*9297;HLA-A*9298;HLA-A*9299;HLA-A*0201;HLA-A*0209;HLA-A*0266;HLA-A*0289;HLA-A*0275;HLA-A*0297;HLA-A*0296;HLA-A*9221;HLA-A*9232;HLA-A*9234;HLA-A*9240;HLA-A*9299 |
| 2VLK | GILGFVFTL | 2;9 | HLA-A*0201;HLA-A*0203;HLA-A*0204;HLA-A*0207;HLA-A*0209;HLA-A*0212;HLA-A*0213;HLA-A*0216;HLA-A*0217;HLA-A*0218;HLA-A*0219;HLA-A*0222;HLA-A*0224;HLA-A*0225;HLA-A*0226;HLA-A*0227;HLA-A*0233;HLA-A*0236;HLA-A*0237;HLA-A*0238;HLA-A*0239;HLA-A*0240;HLA-A*0249;HLA-A*0252;HLA-A*0266;HLA-A*0289;HLA-A*0271;HLA-A*0268;HLA-A*0274;HLA-A*0258;HLA-A*0280;HLA-A*0265;HLA-A*0286;HLA-A*0277;HLA-A*0260;HLA-A*0273;HLA-A*0285;HLA-A*0264;HLA-A*0275;HLA-A*0267;HLA-A*0295;HLA-A*0297;HLA-A*9201;HLA-A*0296;HLA-A*0293;HLA-A*9221;HLA-A*9231;HLA-A*9232;HLA-A*9214;HLA-A*9202;HLA-A*9210;HLA-A*9205;HLA-A*9217;HLA-A*9211;HLA-A*9204;HLA-A*9230;HLA-A*9234;HLA-A*9218;HLA-A*9247;HLA-A*9249;HLA-A*9251;HLA-A*9248;HLA-A*9256;HLA-A*9235;HLA-A*9252;HLA-A*9240;HLA-A*9253;HLA-A*9245;HLA-A*9277;HLA-A*9257;HLA-A*9271;HLA-A*9294;HLA-A*9264;HLA-A*9290;HLA-A*9266;HLA-A*9291;HLA-A*9289;HLA-A*9274;HLA-A*9262;HLA-A*9259;HLA-A*9263;HLA-A*9268;HLA-A*9288;HLA-A*9293;HLA-A*9287;HLA-A*9283;HLA-A*9267;HLA-A*9265;HLA-A*9282;HLA-A*9297;HLA-A*9298;HLA-A*9299;HLA-A*0201;HLA-A*0209;HLA-A*0266;HLA-A*0289;HLA-A*0275;HLA-A*0297;HLA-A*0296;HLA-A*9221;HLA-A*9232;HLA-A*9234;HLA-A*9240;HLA-A*9299 |
| 2VLL | GILGFVFTL | 2;9 | HLA-A*0201;HLA-A*0201 |
| 2VLR | GILGFVFTL | 2;9 | HLA-A*0201;HLA-A*0203;HLA-A*0204;HLA-A*0207;HLA-A*0209;HLA-A*0212;HLA-A*0213;HLA-A*0216;HLA-A*0217;HLA-A*0218;HLA-A*0219;HLA-A*0222;HLA-A*0224;HLA-A*0225;HLA-A*0226;HLA-A*0227;HLA-A*0233;HLA-A*0236;HLA-A*0237;HLA-A*0238;HLA-A*0239;HLA-A*0240;HLA-A*0249;HLA-A*0252;HLA-A*0266;HLA-A*0289;HLA-A*0271;HLA-A*0268;HLA-A*0274;HLA-A*0258;HLA-A*0280;HLA-A*0265;HLA-A*0286;HLA-A*0277;HLA-A*0260;HLA-A*0273;HLA-A*0285;HLA-A*0264;HLA-A*0275;HLA-A*0267;HLA-A*0295;HLA-A*0297;HLA-A*9201;HLA-A*0296;HLA-A*0293;HLA-A*9221;HLA-A*9231;HLA-A*9232;HLA-A*9214;HLA-A*9202;HLA-A*9210;HLA-A*9205;HLA-A*9217;HLA-A*9211;HLA-A*9204;HLA-A*9230;HLA-A*9234;HLA-A*9218;HLA-A*9247;HLA-A*9249;HLA-A*9251;HLA-A*9248;HLA-A*9256;HLA-A*9235;HLA-A*9252;HLA-A*9240;HLA-A*9253;HLA-A*9245;HLA-A*9277;HLA-A*9257;HLA-A*9271;HLA-A*9294;HLA-A*9264;HLA-A*9290;HLA-A*9266;HLA-A*9291;HLA-A*9289;HLA-A*9274;HLA-A*9262;HLA-A*9259;HLA-A*9263;HLA-A*9268;HLA-A*9288;HLA-A*9293;HLA-A*9287;HLA-A*9283;HLA-A*9267;HLA-A*9265;HLA-A*9282;HLA-A*9297;HLA-A*9298;HLA-A*9299;HLA-A*0201;HLA-A*0209;HLA-A*0266;HLA-A*0289;HLA-A*0275;HLA-A*0297;HLA-A*0296;HLA-A*9221;HLA-A*9232;HLA-A*9234;HLA-A*9240;HLA-A*9299 |
| 2X4O | KLTPLCVTL | 2;9 | HLA-A*0201;HLA-A*0203;HLA-A*0204;HLA-A*0207;HLA-A*0209;HLA-A*0212;HLA-A*0213;HLA-A*0216;HLA-A*0217;HLA-A*0218;HLA-A*0219;HLA-A*0222;HLA-A*0224;HLA-A*0225;HLA-A*0226;HLA-A*0227;HLA-A*0233;HLA-A*0236;HLA-A*0237;HLA-A*0238;HLA-A*0239;HLA-A*0240;HLA-A*0249;HLA-A*0252;HLA-A*0266;HLA-A*0289;HLA-A*0271;HLA-A*0268;HLA-A*0274;HLA-A*0258;HLA-A*0280;HLA-A*0265;HLA-A*0286;HLA-A*0277;HLA-A*0260;HLA-A*0273;HLA-A*0285;HLA-A*0264;HLA-A*0275;HLA-A*0267;HLA-A*0295;HLA-A*0297;HLA-A*9201;HLA-A*0296;HLA-A*0293;HLA-A*9221;HLA-A*9231;HLA-A*9232;HLA-A*9214;HLA-A*9202;HLA-A*9210;HLA-A*9205;HLA-A*9217;HLA-A*9211;HLA-A*9204;HLA-A*9230;HLA-A*9234;HLA-A*9218;HLA-A*9247;HLA-A*9249;HLA-A*9251;HLA-A*9248;HLA-A*9256;HLA-A*9235;HLA-A*9252;HLA-A*9240;HLA-A*9253;HLA-A*9245;HLA-A*9277;HLA-A*9257;HLA-A*9271;HLA-A*9294;HLA-A*9264;HLA-A*9290;HLA-A*9266;HLA-A*9291;HLA-A*9289;HLA-A*9274;HLA-A*9262;HLA-A*9259;HLA-A*9263;HLA-A*9268;HLA-A*9288;HLA-A*9293;HLA-A*9287;HLA-A*9283;HLA-A*9267;HLA-A*9265;HLA-A*9282;HLA-A*9297;HLA-A*9298;HLA-A*9299;HLA-A*0201;HLA-A*0209;HLA-A*0266;HLA-A*0289;HLA-A*0275;HLA-A*0297;HLA-A*0296;HLA-A*9221;HLA-A*9232;HLA-A*9234;HLA-A*9240;HLA-A*9299 |
| 2X4R | NLVPMVATV | 2;9 | HLA-A*0201;HLA-A*0203;HLA-A*0204;HLA-A*0207;HLA-A*0209;HLA-A*0212;HLA-A*0213;HLA-A*0216;HLA-A*0217;HLA-A*0218;HLA-A*0219;HLA-A*0222;HLA-A*0224;HLA-A*0225;HLA-A*0226;HLA-A*0227;HLA-A*0233;HLA-A*0236;HLA-A*0237;HLA-A*0238;HLA-A*0239;HLA-A*0240;HLA-A*0249;HLA-A*0252;HLA-A*0266;HLA-A*0289;HLA-A*0271;HLA-A*0268;HLA-A*0274;HLA-A*0258;HLA-A*0280;HLA-A*0265;HLA-A*0286;HLA-A*0277;HLA-A*0260;HLA-A*0273;HLA-A*0285;HLA-A*0264;HLA-A*0275;HLA-A*0267;HLA-A*0295;HLA-A*0297;HLA-A*9201;HLA-A*0296;HLA-A*0293;HLA-A*9221;HLA-A*9231;HLA-A*9232;HLA-A*9214;HLA-A*9202;HLA-A*9210;HLA-A*9205;HLA-A*9217;HLA-A*9211;HLA-A*9204;HLA-A*9230;HLA-A*9234;HLA-A*9218;HLA-A*9247;HLA-A*9249;HLA-A*9251;HLA-A*9248;HLA-A*9256;HLA-A*9235;HLA-A*9252;HLA-A*9240;HLA-A*9253;HLA-A*9245;HLA-A*9277;HLA-A*9257;HLA-A*9271;HLA-A*9294;HLA-A*9264;HLA-A*9290;HLA-A*9266;HLA-A*9291;HLA-A*9289;HLA-A*9274;HLA-A*9262;HLA-A*9259;HLA-A*9263;HLA-A*9268;HLA-A*9288;HLA-A*9293;HLA-A*9287;HLA-A*9283;HLA-A*9267;HLA-A*9265;HLA-A*9282;HLA-A*9297;HLA-A*9298;HLA-A*9299;HLA-A*0201;HLA-A*0209;HLA-A*0266;HLA-A*0289;HLA-A*0275;HLA-A*0297;HLA-A*0296;HLA-A*9221;HLA-A*9232;HLA-A*9234;HLA-A*9240;HLA-A*9299 |
| 2X4S | AMDSNTLEL | 2;9 | HLA-A*0201;HLA-A*0203;HLA-A*0204;HLA-A*0207;HLA-A*0209;HLA-A*0212;HLA-A*0213;HLA-A*0216;HLA-A*0217;HLA-A*0218;HLA-A*0219;HLA-A*0222;HLA-A*0224;HLA-A*0225;HLA-A*0226;HLA-A*0227;HLA-A*0233;HLA-A*0236;HLA-A*0237;HLA-A*0238;HLA-A*0239;HLA-A*0240;HLA-A*0249;HLA-A*0252;HLA-A*0266;HLA-A*0289;HLA-A*0271;HLA-A*0268;HLA-A*0274;HLA-A*0258;HLA-A*0280;HLA-A*0265;HLA-A*0286;HLA-A*0277;HLA-A*0260;HLA-A*0273;HLA-A*0285;HLA-A*0264;HLA-A*0275;HLA-A*0267;HLA-A*0295;HLA-A*0297;HLA-A*9201;HLA-A*0296;HLA-A*0293;HLA-A*9221;HLA-A*9231;HLA-A*9232;HLA-A*9214;HLA-A*9202;HLA-A*9210;HLA-A*9205;HLA-A*9217;HLA-A*9211;HLA-A*9204;HLA-A*9230;HLA-A*9234;HLA-A*9218;HLA-A*9247;HLA-A*9249;HLA-A*9251;HLA-A*9248;HLA-A*9256;HLA-A*9235;HLA-A*9252;HLA-A*9240;HLA-A*9253;HLA-A*9245;HLA-A*9277;HLA-A*9257;HLA-A*9271;HLA-A*9294;HLA-A*9264;HLA-A*9290;HLA-A*9266;HLA-A*9291;HLA-A*9289;HLA-A*9274;HLA-A*9262;HLA-A*9259;HLA-A*9263;HLA-A*9268;HLA-A*9288;HLA-A*9293;HLA-A*9287;HLA-A*9283;HLA-A*9267;HLA-A*9265;HLA-A*9282;HLA-A*9297;HLA-A*9298;HLA-A*9299;HLA-A*0201;HLA-A*0209;HLA-A*0266;HLA-A*0289;HLA-A*0275;HLA-A*0297;HLA-A*0296;HLA-A*9221;HLA-A*9232;HLA-A*9234;HLA-A*9240;HLA-A*9299 |
| 2X4U | ILKEPVHGV | 2;9 | HLA-A*0201;HLA-A*0203;HLA-A*0204;HLA-A*0207;HLA-A*0209;HLA-A*0212;HLA-A*0213;HLA-A*0216;HLA-A*0217;HLA-A*0218;HLA-A*0219;HLA-A*0222;HLA-A*0224;HLA-A*0225;HLA-A*0226;HLA-A*0227;HLA-A*0233;HLA-A*0236;HLA-A*0237;HLA-A*0238;HLA-A*0239;HLA-A*0240;HLA-A*0249;HLA-A*0252;HLA-A*0266;HLA-A*0289;HLA-A*0271;HLA-A*0268;HLA-A*0274;HLA-A*0258;HLA-A*0280;HLA-A*0265;HLA-A*0286;HLA-A*0277;HLA-A*0260;HLA-A*0273;HLA-A*0285;HLA-A*0264;HLA-A*0275;HLA-A*0267;HLA-A*0295;HLA-A*0297;HLA-A*9201;HLA-A*0296;HLA-A*0293;HLA-A*9221;HLA-A*9231;HLA-A*9232;HLA-A*9214;HLA-A*9202;HLA-A*9210;HLA-A*9205;HLA-A*9217;HLA-A*9211;HLA-A*9204;HLA-A*9230;HLA-A*9234;HLA-A*9218;HLA-A*9247;HLA-A*9249;HLA-A*9251;HLA-A*9248;HLA-A*9256;HLA-A*9235;HLA-A*9252;HLA-A*9240;HLA-A*9253;HLA-A*9245;HLA-A*9277;HLA-A*9257;HLA-A*9271;HLA-A*9294;HLA-A*9264;HLA-A*9290;HLA-A*9266;HLA-A*9291;HLA-A*9289;HLA-A*9274;HLA-A*9262;HLA-A*9259;HLA-A*9263;HLA-A*9268;HLA-A*9288;HLA-A*9293;HLA-A*9287;HLA-A*9283;HLA-A*9267;HLA-A*9265;HLA-A*9282;HLA-A*9297;HLA-A*9298;HLA-A*9299;HLA-A*0201;HLA-A*0209;HLA-A*0266;HLA-A*0289;HLA-A*0275;HLA-A*0297;HLA-A*0296;HLA-A*9221;HLA-A*9232;HLA-A*9234;HLA-A*9240;HLA-A*9299 |
| 2XFX | VGYPKVKEEML | 2;11 | MH1-N*01301;MH1-N*01301 |
| 2XPG | KLIETYFSK | 2;9 | HLA-A*0301;HLA-A*0301 |
| 2YEZ | TNPESKVFYL | 2;10 | MH1-B*2101;MH1-B*2101 |
| 2YF5 | TAGQSNYDRL | 2;10 | MH1-B*2101;MH1-B*2101 |
| 2YF6 | TAGQEDYDRL | 2;10 | MH1-B*2101;MH1-B*2101 |
| 2YPK | KAFSPEVIPMF | 2;11 | HLA-B*5701;HLA-B*5701 |
| 2YPL | KAFSPEVIPMF | 2;11 | HLA-B*5701;HLA-B*5702;HLA-B*5703;HLA-B*5704;HLA-B*5705;HLA-B*5706;HLA-B*5707;HLA-B*5709;HLA-B*5711;HLA-B*5714;HLA-B*5713;HLA-B*5718;HLA-B*5717;HLA-B*5719;HLA-B*5725;HLA-B*5722;HLA-B*5721;HLA-B*5726;HLA-B*5724;HLA-B*5729;HLA-B*5730;HLA-B*5731;HLA-B*5727;HLA-B*5706 |
| 3AM8 | VMGPRTLIL | 2;9 | HLA-E*0101;HLA-E*0101 |
| 3BEV | GHAEEYGAETL | 2;11 | MH1-B*2101;MH1-B*2101 |
| 3BEW | REVDEQLLSV | 2;10 | MH1-B*2101;MH1-B*2101 |
| 3BGM | RQASLSISV | 2;9 | HLA-A*0201;HLA-A*0296;HLA-A*9221;HLA-A*0201;HLA-A*0296;HLA-A*9221 |
| 3BH8 | RQASIELPSM | 2;10 | HLA-A*0201;HLA-A*0296;HLA-A*9221;HLA-A*0201;HLA-A*0296;HLA-A*9221 |
| 3BH9 | RTYSGPMNKV | 2;10 | HLA-A*0201;HLA-A*0296;HLA-A*9221;HLA-A*0201;HLA-A*0296;HLA-A*9221 |
| 3BHB | KMDSFLDMQL | 2;10 | HLA-A*0201;HLA-A*0296;HLA-A*9221;HLA-A*0201;HLA-A*0296;HLA-A*9221 |
| 3BO8 | EADPTGHSY | 2;9 | HLA-A*0101;HLA-A*0101 |
| 3BP4 | IRAAPPPLF | 2;9 | HLA-B*2705;HLA-B*2713;HLA-B*2705;HLA-B*2713 |
| 3BP7 | IRAAPPPLF | 2;9 | HLA-B*2709;HLA-B*2709 |
| 3BUY | LSLRNPILV | 2;9 | H2-D1b;H2-D1b |
| 3BVN | RRRWRRLTV | 2;9 | HLA-B*1402;HLA-B*1402 |
| 3BW9 | CPSQEPMSIYVY | 2;12 | HLA-B*3508;HLA-B*3508 |
| 3BWA | FPTKDVAL | 2;8 | HLA-B*3508;HLA-B*3508 |
| 3BXN | IRAAPPPLF | 2;9 | HLA-B*1402;HLA-B*1402 |
| 3BZE | VMAPRTLFL | 2;9 | HLA-E*0101;HLA-E*0101 |
| 3BZF | VMAPRALLL | 2;9 | HLA-E*0101;HLA-E*0101 |
| 3C8K | SIINFEKL | 2;8 | MH1-K1b;MH1-K1b |
| 3C9N | VQQESSFVM | 2;9 | HLA-B*1501;HLA-B*1501 |
| 3CC5 | KVPRNQDWL | 2;9 | H2-D1b;H2-D1b |
| 3CCH | EGSRNQDWL | 2;9 | H2-D1b;H2-D1b |
| 3CDG | VMAPRTLFL | 2;9 | HLA-E*0101;HLA-E*0103;HLA-E*0101 |
| 3CH1 | EGPRNQDWL | 2;9 | H2-D1b;H2-D1b |
| 3CII | VMAPRTLFL | 2;9 | HLA-E*0101;HLA-E*0103;HLA-E*0101 |
| 3CPL | ASNENAETM | 2;9 | H2-D1b;H2-D1b |
| 3CVH | SIINFEKL | 2;8 | MH1-K1b;MH1-K1b |
| 3CZF | RRRWHRWRL | 2;9 | HLA-B*2709;HLA-B*2709 |
| 3D25 | VLHDDLLEA | 2;9 | HLA-A*0201;HLA-A*0296;HLA-A*9221;HLA-A*0201;HLA-A*0296;HLA-A*9221 |
| 3D3V | LLFGFPVYV | 2;9 | HLA-A*0201;HLA-A*0203;HLA-A*0204;HLA-A*0207;HLA-A*0209;HLA-A*0212;HLA-A*0213;HLA-A*0216;HLA-A*0217;HLA-A*0218;HLA-A*0219;HLA-A*0222;HLA-A*0224;HLA-A*0225;HLA-A*0226;HLA-A*0227;HLA-A*0233;HLA-A*0236;HLA-A*0237;HLA-A*0238;HLA-A*0239;HLA-A*0240;HLA-A*0249;HLA-A*0252;HLA-A*0266;HLA-A*0289;HLA-A*0271;HLA-A*0268;HLA-A*0274;HLA-A*0258;HLA-A*0280;HLA-A*0265;HLA-A*0286;HLA-A*0277;HLA-A*0260;HLA-A*0273;HLA-A*0285;HLA-A*0264;HLA-A*0275;HLA-A*0267;HLA-A*0295;HLA-A*0297;HLA-A*9201;HLA-A*0296;HLA-A*0293;HLA-A*9221;HLA-A*9231;HLA-A*9232;HLA-A*9214;HLA-A*9202;HLA-A*9210;HLA-A*9205;HLA-A*9217;HLA-A*9211;HLA-A*9204;HLA-A*9230;HLA-A*9234;HLA-A*9218;HLA-A*9247;HLA-A*9249;HLA-A*9251;HLA-A*9248;HLA-A*9256;HLA-A*9235;HLA-A*9252;HLA-A*9240;HLA-A*9253;HLA-A*9245;HLA-A*9277;HLA-A*9257;HLA-A*9271;HLA-A*9294;HLA-A*9264;HLA-A*9290;HLA-A*9266;HLA-A*9291;HLA-A*9289;HLA-A*9274;HLA-A*9262;HLA-A*9259;HLA-A*9263;HLA-A*9268;HLA-A*9288;HLA-A*9293;HLA-A*9287;HLA-A*9283;HLA-A*9267;HLA-A*9265;HLA-A*9282;HLA-A*9297;HLA-A*9298;HLA-A*9299;HLA-A*0201;HLA-A*0209;HLA-A*0266;HLA-A*0289;HLA-A*0275;HLA-A*0297;HLA-A*0296;HLA-A*9221;HLA-A*9232;HLA-A*9234;HLA-A*9240;HLA-A*9299 |
| 3DMM | RGPGRAFVTI | 2;10 | H2-D1*02;H2-D1*02 |
| 3DX6 | EENLLDFVRF | 2;10 | HLA-B*4402;HLA-B*4402 |
| 3DX7 | EENLLDFVRF | 2;10 | HLA-B*4403;HLA-B*4403 |
| 3DX8 | EENLLDFVRF | 2;10 | HLA-B*4405;HLA-B*4405 |
| 3DXA | EENLLDFVRF | 2;10 | HLA-B*4402;HLA-B*4403;HLA-B*4404;HLA-B*4405;HLA-B*4410;HLA-B*4414;HLA-B*4416;HLA-B*4417;HLA-B*4420;HLA-B*4421;HLA-B*4427;HLA-B*4428;HLA-B*4429;HLA-B*4430;HLA-B*4431;HLA-B*4704;HLA-B*4438;HLA-B*4433;HLA-B*4445;HLA-B*4436;HLA-B*4441;HLA-B*4443;HLA-B*4435;HLA-B*4442;HLA-B*4437;HLA-B*4447;HLA-B*4448;HLA-B*4454;HLA-B*4453;HLA-B*4451;HLA-B*4465;HLA-B*4462;HLA-B*4464;HLA-B*4459;HLA-B*4466;HLA-B*4477;HLA-B*4488;HLA-B*4473;HLA-B*4482;HLA-B*4470;HLA-B*4478;HLA-B*4474;HLA-B*4487;HLA-B*4471;HLA-B*4479;HLA-B*4468;HLA-B*4476;HLA-B*4496;HLA-B*4491;HLA-B*4499;HLA-B*4492;HLA-B*4498;HLA-B*4494;HLA-B*4489;HLA-B*4405 |
| 3E6F | IGPGRAFYA | 2;9 | H2-D1*02;H2-D1*02 |
| 3E6H | IGPGRAFYTI | 2;10 | H2-D1*02;H2-D1*02 |
| 3ECB | RGPGRAFVTI | 2;10 | H2-D1*02;H2-D1*02 |
| 3FFC | FLRGRAYGL | 2;9 | HLA-B*0801;HLA-B*0807;HLA-B*0809;HLA-B*0811;HLA-B*0812;HLA-B*0813;HLA-B*0814;HLA-B*0827;HLA-B*0822;HLA-B*0820;HLA-B*0825;HLA-B*0821;HLA-B*0818;HLA-B*0828;HLA-B*0831;HLA-B*0833;HLA-B*0835;HLA-B*0837;HLA-B*0840;HLA-B*0839;HLA-B*0842;HLA-B*0843;HLA-B*0853;HLA-B*0858;HLA-B*0848;HLA-B*0849;HLA-B*0860;HLA-B*0855;HLA-B*9580;HLA-B*0801;HLA-B*0818;HLA-B*0839 |
| 3FOL | VNDIFERI | 2;8 | MH1-K1w28;MH1-K1w28 |
| 3FOM | IQQSIERI | 2;8 | MH1-K1w28;MH1-K1w28 |
| 3FON | VNDIFEAI | 2;8 | MH1-K1w28;MH1-K1w28 |
| 3FQR | YLDSGIHSGA | 2;10 | HLA-A*0201;HLA-A*0201 |
| 3FQT | GLLGSPVRA | 2;9 | HLA-A*0201;HLA-A*0203;HLA-A*0204;HLA-A*0207;HLA-A*0209;HLA-A*0212;HLA-A*0213;HLA-A*0216;HLA-A*0217;HLA-A*0218;HLA-A*0219;HLA-A*0222;HLA-A*0224;HLA-A*0225;HLA-A*0226;HLA-A*0227;HLA-A*0233;HLA-A*0236;HLA-A*0237;HLA-A*0238;HLA-A*0239;HLA-A*0240;HLA-A*0249;HLA-A*0252;HLA-A*0266;HLA-A*0289;HLA-A*0271;HLA-A*0268;HLA-A*0274;HLA-A*0258;HLA-A*0280;HLA-A*0265;HLA-A*0286;HLA-A*0277;HLA-A*0260;HLA-A*0273;HLA-A*0285;HLA-A*0264;HLA-A*0275;HLA-A*0267;HLA-A*0295;HLA-A*0297;HLA-A*9201;HLA-A*0296;HLA-A*0293;HLA-A*9221;HLA-A*9231;HLA-A*9232;HLA-A*9214;HLA-A*9202;HLA-A*9210;HLA-A*9205;HLA-A*9217;HLA-A*9211;HLA-A*9204;HLA-A*9230;HLA-A*9234;HLA-A*9218;HLA-A*9247;HLA-A*9249;HLA-A*9251;HLA-A*9248;HLA-A*9256;HLA-A*9235;HLA-A*9252;HLA-A*9240;HLA-A*9253;HLA-A*9245;HLA-A*9277;HLA-A*9257;HLA-A*9271;HLA-A*9294;HLA-A*9264;HLA-A*9290;HLA-A*9266;HLA-A*9291;HLA-A*9289;HLA-A*9274;HLA-A*9262;HLA-A*9259;HLA-A*9263;HLA-A*9268;HLA-A*9288;HLA-A*9293;HLA-A*9287;HLA-A*9283;HLA-A*9267;HLA-A*9265;HLA-A*9282;HLA-A*9297;HLA-A*9298;HLA-A*9299;HLA-A*0201;HLA-A*0209;HLA-A*0266;HLA-A*0289;HLA-A*0275;HLA-A*0297;HLA-A*0296;HLA-A*9221;HLA-A*9232;HLA-A*9234;HLA-A*9240;HLA-A*9299 |
| 3FQU | GLLGSPVRA | 2;9 | HLA-A*0201;HLA-A*0203;HLA-A*0204;HLA-A*0207;HLA-A*0209;HLA-A*0212;HLA-A*0213;HLA-A*0216;HLA-A*0217;HLA-A*0218;HLA-A*0219;HLA-A*0222;HLA-A*0224;HLA-A*0225;HLA-A*0226;HLA-A*0227;HLA-A*0233;HLA-A*0236;HLA-A*0237;HLA-A*0238;HLA-A*0239;HLA-A*0240;HLA-A*0249;HLA-A*0252;HLA-A*0266;HLA-A*0289;HLA-A*0271;HLA-A*0268;HLA-A*0274;HLA-A*0258;HLA-A*0280;HLA-A*0265;HLA-A*0286;HLA-A*0277;HLA-A*0260;HLA-A*0273;HLA-A*0285;HLA-A*0264;HLA-A*0275;HLA-A*0267;HLA-A*0295;HLA-A*0297;HLA-A*9201;HLA-A*0296;HLA-A*0293;HLA-A*9221;HLA-A*9231;HLA-A*9232;HLA-A*9214;HLA-A*9202;HLA-A*9210;HLA-A*9205;HLA-A*9217;HLA-A*9211;HLA-A*9204;HLA-A*9230;HLA-A*9234;HLA-A*9218;HLA-A*9247;HLA-A*9249;HLA-A*9251;HLA-A*9248;HLA-A*9256;HLA-A*9235;HLA-A*9252;HLA-A*9240;HLA-A*9253;HLA-A*9245;HLA-A*9277;HLA-A*9257;HLA-A*9271;HLA-A*9294;HLA-A*9264;HLA-A*9290;HLA-A*9266;HLA-A*9291;HLA-A*9289;HLA-A*9274;HLA-A*9262;HLA-A*9259;HLA-A*9263;HLA-A*9268;HLA-A*9288;HLA-A*9293;HLA-A*9287;HLA-A*9283;HLA-A*9267;HLA-A*9265;HLA-A*9282;HLA-A*9297;HLA-A*9298;HLA-A*9299;HLA-A*0201;HLA-A*0209;HLA-A*0266;HLA-A*0289;HLA-A*0275;HLA-A*0297;HLA-A*0296;HLA-A*9221;HLA-A*9232;HLA-A*9234;HLA-A*9240;HLA-A*9299 |
| 3FQW | RVASPTSGV | 2;9 | HLA-A*0201;HLA-A*0296;HLA-A*9221;HLA-A*0201;HLA-A*0296;HLA-A*9221 |
| 3FQX | RVASPTSGV | 2;9 | HLA-A*0201;HLA-A*0203;HLA-A*0204;HLA-A*0207;HLA-A*0209;HLA-A*0212;HLA-A*0213;HLA-A*0216;HLA-A*0217;HLA-A*0218;HLA-A*0219;HLA-A*0222;HLA-A*0224;HLA-A*0225;HLA-A*0226;HLA-A*0227;HLA-A*0233;HLA-A*0236;HLA-A*0237;HLA-A*0238;HLA-A*0239;HLA-A*0240;HLA-A*0249;HLA-A*0252;HLA-A*0266;HLA-A*0289;HLA-A*0271;HLA-A*0268;HLA-A*0274;HLA-A*0258;HLA-A*0280;HLA-A*0265;HLA-A*0286;HLA-A*0277;HLA-A*0260;HLA-A*0273;HLA-A*0285;HLA-A*0264;HLA-A*0275;HLA-A*0267;HLA-A*0295;HLA-A*0297;HLA-A*9201;HLA-A*0296;HLA-A*0293;HLA-A*9221;HLA-A*9231;HLA-A*9232;HLA-A*9214;HLA-A*9202;HLA-A*9210;HLA-A*9205;HLA-A*9217;HLA-A*9211;HLA-A*9204;HLA-A*9230;HLA-A*9234;HLA-A*9218;HLA-A*9247;HLA-A*9249;HLA-A*9251;HLA-A*9248;HLA-A*9256;HLA-A*9235;HLA-A*9252;HLA-A*9240;HLA-A*9253;HLA-A*9245;HLA-A*9277;HLA-A*9257;HLA-A*9271;HLA-A*9294;HLA-A*9264;HLA-A*9290;HLA-A*9266;HLA-A*9291;HLA-A*9289;HLA-A*9274;HLA-A*9262;HLA-A*9259;HLA-A*9263;HLA-A*9268;HLA-A*9288;HLA-A*9293;HLA-A*9287;HLA-A*9283;HLA-A*9267;HLA-A*9265;HLA-A*9282;HLA-A*9297;HLA-A*9298;HLA-A*9299;HLA-A*0201;HLA-A*0209;HLA-A*0266;HLA-A*0289;HLA-A*0275;HLA-A*0297;HLA-A*0296;HLA-A*9221;HLA-A*9232;HLA-A*9234;HLA-A*9240;HLA-A*9299 |
| 3FT3 | VLHDDLLEA | 2;9 | HLA-A*0201;HLA-A*0201 |
| 3FT4 | VLRDDLLEA | 2;9 | HLA-A*0201;HLA-A*0201 |
| 3FTG | ASAENMETM | 2;9 | H2-D1b;H2-D1b |
| 3GIV | SLFNTVATLY | 2;9 | HLA-A*0201;HLA-A*0296;HLA-A*9221;HLA-A*0201;HLA-A*0296;HLA-A*9221 |
| 3GJF | SLLMWITQV | 2;9 | HLA-A*0201;HLA-A*0201 |
| 3GJG | SLLMWITQV | 2;9 | HLA-A*0201;HLA-A*0201 |
| 3GSN | NLVPMVATV | 2;9 | HLA-A*0201;HLA-A*0201 |
| 3GSO | NLVPMVATV | 2;9 | HLA-A*0201;HLA-A*0201 |
| 3GSQ | NLVPSVATV | 2;9 | HLA-A*0201;HLA-A*0201 |
| 3GSR | NLVPVVATV | 2;9 | HLA-A*0201;HLA-A*0201 |
| 3GSU | NLVPTVATV | 2;9 | HLA-A*0201;HLA-A*0201 |
| 3GSV | NLVPQVATV | 2;9 | HLA-A*0201;HLA-A*0201 |
| 3GSW | NLVPMVAAV | 2;9 | HLA-A*0201;HLA-A*0201 |
| 3H7B | MLWGYLQYV | 2;9 | HLA-A*0201;HLA-A*0296;HLA-A*9221;HLA-A*0201;HLA-A*0296;HLA-A*9221 |
| 3H9H | MLWGYLQYV | 2;9 | HLA-A*0201;HLA-A*0296;HLA-A*9221;HLA-A*0201;HLA-A*0296;HLA-A*9221 |
| 3H9S | MLWGYLQYV | 2;9 | HLA-A*0201;HLA-A*0203;HLA-A*0204;HLA-A*0207;HLA-A*0209;HLA-A*0212;HLA-A*0213;HLA-A*0216;HLA-A*0217;HLA-A*0218;HLA-A*0219;HLA-A*0222;HLA-A*0224;HLA-A*0225;HLA-A*0226;HLA-A*0227;HLA-A*0233;HLA-A*0236;HLA-A*0237;HLA-A*0238;HLA-A*0239;HLA-A*0240;HLA-A*0249;HLA-A*0252;HLA-A*0266;HLA-A*0289;HLA-A*0271;HLA-A*0268;HLA-A*0274;HLA-A*0258;HLA-A*0280;HLA-A*0265;HLA-A*0286;HLA-A*0277;HLA-A*0260;HLA-A*0273;HLA-A*0285;HLA-A*0264;HLA-A*0275;HLA-A*0267;HLA-A*0295;HLA-A*0297;HLA-A*9201;HLA-A*0296;HLA-A*0293;HLA-A*9221;HLA-A*9231;HLA-A*9232;HLA-A*9214;HLA-A*9202;HLA-A*9210;HLA-A*9205;HLA-A*9217;HLA-A*9211;HLA-A*9204;HLA-A*9230;HLA-A*9234;HLA-A*9218;HLA-A*9247;HLA-A*9249;HLA-A*9251;HLA-A*9248;HLA-A*9256;HLA-A*9235;HLA-A*9252;HLA-A*9240;HLA-A*9253;HLA-A*9245;HLA-A*9277;HLA-A*9257;HLA-A*9271;HLA-A*9294;HLA-A*9264;HLA-A*9290;HLA-A*9266;HLA-A*9291;HLA-A*9289;HLA-A*9274;HLA-A*9262;HLA-A*9259;HLA-A*9263;HLA-A*9268;HLA-A*9288;HLA-A*9293;HLA-A*9287;HLA-A*9283;HLA-A*9267;HLA-A*9265;HLA-A*9282;HLA-A*9297;HLA-A*9298;HLA-A*9299;HLA-A*0201;HLA-A*0209;HLA-A*0266;HLA-A*0289;HLA-A*0275;HLA-A*0297;HLA-A*0296;HLA-A*9221;HLA-A*9232;HLA-A*9234;HLA-A*9240;HLA-A*9299 |
| 3HAE | SLLMWITQV | 2;9 | HLA-A*0201;HLA-A*0203;HLA-A*0204;HLA-A*0207;HLA-A*0209;HLA-A*0212;HLA-A*0213;HLA-A*0216;HLA-A*0217;HLA-A*0218;HLA-A*0219;HLA-A*0222;HLA-A*0224;HLA-A*0225;HLA-A*0226;HLA-A*0227;HLA-A*0233;HLA-A*0236;HLA-A*0237;HLA-A*0238;HLA-A*0239;HLA-A*0240;HLA-A*0249;HLA-A*0252;HLA-A*0266;HLA-A*0289;HLA-A*0271;HLA-A*0268;HLA-A*0274;HLA-A*0258;HLA-A*0280;HLA-A*0265;HLA-A*0286;HLA-A*0277;HLA-A*0260;HLA-A*0273;HLA-A*0285;HLA-A*0264;HLA-A*0275;HLA-A*0267;HLA-A*0295;HLA-A*0297;HLA-A*9201;HLA-A*0296;HLA-A*0293;HLA-A*9221;HLA-A*9231;HLA-A*9232;HLA-A*9214;HLA-A*9202;HLA-A*9210;HLA-A*9205;HLA-A*9217;HLA-A*9211;HLA-A*9204;HLA-A*9230;HLA-A*9234;HLA-A*9218;HLA-A*9247;HLA-A*9249;HLA-A*9251;HLA-A*9248;HLA-A*9256;HLA-A*9235;HLA-A*9252;HLA-A*9240;HLA-A*9253;HLA-A*9245;HLA-A*9277;HLA-A*9257;HLA-A*9271;HLA-A*9294;HLA-A*9264;HLA-A*9290;HLA-A*9266;HLA-A*9291;HLA-A*9289;HLA-A*9274;HLA-A*9262;HLA-A*9259;HLA-A*9263;HLA-A*9268;HLA-A*9288;HLA-A*9293;HLA-A*9287;HLA-A*9283;HLA-A*9267;HLA-A*9265;HLA-A*9282;HLA-A*9297;HLA-A*9298;HLA-A*9299;HLA-A*0201;HLA-A*0209;HLA-A*0266;HLA-A*0289;HLA-A*0275;HLA-A*0297;HLA-A*0296;HLA-A*9221;HLA-A*9232;HLA-A*9234;HLA-A*9240;HLA-A*9299 |
| 3HG1 | ELAGIGILTV | 2;10 | HLA-A*0201;HLA-A*0296;HLA-A*9221;HLA-A*0201;HLA-A*0296;HLA-A*9221 |
| 3HPJ | RMFPNAPYL | 2;9 | HLA-A*0201;HLA-A*0296;HLA-A*9221;HLA-A*0201;HLA-A*0296;HLA-A*9221 |
| 3I6G | GLMWLSYFV | 2;9 | HLA-A*0201;HLA-A*0296;HLA-A*9221;HLA-A*0201;HLA-A*0296;HLA-A*9221 |
| 3I6K | TLACFVLAAV | 2;10 | HLA-A*0201;HLA-A*0296;HLA-A*9221;HLA-A*0201;HLA-A*0296;HLA-A*9221 |
| 3I6L | QFKDNVILL | 2;9 | HLA-A*2402;HLA-A*2402 |
| 3IXA | LLFGYPVYV | 2;9 | HLA-A*0201;HLA-A*0296;HLA-A*9221;HLA-A*0201;HLA-A*0296;HLA-A*9221 |
| 3JTS | GSENLKSLY | 2;9 | MH1-A*02;MH1-A*02 |
| 3JTT | YTSGPGIRY | 2;9 | MH1-A*02;MH1-A*02 |
| 3KLA | SLLMWITQL | 2;9 | HLA-A*0201;HLA-A*0296;HLA-A*9221;HLA-A*0201;HLA-A*0296;HLA-A*9221 |
| 3KPL | EEYLQAFTY | 2;9 | HLA-B*4402;HLA-B*4402 |
| 3KPM | EEYLKAWTF | 2;9 | HLA-B*4402;HLA-B*4402 |
| 3KPN | EEYLQAFTY | 2;9 | HLA-B*4403;HLA-B*4403 |
| 3KPO | EEYLKAWTF | 2;9 | HLA-B*4403;HLA-B*4403 |
| 3KPP | EEYLQAFTY | 2;9 | HLA-B*4405;HLA-B*4405 |
| 3KPQ | EEYLKAWTF | 2;9 | HLA-B*4405;HLA-B*4405 |
| 3KPS | EEYLQAFTY | 2;9 | HLA-B*4402;HLA-B*4403;HLA-B*4404;HLA-B*4405;HLA-B*4410;HLA-B*4414;HLA-B*4416;HLA-B*4417;HLA-B*4420;HLA-B*4421;HLA-B*4427;HLA-B*4428;HLA-B*4429;HLA-B*4430;HLA-B*4431;HLA-B*4704;HLA-B*4438;HLA-B*4433;HLA-B*4445;HLA-B*4436;HLA-B*4441;HLA-B*4443;HLA-B*4435;HLA-B*4442;HLA-B*4437;HLA-B*4447;HLA-B*4448;HLA-B*4454;HLA-B*4453;HLA-B*4451;HLA-B*4465;HLA-B*4462;HLA-B*4464;HLA-B*4459;HLA-B*4466;HLA-B*4477;HLA-B*4488;HLA-B*4473;HLA-B*4482;HLA-B*4470;HLA-B*4478;HLA-B*4474;HLA-B*4487;HLA-B*4471;HLA-B*4479;HLA-B*4468;HLA-B*4476;HLA-B*4496;HLA-B*4491;HLA-B*4499;HLA-B*4492;HLA-B*4498;HLA-B*4494;HLA-B*4489;HLA-B*4405 |
| 3KXF | LPEPLPQGQLTAY | 2;13 | HLA-B*3578 |
| 3KYN | KGPPAALTL | 2;9 | HLA-G*0104;HLA-G*0104 |
| 3KYO | KLPAQFYIL | 2;9 | HLA-G*0104;HLA-G*0104 |
| 3L3D | EEAGRAFSF | 2;9 | HLA-B*4402;HLA-B*4402 |
| 3L3G | EEFGAAFSF | 2;9 | HLA-B*4402;HLA-B*4403;HLA-B*4404;HLA-B*4405;HLA-B*4410;HLA-B*4414;HLA-B*4416;HLA-B*4417;HLA-B*4420;HLA-B*4421;HLA-B*4427;HLA-B*4428;HLA-B*4429;HLA-B*4430;HLA-B*4431;HLA-B*4704;HLA-B*4438;HLA-B*4433;HLA-B*4445;HLA-B*4436;HLA-B*4441;HLA-B*4443;HLA-B*4435;HLA-B*4442;HLA-B*4437;HLA-B*4447;HLA-B*4448;HLA-B*4454;HLA-B*4453;HLA-B*4451;HLA-B*4465;HLA-B*4462;HLA-B*4464;HLA-B*4459;HLA-B*4466;HLA-B*4477;HLA-B*4488;HLA-B*4473;HLA-B*4482;HLA-B*4470;HLA-B*4478;HLA-B*4474;HLA-B*4487;HLA-B*4471;HLA-B*4479;HLA-B*4468;HLA-B*4476;HLA-B*4496;HLA-B*4491;HLA-B*4499;HLA-B*4492;HLA-B*4498;HLA-B*4494;HLA-B*4489;HLA-B*4402;HLA-B*4427;HLA-B*4466 |
| 3L3H | SSLENARAYV | 2;10 | H2-D1b;H2-D1b |
| 3L3J | EEAGAAFSF | 2;9 | HLA-B*4402;HLA-B*4402 |
| 3L3K | EEFGAAASF | 2;9 | HLA-B*4402;HLA-B*4402 |
| 3LKN | LPFERATIM | 2;9 | HLA-B*3501;HLA-B*3542;HLA-B*3501;HLA-B*3542 |
| 3LKO | LPFDRTTIM | 2;9 | HLA-B*3501;HLA-B*3542;HLA-B*3501;HLA-B*3542 |
| 3LKP | LPFDKSTIM | 2;9 | HLA-B*3501;HLA-B*3542;HLA-B*3501;HLA-B*3542 |
| 3LKQ | LPFDKTTIM | 2;9 | HLA-B*3501;HLA-B*3542;HLA-B*3501;HLA-B*3542 |
| 3LKR | LPFERATVM | 2;9 | HLA-B*3501;HLA-B*3542;HLA-B*3501;HLA-B*3542 |
| 3LKS | LPFEKSTVM | 2;9 | HLA-B*3501;HLA-B*3542;HLA-B*3501;HLA-B*3542 |
| 3LN5 | HEEAVSVDRVL | 2;11 | HLA-B*4001;HLA-B*4014;HLA-B*4015;HLA-B*4016;HLA-B*4023;HLA-B*4030;HLA-B*4031;HLA-B*4032;HLA-B*4033;HLA-B*4034;HLA-B*4036;HLA-B*4038;HLA-B*4042;HLA-B*4101;HLA-B*4102;HLA-B*4103;HLA-B*4104;HLA-B*4106;HLA-B*4501;HLA-B*4502;HLA-B*4503;HLA-B*4504;HLA-B*4505;HLA-B*5001;HLA-B*5002;HLA-B*5004;HLA-B*4053;HLA-B*4507;HLA-B*4062;HLA-B*4045;HLA-B*4051;HLA-B*3563;HLA-B*4107;HLA-B*4060;HLA-B*4059;HLA-B*4054;HLA-B*4052;HLA-B*4108;HLA-B*4048;HLA-B*4055;HLA-B*4065;HLA-B*4067;HLA-B*4063;HLA-B*4077;HLA-B*4072;HLA-B*4087;HLA-B*4092;HLA-B*4508;HLA-B*4088;HLA-B*4080;HLA-B*4509;HLA-B*4510;HLA-B*5008;HLA-B*4110;HLA-B*4109;HLA-B*5009;HLA-B*4511;HLA-B*4104 |
| 3LV3 | SRRWRRWNR | 2;9 | HLA-B*2705;HLA-B*2707;HLA-B*2709;HLA-B*2710;HLA-B*2713;HLA-B*2714;HLA-B*2719;HLA-B*2728;HLA-B*2727;HLA-B*2732;HLA-B*2735;HLA-B*2734;HLA-B*2738;HLA-B*2741;HLA-B*2745;HLA-B*2743;HLA-B*2750;HLA-B*2746;HLA-B*2755;HLA-B*2756;HLA-B*2747;HLA-B*2754;HLA-B*2758;HLA-B*2760;HLA-B*2705;HLA-B*2713 |
| 3MGO | RLYQNPTTYI | 2;10 | HLA-A*0201;HLA-A*0296;HLA-A*9221;HLA-A*0201;HLA-A*0296;HLA-A*9221 |
| 3MGT | KLYQNPTTYI | 2;10 | HLA-A*0201;HLA-A*0296;HLA-A*9221;HLA-A*0201;HLA-A*0296;HLA-A*9221 |
| 3MR9 | NLVPAVATV | 2;9 | HLA-A*0201;HLA-A*0201 |
| 3MRB | NLVPMVHTV | 2;9 | HLA-A*0201;HLA-A*0201 |
| 3MRC | NLVPMCATV | 2;9 | HLA-A*0201;HLA-A*0201 |
| 3MRD | NLVPMGATV | 2;9 | HLA-A*0201;HLA-A*0201 |
| 3MRE | GLCTLVAML | 2;9 | HLA-A*0201;HLA-A*0201 |
| 3MRF | GLCPLVAML | 2;9 | HLA-A*0201;HLA-A*0201 |
| 3MRG | CINGVCWTV | 2;9 | HLA-A*0201;HLA-A*0201 |
| 3MRH | CISGVCWTV | 2;9 | HLA-A*0201;HLA-A*0201 |
| 3MRI | CINMWCWTV | 2;9 | HLA-A*0201;HLA-A*0201 |
| 3MRJ | CINGMCWTV | 2;9 | HLA-A*0201;HLA-A*0201 |
| 3MRK | PLFQVPEPV | 2;9 | HLA-A*0201;HLA-A*0201 |
| 3MRL | CINGVVWTV | 2;9 | HLA-A*0201;HLA-A*0201 |
| 3MRM | KLVALGINAV | 2;10 | HLA-A*0201;HLA-A*0201 |
| 3MRN | LLFNILGGWV | 2;10 | HLA-A*0201;HLA-A*0201 |
| 3MRO | ELAGWGILTV | 2;10 | HLA-A*0201;HLA-A*0201 |
| 3MRP | ELAGLGINTV | 2;10 | HLA-A*0201;HLA-A*0201 |
| 3MRQ | ELAGLGINTV | 2;10 | HLA-A*0201;HLA-A*0201 |
| 3MRR | LLAGIGTVPI | 2;10 | HLA-A*0201;HLA-A*0201 |
| 3MV7 | HPVGEADYFEY | 2;11 | HLA-B*3501;HLA-B*3502;HLA-B*3503;HLA-B*3504;HLA-B*3505;HLA-B*3506;HLA-B*3508;HLA-B*3509;HLA-B*3512;HLA-B*3514;HLA-B*3515;HLA-B*3517;HLA-B*3518;HLA-B*3521;HLA-B*3522;HLA-B*3523;HLA-B*3524;HLA-B*3530;HLA-B*3531;HLA-B*3532;HLA-B*3533;HLA-B*3534;HLA-B*3535;HLA-B*3537;HLA-B*3538;HLA-B*3539;HLA-B*3541;HLA-B*7802;HLA-B*7804;HLA-B*3542;HLA-B*3565;HLA-B*3555;HLA-B*3560;HLA-B*3548;HLA-B*3559;HLA-B*3544;HLA-B*3558;HLA-B*3561;HLA-B*3551;HLA-B*3557;HLA-B*3545;HLA-B*3564;HLA-B*3567;HLA-B*3566;HLA-B*3562;HLA-B*3579;HLA-B*3575;HLA-B*3571;HLA-B*3570;HLA-B*3568;HLA-B*3583;HLA-B*3593;HLA-B*3587;HLA-B*3586;HLA-B*3581;HLA-B*3589;HLA-B*3591;HLA-B*3594;HLA-B*3588;HLA-B*3599;HLA-B*3596;HLA-B*3598;HLA-B*3501;HLA-B*3542;HLA-B*3557;HLA-B*3594 |
| 3MV8 | HPVGEADYFEY | 2;11 | HLA-B*3501;HLA-B*3502;HLA-B*3503;HLA-B*3504;HLA-B*3505;HLA-B*3506;HLA-B*3508;HLA-B*3509;HLA-B*3512;HLA-B*3514;HLA-B*3515;HLA-B*3517;HLA-B*3518;HLA-B*3521;HLA-B*3522;HLA-B*3523;HLA-B*3524;HLA-B*3530;HLA-B*3531;HLA-B*3532;HLA-B*3533;HLA-B*3534;HLA-B*3535;HLA-B*3537;HLA-B*3538;HLA-B*3539;HLA-B*3541;HLA-B*7802;HLA-B*7804;HLA-B*3542;HLA-B*3565;HLA-B*3555;HLA-B*3560;HLA-B*3548;HLA-B*3559;HLA-B*3544;HLA-B*3558;HLA-B*3561;HLA-B*3551;HLA-B*3557;HLA-B*3545;HLA-B*3564;HLA-B*3567;HLA-B*3566;HLA-B*3562;HLA-B*3579;HLA-B*3575;HLA-B*3571;HLA-B*3570;HLA-B*3568;HLA-B*3583;HLA-B*3593;HLA-B*3587;HLA-B*3586;HLA-B*3581;HLA-B*3589;HLA-B*3591;HLA-B*3594;HLA-B*3588;HLA-B*3599;HLA-B*3596;HLA-B*3598;HLA-B*3501;HLA-B*3542;HLA-B*3557;HLA-B*3594 |
| 3MV9 | HPVGEADYFEY | 2;11 | HLA-B*3501;HLA-B*3502;HLA-B*3503;HLA-B*3504;HLA-B*3505;HLA-B*3506;HLA-B*3508;HLA-B*3509;HLA-B*3512;HLA-B*3514;HLA-B*3515;HLA-B*3517;HLA-B*3518;HLA-B*3521;HLA-B*3522;HLA-B*3523;HLA-B*3524;HLA-B*3530;HLA-B*3531;HLA-B*3532;HLA-B*3533;HLA-B*3534;HLA-B*3535;HLA-B*3537;HLA-B*3538;HLA-B*3539;HLA-B*3541;HLA-B*7802;HLA-B*7804;HLA-B*3542;HLA-B*3565;HLA-B*3555;HLA-B*3560;HLA-B*3548;HLA-B*3559;HLA-B*3544;HLA-B*3558;HLA-B*3561;HLA-B*3551;HLA-B*3557;HLA-B*3545;HLA-B*3564;HLA-B*3567;HLA-B*3566;HLA-B*3562;HLA-B*3579;HLA-B*3575;HLA-B*3571;HLA-B*3570;HLA-B*3568;HLA-B*3583;HLA-B*3593;HLA-B*3587;HLA-B*3586;HLA-B*3581;HLA-B*3589;HLA-B*3591;HLA-B*3594;HLA-B*3588;HLA-B*3599;HLA-B*3596;HLA-B*3598;HLA-B*3501;HLA-B*3542;HLA-B*3557;HLA-B*3594 |
| 3MYJ | YMFPNAPYL | 2;9 | HLA-A*0201;HLA-A*0201 |
| 3NFJ | RYPLTFGWCF | 2;10 | HLA-A*2402;HLA-A*2402 |
| 3NFN | RYPLTFGWCF | 2;10 | HLA-A*2402;HLA-A*2402 |
| 3O4L | GLCTLVAML | 2;9 | HLA-A*0201;HLA-A*0203;HLA-A*0204;HLA-A*0207;HLA-A*0209;HLA-A*0212;HLA-A*0213;HLA-A*0216;HLA-A*0217;HLA-A*0218;HLA-A*0219;HLA-A*0222;HLA-A*0224;HLA-A*0225;HLA-A*0226;HLA-A*0227;HLA-A*0233;HLA-A*0236;HLA-A*0237;HLA-A*0238;HLA-A*0239;HLA-A*0240;HLA-A*0249;HLA-A*0252;HLA-A*0266;HLA-A*0289;HLA-A*0271;HLA-A*0268;HLA-A*0274;HLA-A*0258;HLA-A*0280;HLA-A*0265;HLA-A*0286;HLA-A*0277;HLA-A*0260;HLA-A*0273;HLA-A*0285;HLA-A*0264;HLA-A*0275;HLA-A*0267;HLA-A*0295;HLA-A*0297;HLA-A*9201;HLA-A*0296;HLA-A*0293;HLA-A*9221;HLA-A*9231;HLA-A*9232;HLA-A*9214;HLA-A*9202;HLA-A*9210;HLA-A*9205;HLA-A*9217;HLA-A*9211;HLA-A*9204;HLA-A*9230;HLA-A*9234;HLA-A*9218;HLA-A*9247;HLA-A*9249;HLA-A*9251;HLA-A*9248;HLA-A*9256;HLA-A*9235;HLA-A*9252;HLA-A*9240;HLA-A*9253;HLA-A*9245;HLA-A*9277;HLA-A*9257;HLA-A*9271;HLA-A*9294;HLA-A*9264;HLA-A*9290;HLA-A*9266;HLA-A*9291;HLA-A*9289;HLA-A*9274;HLA-A*9262;HLA-A*9259;HLA-A*9263;HLA-A*9268;HLA-A*9288;HLA-A*9293;HLA-A*9287;HLA-A*9283;HLA-A*9267;HLA-A*9265;HLA-A*9282;HLA-A*9297;HLA-A*9298;HLA-A*9299;HLA-A*0201;HLA-A*0209;HLA-A*0266;HLA-A*0289;HLA-A*0275;HLA-A*0297;HLA-A*0296;HLA-A*9221;HLA-A*9232;HLA-A*9234;HLA-A*9240;HLA-A*9299 |
| 3OX8 | FLPSDFFPSV | 2;10 | HLA-A*0203;HLA-A*0203 |
| 3OXR | FLPSDFFPSV | 2;10 | HLA-A*0206;HLA-A*0206 |
| 3OXS | FLPSDFFPSV | 2;10 | HLA-A*0207;HLA-A*0207 |
| 3P4M | YTVKYPNL | 2;8 | MH1-K1b;MH1-K1b |
| 3P4N | YTVKFPNM | 2;8 | MH1-K1b;MH1-K1b |
| 3P4O | YTAKYPNL | 2;8 | MH1-K1b;MH1-K1b |
| 3P9L | SIINFEKL | 2;8 | MH1-K1b;MH1-K1b |
| 3P9M | SIIGFEKL | 2;8 | MH1-K1b;MH1-K1b |
| 3PAB | EIINFEKL | 2;8 | MH1-K1b;MH1-K1b |
| 3PQY | SSLENFRAYV | 2;10 | H2-D1b;H2-D1b |
| 3PWJ | LLYGFVNYV | 2;9 | HLA-A*0201;HLA-A*0201 |
| 3PWL | LGYGFVNYI | 2;9 | HLA-A*0201;HLA-A*0201 |
| 3PWN | LLYGFVNYI | 2;9 | HLA-A*0201;HLA-A*0201 |
| 3PWP | LGYGFVNYI | 2;9 | HLA-A*0201;HLA-A*0203;HLA-A*0204;HLA-A*0207;HLA-A*0209;HLA-A*0212;HLA-A*0213;HLA-A*0216;HLA-A*0217;HLA-A*0218;HLA-A*0219;HLA-A*0222;HLA-A*0224;HLA-A*0225;HLA-A*0226;HLA-A*0227;HLA-A*0233;HLA-A*0236;HLA-A*0237;HLA-A*0238;HLA-A*0239;HLA-A*0240;HLA-A*0249;HLA-A*0252;HLA-A*0266;HLA-A*0289;HLA-A*0271;HLA-A*0268;HLA-A*0274;HLA-A*0258;HLA-A*0280;HLA-A*0265;HLA-A*0286;HLA-A*0277;HLA-A*0260;HLA-A*0273;HLA-A*0285;HLA-A*0264;HLA-A*0275;HLA-A*0267;HLA-A*0295;HLA-A*0297;HLA-A*9201;HLA-A*0296;HLA-A*0293;HLA-A*9221;HLA-A*9231;HLA-A*9232;HLA-A*9214;HLA-A*9202;HLA-A*9210;HLA-A*9205;HLA-A*9217;HLA-A*9211;HLA-A*9204;HLA-A*9230;HLA-A*9234;HLA-A*9218;HLA-A*9247;HLA-A*9249;HLA-A*9251;HLA-A*9248;HLA-A*9256;HLA-A*9235;HLA-A*9252;HLA-A*9240;HLA-A*9253;HLA-A*9245;HLA-A*9277;HLA-A*9257;HLA-A*9271;HLA-A*9294;HLA-A*9264;HLA-A*9290;HLA-A*9266;HLA-A*9291;HLA-A*9289;HLA-A*9274;HLA-A*9262;HLA-A*9259;HLA-A*9263;HLA-A*9268;HLA-A*9288;HLA-A*9293;HLA-A*9287;HLA-A*9283;HLA-A*9267;HLA-A*9265;HLA-A*9282;HLA-A*9297;HLA-A*9298;HLA-A*9299;HLA-A*0201;HLA-A*0209;HLA-A*0266;HLA-A*0289;HLA-A*0275;HLA-A*0297;HLA-A*0296;HLA-A*9221;HLA-A*9232;HLA-A*9234;HLA-A*9240;HLA-A*9299 |
| 3PWU | IPAYGVLTI | 2;9 | MH1-N*01802;MH1-N*01802 |
| 3PWV | IPAYGVLTI | 2;9 | MH1-N*01802;MH1-N*01802 |
| 3QDG | ELAGIGILTV | 2;10 | HLA-A*0201;HLA-A*0203;HLA-A*0204;HLA-A*0207;HLA-A*0209;HLA-A*0212;HLA-A*0213;HLA-A*0216;HLA-A*0217;HLA-A*0218;HLA-A*0219;HLA-A*0222;HLA-A*0224;HLA-A*0225;HLA-A*0226;HLA-A*0227;HLA-A*0233;HLA-A*0236;HLA-A*0237;HLA-A*0238;HLA-A*0239;HLA-A*0240;HLA-A*0249;HLA-A*0252;HLA-A*0266;HLA-A*0289;HLA-A*0271;HLA-A*0268;HLA-A*0274;HLA-A*0258;HLA-A*0280;HLA-A*0265;HLA-A*0286;HLA-A*0277;HLA-A*0260;HLA-A*0273;HLA-A*0285;HLA-A*0264;HLA-A*0275;HLA-A*0267;HLA-A*0295;HLA-A*0297;HLA-A*9201;HLA-A*0296;HLA-A*0293;HLA-A*9221;HLA-A*9231;HLA-A*9232;HLA-A*9214;HLA-A*9202;HLA-A*9210;HLA-A*9205;HLA-A*9217;HLA-A*9211;HLA-A*9204;HLA-A*9230;HLA-A*9234;HLA-A*9218;HLA-A*9247;HLA-A*9249;HLA-A*9251;HLA-A*9248;HLA-A*9256;HLA-A*9235;HLA-A*9252;HLA-A*9240;HLA-A*9253;HLA-A*9245;HLA-A*9277;HLA-A*9257;HLA-A*9271;HLA-A*9294;HLA-A*9264;HLA-A*9290;HLA-A*9266;HLA-A*9291;HLA-A*9289;HLA-A*9274;HLA-A*9262;HLA-A*9259;HLA-A*9263;HLA-A*9268;HLA-A*9288;HLA-A*9293;HLA-A*9287;HLA-A*9283;HLA-A*9267;HLA-A*9265;HLA-A*9282;HLA-A*9297;HLA-A*9298;HLA-A*9299;HLA-A*0201;HLA-A*0209;HLA-A*0266;HLA-A*0289;HLA-A*0275;HLA-A*0297;HLA-A*0296;HLA-A*9221;HLA-A*9232;HLA-A*9234;HLA-A*9240;HLA-A*9299 |
| 3QDJ | AAGIGILTV | 2;9 | HLA-A*0201;HLA-A*0203;HLA-A*0204;HLA-A*0207;HLA-A*0209;HLA-A*0212;HLA-A*0213;HLA-A*0216;HLA-A*0217;HLA-A*0218;HLA-A*0219;HLA-A*0222;HLA-A*0224;HLA-A*0225;HLA-A*0226;HLA-A*0227;HLA-A*0233;HLA-A*0236;HLA-A*0237;HLA-A*0238;HLA-A*0239;HLA-A*0240;HLA-A*0249;HLA-A*0252;HLA-A*0266;HLA-A*0289;HLA-A*0271;HLA-A*0268;HLA-A*0274;HLA-A*0258;HLA-A*0280;HLA-A*0265;HLA-A*0286;HLA-A*0277;HLA-A*0260;HLA-A*0273;HLA-A*0285;HLA-A*0264;HLA-A*0275;HLA-A*0267;HLA-A*0295;HLA-A*0297;HLA-A*9201;HLA-A*0296;HLA-A*0293;HLA-A*9221;HLA-A*9231;HLA-A*9232;HLA-A*9214;HLA-A*9202;HLA-A*9210;HLA-A*9205;HLA-A*9217;HLA-A*9211;HLA-A*9204;HLA-A*9230;HLA-A*9234;HLA-A*9218;HLA-A*9247;HLA-A*9249;HLA-A*9251;HLA-A*9248;HLA-A*9256;HLA-A*9235;HLA-A*9252;HLA-A*9240;HLA-A*9253;HLA-A*9245;HLA-A*9277;HLA-A*9257;HLA-A*9271;HLA-A*9294;HLA-A*9264;HLA-A*9290;HLA-A*9266;HLA-A*9291;HLA-A*9289;HLA-A*9274;HLA-A*9262;HLA-A*9259;HLA-A*9263;HLA-A*9268;HLA-A*9288;HLA-A*9293;HLA-A*9287;HLA-A*9283;HLA-A*9267;HLA-A*9265;HLA-A*9282;HLA-A*9297;HLA-A*9298;HLA-A*9299;HLA-A*0201;HLA-A*0209;HLA-A*0266;HLA-A*0289;HLA-A*0275;HLA-A*0297;HLA-A*0296;HLA-A*9221;HLA-A*9232;HLA-A*9234;HLA-A*9240;HLA-A*9299 |
| 3QDM | ELAGIGILTV | 2;10 | HLA-A*0201;HLA-A*0296;HLA-A*9221;HLA-A*0201;HLA-A*0296;HLA-A*9221 |
| 3QEQ | AAGIGILTV | 2;9 | HLA-A*0201;HLA-A*0296;HLA-A*9221;HLA-A*0201;HLA-A*0296;HLA-A*9221 |
| 3QFD | AAGIGILTV | 2;9 | HLA-A*0201;HLA-A*0296;HLA-A*9221;HLA-A*0201;HLA-A*0296;HLA-A*9221 |
| 3QFJ | LLFGFPVYV | 2;9 | HLA-A*0201;HLA-A*0203;HLA-A*0204;HLA-A*0207;HLA-A*0209;HLA-A*0212;HLA-A*0213;HLA-A*0216;HLA-A*0217;HLA-A*0218;HLA-A*0219;HLA-A*0222;HLA-A*0224;HLA-A*0225;HLA-A*0226;HLA-A*0227;HLA-A*0233;HLA-A*0236;HLA-A*0237;HLA-A*0238;HLA-A*0239;HLA-A*0240;HLA-A*0249;HLA-A*0252;HLA-A*0266;HLA-A*0289;HLA-A*0271;HLA-A*0268;HLA-A*0274;HLA-A*0258;HLA-A*0280;HLA-A*0265;HLA-A*0286;HLA-A*0277;HLA-A*0260;HLA-A*0273;HLA-A*0285;HLA-A*0264;HLA-A*0275;HLA-A*0267;HLA-A*0295;HLA-A*0297;HLA-A*9201;HLA-A*0296;HLA-A*0293;HLA-A*9221;HLA-A*9231;HLA-A*9232;HLA-A*9214;HLA-A*9202;HLA-A*9210;HLA-A*9205;HLA-A*9217;HLA-A*9211;HLA-A*9204;HLA-A*9230;HLA-A*9234;HLA-A*9218;HLA-A*9247;HLA-A*9249;HLA-A*9251;HLA-A*9248;HLA-A*9256;HLA-A*9235;HLA-A*9252;HLA-A*9240;HLA-A*9253;HLA-A*9245;HLA-A*9277;HLA-A*9257;HLA-A*9271;HLA-A*9294;HLA-A*9264;HLA-A*9290;HLA-A*9266;HLA-A*9291;HLA-A*9289;HLA-A*9274;HLA-A*9262;HLA-A*9259;HLA-A*9263;HLA-A*9268;HLA-A*9288;HLA-A*9293;HLA-A*9287;HLA-A*9283;HLA-A*9267;HLA-A*9265;HLA-A*9282;HLA-A*9297;HLA-A*9298;HLA-A*9299;HLA-A*0201;HLA-A*0209;HLA-A*0266;HLA-A*0289;HLA-A*0275;HLA-A*0297;HLA-A*0296;HLA-A*9221;HLA-A*9232;HLA-A*9234;HLA-A*9240;HLA-A*9299 |
| 3QQ3 | NSDTVGWSW | 2;9 | MH1*0401;MH1*0401 |
| 3QQ4 | ATAAATEAY | 2;9 | MH1*0401;MH1*0401 |
| 3QUK | KAVANFATM | 2;9 | H2-D1b;H2-D1b |
| 3QUL | KAVSNFATM | 2;9 | H2-D1b;H2-D1b |
| 3QZW | RYPLTFGWCF | 2;10 | HLA-A*2402;HLA-A*2402 |
| 3REW | CLGGLLTMV | 2;9 | HLA-A*0201;HLA-A*0201 |
| 3RL1 | AIFQSSMTK | 2;9 | HLA-A*0301;HLA-A*0301 |
| 3RL2 | QVPLRPMTYK | 2;10 | HLA-A*0301;HLA-A*0301 |
| 3ROO | AVYNFATM | 2;8 | MH1-K1b;MH1-K1b |
| 3RWC | IRYPKTFGW | 2;9 | MH1-A*02;MH1-A*02 |
| 3RWD | IRYPKTFGWLW | 2;11 | MH1-A*02;MH1-A*02 |
| 3RWE | FQWMGYELW | 2;9 | MH1-A*02;MH1-A*02 |
| 3RWF | QTSQWDDPW | 2;9 | MH1-A*02;MH1-A*02 |
| 3RWG | MHPAQTSQW | 2;9 | MH1-A*02;MH1-A*02 |
| 3RWH | MRHVLEPF | 2;8 | MH1-A*02;MH1-A*02 |
| 3RWI | GSHLEVQGYW | 3;10 | MH1-A*02;MH1-A*02 |
| 3RWJ | HLEVQGYW | 1;8 | MH1-A*02;MH1-A*02 |
| 3SJV | FLRGRAYGL | 2;9 | HLA-B*0801;HLA-B*0807;HLA-B*0809;HLA-B*0811;HLA-B*0812;HLA-B*0813;HLA-B*0814;HLA-B*0827;HLA-B*0822;HLA-B*0820;HLA-B*0825;HLA-B*0821;HLA-B*0818;HLA-B*0828;HLA-B*0831;HLA-B*0833;HLA-B*0835;HLA-B*0837;HLA-B*0840;HLA-B*0839;HLA-B*0842;HLA-B*0843;HLA-B*0853;HLA-B*0858;HLA-B*0848;HLA-B*0849;HLA-B*0860;HLA-B*0855;HLA-B*9580;HLA-B*0801;HLA-B*0818;HLA-B*0839 |
| 3SKM | FLRGRAYVL | 2;9 | HLA-B*0801;HLA-B*0801 |
| 3SKO | FLRGRAYGL | 2;9 | HLA-B*0801;HLA-B*0801 |
| 3SPV | RAKFKQLL | 2;8 | HLA-B*0801;HLA-B*0801 |
| 3TBS | KAPANFATM | 2;9 | H2-D1b;H2-D1b |
| 3TBT | KAPSNFATM | 2;9 | H2-D1b;H2-D1b |
| 3TBV | KGPANFATM | 2;9 | H2-D1b;H2-D1b |
| 3TBW | KGPSNFATM | 2;9 | H2-D1b;H2-D1b |
| 3TBY | KAPFNFATM | 2;9 | H2-D1b;H2-D1b |
| 3TID | AVYNFATM | 2;8 | MH1-K1b;MH1-K1b |
| 3TIE | AIVNYANL | 2;8 | MH1-K1b;MH1-K1b |
| 3TO2 | LACFVLAAV | 2;9 | HLA-A*0201;HLA-A*0296;HLA-A*9221;HLA-A*0201;HLA-A*0296;HLA-A*9221 |
| 3UTQ | ALWGPDPAAA | 2;10 | HLA-A*0201;HLA-A*0296;HLA-A*9221;HLA-A*0201;HLA-A*0296;HLA-A*9221 |
| 3UTS | ALWGPDPAAA | 2;10 | HLA-A*0201;HLA-A*0203;HLA-A*0204;HLA-A*0207;HLA-A*0209;HLA-A*0212;HLA-A*0213;HLA-A*0216;HLA-A*0217;HLA-A*0218;HLA-A*0219;HLA-A*0222;HLA-A*0224;HLA-A*0225;HLA-A*0226;HLA-A*0227;HLA-A*0233;HLA-A*0236;HLA-A*0237;HLA-A*0238;HLA-A*0239;HLA-A*0240;HLA-A*0249;HLA-A*0252;HLA-A*0266;HLA-A*0289;HLA-A*0271;HLA-A*0268;HLA-A*0274;HLA-A*0258;HLA-A*0280;HLA-A*0265;HLA-A*0286;HLA-A*0277;HLA-A*0260;HLA-A*0273;HLA-A*0285;HLA-A*0264;HLA-A*0275;HLA-A*0267;HLA-A*0295;HLA-A*0297;HLA-A*9201;HLA-A*0296;HLA-A*0293;HLA-A*9221;HLA-A*9231;HLA-A*9232;HLA-A*9214;HLA-A*9202;HLA-A*9210;HLA-A*9205;HLA-A*9217;HLA-A*9211;HLA-A*9204;HLA-A*9230;HLA-A*9234;HLA-A*9218;HLA-A*9247;HLA-A*9249;HLA-A*9251;HLA-A*9248;HLA-A*9256;HLA-A*9235;HLA-A*9252;HLA-A*9240;HLA-A*9253;HLA-A*9245;HLA-A*9277;HLA-A*9257;HLA-A*9271;HLA-A*9294;HLA-A*9264;HLA-A*9290;HLA-A*9266;HLA-A*9291;HLA-A*9289;HLA-A*9274;HLA-A*9262;HLA-A*9259;HLA-A*9263;HLA-A*9268;HLA-A*9288;HLA-A*9293;HLA-A*9287;HLA-A*9283;HLA-A*9267;HLA-A*9265;HLA-A*9282;HLA-A*9297;HLA-A*9298;HLA-A*9299;HLA-A*0201;HLA-A*0209;HLA-A*0266;HLA-A*0289;HLA-A*0275;HLA-A*0297;HLA-A*0296;HLA-A*9221;HLA-A*9232;HLA-A*9234;HLA-A*9240;HLA-A*9299 |
| 3UTT | ALWGPDPAAA | 2;10 | HLA-A*0201;HLA-A*0203;HLA-A*0204;HLA-A*0207;HLA-A*0209;HLA-A*0212;HLA-A*0213;HLA-A*0216;HLA-A*0217;HLA-A*0218;HLA-A*0219;HLA-A*0222;HLA-A*0224;HLA-A*0225;HLA-A*0226;HLA-A*0227;HLA-A*0233;HLA-A*0236;HLA-A*0237;HLA-A*0238;HLA-A*0239;HLA-A*0240;HLA-A*0249;HLA-A*0252;HLA-A*0266;HLA-A*0289;HLA-A*0271;HLA-A*0268;HLA-A*0274;HLA-A*0258;HLA-A*0280;HLA-A*0265;HLA-A*0286;HLA-A*0277;HLA-A*0260;HLA-A*0273;HLA-A*0285;HLA-A*0264;HLA-A*0275;HLA-A*0267;HLA-A*0295;HLA-A*0297;HLA-A*9201;HLA-A*0296;HLA-A*0293;HLA-A*9221;HLA-A*9231;HLA-A*9232;HLA-A*9214;HLA-A*9202;HLA-A*9210;HLA-A*9205;HLA-A*9217;HLA-A*9211;HLA-A*9204;HLA-A*9230;HLA-A*9234;HLA-A*9218;HLA-A*9247;HLA-A*9249;HLA-A*9251;HLA-A*9248;HLA-A*9256;HLA-A*9235;HLA-A*9252;HLA-A*9240;HLA-A*9253;HLA-A*9245;HLA-A*9277;HLA-A*9257;HLA-A*9271;HLA-A*9294;HLA-A*9264;HLA-A*9290;HLA-A*9266;HLA-A*9291;HLA-A*9289;HLA-A*9274;HLA-A*9262;HLA-A*9259;HLA-A*9263;HLA-A*9268;HLA-A*9288;HLA-A*9293;HLA-A*9287;HLA-A*9283;HLA-A*9267;HLA-A*9265;HLA-A*9282;HLA-A*9297;HLA-A*9298;HLA-A*9299;HLA-A*0201;HLA-A*0209;HLA-A*0266;HLA-A*0289;HLA-A*0275;HLA-A*0297;HLA-A*0296;HLA-A*9221;HLA-A*9232;HLA-A*9234;HLA-A*9240;HLA-A*9299 |
| 3V5D | KVAELVHFL | 2;9 | HLA-A*0201;HLA-A*0296;HLA-A*9221;HLA-A*0201;HLA-A*0296;HLA-A*9221 |
| 3V5H | KVAEIVHFL | 2;9 | HLA-A*0201;HLA-A*0296;HLA-A*9221;HLA-A*0201;HLA-A*0296;HLA-A*9221 |
| 3V5K | KVAELVWFL | 2;9 | HLA-A*0201;HLA-A*0296;HLA-A*9221;HLA-A*0201;HLA-A*0296;HLA-A*9221 |
| 3VCL | RPHERNGFTVL | 2;11 | HLA-B*0702;HLA-B*0702 |
| 3VFM | LPEPLPQGQLTAY | 2;13 | HLA-B*3508;HLA-B*3508 |
| 3VFN | LPEPLPQGQLTAY | 2;13 | HLA-B*3508;HLA-B*3508 |
| 3VFO | LPEPLPQGQLTAY | 2;13 | HLA-B*3508;HLA-B*3508 |
| 3VFP | LPEPLPQGQLTAY | 2;13 | HLA-B*3508;HLA-B*3508 |
| 3VFR | LPEALPQGQLTAY | 2;13 | HLA-B*3508;HLA-B*3508 |
| 3VFS | LPEPAPQGQLTAY | 2;13 | HLA-B*3508;HLA-B*3508 |
| 3VFT | LPEPLAQGQLTAY | 2;13 | HLA-B*3508;HLA-B*3508 |
| 3VFU | LPEPLPAGQLTAY | 2;13 | HLA-B*3508;HLA-B*3508 |
| 3VFV | LPEPLPQGALTAY | 2;13 | HLA-B*3508;HLA-B*3508 |
| 3VFW | LPEPLPQGQATAY | 2;13 | HLA-B*3508;HLA-B*3508 |
| 3VH8 | LSSPVTKSF | 2;9 | HLA-B*5701;HLA-B*5701 |
| 3VJ6 | AMAPRTLLL | 2;9 | H2-T23*02;H2-T23*02 |
| 3VXM | RFPLTFGWCF | 2;10 | HLA-A*2301;HLA-A*2302;HLA-A*2303;HLA-A*2304;HLA-A*2402;HLA-A*2403;HLA-A*2405;HLA-A*2406;HLA-A*2410;HLA-A*2413;HLA-A*2414;HLA-A*2415;HLA-A*2417;HLA-A*2418;HLA-A*2422;HLA-A*2423;HLA-A*2426;HLA-A*2427;HLA-A*2433;HLA-A*2454;HLA-A*2453;HLA-A*2443;HLA-A*2456;HLA-A*2439;HLA-A*2441;HLA-A*2452;HLA-A*2310;HLA-A*2455;HLA-A*2446;HLA-A*2457;HLA-A*2314;HLA-A*2451;HLA-A*2459;HLA-A*2464;HLA-A*2466;HLA-A*2462;HLA-A*2479;HLA-A*2470;HLA-A*2473;HLA-A*2476;HLA-A*2478;HLA-A*2472;HLA-A*2316;HLA-A*2468;HLA-A*2315;HLA-A*2475;HLA-A*2481;HLA-A*2471;HLA-A*2493;HLA-A*2494;HLA-A*2318;HLA-A*2319;HLA-A*2487;HLA-A*2317;HLA-A*2491;HLA-A*2496;HLA-A*2497;HLA-A*2492;HLA-A*2485;HLA-A*2498;HLA-A*2320;HLA-A*2499;HLA-A*2323;HLA-A*2325;HLA-A*2324;HLA-A*2402;HLA-A*2479;HLA-A*2476 |
| 3VXN | RYPLTFGWCF | 2;10 | HLA-A*2301;HLA-A*2302;HLA-A*2303;HLA-A*2304;HLA-A*2402;HLA-A*2403;HLA-A*2405;HLA-A*2406;HLA-A*2410;HLA-A*2413;HLA-A*2414;HLA-A*2415;HLA-A*2417;HLA-A*2418;HLA-A*2422;HLA-A*2423;HLA-A*2426;HLA-A*2427;HLA-A*2433;HLA-A*2454;HLA-A*2453;HLA-A*2443;HLA-A*2456;HLA-A*2439;HLA-A*2441;HLA-A*2452;HLA-A*2310;HLA-A*2455;HLA-A*2446;HLA-A*2457;HLA-A*2314;HLA-A*2451;HLA-A*2459;HLA-A*2464;HLA-A*2466;HLA-A*2462;HLA-A*2479;HLA-A*2470;HLA-A*2473;HLA-A*2476;HLA-A*2478;HLA-A*2472;HLA-A*2316;HLA-A*2468;HLA-A*2315;HLA-A*2475;HLA-A*2481;HLA-A*2471;HLA-A*2493;HLA-A*2494;HLA-A*2318;HLA-A*2319;HLA-A*2487;HLA-A*2317;HLA-A*2491;HLA-A*2496;HLA-A*2497;HLA-A*2492;HLA-A*2485;HLA-A*2498;HLA-A*2320;HLA-A*2499;HLA-A*2323;HLA-A*2325;HLA-A*2324;HLA-A*2402;HLA-A*2479;HLA-A*2476 |
| 3VXO | RFPLTFGWCF | 2;10 | HLA-A*2301;HLA-A*2302;HLA-A*2303;HLA-A*2304;HLA-A*2402;HLA-A*2403;HLA-A*2405;HLA-A*2406;HLA-A*2410;HLA-A*2413;HLA-A*2414;HLA-A*2415;HLA-A*2417;HLA-A*2418;HLA-A*2422;HLA-A*2423;HLA-A*2426;HLA-A*2427;HLA-A*2433;HLA-A*2454;HLA-A*2453;HLA-A*2443;HLA-A*2456;HLA-A*2439;HLA-A*2441;HLA-A*2452;HLA-A*2310;HLA-A*2455;HLA-A*2446;HLA-A*2457;HLA-A*2314;HLA-A*2451;HLA-A*2459;HLA-A*2464;HLA-A*2466;HLA-A*2462;HLA-A*2479;HLA-A*2470;HLA-A*2473;HLA-A*2476;HLA-A*2478;HLA-A*2472;HLA-A*2316;HLA-A*2468;HLA-A*2315;HLA-A*2475;HLA-A*2481;HLA-A*2471;HLA-A*2493;HLA-A*2494;HLA-A*2318;HLA-A*2319;HLA-A*2487;HLA-A*2317;HLA-A*2491;HLA-A*2496;HLA-A*2497;HLA-A*2492;HLA-A*2485;HLA-A*2498;HLA-A*2320;HLA-A*2499;HLA-A*2323;HLA-A*2325;HLA-A*2324;HLA-A*2402;HLA-A*2479;HLA-A*2476 |
| 3VXP | RYPLTLGWCF | 2;10 | HLA-A*2301;HLA-A*2302;HLA-A*2303;HLA-A*2304;HLA-A*2402;HLA-A*2403;HLA-A*2405;HLA-A*2406;HLA-A*2410;HLA-A*2413;HLA-A*2414;HLA-A*2415;HLA-A*2417;HLA-A*2418;HLA-A*2422;HLA-A*2423;HLA-A*2426;HLA-A*2427;HLA-A*2433;HLA-A*2454;HLA-A*2453;HLA-A*2443;HLA-A*2456;HLA-A*2439;HLA-A*2441;HLA-A*2452;HLA-A*2310;HLA-A*2455;HLA-A*2446;HLA-A*2457;HLA-A*2314;HLA-A*2451;HLA-A*2459;HLA-A*2464;HLA-A*2466;HLA-A*2462;HLA-A*2479;HLA-A*2470;HLA-A*2473;HLA-A*2476;HLA-A*2478;HLA-A*2472;HLA-A*2316;HLA-A*2468;HLA-A*2315;HLA-A*2475;HLA-A*2481;HLA-A*2471;HLA-A*2493;HLA-A*2494;HLA-A*2318;HLA-A*2319;HLA-A*2487;HLA-A*2317;HLA-A*2491;HLA-A*2496;HLA-A*2497;HLA-A*2492;HLA-A*2485;HLA-A*2498;HLA-A*2320;HLA-A*2499;HLA-A*2323;HLA-A*2325;HLA-A*2324;HLA-A*2402;HLA-A*2479;HLA-A*2476 |
| 3VXR | RYPLTFGWCF | 2;10 | HLA-A*2301;HLA-A*2302;HLA-A*2303;HLA-A*2304;HLA-A*2402;HLA-A*2403;HLA-A*2405;HLA-A*2406;HLA-A*2410;HLA-A*2413;HLA-A*2414;HLA-A*2415;HLA-A*2417;HLA-A*2418;HLA-A*2422;HLA-A*2423;HLA-A*2426;HLA-A*2427;HLA-A*2433;HLA-A*2454;HLA-A*2453;HLA-A*2443;HLA-A*2456;HLA-A*2439;HLA-A*2441;HLA-A*2452;HLA-A*2310;HLA-A*2455;HLA-A*2446;HLA-A*2457;HLA-A*2314;HLA-A*2451;HLA-A*2459;HLA-A*2464;HLA-A*2466;HLA-A*2462;HLA-A*2479;HLA-A*2470;HLA-A*2473;HLA-A*2476;HLA-A*2478;HLA-A*2472;HLA-A*2316;HLA-A*2468;HLA-A*2315;HLA-A*2475;HLA-A*2481;HLA-A*2471;HLA-A*2493;HLA-A*2494;HLA-A*2318;HLA-A*2319;HLA-A*2487;HLA-A*2317;HLA-A*2491;HLA-A*2496;HLA-A*2497;HLA-A*2492;HLA-A*2485;HLA-A*2498;HLA-A*2320;HLA-A*2499;HLA-A*2323;HLA-A*2325;HLA-A*2324;HLA-A*2402;HLA-A*2479;HLA-A*2476 |
| 3VXS | RYPLTLGWCF | 2;10 | HLA-A*2301;HLA-A*2302;HLA-A*2303;HLA-A*2304;HLA-A*2402;HLA-A*2403;HLA-A*2405;HLA-A*2406;HLA-A*2410;HLA-A*2413;HLA-A*2414;HLA-A*2415;HLA-A*2417;HLA-A*2418;HLA-A*2422;HLA-A*2423;HLA-A*2426;HLA-A*2427;HLA-A*2433;HLA-A*2454;HLA-A*2453;HLA-A*2443;HLA-A*2456;HLA-A*2439;HLA-A*2441;HLA-A*2452;HLA-A*2310;HLA-A*2455;HLA-A*2446;HLA-A*2457;HLA-A*2314;HLA-A*2451;HLA-A*2459;HLA-A*2464;HLA-A*2466;HLA-A*2462;HLA-A*2479;HLA-A*2470;HLA-A*2473;HLA-A*2476;HLA-A*2478;HLA-A*2472;HLA-A*2316;HLA-A*2468;HLA-A*2315;HLA-A*2475;HLA-A*2481;HLA-A*2471;HLA-A*2493;HLA-A*2494;HLA-A*2318;HLA-A*2319;HLA-A*2487;HLA-A*2317;HLA-A*2491;HLA-A*2496;HLA-A*2497;HLA-A*2492;HLA-A*2485;HLA-A*2498;HLA-A*2320;HLA-A*2499;HLA-A*2323;HLA-A*2325;HLA-A*2324;HLA-A*2402;HLA-A*2479;HLA-A*2476 |
| 3VXU | RFPLTFGWCF | 2;10 | HLA-A*2301;HLA-A*2302;HLA-A*2303;HLA-A*2304;HLA-A*2402;HLA-A*2403;HLA-A*2405;HLA-A*2406;HLA-A*2410;HLA-A*2413;HLA-A*2414;HLA-A*2415;HLA-A*2417;HLA-A*2418;HLA-A*2422;HLA-A*2423;HLA-A*2426;HLA-A*2427;HLA-A*2433;HLA-A*2454;HLA-A*2453;HLA-A*2443;HLA-A*2456;HLA-A*2439;HLA-A*2441;HLA-A*2452;HLA-A*2310;HLA-A*2455;HLA-A*2446;HLA-A*2457;HLA-A*2314;HLA-A*2451;HLA-A*2459;HLA-A*2464;HLA-A*2466;HLA-A*2462;HLA-A*2479;HLA-A*2470;HLA-A*2473;HLA-A*2476;HLA-A*2478;HLA-A*2472;HLA-A*2316;HLA-A*2468;HLA-A*2315;HLA-A*2475;HLA-A*2481;HLA-A*2471;HLA-A*2493;HLA-A*2494;HLA-A*2318;HLA-A*2319;HLA-A*2487;HLA-A*2317;HLA-A*2491;HLA-A*2496;HLA-A*2497;HLA-A*2492;HLA-A*2485;HLA-A*2498;HLA-A*2320;HLA-A*2499;HLA-A*2323;HLA-A*2325;HLA-A*2324;HLA-A*2402;HLA-A*2479;HLA-A*2476 |
| 3W0W | RFPLTFGWCF | 2;10 | HLA-A*2301;HLA-A*2302;HLA-A*2303;HLA-A*2304;HLA-A*2402;HLA-A*2403;HLA-A*2405;HLA-A*2406;HLA-A*2410;HLA-A*2413;HLA-A*2414;HLA-A*2415;HLA-A*2417;HLA-A*2418;HLA-A*2422;HLA-A*2423;HLA-A*2426;HLA-A*2427;HLA-A*2433;HLA-A*2454;HLA-A*2453;HLA-A*2443;HLA-A*2456;HLA-A*2439;HLA-A*2441;HLA-A*2452;HLA-A*2310;HLA-A*2455;HLA-A*2446;HLA-A*2457;HLA-A*2314;HLA-A*2451;HLA-A*2459;HLA-A*2464;HLA-A*2466;HLA-A*2462;HLA-A*2479;HLA-A*2470;HLA-A*2473;HLA-A*2476;HLA-A*2478;HLA-A*2472;HLA-A*2316;HLA-A*2468;HLA-A*2315;HLA-A*2475;HLA-A*2481;HLA-A*2471;HLA-A*2493;HLA-A*2494;HLA-A*2318;HLA-A*2319;HLA-A*2487;HLA-A*2317;HLA-A*2491;HLA-A*2496;HLA-A*2497;HLA-A*2492;HLA-A*2485;HLA-A*2498;HLA-A*2320;HLA-A*2499;HLA-A*2323;HLA-A*2325;HLA-A*2324;HLA-A*2402;HLA-A*2479;HLA-A*2476 |
| 3W39 | TAFTIPSI | 2;8 | HLA-B*5201;HLA-B*5201 |
| 3WL9 | NYTPGPGIRF | 2;10 | HLA-A*2301;HLA-A*2302;HLA-A*2303;HLA-A*2304;HLA-A*2402;HLA-A*2403;HLA-A*2405;HLA-A*2406;HLA-A*2410;HLA-A*2413;HLA-A*2414;HLA-A*2415;HLA-A*2417;HLA-A*2418;HLA-A*2422;HLA-A*2423;HLA-A*2426;HLA-A*2427;HLA-A*2433;HLA-A*2454;HLA-A*2453;HLA-A*2443;HLA-A*2456;HLA-A*2439;HLA-A*2441;HLA-A*2452;HLA-A*2310;HLA-A*2455;HLA-A*2446;HLA-A*2457;HLA-A*2314;HLA-A*2451;HLA-A*2459;HLA-A*2464;HLA-A*2466;HLA-A*2462;HLA-A*2479;HLA-A*2470;HLA-A*2473;HLA-A*2476;HLA-A*2478;HLA-A*2472;HLA-A*2316;HLA-A*2468;HLA-A*2315;HLA-A*2475;HLA-A*2481;HLA-A*2471;HLA-A*2493;HLA-A*2494;HLA-A*2318;HLA-A*2319;HLA-A*2487;HLA-A*2317;HLA-A*2491;HLA-A*2496;HLA-A*2497;HLA-A*2492;HLA-A*2485;HLA-A*2498;HLA-A*2320;HLA-A*2499;HLA-A*2323;HLA-A*2325;HLA-A*2324;HLA-A*2402;HLA-A*2479;HLA-A*2476 |
| 3WLB | NYTPGPGTRF | 2;10 | HLA-A*2301;HLA-A*2302;HLA-A*2303;HLA-A*2304;HLA-A*2402;HLA-A*2403;HLA-A*2405;HLA-A*2406;HLA-A*2410;HLA-A*2413;HLA-A*2414;HLA-A*2415;HLA-A*2417;HLA-A*2418;HLA-A*2422;HLA-A*2423;HLA-A*2426;HLA-A*2427;HLA-A*2433;HLA-A*2454;HLA-A*2453;HLA-A*2443;HLA-A*2456;HLA-A*2439;HLA-A*2441;HLA-A*2452;HLA-A*2310;HLA-A*2455;HLA-A*2446;HLA-A*2457;HLA-A*2314;HLA-A*2451;HLA-A*2459;HLA-A*2464;HLA-A*2466;HLA-A*2462;HLA-A*2479;HLA-A*2470;HLA-A*2473;HLA-A*2476;HLA-A*2478;HLA-A*2472;HLA-A*2316;HLA-A*2468;HLA-A*2315;HLA-A*2475;HLA-A*2481;HLA-A*2471;HLA-A*2493;HLA-A*2494;HLA-A*2318;HLA-A*2319;HLA-A*2487;HLA-A*2317;HLA-A*2491;HLA-A*2496;HLA-A*2497;HLA-A*2492;HLA-A*2485;HLA-A*2498;HLA-A*2320;HLA-A*2499;HLA-A*2323;HLA-A*2325;HLA-A*2324;HLA-A*2402;HLA-A*2479;HLA-A*2476 |
| 3WS3 | YQLENYCGL | 2;9 | H2-D1b;H2-D1b |
| 3WS6 | YAIENYLEL | 2;9 | H2-D1b;H2-D1b |
| 3WUW | LSSPVTKSF | 2;9 | HLA-B*5701;HLA-B*5701 |
| 3X11 | LSSPVTKSF | 2;9 | HLA-B*5712 |
| 3X12 | LSSPVTKSF | 2;9 | HLA-B*5701;HLA-B*5702;HLA-B*5703;HLA-B*5704;HLA-B*5705;HLA-B*5706;HLA-B*5707;HLA-B*5709;HLA-B*5711;HLA-B*5714;HLA-B*5713;HLA-B*5718;HLA-B*5717;HLA-B*5719;HLA-B*5725;HLA-B*5722;HLA-B*5721;HLA-B*5726;HLA-B*5724;HLA-B*5729;HLA-B*5730;HLA-B*5731;HLA-B*5727;HLA-B*5701;HLA-B*5729 |
| 3X13 | FLRGRAYGL | 2;9 | HLA-B*0801;HLA-B*0807;HLA-B*0809;HLA-B*0811;HLA-B*0812;HLA-B*0813;HLA-B*0814;HLA-B*0827;HLA-B*0822;HLA-B*0820;HLA-B*0825;HLA-B*0821;HLA-B*0818;HLA-B*0828;HLA-B*0831;HLA-B*0833;HLA-B*0835;HLA-B*0837;HLA-B*0840;HLA-B*0839;HLA-B*0842;HLA-B*0843;HLA-B*0853;HLA-B*0858;HLA-B*0848;HLA-B*0849;HLA-B*0860;HLA-B*0855;HLA-B*9580;HLA-B*0801;HLA-B*0818;HLA-B*0839 |
| 3X14 | FLRGRAYGL | 2;9 | HLA-B*0852;HLA-B*0852 |
| 4CVX | YPYLGPNTL | 2;9 | MH1-B*2401 |
| 4CVZ | YELDEKFDRL | 2;10 | MH1-B*2101;MH1-B*2101 |
| 4CW1 | SWFRKPMTR | 2;9 | MH1-B*2401 |
| 4D0B | TAGQEDYDRL | 2;10 | MH1-B*2101;MH1-B*2101 |
| 4D0C | TAGQSNYDRL | 2;10 | MH1-B*2101;MH1-B*2101 |
| 4D0D | VIFPAKSL | 2;8 | MH1-B*2401 |
| 4E0R | IDWFDGKE | 2;8 | MH1-B*2401;MH1-B*2401 |
| 4E5X | LLFGYPVYV | 2;9 | HLA-A*0201;HLA-A*0296;HLA-A*9221;HLA-A*0201;HLA-A*0296;HLA-A*9221 |
| 4EUP | ALGIGILTV | 2;9 | HLA-A*0201;HLA-A*0203;HLA-A*0204;HLA-A*0207;HLA-A*0209;HLA-A*0212;HLA-A*0213;HLA-A*0216;HLA-A*0217;HLA-A*0218;HLA-A*0219;HLA-A*0222;HLA-A*0224;HLA-A*0225;HLA-A*0226;HLA-A*0227;HLA-A*0233;HLA-A*0236;HLA-A*0237;HLA-A*0238;HLA-A*0239;HLA-A*0240;HLA-A*0249;HLA-A*0252;HLA-A*0266;HLA-A*0289;HLA-A*0271;HLA-A*0268;HLA-A*0274;HLA-A*0258;HLA-A*0280;HLA-A*0265;HLA-A*0286;HLA-A*0277;HLA-A*0260;HLA-A*0273;HLA-A*0285;HLA-A*0264;HLA-A*0275;HLA-A*0267;HLA-A*0295;HLA-A*0297;HLA-A*9201;HLA-A*0296;HLA-A*0293;HLA-A*9221;HLA-A*9231;HLA-A*9232;HLA-A*9214;HLA-A*9202;HLA-A*9210;HLA-A*9205;HLA-A*9217;HLA-A*9211;HLA-A*9204;HLA-A*9230;HLA-A*9234;HLA-A*9218;HLA-A*9247;HLA-A*9249;HLA-A*9251;HLA-A*9248;HLA-A*9256;HLA-A*9235;HLA-A*9252;HLA-A*9240;HLA-A*9253;HLA-A*9245;HLA-A*9277;HLA-A*9257;HLA-A*9271;HLA-A*9294;HLA-A*9264;HLA-A*9290;HLA-A*9266;HLA-A*9291;HLA-A*9289;HLA-A*9274;HLA-A*9262;HLA-A*9259;HLA-A*9263;HLA-A*9268;HLA-A*9288;HLA-A*9293;HLA-A*9287;HLA-A*9283;HLA-A*9267;HLA-A*9265;HLA-A*9282;HLA-A*9297;HLA-A*9298;HLA-A*9299;HLA-A*0201;HLA-A*0209;HLA-A*0266;HLA-A*0289;HLA-A*0275;HLA-A*0297;HLA-A*0296;HLA-A*9221;HLA-A*9232;HLA-A*9234;HLA-A*9240;HLA-A*9299 |
| 4EUQ | EAAGIGILTV | 2;10 | HLA-A*0201;HLA-A*0203;HLA-A*0204;HLA-A*0207;HLA-A*0209;HLA-A*0212;HLA-A*0213;HLA-A*0216;HLA-A*0217;HLA-A*0218;HLA-A*0219;HLA-A*0222;HLA-A*0224;HLA-A*0225;HLA-A*0226;HLA-A*0227;HLA-A*0233;HLA-A*0236;HLA-A*0237;HLA-A*0238;HLA-A*0239;HLA-A*0240;HLA-A*0249;HLA-A*0252;HLA-A*0266;HLA-A*0289;HLA-A*0271;HLA-A*0268;HLA-A*0274;HLA-A*0258;HLA-A*0280;HLA-A*0265;HLA-A*0286;HLA-A*0277;HLA-A*0260;HLA-A*0273;HLA-A*0285;HLA-A*0264;HLA-A*0275;HLA-A*0267;HLA-A*0295;HLA-A*0297;HLA-A*9201;HLA-A*0296;HLA-A*0293;HLA-A*9221;HLA-A*9231;HLA-A*9232;HLA-A*9214;HLA-A*9202;HLA-A*9210;HLA-A*9205;HLA-A*9217;HLA-A*9211;HLA-A*9204;HLA-A*9230;HLA-A*9234;HLA-A*9218;HLA-A*9247;HLA-A*9249;HLA-A*9251;HLA-A*9248;HLA-A*9256;HLA-A*9235;HLA-A*9252;HLA-A*9240;HLA-A*9253;HLA-A*9245;HLA-A*9277;HLA-A*9257;HLA-A*9271;HLA-A*9294;HLA-A*9264;HLA-A*9290;HLA-A*9266;HLA-A*9291;HLA-A*9289;HLA-A*9274;HLA-A*9262;HLA-A*9259;HLA-A*9263;HLA-A*9268;HLA-A*9288;HLA-A*9293;HLA-A*9287;HLA-A*9283;HLA-A*9267;HLA-A*9265;HLA-A*9282;HLA-A*9297;HLA-A*9298;HLA-A*9299;HLA-A*0201;HLA-A*0209;HLA-A*0266;HLA-A*0289;HLA-A*0275;HLA-A*0297;HLA-A*0296;HLA-A*9221;HLA-A*9232;HLA-A*9234;HLA-A*9240;HLA-A*9299 |
| 4F7M | LYASPQLEGF | 2;10 | HLA-A*2402;HLA-A*2402 |
| 4F7T | RYGFVANF | 2;8 | HLA-A*2402;HLA-A*2402 |
| 4FTV | LLFGYPVYV | 2;9 | HLA-A*0201;HLA-A*0203;HLA-A*0204;HLA-A*0207;HLA-A*0209;HLA-A*0212;HLA-A*0213;HLA-A*0216;HLA-A*0217;HLA-A*0218;HLA-A*0219;HLA-A*0222;HLA-A*0224;HLA-A*0225;HLA-A*0226;HLA-A*0227;HLA-A*0233;HLA-A*0236;HLA-A*0237;HLA-A*0238;HLA-A*0239;HLA-A*0240;HLA-A*0249;HLA-A*0252;HLA-A*0266;HLA-A*0289;HLA-A*0271;HLA-A*0268;HLA-A*0274;HLA-A*0258;HLA-A*0280;HLA-A*0265;HLA-A*0286;HLA-A*0277;HLA-A*0260;HLA-A*0273;HLA-A*0285;HLA-A*0264;HLA-A*0275;HLA-A*0267;HLA-A*0295;HLA-A*0297;HLA-A*9201;HLA-A*0296;HLA-A*0293;HLA-A*9221;HLA-A*9231;HLA-A*9232;HLA-A*9214;HLA-A*9202;HLA-A*9210;HLA-A*9205;HLA-A*9217;HLA-A*9211;HLA-A*9204;HLA-A*9230;HLA-A*9234;HLA-A*9218;HLA-A*9247;HLA-A*9249;HLA-A*9251;HLA-A*9248;HLA-A*9256;HLA-A*9235;HLA-A*9252;HLA-A*9240;HLA-A*9253;HLA-A*9245;HLA-A*9277;HLA-A*9257;HLA-A*9271;HLA-A*9294;HLA-A*9264;HLA-A*9290;HLA-A*9266;HLA-A*9291;HLA-A*9289;HLA-A*9274;HLA-A*9262;HLA-A*9259;HLA-A*9263;HLA-A*9268;HLA-A*9288;HLA-A*9293;HLA-A*9287;HLA-A*9283;HLA-A*9267;HLA-A*9265;HLA-A*9282;HLA-A*9297;HLA-A*9298;HLA-A*9299;HLA-A*0201;HLA-A*0209;HLA-A*0266;HLA-A*0289;HLA-A*0275;HLA-A*0297;HLA-A*0296;HLA-A*9221;HLA-A*9232;HLA-A*9234;HLA-A*9240;HLA-A*9299 |
| 4G42 | IDWFDGKD | 2;8 | MH1-B*2401;MH1-B*2401 |
| 4G43 | IDWFEGKE | 2;8 | MH1-B*2401;MH1-B*2401 |
| 4G8G | KRWIILGLNK | 2;10 | HLA-B*2705;HLA-B*2713;HLA-B*2705;HLA-B*2713 |
| 4G8I | KRWIIMGLNK | 2;10 | HLA-B*2705;HLA-B*2707;HLA-B*2709;HLA-B*2710;HLA-B*2713;HLA-B*2714;HLA-B*2719;HLA-B*2728;HLA-B*2727;HLA-B*2732;HLA-B*2735;HLA-B*2734;HLA-B*2738;HLA-B*2741;HLA-B*2745;HLA-B*2743;HLA-B*2750;HLA-B*2746;HLA-B*2755;HLA-B*2756;HLA-B*2747;HLA-B*2754;HLA-B*2758;HLA-B*2760;HLA-B*2705;HLA-B*2713 |
| 4G9D | KRWIILGLNK | 2;10 | HLA-B*2705;HLA-B*2713;HLA-B*2705;HLA-B*2713 |
| 4G9F | KRWIIMGLNK | 2;10 | HLA-B*2705;HLA-B*2713;HLA-B*2705;HLA-B*2713 |
| 4GKN | FATGIGIITV | 2;10 | HLA-A*0201;HLA-A*0296;HLA-A*9221;HLA-A*0201;HLA-A*0296;HLA-A*9221 |
| 4GKS | FLTGIGIITV | 2;10 | HLA-A*0201;HLA-A*0296;HLA-A*9221;HLA-A*0201;HLA-A*0296;HLA-A*9221 |
| 4HKJ | SIINFEKL | 2;8 | MH1-K1b;MH1-K1b |
| 4HS3 | AVYNFATM | 2;8 | MH1-K1b;MH1-K1b |
| 4HUU | ASNENIETM | 2;9 | H2-D1b;H2-D1b |
| 4HUV | ASNENWETM | 2;9 | H2-D1b;H2-D1b |
| 4HUW | ASNENTETM | 2;9 | H2-D1b;H2-D1b |
| 4HUX | ASNENMETM | 2;9 | H2-D1b;H2-D1b |
| 4HV8 | ASNENIETM | 2;9 | H2-D1b;H2-D1b |
| 4HWZ | AIFQSSMTK | 2;9 | HLA-A*1110;HLA-A*3402;HLA-A*3403;HLA-A*6601;HLA-A*6602;HLA-A*6604;HLA-A*6801;HLA-A*6806;HLA-A*6807;HLA-A*6808;HLA-A*6809;HLA-A*6812;HLA-A*6816;HLA-A*6817;HLA-A*6819;HLA-A*6821;HLA-A*6901;HLA-A*6826;HLA-A*3406;HLA-A*6827;HLA-A*6823;HLA-A*6825;HLA-A*6833;HLA-A*6606;HLA-A*6838;HLA-A*6841;HLA-A*6842;HLA-A*6608;HLA-A*6607;HLA-A*6609;HLA-A*6610;HLA-A*6847;HLA-A*6612;HLA-A*6801;HLA-A*6833 |
| 4HX1 | SVYDFFVWL | 2;9 | HLA-C*1510 |
| 4I48 | TLTSCNTSV | 2;9 | HLA-C*1510 |
| 4I4W | ILAKFLHRL | 2;9 | HLA-A*0201;HLA-A*0203;HLA-A*0204;HLA-A*0207;HLA-A*0209;HLA-A*0212;HLA-A*0213;HLA-A*0216;HLA-A*0217;HLA-A*0218;HLA-A*0219;HLA-A*0222;HLA-A*0224;HLA-A*0225;HLA-A*0226;HLA-A*0227;HLA-A*0233;HLA-A*0236;HLA-A*0237;HLA-A*0238;HLA-A*0239;HLA-A*0240;HLA-A*0249;HLA-A*0252;HLA-A*0266;HLA-A*0289;HLA-A*0271;HLA-A*0268;HLA-A*0274;HLA-A*0258;HLA-A*0280;HLA-A*0265;HLA-A*0286;HLA-A*0277;HLA-A*0260;HLA-A*0273;HLA-A*0285;HLA-A*0264;HLA-A*0275;HLA-A*0267;HLA-A*0295;HLA-A*0297;HLA-A*9201;HLA-A*0296;HLA-A*0293;HLA-A*9221;HLA-A*9231;HLA-A*9232;HLA-A*9214;HLA-A*9202;HLA-A*9210;HLA-A*9205;HLA-A*9217;HLA-A*9211;HLA-A*9204;HLA-A*9230;HLA-A*9234;HLA-A*9218;HLA-A*9247;HLA-A*9249;HLA-A*9251;HLA-A*9248;HLA-A*9256;HLA-A*9235;HLA-A*9252;HLA-A*9240;HLA-A*9253;HLA-A*9245;HLA-A*9277;HLA-A*9257;HLA-A*9271;HLA-A*9294;HLA-A*9264;HLA-A*9290;HLA-A*9266;HLA-A*9291;HLA-A*9289;HLA-A*9274;HLA-A*9262;HLA-A*9259;HLA-A*9263;HLA-A*9268;HLA-A*9288;HLA-A*9293;HLA-A*9287;HLA-A*9283;HLA-A*9267;HLA-A*9265;HLA-A*9282;HLA-A*9297;HLA-A*9298;HLA-A*9299;HLA-A*0201;HLA-A*0209;HLA-A*0266;HLA-A*0289;HLA-A*0275;HLA-A*0297;HLA-A*0296;HLA-A*9221;HLA-A*9232;HLA-A*9234;HLA-A*9240;HLA-A*9299 |
| 4IHO | EGPRNQDWL | 2;9 | H2-D1b;H2-D1b |
| 4JFD | ELAAIGILTV | 2;10 | HLA-A*0201;HLA-A*0203;HLA-A*0204;HLA-A*0207;HLA-A*0209;HLA-A*0212;HLA-A*0213;HLA-A*0216;HLA-A*0217;HLA-A*0218;HLA-A*0219;HLA-A*0222;HLA-A*0224;HLA-A*0225;HLA-A*0226;HLA-A*0227;HLA-A*0233;HLA-A*0236;HLA-A*0237;HLA-A*0238;HLA-A*0239;HLA-A*0240;HLA-A*0249;HLA-A*0252;HLA-A*0266;HLA-A*0289;HLA-A*0271;HLA-A*0268;HLA-A*0274;HLA-A*0258;HLA-A*0280;HLA-A*0265;HLA-A*0286;HLA-A*0277;HLA-A*0260;HLA-A*0273;HLA-A*0285;HLA-A*0264;HLA-A*0275;HLA-A*0267;HLA-A*0295;HLA-A*0297;HLA-A*9201;HLA-A*0296;HLA-A*0293;HLA-A*9221;HLA-A*9231;HLA-A*9232;HLA-A*9214;HLA-A*9202;HLA-A*9210;HLA-A*9205;HLA-A*9217;HLA-A*9211;HLA-A*9204;HLA-A*9230;HLA-A*9234;HLA-A*9218;HLA-A*9247;HLA-A*9249;HLA-A*9251;HLA-A*9248;HLA-A*9256;HLA-A*9235;HLA-A*9252;HLA-A*9240;HLA-A*9253;HLA-A*9245;HLA-A*9277;HLA-A*9257;HLA-A*9271;HLA-A*9294;HLA-A*9264;HLA-A*9290;HLA-A*9266;HLA-A*9291;HLA-A*9289;HLA-A*9274;HLA-A*9262;HLA-A*9259;HLA-A*9263;HLA-A*9268;HLA-A*9288;HLA-A*9293;HLA-A*9287;HLA-A*9283;HLA-A*9267;HLA-A*9265;HLA-A*9282;HLA-A*9297;HLA-A*9298;HLA-A*9299;HLA-A*0201;HLA-A*0209;HLA-A*0266;HLA-A*0289;HLA-A*0275;HLA-A*0297;HLA-A*0296;HLA-A*9221;HLA-A*9232;HLA-A*9234;HLA-A*9240;HLA-A*9299 |
| 4JFE | ELAGIGALTV | 2;10 | HLA-A*0201;HLA-A*0203;HLA-A*0204;HLA-A*0207;HLA-A*0209;HLA-A*0212;HLA-A*0213;HLA-A*0216;HLA-A*0217;HLA-A*0218;HLA-A*0219;HLA-A*0222;HLA-A*0224;HLA-A*0225;HLA-A*0226;HLA-A*0227;HLA-A*0233;HLA-A*0236;HLA-A*0237;HLA-A*0238;HLA-A*0239;HLA-A*0240;HLA-A*0249;HLA-A*0252;HLA-A*0266;HLA-A*0289;HLA-A*0271;HLA-A*0268;HLA-A*0274;HLA-A*0258;HLA-A*0280;HLA-A*0265;HLA-A*0286;HLA-A*0277;HLA-A*0260;HLA-A*0273;HLA-A*0285;HLA-A*0264;HLA-A*0275;HLA-A*0267;HLA-A*0295;HLA-A*0297;HLA-A*9201;HLA-A*0296;HLA-A*0293;HLA-A*9221;HLA-A*9231;HLA-A*9232;HLA-A*9214;HLA-A*9202;HLA-A*9210;HLA-A*9205;HLA-A*9217;HLA-A*9211;HLA-A*9204;HLA-A*9230;HLA-A*9234;HLA-A*9218;HLA-A*9247;HLA-A*9249;HLA-A*9251;HLA-A*9248;HLA-A*9256;HLA-A*9235;HLA-A*9252;HLA-A*9240;HLA-A*9253;HLA-A*9245;HLA-A*9277;HLA-A*9257;HLA-A*9271;HLA-A*9294;HLA-A*9264;HLA-A*9290;HLA-A*9266;HLA-A*9291;HLA-A*9289;HLA-A*9274;HLA-A*9262;HLA-A*9259;HLA-A*9263;HLA-A*9268;HLA-A*9288;HLA-A*9293;HLA-A*9287;HLA-A*9283;HLA-A*9267;HLA-A*9265;HLA-A*9282;HLA-A*9297;HLA-A*9298;HLA-A*9299;HLA-A*0201;HLA-A*0209;HLA-A*0266;HLA-A*0289;HLA-A*0275;HLA-A*0297;HLA-A*0296;HLA-A*9221;HLA-A*9232;HLA-A*9234;HLA-A*9240;HLA-A*9299 |
| 4JFF | ELAGIGILTV | 2;10 | HLA-A*0201;HLA-A*0203;HLA-A*0204;HLA-A*0207;HLA-A*0209;HLA-A*0212;HLA-A*0213;HLA-A*0216;HLA-A*0217;HLA-A*0218;HLA-A*0219;HLA-A*0222;HLA-A*0224;HLA-A*0225;HLA-A*0226;HLA-A*0227;HLA-A*0233;HLA-A*0236;HLA-A*0237;HLA-A*0238;HLA-A*0239;HLA-A*0240;HLA-A*0249;HLA-A*0252;HLA-A*0266;HLA-A*0289;HLA-A*0271;HLA-A*0268;HLA-A*0274;HLA-A*0258;HLA-A*0280;HLA-A*0265;HLA-A*0286;HLA-A*0277;HLA-A*0260;HLA-A*0273;HLA-A*0285;HLA-A*0264;HLA-A*0275;HLA-A*0267;HLA-A*0295;HLA-A*0297;HLA-A*9201;HLA-A*0296;HLA-A*0293;HLA-A*9221;HLA-A*9231;HLA-A*9232;HLA-A*9214;HLA-A*9202;HLA-A*9210;HLA-A*9205;HLA-A*9217;HLA-A*9211;HLA-A*9204;HLA-A*9230;HLA-A*9234;HLA-A*9218;HLA-A*9247;HLA-A*9249;HLA-A*9251;HLA-A*9248;HLA-A*9256;HLA-A*9235;HLA-A*9252;HLA-A*9240;HLA-A*9253;HLA-A*9245;HLA-A*9277;HLA-A*9257;HLA-A*9271;HLA-A*9294;HLA-A*9264;HLA-A*9290;HLA-A*9266;HLA-A*9291;HLA-A*9289;HLA-A*9274;HLA-A*9262;HLA-A*9259;HLA-A*9263;HLA-A*9268;HLA-A*9288;HLA-A*9293;HLA-A*9287;HLA-A*9283;HLA-A*9267;HLA-A*9265;HLA-A*9282;HLA-A*9297;HLA-A*9298;HLA-A*9299;HLA-A*0201;HLA-A*0209;HLA-A*0266;HLA-A*0289;HLA-A*0275;HLA-A*0297;HLA-A*0296;HLA-A*9221;HLA-A*9232;HLA-A*9234;HLA-A*9240;HLA-A*9299 |
| 4JFO | ALAGIGILTV | 2;10 | HLA-A*0201;HLA-A*0203;HLA-A*0204;HLA-A*0207;HLA-A*0209;HLA-A*0212;HLA-A*0213;HLA-A*0216;HLA-A*0217;HLA-A*0218;HLA-A*0219;HLA-A*0222;HLA-A*0224;HLA-A*0225;HLA-A*0226;HLA-A*0227;HLA-A*0233;HLA-A*0236;HLA-A*0237;HLA-A*0238;HLA-A*0239;HLA-A*0240;HLA-A*0249;HLA-A*0252;HLA-A*0266;HLA-A*0289;HLA-A*0271;HLA-A*0268;HLA-A*0274;HLA-A*0258;HLA-A*0280;HLA-A*0265;HLA-A*0286;HLA-A*0277;HLA-A*0260;HLA-A*0273;HLA-A*0285;HLA-A*0264;HLA-A*0275;HLA-A*0267;HLA-A*0295;HLA-A*0297;HLA-A*9201;HLA-A*0296;HLA-A*0293;HLA-A*9221;HLA-A*9231;HLA-A*9232;HLA-A*9214;HLA-A*9202;HLA-A*9210;HLA-A*9205;HLA-A*9217;HLA-A*9211;HLA-A*9204;HLA-A*9230;HLA-A*9234;HLA-A*9218;HLA-A*9247;HLA-A*9249;HLA-A*9251;HLA-A*9248;HLA-A*9256;HLA-A*9235;HLA-A*9252;HLA-A*9240;HLA-A*9253;HLA-A*9245;HLA-A*9277;HLA-A*9257;HLA-A*9271;HLA-A*9294;HLA-A*9264;HLA-A*9290;HLA-A*9266;HLA-A*9291;HLA-A*9289;HLA-A*9274;HLA-A*9262;HLA-A*9259;HLA-A*9263;HLA-A*9268;HLA-A*9288;HLA-A*9293;HLA-A*9287;HLA-A*9283;HLA-A*9267;HLA-A*9265;HLA-A*9282;HLA-A*9297;HLA-A*9298;HLA-A*9299;HLA-A*0201;HLA-A*0209;HLA-A*0266;HLA-A*0289;HLA-A*0275;HLA-A*0297;HLA-A*0296;HLA-A*9221;HLA-A*9232;HLA-A*9234;HLA-A*9240;HLA-A*9299 |
| 4JFP | ELAAIGILTV | 2;10 | HLA-A*0201;HLA-A*0203;HLA-A*0204;HLA-A*0207;HLA-A*0209;HLA-A*0212;HLA-A*0213;HLA-A*0216;HLA-A*0217;HLA-A*0218;HLA-A*0219;HLA-A*0222;HLA-A*0224;HLA-A*0225;HLA-A*0226;HLA-A*0227;HLA-A*0233;HLA-A*0236;HLA-A*0237;HLA-A*0238;HLA-A*0239;HLA-A*0240;HLA-A*0249;HLA-A*0252;HLA-A*0266;HLA-A*0289;HLA-A*0271;HLA-A*0268;HLA-A*0274;HLA-A*0258;HLA-A*0280;HLA-A*0265;HLA-A*0286;HLA-A*0277;HLA-A*0260;HLA-A*0273;HLA-A*0285;HLA-A*0264;HLA-A*0275;HLA-A*0267;HLA-A*0295;HLA-A*0297;HLA-A*9201;HLA-A*0296;HLA-A*0293;HLA-A*9221;HLA-A*9231;HLA-A*9232;HLA-A*9214;HLA-A*9202;HLA-A*9210;HLA-A*9205;HLA-A*9217;HLA-A*9211;HLA-A*9204;HLA-A*9230;HLA-A*9234;HLA-A*9218;HLA-A*9247;HLA-A*9249;HLA-A*9251;HLA-A*9248;HLA-A*9256;HLA-A*9235;HLA-A*9252;HLA-A*9240;HLA-A*9253;HLA-A*9245;HLA-A*9277;HLA-A*9257;HLA-A*9271;HLA-A*9294;HLA-A*9264;HLA-A*9290;HLA-A*9266;HLA-A*9291;HLA-A*9289;HLA-A*9274;HLA-A*9262;HLA-A*9259;HLA-A*9263;HLA-A*9268;HLA-A*9288;HLA-A*9293;HLA-A*9287;HLA-A*9283;HLA-A*9267;HLA-A*9265;HLA-A*9282;HLA-A*9297;HLA-A*9298;HLA-A*9299;HLA-A*0201;HLA-A*0209;HLA-A*0266;HLA-A*0289;HLA-A*0275;HLA-A*0297;HLA-A*0296;HLA-A*9221;HLA-A*9232;HLA-A*9234;HLA-A*9240;HLA-A*9299 |
| 4JFQ | ELAGIGIATV | 2;10 | HLA-A*0201;HLA-A*0203;HLA-A*0204;HLA-A*0207;HLA-A*0209;HLA-A*0212;HLA-A*0213;HLA-A*0216;HLA-A*0217;HLA-A*0218;HLA-A*0219;HLA-A*0222;HLA-A*0224;HLA-A*0225;HLA-A*0226;HLA-A*0227;HLA-A*0233;HLA-A*0236;HLA-A*0237;HLA-A*0238;HLA-A*0239;HLA-A*0240;HLA-A*0249;HLA-A*0252;HLA-A*0266;HLA-A*0289;HLA-A*0271;HLA-A*0268;HLA-A*0274;HLA-A*0258;HLA-A*0280;HLA-A*0265;HLA-A*0286;HLA-A*0277;HLA-A*0260;HLA-A*0273;HLA-A*0285;HLA-A*0264;HLA-A*0275;HLA-A*0267;HLA-A*0295;HLA-A*0297;HLA-A*9201;HLA-A*0296;HLA-A*0293;HLA-A*9221;HLA-A*9231;HLA-A*9232;HLA-A*9214;HLA-A*9202;HLA-A*9210;HLA-A*9205;HLA-A*9217;HLA-A*9211;HLA-A*9204;HLA-A*9230;HLA-A*9234;HLA-A*9218;HLA-A*9247;HLA-A*9249;HLA-A*9251;HLA-A*9248;HLA-A*9256;HLA-A*9235;HLA-A*9252;HLA-A*9240;HLA-A*9253;HLA-A*9245;HLA-A*9277;HLA-A*9257;HLA-A*9271;HLA-A*9294;HLA-A*9264;HLA-A*9290;HLA-A*9266;HLA-A*9291;HLA-A*9289;HLA-A*9274;HLA-A*9262;HLA-A*9259;HLA-A*9263;HLA-A*9268;HLA-A*9288;HLA-A*9293;HLA-A*9287;HLA-A*9283;HLA-A*9267;HLA-A*9265;HLA-A*9282;HLA-A*9297;HLA-A*9298;HLA-A*9299;HLA-A*0201;HLA-A*0209;HLA-A*0266;HLA-A*0289;HLA-A*0275;HLA-A*0297;HLA-A*0296;HLA-A*9221;HLA-A*9232;HLA-A*9234;HLA-A*9240;HLA-A*9299 |
| 4JQV | SELEIKRY | 2;8 | HLA-B*1801;HLA-B*1802;HLA-B*1805;HLA-B*1808;HLA-B*1810;HLA-B*1811;HLA-B*1813;HLA-B*1814;HLA-B*1815;HLA-B*1819;HLA-B*1822;HLA-B*1818;HLA-B*1820;HLA-B*1821;HLA-B*1826;HLA-B*1827;HLA-B*1832;HLA-B*1831;HLA-B*1830;HLA-B*1828;HLA-B*1835;HLA-B*1839;HLA-B*1840;HLA-B*1841;HLA-B*1836;HLA-B*1838;HLA-B*1844;HLA-B*1834;HLA-B*1846;HLA-B*1801 |
| 4JQX | EECDSELEIKRY | 2;12 | HLA-B*4402;HLA-B*4403;HLA-B*4404;HLA-B*4405;HLA-B*4410;HLA-B*4414;HLA-B*4416;HLA-B*4417;HLA-B*4420;HLA-B*4421;HLA-B*4427;HLA-B*4428;HLA-B*4429;HLA-B*4430;HLA-B*4431;HLA-B*4704;HLA-B*4438;HLA-B*4433;HLA-B*4445;HLA-B*4436;HLA-B*4441;HLA-B*4443;HLA-B*4435;HLA-B*4442;HLA-B*4437;HLA-B*4447;HLA-B*4448;HLA-B*4454;HLA-B*4453;HLA-B*4451;HLA-B*4465;HLA-B*4462;HLA-B*4464;HLA-B*4459;HLA-B*4466;HLA-B*4477;HLA-B*4488;HLA-B*4473;HLA-B*4482;HLA-B*4470;HLA-B*4478;HLA-B*4474;HLA-B*4487;HLA-B*4471;HLA-B*4479;HLA-B*4468;HLA-B*4476;HLA-B*4496;HLA-B*4491;HLA-B*4499;HLA-B*4492;HLA-B*4498;HLA-B*4494;HLA-B*4489;HLA-B*4403 |
| 4JRX | LPEPLPQGQLTAY | 2;13 | HLA-B*3501;HLA-B*3502;HLA-B*3503;HLA-B*3504;HLA-B*3505;HLA-B*3506;HLA-B*3508;HLA-B*3509;HLA-B*3512;HLA-B*3514;HLA-B*3515;HLA-B*3517;HLA-B*3518;HLA-B*3521;HLA-B*3522;HLA-B*3523;HLA-B*3524;HLA-B*3530;HLA-B*3531;HLA-B*3532;HLA-B*3533;HLA-B*3534;HLA-B*3535;HLA-B*3537;HLA-B*3538;HLA-B*3539;HLA-B*3541;HLA-B*7802;HLA-B*7804;HLA-B*3542;HLA-B*3565;HLA-B*3555;HLA-B*3560;HLA-B*3548;HLA-B*3559;HLA-B*3544;HLA-B*3558;HLA-B*3561;HLA-B*3551;HLA-B*3557;HLA-B*3545;HLA-B*3564;HLA-B*3567;HLA-B*3566;HLA-B*3562;HLA-B*3579;HLA-B*3575;HLA-B*3571;HLA-B*3570;HLA-B*3568;HLA-B*3583;HLA-B*3593;HLA-B*3587;HLA-B*3586;HLA-B*3581;HLA-B*3589;HLA-B*3591;HLA-B*3594;HLA-B*3588;HLA-B*3599;HLA-B*3596;HLA-B*3598;HLA-B*3508 |
| 4JRY | LPEPLPQGQLTAY | 2;13 | HLA-B*3501;HLA-B*3502;HLA-B*3503;HLA-B*3504;HLA-B*3505;HLA-B*3506;HLA-B*3508;HLA-B*3509;HLA-B*3512;HLA-B*3514;HLA-B*3515;HLA-B*3517;HLA-B*3518;HLA-B*3521;HLA-B*3522;HLA-B*3523;HLA-B*3524;HLA-B*3530;HLA-B*3531;HLA-B*3532;HLA-B*3533;HLA-B*3534;HLA-B*3535;HLA-B*3537;HLA-B*3538;HLA-B*3539;HLA-B*3541;HLA-B*7802;HLA-B*7804;HLA-B*3542;HLA-B*3565;HLA-B*3555;HLA-B*3560;HLA-B*3548;HLA-B*3559;HLA-B*3544;HLA-B*3558;HLA-B*3561;HLA-B*3551;HLA-B*3557;HLA-B*3545;HLA-B*3564;HLA-B*3567;HLA-B*3566;HLA-B*3562;HLA-B*3579;HLA-B*3575;HLA-B*3571;HLA-B*3570;HLA-B*3568;HLA-B*3583;HLA-B*3593;HLA-B*3587;HLA-B*3586;HLA-B*3581;HLA-B*3589;HLA-B*3591;HLA-B*3594;HLA-B*3588;HLA-B*3599;HLA-B*3596;HLA-B*3598;HLA-B*3508 |
| 4K7F | VCWGELMNL | 2;9 | HLA-A*0201;HLA-A*0203;HLA-A*0204;HLA-A*0207;HLA-A*0209;HLA-A*0212;HLA-A*0213;HLA-A*0216;HLA-A*0217;HLA-A*0218;HLA-A*0219;HLA-A*0222;HLA-A*0224;HLA-A*0225;HLA-A*0226;HLA-A*0227;HLA-A*0233;HLA-A*0236;HLA-A*0237;HLA-A*0238;HLA-A*0239;HLA-A*0240;HLA-A*0249;HLA-A*0252;HLA-A*0266;HLA-A*0289;HLA-A*0271;HLA-A*0268;HLA-A*0274;HLA-A*0258;HLA-A*0280;HLA-A*0265;HLA-A*0286;HLA-A*0277;HLA-A*0260;HLA-A*0273;HLA-A*0285;HLA-A*0264;HLA-A*0275;HLA-A*0267;HLA-A*0295;HLA-A*0297;HLA-A*9201;HLA-A*0296;HLA-A*0293;HLA-A*9221;HLA-A*9231;HLA-A*9232;HLA-A*9214;HLA-A*9202;HLA-A*9210;HLA-A*9205;HLA-A*9217;HLA-A*9211;HLA-A*9204;HLA-A*9230;HLA-A*9234;HLA-A*9218;HLA-A*9247;HLA-A*9249;HLA-A*9251;HLA-A*9248;HLA-A*9256;HLA-A*9235;HLA-A*9252;HLA-A*9240;HLA-A*9253;HLA-A*9245;HLA-A*9277;HLA-A*9257;HLA-A*9271;HLA-A*9294;HLA-A*9264;HLA-A*9290;HLA-A*9266;HLA-A*9291;HLA-A*9289;HLA-A*9274;HLA-A*9262;HLA-A*9259;HLA-A*9263;HLA-A*9268;HLA-A*9288;HLA-A*9293;HLA-A*9287;HLA-A*9283;HLA-A*9267;HLA-A*9265;HLA-A*9282;HLA-A*9297;HLA-A*9298;HLA-A*9299;HLA-A*0201;HLA-A*0209;HLA-A*0266;HLA-A*0289;HLA-A*0275;HLA-A*0297;HLA-A*0296;HLA-A*9221;HLA-A*9232;HLA-A*9234;HLA-A*9240;HLA-A*9299 |
| 4L29 | YLLMWITQV | 2;9 | HLA-A*0201;HLA-A*0201 |
| 4L3C | YLLMWITQV | 2;9 | HLA-A*0201;HLA-A*0203;HLA-A*0204;HLA-A*0207;HLA-A*0209;HLA-A*0212;HLA-A*0213;HLA-A*0216;HLA-A*0217;HLA-A*0218;HLA-A*0219;HLA-A*0222;HLA-A*0224;HLA-A*0225;HLA-A*0226;HLA-A*0227;HLA-A*0233;HLA-A*0236;HLA-A*0237;HLA-A*0238;HLA-A*0239;HLA-A*0240;HLA-A*0249;HLA-A*0252;HLA-A*0266;HLA-A*0289;HLA-A*0271;HLA-A*0268;HLA-A*0274;HLA-A*0258;HLA-A*0280;HLA-A*0265;HLA-A*0286;HLA-A*0277;HLA-A*0260;HLA-A*0273;HLA-A*0285;HLA-A*0264;HLA-A*0275;HLA-A*0267;HLA-A*0295;HLA-A*0297;HLA-A*9201;HLA-A*0296;HLA-A*0293;HLA-A*9221;HLA-A*9231;HLA-A*9232;HLA-A*9214;HLA-A*9202;HLA-A*9210;HLA-A*9205;HLA-A*9217;HLA-A*9211;HLA-A*9204;HLA-A*9230;HLA-A*9234;HLA-A*9218;HLA-A*9247;HLA-A*9249;HLA-A*9251;HLA-A*9248;HLA-A*9256;HLA-A*9235;HLA-A*9252;HLA-A*9240;HLA-A*9253;HLA-A*9245;HLA-A*9277;HLA-A*9257;HLA-A*9271;HLA-A*9294;HLA-A*9264;HLA-A*9290;HLA-A*9266;HLA-A*9291;HLA-A*9289;HLA-A*9274;HLA-A*9262;HLA-A*9259;HLA-A*9263;HLA-A*9268;HLA-A*9288;HLA-A*9293;HLA-A*9287;HLA-A*9283;HLA-A*9267;HLA-A*9265;HLA-A*9282;HLA-A*9297;HLA-A*9298;HLA-A*9299;HLA-A*0201;HLA-A*0209;HLA-A*0266;HLA-A*0289;HLA-A*0275;HLA-A*0297;HLA-A*0296;HLA-A*9221;HLA-A*9232;HLA-A*9234;HLA-A*9240;HLA-A*9299 |
| 4L3E | ELAGIGILTV | 2;10 | HLA-A*0201;HLA-A*0203;HLA-A*0204;HLA-A*0207;HLA-A*0209;HLA-A*0212;HLA-A*0213;HLA-A*0216;HLA-A*0217;HLA-A*0218;HLA-A*0219;HLA-A*0222;HLA-A*0224;HLA-A*0225;HLA-A*0226;HLA-A*0227;HLA-A*0233;HLA-A*0236;HLA-A*0237;HLA-A*0238;HLA-A*0239;HLA-A*0240;HLA-A*0249;HLA-A*0252;HLA-A*0266;HLA-A*0289;HLA-A*0271;HLA-A*0268;HLA-A*0274;HLA-A*0258;HLA-A*0280;HLA-A*0265;HLA-A*0286;HLA-A*0277;HLA-A*0260;HLA-A*0273;HLA-A*0285;HLA-A*0264;HLA-A*0275;HLA-A*0267;HLA-A*0295;HLA-A*0297;HLA-A*9201;HLA-A*0296;HLA-A*0293;HLA-A*9221;HLA-A*9231;HLA-A*9232;HLA-A*9214;HLA-A*9202;HLA-A*9210;HLA-A*9205;HLA-A*9217;HLA-A*9211;HLA-A*9204;HLA-A*9230;HLA-A*9234;HLA-A*9218;HLA-A*9247;HLA-A*9249;HLA-A*9251;HLA-A*9248;HLA-A*9256;HLA-A*9235;HLA-A*9252;HLA-A*9240;HLA-A*9253;HLA-A*9245;HLA-A*9277;HLA-A*9257;HLA-A*9271;HLA-A*9294;HLA-A*9264;HLA-A*9290;HLA-A*9266;HLA-A*9291;HLA-A*9289;HLA-A*9274;HLA-A*9262;HLA-A*9259;HLA-A*9263;HLA-A*9268;HLA-A*9288;HLA-A*9293;HLA-A*9287;HLA-A*9283;HLA-A*9267;HLA-A*9265;HLA-A*9282;HLA-A*9297;HLA-A*9298;HLA-A*9299;HLA-A*0201;HLA-A*0209;HLA-A*0266;HLA-A*0289;HLA-A*0275;HLA-A*0297;HLA-A*0296;HLA-A*9221;HLA-A*9232;HLA-A*9234;HLA-A*9240;HLA-A*9299 |
| 4L8B | ASNEHMETM | 2;9 | H2-D1b;H2-D1b |
| 4L8C | ASDENMETM | 2;9 | H2-D1b;H2-D1b |
| 4L8D | ASNEDMETM | 2;9 | H2-D1b;H2-D1b |
| 4LCY | FTMRLLSPV | 2;9 | HLA-B*4601;HLA-B*4603;HLA-B*4606;HLA-B*4608;HLA-B*4609;HLA-B*4611;HLA-B*4612;HLA-B*4618;HLA-B*4613;HLA-B*4617;HLA-B*4619;HLA-B*4621;HLA-B*4601 |
| 4LNR | RPQVPLRPMTY | 2;11 | HLA-B*3501;HLA-B*3502;HLA-B*3503;HLA-B*3504;HLA-B*3505;HLA-B*3506;HLA-B*3508;HLA-B*3509;HLA-B*3512;HLA-B*3514;HLA-B*3515;HLA-B*3517;HLA-B*3518;HLA-B*3521;HLA-B*3522;HLA-B*3523;HLA-B*3524;HLA-B*3530;HLA-B*3531;HLA-B*3532;HLA-B*3533;HLA-B*3534;HLA-B*3535;HLA-B*3537;HLA-B*3538;HLA-B*3539;HLA-B*3541;HLA-B*7802;HLA-B*7804;HLA-B*3542;HLA-B*3565;HLA-B*3555;HLA-B*3560;HLA-B*3548;HLA-B*3559;HLA-B*3544;HLA-B*3558;HLA-B*3561;HLA-B*3551;HLA-B*3557;HLA-B*3545;HLA-B*3564;HLA-B*3567;HLA-B*3566;HLA-B*3562;HLA-B*3579;HLA-B*3575;HLA-B*3571;HLA-B*3570;HLA-B*3568;HLA-B*3583;HLA-B*3593;HLA-B*3587;HLA-B*3586;HLA-B*3581;HLA-B*3589;HLA-B*3591;HLA-B*3594;HLA-B*3588;HLA-B*3599;HLA-B*3596;HLA-B*3598;HLA-B*3501;HLA-B*3542;HLA-B*3557;HLA-B*3594 |
| 4MJ5 | TIAMELIRMIK | 2;11 | HLA-A*1101;HLA-A*1103;HLA-A*1104;HLA-A*1105;HLA-A*1107;HLA-A*1108;HLA-A*1109;HLA-A*1112;HLA-A*1113;HLA-A*1120;HLA-A*0312;HLA-A*1122;HLA-A*1115;HLA-A*1123;HLA-A*0278;HLA-A*1126;HLA-A*1127;HLA-A*1125;HLA-A*1124;HLA-A*1132;HLA-A*1129;HLA-A*1130;HLA-A*1131;HLA-A*1133;HLA-A*1139;HLA-A*1135;HLA-A*1136;HLA-A*1134;HLA-A*1141;HLA-A*1144;HLA-A*1148;HLA-A*1150;HLA-A*1159;HLA-A*1154;HLA-A*1101;HLA-A*1112;HLA-A*1132 |
| 4MJ6 | TMVMELIRMIK | 1;11 | HLA-A*1101;HLA-A*1103;HLA-A*1104;HLA-A*1105;HLA-A*1107;HLA-A*1108;HLA-A*1109;HLA-A*1112;HLA-A*1113;HLA-A*1120;HLA-A*0312;HLA-A*1122;HLA-A*1115;HLA-A*1123;HLA-A*0278;HLA-A*1126;HLA-A*1127;HLA-A*1125;HLA-A*1124;HLA-A*1132;HLA-A*1129;HLA-A*1130;HLA-A*1131;HLA-A*1133;HLA-A*1139;HLA-A*1135;HLA-A*1136;HLA-A*1134;HLA-A*1141;HLA-A*1144;HLA-A*1148;HLA-A*1150;HLA-A*1159;HLA-A*1154;HLA-A*1101;HLA-A*1112;HLA-A*1132 |
| 4MJI | TAFTIPSI | 2;8 | HLA-B*4406;HLA-B*5101;HLA-B*5102;HLA-B*5103;HLA-B*5104;HLA-B*5105;HLA-B*5106;HLA-B*5108;HLA-B*5109;HLA-B*5113;HLA-B*5114;HLA-B*5115;HLA-B*5116;HLA-B*5117;HLA-B*5118;HLA-B*5119;HLA-B*5121;HLA-B*5123;HLA-B*5124;HLA-B*5129;HLA-B*5301;HLA-B*5302;HLA-B*5304;HLA-B*5306;HLA-B*5307;HLA-B*5308;HLA-B*5135;HLA-B*5131;HLA-B*5138;HLA-B*5130;HLA-B*5136;HLA-B*5134;HLA-B*5132;HLA-B*5137;HLA-B*5142;HLA-B*5140;HLA-B*5146;HLA-B*5151;HLA-B*5148;HLA-B*5149;HLA-B*5145;HLA-B*5314;HLA-B*5159;HLA-B*5156;HLA-B*5315;HLA-B*5161;HLA-B*5155;HLA-B*5162;HLA-B*5153;HLA-B*5163;HLA-B*5164;HLA-B*5165;HLA-B*5175;HLA-B*5170;HLA-B*5184;HLA-B*5171;HLA-B*5169;HLA-B*5181;HLA-B*5180;HLA-B*5172;HLA-B*5182;HLA-B*5319;HLA-B*5176;HLA-B*5190;HLA-B*5193;HLA-B*5186;HLA-B*5185;HLA-B*5191;HLA-B*5194;HLA-B*5322;HLA-B*5188;HLA-B*5192;HLA-B*5195;HLA-B*5187;HLA-B*5320;HLA-B*5101;HLA-B*5130;HLA-B*5132;HLA-B*5151;HLA-B*5148;HLA-B*5176 |
| 4MNQ | ILAKFLHWL | 2;9 | HLA-A*0201;HLA-A*0203;HLA-A*0204;HLA-A*0207;HLA-A*0209;HLA-A*0212;HLA-A*0213;HLA-A*0216;HLA-A*0217;HLA-A*0218;HLA-A*0219;HLA-A*0222;HLA-A*0224;HLA-A*0225;HLA-A*0226;HLA-A*0227;HLA-A*0233;HLA-A*0236;HLA-A*0237;HLA-A*0238;HLA-A*0239;HLA-A*0240;HLA-A*0249;HLA-A*0252;HLA-A*0266;HLA-A*0289;HLA-A*0271;HLA-A*0268;HLA-A*0274;HLA-A*0258;HLA-A*0280;HLA-A*0265;HLA-A*0286;HLA-A*0277;HLA-A*0260;HLA-A*0273;HLA-A*0285;HLA-A*0264;HLA-A*0275;HLA-A*0267;HLA-A*0295;HLA-A*0297;HLA-A*9201;HLA-A*0296;HLA-A*0293;HLA-A*9221;HLA-A*9231;HLA-A*9232;HLA-A*9214;HLA-A*9202;HLA-A*9210;HLA-A*9205;HLA-A*9217;HLA-A*9211;HLA-A*9204;HLA-A*9230;HLA-A*9234;HLA-A*9218;HLA-A*9247;HLA-A*9249;HLA-A*9251;HLA-A*9248;HLA-A*9256;HLA-A*9235;HLA-A*9252;HLA-A*9240;HLA-A*9253;HLA-A*9245;HLA-A*9277;HLA-A*9257;HLA-A*9271;HLA-A*9294;HLA-A*9264;HLA-A*9290;HLA-A*9266;HLA-A*9291;HLA-A*9289;HLA-A*9274;HLA-A*9262;HLA-A*9259;HLA-A*9263;HLA-A*9268;HLA-A*9288;HLA-A*9293;HLA-A*9287;HLA-A*9283;HLA-A*9267;HLA-A*9265;HLA-A*9282;HLA-A*9297;HLA-A*9298;HLA-A*9299;HLA-A*0201;HLA-A*0209;HLA-A*0266;HLA-A*0289;HLA-A*0275;HLA-A*0297;HLA-A*0296;HLA-A*9221;HLA-A*9232;HLA-A*9234;HLA-A*9240;HLA-A*9299 |
| 4N8V | MLIYSMWGK | 2;9 | HLA-A*1101;HLA-A*1103;HLA-A*1104;HLA-A*1105;HLA-A*1107;HLA-A*1108;HLA-A*1109;HLA-A*1112;HLA-A*1113;HLA-A*1120;HLA-A*0312;HLA-A*1122;HLA-A*1115;HLA-A*1123;HLA-A*0278;HLA-A*1126;HLA-A*1127;HLA-A*1125;HLA-A*1124;HLA-A*1132;HLA-A*1129;HLA-A*1130;HLA-A*1131;HLA-A*1133;HLA-A*1139;HLA-A*1135;HLA-A*1136;HLA-A*1134;HLA-A*1141;HLA-A*1144;HLA-A*1148;HLA-A*1150;HLA-A*1159;HLA-A*1154;HLA-A*1101;HLA-A*1112;HLA-A*1132 |
| 4NNX | RQASLSISV | 2;9 | HLA-A*0201;HLA-A*0203;HLA-A*0204;HLA-A*0207;HLA-A*0209;HLA-A*0212;HLA-A*0213;HLA-A*0216;HLA-A*0217;HLA-A*0218;HLA-A*0219;HLA-A*0222;HLA-A*0224;HLA-A*0225;HLA-A*0226;HLA-A*0227;HLA-A*0233;HLA-A*0236;HLA-A*0237;HLA-A*0238;HLA-A*0239;HLA-A*0240;HLA-A*0249;HLA-A*0252;HLA-A*0266;HLA-A*0289;HLA-A*0271;HLA-A*0268;HLA-A*0274;HLA-A*0258;HLA-A*0280;HLA-A*0265;HLA-A*0286;HLA-A*0277;HLA-A*0260;HLA-A*0273;HLA-A*0285;HLA-A*0264;HLA-A*0275;HLA-A*0267;HLA-A*0295;HLA-A*0297;HLA-A*9201;HLA-A*0296;HLA-A*0293;HLA-A*9221;HLA-A*9231;HLA-A*9232;HLA-A*9214;HLA-A*9202;HLA-A*9210;HLA-A*9205;HLA-A*9217;HLA-A*9211;HLA-A*9204;HLA-A*9230;HLA-A*9234;HLA-A*9218;HLA-A*9247;HLA-A*9249;HLA-A*9251;HLA-A*9248;HLA-A*9256;HLA-A*9235;HLA-A*9252;HLA-A*9240;HLA-A*9253;HLA-A*9245;HLA-A*9277;HLA-A*9257;HLA-A*9271;HLA-A*9294;HLA-A*9264;HLA-A*9290;HLA-A*9266;HLA-A*9291;HLA-A*9289;HLA-A*9274;HLA-A*9262;HLA-A*9259;HLA-A*9263;HLA-A*9268;HLA-A*9288;HLA-A*9293;HLA-A*9287;HLA-A*9283;HLA-A*9267;HLA-A*9265;HLA-A*9282;HLA-A*9297;HLA-A*9298;HLA-A*9299;HLA-A*0201;HLA-A*0209;HLA-A*0266;HLA-A*0289;HLA-A*0275;HLA-A*0297;HLA-A*0296;HLA-A*9221;HLA-A*9232;HLA-A*9234;HLA-A*9240;HLA-A*9299 |
| 4NNY | RQASLSISV | 2;9 | HLA-A*0201;HLA-A*0203;HLA-A*0204;HLA-A*0207;HLA-A*0209;HLA-A*0212;HLA-A*0213;HLA-A*0216;HLA-A*0217;HLA-A*0218;HLA-A*0219;HLA-A*0222;HLA-A*0224;HLA-A*0225;HLA-A*0226;HLA-A*0227;HLA-A*0233;HLA-A*0236;HLA-A*0237;HLA-A*0238;HLA-A*0239;HLA-A*0240;HLA-A*0249;HLA-A*0252;HLA-A*0266;HLA-A*0289;HLA-A*0271;HLA-A*0268;HLA-A*0274;HLA-A*0258;HLA-A*0280;HLA-A*0265;HLA-A*0286;HLA-A*0277;HLA-A*0260;HLA-A*0273;HLA-A*0285;HLA-A*0264;HLA-A*0275;HLA-A*0267;HLA-A*0295;HLA-A*0297;HLA-A*9201;HLA-A*0296;HLA-A*0293;HLA-A*9221;HLA-A*9231;HLA-A*9232;HLA-A*9214;HLA-A*9202;HLA-A*9210;HLA-A*9205;HLA-A*9217;HLA-A*9211;HLA-A*9204;HLA-A*9230;HLA-A*9234;HLA-A*9218;HLA-A*9247;HLA-A*9249;HLA-A*9251;HLA-A*9248;HLA-A*9256;HLA-A*9235;HLA-A*9252;HLA-A*9240;HLA-A*9253;HLA-A*9245;HLA-A*9277;HLA-A*9257;HLA-A*9271;HLA-A*9294;HLA-A*9264;HLA-A*9290;HLA-A*9266;HLA-A*9291;HLA-A*9289;HLA-A*9274;HLA-A*9262;HLA-A*9259;HLA-A*9263;HLA-A*9268;HLA-A*9288;HLA-A*9293;HLA-A*9287;HLA-A*9283;HLA-A*9267;HLA-A*9265;HLA-A*9282;HLA-A*9297;HLA-A*9298;HLA-A*9299;HLA-A*0201;HLA-A*0209;HLA-A*0266;HLA-A*0289;HLA-A*0275;HLA-A*0297;HLA-A*0296;HLA-A*9221;HLA-A*9232;HLA-A*9234;HLA-A*9240;HLA-A*9299 |
| 4NO0 | RQASIELPSMAV | 2;12 | HLA-A*0201;HLA-A*0203;HLA-A*0204;HLA-A*0207;HLA-A*0209;HLA-A*0212;HLA-A*0213;HLA-A*0216;HLA-A*0217;HLA-A*0218;HLA-A*0219;HLA-A*0222;HLA-A*0224;HLA-A*0225;HLA-A*0226;HLA-A*0227;HLA-A*0233;HLA-A*0236;HLA-A*0237;HLA-A*0238;HLA-A*0239;HLA-A*0240;HLA-A*0249;HLA-A*0252;HLA-A*0266;HLA-A*0289;HLA-A*0271;HLA-A*0268;HLA-A*0274;HLA-A*0258;HLA-A*0280;HLA-A*0265;HLA-A*0286;HLA-A*0277;HLA-A*0260;HLA-A*0273;HLA-A*0285;HLA-A*0264;HLA-A*0275;HLA-A*0267;HLA-A*0295;HLA-A*0297;HLA-A*9201;HLA-A*0296;HLA-A*0293;HLA-A*9221;HLA-A*9231;HLA-A*9232;HLA-A*9214;HLA-A*9202;HLA-A*9210;HLA-A*9205;HLA-A*9217;HLA-A*9211;HLA-A*9204;HLA-A*9230;HLA-A*9234;HLA-A*9218;HLA-A*9247;HLA-A*9249;HLA-A*9251;HLA-A*9248;HLA-A*9256;HLA-A*9235;HLA-A*9252;HLA-A*9240;HLA-A*9253;HLA-A*9245;HLA-A*9277;HLA-A*9257;HLA-A*9271;HLA-A*9294;HLA-A*9264;HLA-A*9290;HLA-A*9266;HLA-A*9291;HLA-A*9289;HLA-A*9274;HLA-A*9262;HLA-A*9259;HLA-A*9263;HLA-A*9268;HLA-A*9288;HLA-A*9293;HLA-A*9287;HLA-A*9283;HLA-A*9267;HLA-A*9265;HLA-A*9282;HLA-A*9297;HLA-A*9298;HLA-A*9299;HLA-A*0201;HLA-A*0209;HLA-A*0266;HLA-A*0289;HLA-A*0275;HLA-A*0297;HLA-A*0296;HLA-A*9221;HLA-A*9232;HLA-A*9234;HLA-A*9240;HLA-A*9299 |
| 4NO2 | RQASIELPSMAV | 2;12 | HLA-A*0201;HLA-A*0203;HLA-A*0204;HLA-A*0207;HLA-A*0209;HLA-A*0212;HLA-A*0213;HLA-A*0216;HLA-A*0217;HLA-A*0218;HLA-A*0219;HLA-A*0222;HLA-A*0224;HLA-A*0225;HLA-A*0226;HLA-A*0227;HLA-A*0233;HLA-A*0236;HLA-A*0237;HLA-A*0238;HLA-A*0239;HLA-A*0240;HLA-A*0249;HLA-A*0252;HLA-A*0266;HLA-A*0289;HLA-A*0271;HLA-A*0268;HLA-A*0274;HLA-A*0258;HLA-A*0280;HLA-A*0265;HLA-A*0286;HLA-A*0277;HLA-A*0260;HLA-A*0273;HLA-A*0285;HLA-A*0264;HLA-A*0275;HLA-A*0267;HLA-A*0295;HLA-A*0297;HLA-A*9201;HLA-A*0296;HLA-A*0293;HLA-A*9221;HLA-A*9231;HLA-A*9232;HLA-A*9214;HLA-A*9202;HLA-A*9210;HLA-A*9205;HLA-A*9217;HLA-A*9211;HLA-A*9204;HLA-A*9230;HLA-A*9234;HLA-A*9218;HLA-A*9247;HLA-A*9249;HLA-A*9251;HLA-A*9248;HLA-A*9256;HLA-A*9235;HLA-A*9252;HLA-A*9240;HLA-A*9253;HLA-A*9245;HLA-A*9277;HLA-A*9257;HLA-A*9271;HLA-A*9294;HLA-A*9264;HLA-A*9290;HLA-A*9266;HLA-A*9291;HLA-A*9289;HLA-A*9274;HLA-A*9262;HLA-A*9259;HLA-A*9263;HLA-A*9268;HLA-A*9288;HLA-A*9293;HLA-A*9287;HLA-A*9283;HLA-A*9267;HLA-A*9265;HLA-A*9282;HLA-A*9297;HLA-A*9298;HLA-A*9299;HLA-A*0201;HLA-A*0209;HLA-A*0266;HLA-A*0289;HLA-A*0275;HLA-A*0297;HLA-A*0296;HLA-A*9221;HLA-A*9232;HLA-A*9234;HLA-A*9240;HLA-A*9299 |
| 4NO3 | RQISQDVKL | 2;9 | HLA-A*0201;HLA-A*0203;HLA-A*0204;HLA-A*0207;HLA-A*0209;HLA-A*0212;HLA-A*0213;HLA-A*0216;HLA-A*0217;HLA-A*0218;HLA-A*0219;HLA-A*0222;HLA-A*0224;HLA-A*0225;HLA-A*0226;HLA-A*0227;HLA-A*0233;HLA-A*0236;HLA-A*0237;HLA-A*0238;HLA-A*0239;HLA-A*0240;HLA-A*0249;HLA-A*0252;HLA-A*0266;HLA-A*0289;HLA-A*0271;HLA-A*0268;HLA-A*0274;HLA-A*0258;HLA-A*0280;HLA-A*0265;HLA-A*0286;HLA-A*0277;HLA-A*0260;HLA-A*0273;HLA-A*0285;HLA-A*0264;HLA-A*0275;HLA-A*0267;HLA-A*0295;HLA-A*0297;HLA-A*9201;HLA-A*0296;HLA-A*0293;HLA-A*9221;HLA-A*9231;HLA-A*9232;HLA-A*9214;HLA-A*9202;HLA-A*9210;HLA-A*9205;HLA-A*9217;HLA-A*9211;HLA-A*9204;HLA-A*9230;HLA-A*9234;HLA-A*9218;HLA-A*9247;HLA-A*9249;HLA-A*9251;HLA-A*9248;HLA-A*9256;HLA-A*9235;HLA-A*9252;HLA-A*9240;HLA-A*9253;HLA-A*9245;HLA-A*9277;HLA-A*9257;HLA-A*9271;HLA-A*9294;HLA-A*9264;HLA-A*9290;HLA-A*9266;HLA-A*9291;HLA-A*9289;HLA-A*9274;HLA-A*9262;HLA-A*9259;HLA-A*9263;HLA-A*9268;HLA-A*9288;HLA-A*9293;HLA-A*9287;HLA-A*9283;HLA-A*9267;HLA-A*9265;HLA-A*9282;HLA-A*9297;HLA-A*9298;HLA-A*9299;HLA-A*0201;HLA-A*0209;HLA-A*0266;HLA-A*0289;HLA-A*0275;HLA-A*0297;HLA-A*0296;HLA-A*9221;HLA-A*9232;HLA-A*9234;HLA-A*9240;HLA-A*9299 |
| 4NO5 | RQISQDVKL | 2;9 | HLA-A*0201;HLA-A*0203;HLA-A*0204;HLA-A*0207;HLA-A*0209;HLA-A*0212;HLA-A*0213;HLA-A*0216;HLA-A*0217;HLA-A*0218;HLA-A*0219;HLA-A*0222;HLA-A*0224;HLA-A*0225;HLA-A*0226;HLA-A*0227;HLA-A*0233;HLA-A*0236;HLA-A*0237;HLA-A*0238;HLA-A*0239;HLA-A*0240;HLA-A*0249;HLA-A*0252;HLA-A*0266;HLA-A*0289;HLA-A*0271;HLA-A*0268;HLA-A*0274;HLA-A*0258;HLA-A*0280;HLA-A*0265;HLA-A*0286;HLA-A*0277;HLA-A*0260;HLA-A*0273;HLA-A*0285;HLA-A*0264;HLA-A*0275;HLA-A*0267;HLA-A*0295;HLA-A*0297;HLA-A*9201;HLA-A*0296;HLA-A*0293;HLA-A*9221;HLA-A*9231;HLA-A*9232;HLA-A*9214;HLA-A*9202;HLA-A*9210;HLA-A*9205;HLA-A*9217;HLA-A*9211;HLA-A*9204;HLA-A*9230;HLA-A*9234;HLA-A*9218;HLA-A*9247;HLA-A*9249;HLA-A*9251;HLA-A*9248;HLA-A*9256;HLA-A*9235;HLA-A*9252;HLA-A*9240;HLA-A*9253;HLA-A*9245;HLA-A*9277;HLA-A*9257;HLA-A*9271;HLA-A*9294;HLA-A*9264;HLA-A*9290;HLA-A*9266;HLA-A*9291;HLA-A*9289;HLA-A*9274;HLA-A*9262;HLA-A*9259;HLA-A*9263;HLA-A*9268;HLA-A*9288;HLA-A*9293;HLA-A*9287;HLA-A*9283;HLA-A*9267;HLA-A*9265;HLA-A*9282;HLA-A*9297;HLA-A*9298;HLA-A*9299;HLA-A*0201;HLA-A*0209;HLA-A*0266;HLA-A*0289;HLA-A*0275;HLA-A*0297;HLA-A*0296;HLA-A*9221;HLA-A*9232;HLA-A*9234;HLA-A*9240;HLA-A*9299 |
| 4NQV | CTELKLSDY | 2;9 | HLA-A*0101;HLA-A*0103;HLA-A*0106;HLA-A*0108;HLA-A*3601;HLA-A*3602;HLA-A*3603;HLA-A*0110;HLA-A*0112;HLA-A*0114;HLA-A*3604;HLA-A*0119;HLA-A*0125;HLA-A*0121;HLA-A*0126;HLA-A*0130;HLA-A*0132;HLA-A*0137;HLA-A*0135;HLA-A*0138;HLA-A*0136;HLA-A*0142;HLA-A*0144;HLA-A*0145;HLA-A*0140;HLA-A*0139;HLA-A*0141;HLA-A*0155;HLA-A*0146;HLA-A*0154;HLA-A*0148;HLA-A*3605;HLA-A*0150;HLA-A*0149;HLA-A*0101;HLA-A*0132;HLA-A*0137;HLA-A*0145 |
| 4NQX | CTELKLNDY | 2;9 | HLA-A*0101;HLA-A*0103;HLA-A*0106;HLA-A*0108;HLA-A*3601;HLA-A*3602;HLA-A*3603;HLA-A*0110;HLA-A*0112;HLA-A*0114;HLA-A*3604;HLA-A*0119;HLA-A*0125;HLA-A*0121;HLA-A*0126;HLA-A*0130;HLA-A*0132;HLA-A*0137;HLA-A*0135;HLA-A*0138;HLA-A*0136;HLA-A*0142;HLA-A*0144;HLA-A*0145;HLA-A*0140;HLA-A*0139;HLA-A*0141;HLA-A*0155;HLA-A*0146;HLA-A*0154;HLA-A*0148;HLA-A*3605;HLA-A*0150;HLA-A*0149;HLA-A*0101;HLA-A*0132;HLA-A*0137;HLA-A*0145 |
| 4NSK | KAPYNFATM | 2;9 | H2-D1b;H2-D1b |
| 4NT6 | GILGFVFTL | 2;9 | HLA-C*0801;HLA-C*0802;HLA-C*0803;HLA-C*0804;HLA-C*0806;HLA-C*0807;HLA-C*0808;HLA-C*0809;HLA-C*0811;HLA-C*0812;HLA-C*0815;HLA-C*0819;HLA-C*0824;HLA-C*0822;HLA-C*0827;HLA-C*0823;HLA-C*0820;HLA-C*0828;HLA-C*0829;HLA-C*0831;HLA-C*0830;HLA-C*0801;HLA-C*0824;HLA-C*0822;HLA-C*0820 |
| 4O2E | SHVAVENAL | 2;9 | HLA-B*1401;HLA-B*3901;HLA-B*3903;HLA-B*3906;HLA-B*3909;HLA-B*3914;HLA-B*3915;HLA-B*3918;HLA-B*3924;HLA-B*3926;HLA-B*3936;HLA-B*3928;HLA-B*3938;HLA-B*3933;HLA-B*3929;HLA-B*3935;HLA-B*3930;HLA-B*3932;HLA-B*3934;HLA-B*3942;HLA-B*3943;HLA-B*3944;HLA-B*1408;HLA-B*3946;HLA-B*3947;HLA-B*3948;HLA-B*3950;HLA-B*0769;HLA-B*1410;HLA-B*1412;HLA-B*3953;HLA-B*0785;HLA-B*3951;HLA-B*3957;HLA-B*3901;HLA-B*3946;HLA-B*3951 |
| 4O2F | HVAVENAL | 1;8 | HLA-B*1401;HLA-B*3901;HLA-B*3903;HLA-B*3906;HLA-B*3909;HLA-B*3914;HLA-B*3915;HLA-B*3918;HLA-B*3924;HLA-B*3926;HLA-B*3936;HLA-B*3928;HLA-B*3938;HLA-B*3933;HLA-B*3929;HLA-B*3935;HLA-B*3930;HLA-B*3932;HLA-B*3934;HLA-B*3942;HLA-B*3943;HLA-B*3944;HLA-B*1408;HLA-B*3946;HLA-B*3947;HLA-B*3948;HLA-B*3950;HLA-B*0769;HLA-B*1410;HLA-B*1412;HLA-B*3953;HLA-B*0785;HLA-B*3951;HLA-B*3957;HLA-B*3901;HLA-B*3946;HLA-B*3951 |
| 4PG2 | CSLWNGPHL | 2;9 | H2-D1b;H2-D1b |
| 4PG9 | FAPGNYPAL | 2;9 | MH1-K1b;MH1-K1b |
| 4PGB | FAPGNWPAL | 2;9 | MH1-K1b;MH1-K1b |
| 4PGD | FAPGNYPAF | 2;9 | MH1-K1b;MH1-K1b |
| 4PGE | FAPGNYPAW | 2;9 | MH1-K1b;MH1-K1b |
| 4PR5 | HPVGDADYFEY | 2;11 | HLA-B*3501;HLA-B*3502;HLA-B*3503;HLA-B*3504;HLA-B*3505;HLA-B*3506;HLA-B*3508;HLA-B*3509;HLA-B*3512;HLA-B*3514;HLA-B*3515;HLA-B*3517;HLA-B*3518;HLA-B*3521;HLA-B*3522;HLA-B*3523;HLA-B*3524;HLA-B*3530;HLA-B*3531;HLA-B*3532;HLA-B*3533;HLA-B*3534;HLA-B*3535;HLA-B*3537;HLA-B*3538;HLA-B*3539;HLA-B*3541;HLA-B*7802;HLA-B*7804;HLA-B*3542;HLA-B*3565;HLA-B*3555;HLA-B*3560;HLA-B*3548;HLA-B*3559;HLA-B*3544;HLA-B*3558;HLA-B*3561;HLA-B*3551;HLA-B*3557;HLA-B*3545;HLA-B*3564;HLA-B*3567;HLA-B*3566;HLA-B*3562;HLA-B*3579;HLA-B*3575;HLA-B*3571;HLA-B*3570;HLA-B*3568;HLA-B*3583;HLA-B*3593;HLA-B*3587;HLA-B*3586;HLA-B*3581;HLA-B*3589;HLA-B*3591;HLA-B*3594;HLA-B*3588;HLA-B*3599;HLA-B*3596;HLA-B*3598;HLA-B*3501;HLA-B*3542;HLA-B*3557;HLA-B*3594 |
| 4PRA | HPVGQADYFEY | 2;11 | HLA-B*3501;HLA-B*3502;HLA-B*3503;HLA-B*3504;HLA-B*3505;HLA-B*3506;HLA-B*3508;HLA-B*3509;HLA-B*3512;HLA-B*3514;HLA-B*3515;HLA-B*3517;HLA-B*3518;HLA-B*3521;HLA-B*3522;HLA-B*3523;HLA-B*3524;HLA-B*3530;HLA-B*3531;HLA-B*3532;HLA-B*3533;HLA-B*3534;HLA-B*3535;HLA-B*3537;HLA-B*3538;HLA-B*3539;HLA-B*3541;HLA-B*7802;HLA-B*7804;HLA-B*3542;HLA-B*3565;HLA-B*3555;HLA-B*3560;HLA-B*3548;HLA-B*3559;HLA-B*3544;HLA-B*3558;HLA-B*3561;HLA-B*3551;HLA-B*3557;HLA-B*3545;HLA-B*3564;HLA-B*3567;HLA-B*3566;HLA-B*3562;HLA-B*3579;HLA-B*3575;HLA-B*3571;HLA-B*3570;HLA-B*3568;HLA-B*3583;HLA-B*3593;HLA-B*3587;HLA-B*3586;HLA-B*3581;HLA-B*3589;HLA-B*3591;HLA-B*3594;HLA-B*3588;HLA-B*3599;HLA-B*3596;HLA-B*3598;HLA-B*3501;HLA-B*3542;HLA-B*3557;HLA-B*3594 |
| 4PRB | HPVAEADYFEY | 2;11 | HLA-B*3501;HLA-B*3502;HLA-B*3503;HLA-B*3504;HLA-B*3505;HLA-B*3506;HLA-B*3508;HLA-B*3509;HLA-B*3512;HLA-B*3514;HLA-B*3515;HLA-B*3517;HLA-B*3518;HLA-B*3521;HLA-B*3522;HLA-B*3523;HLA-B*3524;HLA-B*3530;HLA-B*3531;HLA-B*3532;HLA-B*3533;HLA-B*3534;HLA-B*3535;HLA-B*3537;HLA-B*3538;HLA-B*3539;HLA-B*3541;HLA-B*7802;HLA-B*7804;HLA-B*3542;HLA-B*3565;HLA-B*3555;HLA-B*3560;HLA-B*3548;HLA-B*3559;HLA-B*3544;HLA-B*3558;HLA-B*3561;HLA-B*3551;HLA-B*3557;HLA-B*3545;HLA-B*3564;HLA-B*3567;HLA-B*3566;HLA-B*3562;HLA-B*3579;HLA-B*3575;HLA-B*3571;HLA-B*3570;HLA-B*3568;HLA-B*3583;HLA-B*3593;HLA-B*3587;HLA-B*3586;HLA-B*3581;HLA-B*3589;HLA-B*3591;HLA-B*3594;HLA-B*3588;HLA-B*3599;HLA-B*3596;HLA-B*3598;HLA-B*3508 |
| 4PRD | HPVGDADYFEY | 2;11 | HLA-B*3508;HLA-B*3508 |
| 4PRE | HPVGQADYFEY | 2;11 | HLA-B*3508;HLA-B*3508 |
| 4PRH | HPVGDADYFEY | 2;11 | HLA-B*3501;HLA-B*3502;HLA-B*3503;HLA-B*3504;HLA-B*3505;HLA-B*3506;HLA-B*3508;HLA-B*3509;HLA-B*3512;HLA-B*3514;HLA-B*3515;HLA-B*3517;HLA-B*3518;HLA-B*3521;HLA-B*3522;HLA-B*3523;HLA-B*3524;HLA-B*3530;HLA-B*3531;HLA-B*3532;HLA-B*3533;HLA-B*3534;HLA-B*3535;HLA-B*3537;HLA-B*3538;HLA-B*3539;HLA-B*3541;HLA-B*7802;HLA-B*7804;HLA-B*3542;HLA-B*3565;HLA-B*3555;HLA-B*3560;HLA-B*3548;HLA-B*3559;HLA-B*3544;HLA-B*3558;HLA-B*3561;HLA-B*3551;HLA-B*3557;HLA-B*3545;HLA-B*3564;HLA-B*3567;HLA-B*3566;HLA-B*3562;HLA-B*3579;HLA-B*3575;HLA-B*3571;HLA-B*3570;HLA-B*3568;HLA-B*3583;HLA-B*3593;HLA-B*3587;HLA-B*3586;HLA-B*3581;HLA-B*3589;HLA-B*3591;HLA-B*3594;HLA-B*3588;HLA-B*3599;HLA-B*3596;HLA-B*3598;HLA-B*3508 |
| 4PRI | HPVGEADYFEY | 2;11 | HLA-B*3501;HLA-B*3502;HLA-B*3503;HLA-B*3504;HLA-B*3505;HLA-B*3506;HLA-B*3508;HLA-B*3509;HLA-B*3512;HLA-B*3514;HLA-B*3515;HLA-B*3517;HLA-B*3518;HLA-B*3521;HLA-B*3522;HLA-B*3523;HLA-B*3524;HLA-B*3530;HLA-B*3531;HLA-B*3532;HLA-B*3533;HLA-B*3534;HLA-B*3535;HLA-B*3537;HLA-B*3538;HLA-B*3539;HLA-B*3541;HLA-B*7802;HLA-B*7804;HLA-B*3542;HLA-B*3565;HLA-B*3555;HLA-B*3560;HLA-B*3548;HLA-B*3559;HLA-B*3544;HLA-B*3558;HLA-B*3561;HLA-B*3551;HLA-B*3557;HLA-B*3545;HLA-B*3564;HLA-B*3567;HLA-B*3566;HLA-B*3562;HLA-B*3579;HLA-B*3575;HLA-B*3571;HLA-B*3570;HLA-B*3568;HLA-B*3583;HLA-B*3593;HLA-B*3587;HLA-B*3586;HLA-B*3581;HLA-B*3589;HLA-B*3591;HLA-B*3594;HLA-B*3588;HLA-B*3599;HLA-B*3596;HLA-B*3598;HLA-B*3508 |
| 4PRN | HPVAEADYFEY | 2;11 | HLA-B*3501;HLA-B*3502;HLA-B*3503;HLA-B*3504;HLA-B*3505;HLA-B*3506;HLA-B*3508;HLA-B*3509;HLA-B*3512;HLA-B*3514;HLA-B*3515;HLA-B*3517;HLA-B*3518;HLA-B*3521;HLA-B*3522;HLA-B*3523;HLA-B*3524;HLA-B*3530;HLA-B*3531;HLA-B*3532;HLA-B*3533;HLA-B*3534;HLA-B*3535;HLA-B*3537;HLA-B*3538;HLA-B*3539;HLA-B*3541;HLA-B*7802;HLA-B*7804;HLA-B*3542;HLA-B*3565;HLA-B*3555;HLA-B*3560;HLA-B*3548;HLA-B*3559;HLA-B*3544;HLA-B*3558;HLA-B*3561;HLA-B*3551;HLA-B*3557;HLA-B*3545;HLA-B*3564;HLA-B*3567;HLA-B*3566;HLA-B*3562;HLA-B*3579;HLA-B*3575;HLA-B*3571;HLA-B*3570;HLA-B*3568;HLA-B*3583;HLA-B*3593;HLA-B*3587;HLA-B*3586;HLA-B*3581;HLA-B*3589;HLA-B*3591;HLA-B*3594;HLA-B*3588;HLA-B*3599;HLA-B*3596;HLA-B*3598;HLA-B*3501;HLA-B*3542;HLA-B*3557;HLA-B*3594 |
| 4PRP | HPVGQADYFEY | 2;11 | HLA-B*3501;HLA-B*3502;HLA-B*3503;HLA-B*3504;HLA-B*3505;HLA-B*3506;HLA-B*3508;HLA-B*3509;HLA-B*3512;HLA-B*3514;HLA-B*3515;HLA-B*3517;HLA-B*3518;HLA-B*3521;HLA-B*3522;HLA-B*3523;HLA-B*3524;HLA-B*3530;HLA-B*3531;HLA-B*3532;HLA-B*3533;HLA-B*3534;HLA-B*3535;HLA-B*3537;HLA-B*3538;HLA-B*3539;HLA-B*3541;HLA-B*7802;HLA-B*7804;HLA-B*3542;HLA-B*3565;HLA-B*3555;HLA-B*3560;HLA-B*3548;HLA-B*3559;HLA-B*3544;HLA-B*3558;HLA-B*3561;HLA-B*3551;HLA-B*3557;HLA-B*3545;HLA-B*3564;HLA-B*3567;HLA-B*3566;HLA-B*3562;HLA-B*3579;HLA-B*3575;HLA-B*3571;HLA-B*3570;HLA-B*3568;HLA-B*3583;HLA-B*3593;HLA-B*3587;HLA-B*3586;HLA-B*3581;HLA-B*3589;HLA-B*3591;HLA-B*3594;HLA-B*3588;HLA-B*3599;HLA-B*3596;HLA-B*3598;HLA-B*3501;HLA-B*3542;HLA-B*3557;HLA-B*3594 |
| 4QOK | EAAGIGILTV | 2;10 | HLA-A*0201;HLA-A*0203;HLA-A*0204;HLA-A*0207;HLA-A*0209;HLA-A*0212;HLA-A*0213;HLA-A*0216;HLA-A*0217;HLA-A*0218;HLA-A*0219;HLA-A*0222;HLA-A*0224;HLA-A*0225;HLA-A*0226;HLA-A*0227;HLA-A*0233;HLA-A*0236;HLA-A*0237;HLA-A*0238;HLA-A*0239;HLA-A*0240;HLA-A*0249;HLA-A*0252;HLA-A*0266;HLA-A*0289;HLA-A*0271;HLA-A*0268;HLA-A*0274;HLA-A*0258;HLA-A*0280;HLA-A*0265;HLA-A*0286;HLA-A*0277;HLA-A*0260;HLA-A*0273;HLA-A*0285;HLA-A*0264;HLA-A*0275;HLA-A*0267;HLA-A*0295;HLA-A*0297;HLA-A*9201;HLA-A*0296;HLA-A*0293;HLA-A*9221;HLA-A*9231;HLA-A*9232;HLA-A*9214;HLA-A*9202;HLA-A*9210;HLA-A*9205;HLA-A*9217;HLA-A*9211;HLA-A*9204;HLA-A*9230;HLA-A*9234;HLA-A*9218;HLA-A*9247;HLA-A*9249;HLA-A*9251;HLA-A*9248;HLA-A*9256;HLA-A*9235;HLA-A*9252;HLA-A*9240;HLA-A*9253;HLA-A*9245;HLA-A*9277;HLA-A*9257;HLA-A*9271;HLA-A*9294;HLA-A*9264;HLA-A*9290;HLA-A*9266;HLA-A*9291;HLA-A*9289;HLA-A*9274;HLA-A*9262;HLA-A*9259;HLA-A*9263;HLA-A*9268;HLA-A*9288;HLA-A*9293;HLA-A*9287;HLA-A*9283;HLA-A*9267;HLA-A*9265;HLA-A*9282;HLA-A*9297;HLA-A*9298;HLA-A*9299;HLA-A*0201;HLA-A*0209;HLA-A*0266;HLA-A*0289;HLA-A*0275;HLA-A*0297;HLA-A*0296;HLA-A*9221;HLA-A*9232;HLA-A*9234;HLA-A*9240;HLA-A*9299 |
| 4QRP | HSKKKCDEL | 2;9 | HLA-B*0801;HLA-B*0807;HLA-B*0809;HLA-B*0811;HLA-B*0812;HLA-B*0813;HLA-B*0814;HLA-B*0827;HLA-B*0822;HLA-B*0820;HLA-B*0825;HLA-B*0821;HLA-B*0818;HLA-B*0828;HLA-B*0831;HLA-B*0833;HLA-B*0835;HLA-B*0837;HLA-B*0840;HLA-B*0839;HLA-B*0842;HLA-B*0843;HLA-B*0853;HLA-B*0858;HLA-B*0848;HLA-B*0849;HLA-B*0860;HLA-B*0855;HLA-B*9580;HLA-B*0801;HLA-B*0818;HLA-B*0839 |
| 4QRQ | HSKKKCDEL | 2;9 | HLA-B*0801;HLA-B*0807;HLA-B*0809;HLA-B*0811;HLA-B*0812;HLA-B*0813;HLA-B*0814;HLA-B*0827;HLA-B*0822;HLA-B*0820;HLA-B*0825;HLA-B*0821;HLA-B*0818;HLA-B*0828;HLA-B*0831;HLA-B*0833;HLA-B*0835;HLA-B*0837;HLA-B*0840;HLA-B*0839;HLA-B*0842;HLA-B*0843;HLA-B*0853;HLA-B*0858;HLA-B*0848;HLA-B*0849;HLA-B*0860;HLA-B*0855;HLA-B*9580;HLA-B*0801;HLA-B*0818;HLA-B*0839 |
| 4QRR | IPSINVHHY | 2;9 | HLA-B*3501;HLA-B*3502;HLA-B*3503;HLA-B*3504;HLA-B*3505;HLA-B*3506;HLA-B*3508;HLA-B*3509;HLA-B*3512;HLA-B*3514;HLA-B*3515;HLA-B*3517;HLA-B*3518;HLA-B*3521;HLA-B*3522;HLA-B*3523;HLA-B*3524;HLA-B*3530;HLA-B*3531;HLA-B*3532;HLA-B*3533;HLA-B*3534;HLA-B*3535;HLA-B*3537;HLA-B*3538;HLA-B*3539;HLA-B*3541;HLA-B*7802;HLA-B*7804;HLA-B*3542;HLA-B*3565;HLA-B*3555;HLA-B*3560;HLA-B*3548;HLA-B*3559;HLA-B*3544;HLA-B*3558;HLA-B*3561;HLA-B*3551;HLA-B*3557;HLA-B*3545;HLA-B*3564;HLA-B*3567;HLA-B*3566;HLA-B*3562;HLA-B*3579;HLA-B*3575;HLA-B*3571;HLA-B*3570;HLA-B*3568;HLA-B*3583;HLA-B*3593;HLA-B*3587;HLA-B*3586;HLA-B*3581;HLA-B*3589;HLA-B*3591;HLA-B*3594;HLA-B*3588;HLA-B*3599;HLA-B*3596;HLA-B*3598;HLA-B*3501;HLA-B*3542;HLA-B*3557;HLA-B*3594 |
| 4QRS | ELKRKMIYM | 2;9 | HLA-B*0801;HLA-B*0807;HLA-B*0809;HLA-B*0811;HLA-B*0812;HLA-B*0813;HLA-B*0814;HLA-B*0827;HLA-B*0822;HLA-B*0820;HLA-B*0825;HLA-B*0821;HLA-B*0818;HLA-B*0828;HLA-B*0831;HLA-B*0833;HLA-B*0835;HLA-B*0837;HLA-B*0840;HLA-B*0839;HLA-B*0842;HLA-B*0843;HLA-B*0853;HLA-B*0858;HLA-B*0848;HLA-B*0849;HLA-B*0860;HLA-B*0855;HLA-B*9580;HLA-B*0801;HLA-B*0818;HLA-B*0839 |
| 4QRT | ELNRKMIYM | 2;9 | HLA-B*0801;HLA-B*0801 |
| 4QRU | ELRRKMMYM | 2;9 | HLA-B*0801;HLA-B*0801 |
| 4U1H | TPQDLNTML | 2;9 | HLA-B*0702;HLA-B*0704;HLA-B*0705;HLA-B*0706;HLA-B*0707;HLA-B*0709;HLA-B*0712;HLA-B*0714;HLA-B*0717;HLA-B*0718;HLA-B*0719;HLA-B*0720;HLA-B*0722;HLA-B*0724;HLA-B*0725;HLA-B*0726;HLA-B*0728;HLA-B*4201;HLA-B*4204;HLA-B*5510;HLA-B*6701;HLA-B*8101;HLA-B*0734;HLA-B*0742;HLA-B*0744;HLA-B*0745;HLA-B*4205;HLA-B*0740;HLA-B*0733;HLA-B*4208;HLA-B*0731;HLA-B*4206;HLA-B*0735;HLA-B*0743;HLA-B*8102;HLA-B*0748;HLA-B*0751;HLA-B*0758;HLA-B*0760;HLA-B*0755;HLA-B*0753;HLA-B*0759;HLA-B*0752;HLA-B*8103;HLA-B*0766;HLA-B*0764;HLA-B*0761;HLA-B*0762;HLA-B*4210;HLA-B*4212;HLA-B*0777;HLA-B*0795;HLA-B*0796;HLA-B*0799;HLA-B*4213;HLA-B*0774;HLA-B*0780;HLA-B*0778;HLA-B*0784;HLA-B*0798;HLA-B*0782;HLA-B*0787;HLA-B*0790;HLA-B*0793;HLA-B*0789;HLA-B*0702;HLA-B*0744;HLA-B*0758;HLA-B*0759;HLA-B*0761;HLA-B*0782;HLA-B*0793 |
| 4U1I | TPQDLNTML | 2;9 | HLA-B*0702;HLA-B*0704;HLA-B*0705;HLA-B*0706;HLA-B*0707;HLA-B*0709;HLA-B*0712;HLA-B*0714;HLA-B*0717;HLA-B*0718;HLA-B*0719;HLA-B*0720;HLA-B*0722;HLA-B*0724;HLA-B*0725;HLA-B*0726;HLA-B*0728;HLA-B*4201;HLA-B*4204;HLA-B*5510;HLA-B*6701;HLA-B*8101;HLA-B*0734;HLA-B*0742;HLA-B*0744;HLA-B*0745;HLA-B*4205;HLA-B*0740;HLA-B*0733;HLA-B*4208;HLA-B*0731;HLA-B*4206;HLA-B*0735;HLA-B*0743;HLA-B*8102;HLA-B*0748;HLA-B*0751;HLA-B*0758;HLA-B*0760;HLA-B*0755;HLA-B*0753;HLA-B*0759;HLA-B*0752;HLA-B*8103;HLA-B*0766;HLA-B*0764;HLA-B*0761;HLA-B*0762;HLA-B*4210;HLA-B*4212;HLA-B*0777;HLA-B*0795;HLA-B*0796;HLA-B*0799;HLA-B*4213;HLA-B*0774;HLA-B*0780;HLA-B*0778;HLA-B*0784;HLA-B*0798;HLA-B*0782;HLA-B*0787;HLA-B*0790;HLA-B*0793;HLA-B*0789;HLA-B*8101;HLA-B*8102;HLA-B*8103 |
| 4U1J | TPQDLNTML | 2;9 | HLA-B*0702;HLA-B*0704;HLA-B*0705;HLA-B*0706;HLA-B*0707;HLA-B*0709;HLA-B*0712;HLA-B*0714;HLA-B*0717;HLA-B*0718;HLA-B*0719;HLA-B*0720;HLA-B*0722;HLA-B*0724;HLA-B*0725;HLA-B*0726;HLA-B*0728;HLA-B*4201;HLA-B*4204;HLA-B*5510;HLA-B*6701;HLA-B*8101;HLA-B*0734;HLA-B*0742;HLA-B*0744;HLA-B*0745;HLA-B*4205;HLA-B*0740;HLA-B*0733;HLA-B*4208;HLA-B*0731;HLA-B*4206;HLA-B*0735;HLA-B*0743;HLA-B*8102;HLA-B*0748;HLA-B*0751;HLA-B*0758;HLA-B*0760;HLA-B*0755;HLA-B*0753;HLA-B*0759;HLA-B*0752;HLA-B*8103;HLA-B*0766;HLA-B*0764;HLA-B*0761;HLA-B*0762;HLA-B*4210;HLA-B*4212;HLA-B*0777;HLA-B*0795;HLA-B*0796;HLA-B*0799;HLA-B*4213;HLA-B*0774;HLA-B*0780;HLA-B*0778;HLA-B*0784;HLA-B*0798;HLA-B*0782;HLA-B*0787;HLA-B*0790;HLA-B*0793;HLA-B*0789;HLA-B*4201 |
| 4U1K | RPQVPLRPM | 2;9 | HLA-B*0702;HLA-B*0704;HLA-B*0705;HLA-B*0706;HLA-B*0707;HLA-B*0709;HLA-B*0712;HLA-B*0714;HLA-B*0717;HLA-B*0718;HLA-B*0719;HLA-B*0720;HLA-B*0722;HLA-B*0724;HLA-B*0725;HLA-B*0726;HLA-B*0728;HLA-B*4201;HLA-B*4204;HLA-B*5510;HLA-B*6701;HLA-B*8101;HLA-B*0734;HLA-B*0742;HLA-B*0744;HLA-B*0745;HLA-B*4205;HLA-B*0740;HLA-B*0733;HLA-B*4208;HLA-B*0731;HLA-B*4206;HLA-B*0735;HLA-B*0743;HLA-B*8102;HLA-B*0748;HLA-B*0751;HLA-B*0758;HLA-B*0760;HLA-B*0755;HLA-B*0753;HLA-B*0759;HLA-B*0752;HLA-B*8103;HLA-B*0766;HLA-B*0764;HLA-B*0761;HLA-B*0762;HLA-B*4210;HLA-B*4212;HLA-B*0777;HLA-B*0795;HLA-B*0796;HLA-B*0799;HLA-B*4213;HLA-B*0774;HLA-B*0780;HLA-B*0778;HLA-B*0784;HLA-B*0798;HLA-B*0782;HLA-B*0787;HLA-B*0790;HLA-B*0793;HLA-B*0789;HLA-B*0702;HLA-B*0744;HLA-B*0758;HLA-B*0759;HLA-B*0761;HLA-B*0782;HLA-B*0793 |
| 4U1L | RPQVPLRPM | 2;9 | HLA-B*0702;HLA-B*0704;HLA-B*0705;HLA-B*0706;HLA-B*0707;HLA-B*0709;HLA-B*0712;HLA-B*0714;HLA-B*0717;HLA-B*0718;HLA-B*0719;HLA-B*0720;HLA-B*0722;HLA-B*0724;HLA-B*0725;HLA-B*0726;HLA-B*0728;HLA-B*4201;HLA-B*4204;HLA-B*5510;HLA-B*6701;HLA-B*8101;HLA-B*0734;HLA-B*0742;HLA-B*0744;HLA-B*0745;HLA-B*4205;HLA-B*0740;HLA-B*0733;HLA-B*4208;HLA-B*0731;HLA-B*4206;HLA-B*0735;HLA-B*0743;HLA-B*8102;HLA-B*0748;HLA-B*0751;HLA-B*0758;HLA-B*0760;HLA-B*0755;HLA-B*0753;HLA-B*0759;HLA-B*0752;HLA-B*8103;HLA-B*0766;HLA-B*0764;HLA-B*0761;HLA-B*0762;HLA-B*4210;HLA-B*4212;HLA-B*0777;HLA-B*0795;HLA-B*0796;HLA-B*0799;HLA-B*4213;HLA-B*0774;HLA-B*0780;HLA-B*0778;HLA-B*0784;HLA-B*0798;HLA-B*0782;HLA-B*0787;HLA-B*0790;HLA-B*0793;HLA-B*0789;HLA-B*8101;HLA-B*8102;HLA-B*8103 |
| 4U1N | RPQVPLRPM | 2;9 | HLA-B*4202;HLA-B*4209;HLA-B*4202 |
| 4U1S | FPRPWLHGL | 2;9 | HLA-B*0702;HLA-B*0704;HLA-B*0705;HLA-B*0706;HLA-B*0707;HLA-B*0709;HLA-B*0712;HLA-B*0714;HLA-B*0717;HLA-B*0718;HLA-B*0719;HLA-B*0720;HLA-B*0722;HLA-B*0724;HLA-B*0725;HLA-B*0726;HLA-B*0728;HLA-B*4201;HLA-B*4204;HLA-B*5510;HLA-B*6701;HLA-B*8101;HLA-B*0734;HLA-B*0742;HLA-B*0744;HLA-B*0745;HLA-B*4205;HLA-B*0740;HLA-B*0733;HLA-B*4208;HLA-B*0731;HLA-B*4206;HLA-B*0735;HLA-B*0743;HLA-B*8102;HLA-B*0748;HLA-B*0751;HLA-B*0758;HLA-B*0760;HLA-B*0755;HLA-B*0753;HLA-B*0759;HLA-B*0752;HLA-B*8103;HLA-B*0766;HLA-B*0764;HLA-B*0761;HLA-B*0762;HLA-B*4210;HLA-B*4212;HLA-B*0777;HLA-B*0795;HLA-B*0796;HLA-B*0799;HLA-B*4213;HLA-B*0774;HLA-B*0780;HLA-B*0778;HLA-B*0784;HLA-B*0798;HLA-B*0782;HLA-B*0787;HLA-B*0790;HLA-B*0793;HLA-B*0789;HLA-B*8101;HLA-B*8102;HLA-B*8103 |
| 4U6Y | FLNKDLEVDGHFVTM | 2;15 | HLA-A*0201;HLA-A*0203;HLA-A*0204;HLA-A*0207;HLA-A*0209;HLA-A*0212;HLA-A*0213;HLA-A*0216;HLA-A*0217;HLA-A*0218;HLA-A*0219;HLA-A*0222;HLA-A*0224;HLA-A*0225;HLA-A*0226;HLA-A*0227;HLA-A*0233;HLA-A*0236;HLA-A*0237;HLA-A*0238;HLA-A*0239;HLA-A*0240;HLA-A*0249;HLA-A*0252;HLA-A*0266;HLA-A*0289;HLA-A*0271;HLA-A*0268;HLA-A*0274;HLA-A*0258;HLA-A*0280;HLA-A*0265;HLA-A*0286;HLA-A*0277;HLA-A*0260;HLA-A*0273;HLA-A*0285;HLA-A*0264;HLA-A*0275;HLA-A*0267;HLA-A*0295;HLA-A*0297;HLA-A*9201;HLA-A*0296;HLA-A*0293;HLA-A*9221;HLA-A*9231;HLA-A*9232;HLA-A*9214;HLA-A*9202;HLA-A*9210;HLA-A*9205;HLA-A*9217;HLA-A*9211;HLA-A*9204;HLA-A*9230;HLA-A*9234;HLA-A*9218;HLA-A*9247;HLA-A*9249;HLA-A*9251;HLA-A*9248;HLA-A*9256;HLA-A*9235;HLA-A*9252;HLA-A*9240;HLA-A*9253;HLA-A*9245;HLA-A*9277;HLA-A*9257;HLA-A*9271;HLA-A*9294;HLA-A*9264;HLA-A*9290;HLA-A*9266;HLA-A*9291;HLA-A*9289;HLA-A*9274;HLA-A*9262;HLA-A*9259;HLA-A*9263;HLA-A*9268;HLA-A*9288;HLA-A*9293;HLA-A*9287;HLA-A*9283;HLA-A*9267;HLA-A*9265;HLA-A*9282;HLA-A*9297;HLA-A*9298;HLA-A*9299;HLA-A*0201;HLA-A*0209;HLA-A*0266;HLA-A*0289;HLA-A*0275;HLA-A*0297;HLA-A*0296;HLA-A*9221;HLA-A*9232;HLA-A*9234;HLA-A*9240;HLA-A*9299 |
| 4WDI | LYLVCGERG | 2;9 | MH1-K1*02;MH1-K1*02 |
| 4WU5 | RYPLTFGW | 2;8 | HLA-A*2301;HLA-A*2302;HLA-A*2303;HLA-A*2304;HLA-A*2402;HLA-A*2403;HLA-A*2405;HLA-A*2406;HLA-A*2410;HLA-A*2413;HLA-A*2414;HLA-A*2415;HLA-A*2417;HLA-A*2418;HLA-A*2422;HLA-A*2423;HLA-A*2426;HLA-A*2427;HLA-A*2433;HLA-A*2454;HLA-A*2453;HLA-A*2443;HLA-A*2456;HLA-A*2439;HLA-A*2441;HLA-A*2452;HLA-A*2310;HLA-A*2455;HLA-A*2446;HLA-A*2457;HLA-A*2314;HLA-A*2451;HLA-A*2459;HLA-A*2464;HLA-A*2466;HLA-A*2462;HLA-A*2479;HLA-A*2470;HLA-A*2473;HLA-A*2476;HLA-A*2478;HLA-A*2472;HLA-A*2316;HLA-A*2468;HLA-A*2315;HLA-A*2475;HLA-A*2481;HLA-A*2471;HLA-A*2493;HLA-A*2494;HLA-A*2318;HLA-A*2319;HLA-A*2487;HLA-A*2317;HLA-A*2491;HLA-A*2496;HLA-A*2497;HLA-A*2492;HLA-A*2485;HLA-A*2498;HLA-A*2320;HLA-A*2499;HLA-A*2323;HLA-A*2325;HLA-A*2324;HLA-A*2402;HLA-A*2479;HLA-A*2476 |
| 4WU7 | RFPLTFGW | 2;8 | HLA-A*2301;HLA-A*2302;HLA-A*2303;HLA-A*2304;HLA-A*2402;HLA-A*2403;HLA-A*2405;HLA-A*2406;HLA-A*2410;HLA-A*2413;HLA-A*2414;HLA-A*2415;HLA-A*2417;HLA-A*2418;HLA-A*2422;HLA-A*2423;HLA-A*2426;HLA-A*2427;HLA-A*2433;HLA-A*2454;HLA-A*2453;HLA-A*2443;HLA-A*2456;HLA-A*2439;HLA-A*2441;HLA-A*2452;HLA-A*2310;HLA-A*2455;HLA-A*2446;HLA-A*2457;HLA-A*2314;HLA-A*2451;HLA-A*2459;HLA-A*2464;HLA-A*2466;HLA-A*2462;HLA-A*2479;HLA-A*2470;HLA-A*2473;HLA-A*2476;HLA-A*2478;HLA-A*2472;HLA-A*2316;HLA-A*2468;HLA-A*2315;HLA-A*2475;HLA-A*2481;HLA-A*2471;HLA-A*2493;HLA-A*2494;HLA-A*2318;HLA-A*2319;HLA-A*2487;HLA-A*2317;HLA-A*2491;HLA-A*2496;HLA-A*2497;HLA-A*2492;HLA-A*2485;HLA-A*2498;HLA-A*2320;HLA-A*2499;HLA-A*2323;HLA-A*2325;HLA-A*2324;HLA-A*2402;HLA-A*2479;HLA-A*2476 |
| 4WUU | RMFPNAPYL | 2;9 | HLA-A*0201;HLA-A*0203;HLA-A*0204;HLA-A*0207;HLA-A*0209;HLA-A*0212;HLA-A*0213;HLA-A*0216;HLA-A*0217;HLA-A*0218;HLA-A*0222;HLA-A*0224;HLA-A*0226;HLA-A*0233;HLA-A*0266;HLA-A*0289;HLA-A*0268;HLA-A*0265;HLA-A*0277;HLA-A*0260;HLA-A*9232;HLA-A*9234;HLA-A*9235;HLA-A*0201;HLA-A*0209;HLA-A*0266;HLA-A*0289;HLA-A*9232;HLA-A*9234 |
| 4XXC | DELEIKAY | 2;8 | HLA-B*1801;HLA-B*1802;HLA-B*1805;HLA-B*1808;HLA-B*1810;HLA-B*1811;HLA-B*1813;HLA-B*1814;HLA-B*1815;HLA-B*1819;HLA-B*1822;HLA-B*1818;HLA-B*1820;HLA-B*1821;HLA-B*1826;HLA-B*1827;HLA-B*1832;HLA-B*1831;HLA-B*1830;HLA-B*1828;HLA-B*1835;HLA-B*1839;HLA-B*1840;HLA-B*1841;HLA-B*1836;HLA-B*1838;HLA-B*1844;HLA-B*1834;HLA-B*1846;HLA-B*1801 |
| 4Z76 | LYLVCGERV | 2;9 | MH1-K1*02;MH1-K1*02 |
| 4Z77 | LYLVCGERV | 2;9 | MH1-K1*02;MH1-K1*02 |
| 4Z78 | LYLVCGERGF | 2;10 | MH1-K1*02;MH1-K1*02 |
| 4ZEZ | KLVALVINAV | 2;10 | HLA-A*0201;HLA-A*0203;HLA-A*0204;HLA-A*0207;HLA-A*0209;HLA-A*0212;HLA-A*0213;HLA-A*0216;HLA-A*0217;HLA-A*0218;HLA-A*0222;HLA-A*0224;HLA-A*0226;HLA-A*0233;HLA-A*0266;HLA-A*0289;HLA-A*0268;HLA-A*0265;HLA-A*0277;HLA-A*0260;HLA-A*9232;HLA-A*9234;HLA-A*9235;HLA-A*0201;HLA-A*0209;HLA-A*0266;HLA-A*0289;HLA-A*9232;HLA-A*9234 |
| 4ZUS | QAEVLQERLEW | 2;11 | MH1-N*00602;MH1-N*00602 |
| 4ZUT | GSQKLTTGNCNW | 2;12 | MH1-N*00602;MH1-N*00602 |
| 4ZUU | CTSEEMNAF | 2;9 | MH1-N*00602;MH1-N*00602 |
| 4ZUV | RVEDVTNTAEYW | 2;12 | MH1-N*00602;MH1-N*00602 |
| 4ZUW | GSQKLTTGNCNW | 2;12 | MH1-N*00602;MH1-N*00602 |
| 5B38 | LSSPVTKSF | 2;9 | HLA-B*5701;HLA-B*5701 |
| 5B39 | LSSPVTKSF | 2;9 | HLA-B*5701;HLA-B*5701 |
| 5BRZ | EVDPIGHLY | 2;9 | HLA-A*0101;HLA-A*0103;HLA-A*3601;HLA-A*3603;HLA-A*0114;HLA-A*0101 |
| 5BS0 | ESDPIVAQY | 2;9 | HLA-A*0101;HLA-A*0103;HLA-A*3601;HLA-A*3603;HLA-A*0114;HLA-A*0101 |
| 5C07 | YQFGPDFPIA | 2;10 | HLA-A*0201;HLA-A*0201 |
| 5C08 | RQWGPDPAAV | 2;10 | HLA-A*0201;HLA-A*0201 |
| 5C09 | YLGGPDFPTI | 2;10 | HLA-A*0201;HLA-A*0201 |
| 5C0A | MVWGPDPLYV | 2;10 | HLA-A*0201;HLA-A*0201 |
| 5C0B | RQFGPDFPTI | 2;10 | HLA-A*0201;HLA-A*0201 |
| 5C0C | RQFGPDWIVA | 2;10 | HLA-A*0201;HLA-A*0201 |
| 5C0D | AQWGPDPAAA | 2;10 | HLA-A*0201;HLA-A*0201 |
| 5C0E | YQFGPDFPIA | 2;10 | HLA-A*0201;HLA-A*0201 |
| 5C0F | RQWGPDPAAV | 2;10 | HLA-A*0201;HLA-A*0201 |
| 5C0G | YLGGPDFPTI | 2;10 | HLA-A*0201;HLA-A*0201 |
| 5C0H | YLGGPDFPTI | 2;10 | HLA-A*0201;HLA-A*0201 |
| 5C0I | RQFGPDFPTI | 2;10 | HLA-A*0201;HLA-A*0201 |
| 5C0J | RQFGPDWIVA | 2;10 | HLA-A*0201;HLA-A*0201 |
| 5CNZ | FANFCLMMI | 2;9 | MH1-A*01;MH1-A*01 |
| 5D2L | NLVPMVATV | 2;9 | HLA-A*0201;HLA-A*0203;HLA-A*0204;HLA-A*0207;HLA-A*0209;HLA-A*0212;HLA-A*0213;HLA-A*0216;HLA-A*0217;HLA-A*0218;HLA-A*0222;HLA-A*0224;HLA-A*0226;HLA-A*0233;HLA-A*0266;HLA-A*0289;HLA-A*0268;HLA-A*0265;HLA-A*0277;HLA-A*0260;HLA-A*9232;HLA-A*9234;HLA-A*9235;HLA-A*0201;HLA-A*0209;HLA-A*0266;HLA-A*0289;HLA-A*9232;HLA-A*9234 |
| 5D2N | NLVPMVATV | 2;9 | HLA-A*0201;HLA-A*0203;HLA-A*0204;HLA-A*0207;HLA-A*0209;HLA-A*0212;HLA-A*0213;HLA-A*0216;HLA-A*0217;HLA-A*0218;HLA-A*0222;HLA-A*0224;HLA-A*0226;HLA-A*0233;HLA-A*0266;HLA-A*0289;HLA-A*0268;HLA-A*0265;HLA-A*0277;HLA-A*0260;HLA-A*9232;HLA-A*9234;HLA-A*9235;HLA-A*0201;HLA-A*0209;HLA-A*0266;HLA-A*0289;HLA-A*9232;HLA-A*9234 |
| 5D9S | FVLELEPEWTV | 2;11 | HLA-A*0201;HLA-A*0201 |
| 5DDH | FVLELEPEWTVK | 2;11 | HLA-A*0201;HLA-A*0201 |
| 5DEG | RRKWRRWHL | 2;9 | HLA-B*2706;HLA-B*2706 |
| 5E00 | GVWIRTPPA | 2;9 | HLA-A*0201;HLA-A*0203;HLA-A*0204;HLA-A*0207;HLA-A*0209;HLA-A*0212;HLA-A*0213;HLA-A*0216;HLA-A*0217;HLA-A*0218;HLA-A*0222;HLA-A*0224;HLA-A*0226;HLA-A*0233;HLA-A*0266;HLA-A*0289;HLA-A*0268;HLA-A*0265;HLA-A*0277;HLA-A*0260;HLA-A*9232;HLA-A*9234;HLA-A*9235;HLA-A*0201;HLA-A*0209;HLA-A*0266;HLA-A*0289;HLA-A*9232;HLA-A*9234 |
| 5E8N | MCLRMTAVM | 2;9 | H2-D1b;H2-D1b |
| 5E9D | ELAGIGILTV | 2;10 | HLA-A*0201;HLA-A*0203;HLA-A*0204;HLA-A*0207;HLA-A*0209;HLA-A*0212;HLA-A*0213;HLA-A*0216;HLA-A*0217;HLA-A*0218;HLA-A*0222;HLA-A*0224;HLA-A*0226;HLA-A*0233;HLA-A*0266;HLA-A*0289;HLA-A*0268;HLA-A*0265;HLA-A*0277;HLA-A*0260;HLA-A*9232;HLA-A*9234;HLA-A*9235;HLA-A*0201;HLA-A*0209;HLA-A*0266;HLA-A*0289;HLA-A*9232;HLA-A*9234 |
| 5ENW | GLKEGIPAL | 2;9 | HLA-A*0201;HLA-A*0203;HLA-A*0204;HLA-A*0207;HLA-A*0209;HLA-A*0212;HLA-A*0213;HLA-A*0216;HLA-A*0217;HLA-A*0218;HLA-A*0222;HLA-A*0224;HLA-A*0226;HLA-A*0233;HLA-A*0266;HLA-A*0289;HLA-A*0268;HLA-A*0265;HLA-A*0277;HLA-A*0260;HLA-A*9232;HLA-A*9234;HLA-A*9235;HLA-A*0201;HLA-A*0209;HLA-A*0266;HLA-A*0289;HLA-A*9232;HLA-A*9234 |
| 5EO0 | RPMTFKGAL | 2;9 | HLA-B*0702;HLA-B*0702 |
| 5EO1 | RPMTYKGAL | 2;9 | HLA-B*0702;HLA-B*0702 |
| 5EOT | GLLPELPAVGG | 2;9 | HLA-A*0201;HLA-A*0203;HLA-A*0204;HLA-A*0207;HLA-A*0209;HLA-A*0212;HLA-A*0213;HLA-A*0216;HLA-A*0217;HLA-A*0218;HLA-A*0219;HLA-A*0222;HLA-A*0224;HLA-A*0225;HLA-A*0226;HLA-A*0227;HLA-A*0233;HLA-A*0236;HLA-A*0237;HLA-A*0238;HLA-A*0239;HLA-A*0240;HLA-A*0249;HLA-A*0252;HLA-A*0266;HLA-A*0289;HLA-A*0271;HLA-A*0268;HLA-A*0274;HLA-A*0258;HLA-A*0280;HLA-A*0265;HLA-A*0286;HLA-A*0277;HLA-A*0260;HLA-A*0273;HLA-A*0285;HLA-A*0264;HLA-A*0275;HLA-A*0267;HLA-A*0295;HLA-A*0297;HLA-A*9201;HLA-A*0296;HLA-A*0293;HLA-A*9221;HLA-A*9231;HLA-A*9232;HLA-A*9214;HLA-A*9202;HLA-A*9210;HLA-A*9205;HLA-A*9217;HLA-A*9211;HLA-A*9204;HLA-A*9230;HLA-A*9234;HLA-A*9218;HLA-A*9247;HLA-A*9249;HLA-A*9251;HLA-A*9248;HLA-A*9256;HLA-A*9235;HLA-A*9252;HLA-A*9240;HLA-A*9253;HLA-A*9245;HLA-A*9277;HLA-A*9257;HLA-A*9271;HLA-A*9294;HLA-A*9264;HLA-A*9290;HLA-A*9266;HLA-A*9291;HLA-A*9289;HLA-A*9274;HLA-A*9262;HLA-A*9259;HLA-A*9263;HLA-A*9268;HLA-A*9288;HLA-A*9293;HLA-A*9287;HLA-A*9283;HLA-A*9267;HLA-A*9265;HLA-A*9282;HLA-A*9297;HLA-A*9298;HLA-A*0201;HLA-A*0209;HLA-A*0266;HLA-A*0289;HLA-A*0275;HLA-A*0297;HLA-A*9232;HLA-A*9234;HLA-A*9240 |
| 5EU3 | YLEPGPVTA | 2;9 | HLA-A*0201;HLA-A*0203;HLA-A*0204;HLA-A*0207;HLA-A*0209;HLA-A*0212;HLA-A*0213;HLA-A*0216;HLA-A*0217;HLA-A*0218;HLA-A*0222;HLA-A*0224;HLA-A*0226;HLA-A*0233;HLA-A*0266;HLA-A*0289;HLA-A*0268;HLA-A*0265;HLA-A*0277;HLA-A*0260;HLA-A*9232;HLA-A*9234;HLA-A*9235;HLA-A*0201;HLA-A*0209;HLA-A*0266;HLA-A*0289;HLA-A*9232;HLA-A*9234 |
| 5EU4 | YLAPGPVTA | 2;9 | HLA-A*0201;HLA-A*0201 |
| 5EU5 | YLEPAPVTA | 2;9 | HLA-A*0201;HLA-A*0201 |
| 5EU6 | YLEPGPVTV | 2;9 | HLA-A*0201;HLA-A*0201 |
| 5EUO | GILGFVFTL | 2;9 | HLA-A*0201;HLA-A*0203;HLA-A*0204;HLA-A*0207;HLA-A*0209;HLA-A*0212;HLA-A*0213;HLA-A*0216;HLA-A*0217;HLA-A*0218;HLA-A*0222;HLA-A*0224;HLA-A*0226;HLA-A*0233;HLA-A*0266;HLA-A*0289;HLA-A*0268;HLA-A*0265;HLA-A*0277;HLA-A*0260;HLA-A*9232;HLA-A*9234;HLA-A*9235;HLA-A*0201;HLA-A*0209;HLA-A*0266;HLA-A*0289;HLA-A*9232;HLA-A*9234 |
| 5F1I | KLFSGELTK | 2;9 | HLA-A*0205;HLA-A*0206;HLA-A*0210;HLA-A*0214;HLA-A*0251;HLA-A*0257;HLA-A*0279 |
| 5F7D | GLKEGIPALD | 2;9 | HLA-A*0201;HLA-A*0203;HLA-A*0204;HLA-A*0207;HLA-A*0209;HLA-A*0212;HLA-A*0213;HLA-A*0216;HLA-A*0217;HLA-A*0218;HLA-A*0219;HLA-A*0222;HLA-A*0224;HLA-A*0225;HLA-A*0226;HLA-A*0227;HLA-A*0233;HLA-A*0236;HLA-A*0237;HLA-A*0238;HLA-A*0239;HLA-A*0240;HLA-A*0249;HLA-A*0252;HLA-A*0266;HLA-A*0289;HLA-A*0271;HLA-A*0268;HLA-A*0274;HLA-A*0258;HLA-A*0280;HLA-A*0265;HLA-A*0286;HLA-A*0277;HLA-A*0260;HLA-A*0273;HLA-A*0285;HLA-A*0264;HLA-A*0275;HLA-A*0267;HLA-A*0295;HLA-A*0297;HLA-A*9201;HLA-A*0296;HLA-A*0293;HLA-A*9221;HLA-A*9231;HLA-A*9232;HLA-A*9214;HLA-A*9202;HLA-A*9210;HLA-A*9205;HLA-A*9217;HLA-A*9211;HLA-A*9204;HLA-A*9230;HLA-A*9234;HLA-A*9218;HLA-A*9247;HLA-A*9249;HLA-A*9251;HLA-A*9248;HLA-A*9256;HLA-A*9235;HLA-A*9252;HLA-A*9240;HLA-A*9253;HLA-A*9245;HLA-A*9277;HLA-A*9257;HLA-A*9271;HLA-A*9294;HLA-A*9264;HLA-A*9290;HLA-A*9266;HLA-A*9291;HLA-A*9289;HLA-A*9274;HLA-A*9262;HLA-A*9259;HLA-A*9263;HLA-A*9268;HLA-A*9288;HLA-A*9293;HLA-A*9287;HLA-A*9283;HLA-A*9267;HLA-A*9265;HLA-A*9282;HLA-A*9297;HLA-A*9298;HLA-A*0201;HLA-A*0209;HLA-A*0266;HLA-A*0289;HLA-A*0275;HLA-A*0297;HLA-A*9232;HLA-A*9234;HLA-A*9240 |
| 5F9J | YLSPIASPL | 2;9 | HLA-A*0201;HLA-A*0203;HLA-A*0204;HLA-A*0207;HLA-A*0209;HLA-A*0212;HLA-A*0213;HLA-A*0216;HLA-A*0217;HLA-A*0218;HLA-A*0222;HLA-A*0224;HLA-A*0226;HLA-A*0233;HLA-A*0266;HLA-A*0289;HLA-A*0268;HLA-A*0265;HLA-A*0277;HLA-A*0260;HLA-A*9232;HLA-A*9234;HLA-A*9235;HLA-A*0201;HLA-A*0209;HLA-A*0266;HLA-A*0289;HLA-A*9232;HLA-A*9234 |
| 5FA3 | GLLPELPAV | 2;9 | HLA-A*0201;HLA-A*0203;HLA-A*0204;HLA-A*0207;HLA-A*0209;HLA-A*0212;HLA-A*0213;HLA-A*0216;HLA-A*0217;HLA-A*0218;HLA-A*0219;HLA-A*0222;HLA-A*0224;HLA-A*0225;HLA-A*0226;HLA-A*0227;HLA-A*0233;HLA-A*0236;HLA-A*0237;HLA-A*0238;HLA-A*0239;HLA-A*0240;HLA-A*0249;HLA-A*0252;HLA-A*0266;HLA-A*0289;HLA-A*0271;HLA-A*0268;HLA-A*0274;HLA-A*0258;HLA-A*0280;HLA-A*0265;HLA-A*0286;HLA-A*0277;HLA-A*0260;HLA-A*0273;HLA-A*0285;HLA-A*0264;HLA-A*0275;HLA-A*0267;HLA-A*0295;HLA-A*0297;HLA-A*9201;HLA-A*0296;HLA-A*0293;HLA-A*9221;HLA-A*9231;HLA-A*9232;HLA-A*9214;HLA-A*9202;HLA-A*9210;HLA-A*9205;HLA-A*9217;HLA-A*9211;HLA-A*9204;HLA-A*9230;HLA-A*9234;HLA-A*9218;HLA-A*9247;HLA-A*9249;HLA-A*9251;HLA-A*9248;HLA-A*9256;HLA-A*9235;HLA-A*9252;HLA-A*9240;HLA-A*9253;HLA-A*9245;HLA-A*9277;HLA-A*9257;HLA-A*9271;HLA-A*9294;HLA-A*9264;HLA-A*9290;HLA-A*9266;HLA-A*9291;HLA-A*9289;HLA-A*9274;HLA-A*9262;HLA-A*9259;HLA-A*9263;HLA-A*9268;HLA-A*9288;HLA-A*9293;HLA-A*9287;HLA-A*9283;HLA-A*9267;HLA-A*9265;HLA-A*9282;HLA-A*9297;HLA-A*9298;HLA-A*0201;HLA-A*0209;HLA-A*0266;HLA-A*0289;HLA-A*0275;HLA-A*0297;HLA-A*9232;HLA-A*9234;HLA-A*9240 |
| 5FA4 | YLSPIASPLLD | 2;9 | HLA-A*0201;HLA-A*0203;HLA-A*0204;HLA-A*0207;HLA-A*0209;HLA-A*0212;HLA-A*0213;HLA-A*0216;HLA-A*0217;HLA-A*0218;HLA-A*0222;HLA-A*0224;HLA-A*0226;HLA-A*0233;HLA-A*0266;HLA-A*0289;HLA-A*0268;HLA-A*0265;HLA-A*0277;HLA-A*0260;HLA-A*9232;HLA-A*9234;HLA-A*9235;HLA-A*0201;HLA-A*0209;HLA-A*0266;HLA-A*0289;HLA-A*9232;HLA-A*9234 |
| 5FDW | YLSPIASPLL | 2;10 | HLA-A*0201;HLA-A*0203;HLA-A*0204;HLA-A*0207;HLA-A*0209;HLA-A*0212;HLA-A*0213;HLA-A*0216;HLA-A*0217;HLA-A*0218;HLA-A*0222;HLA-A*0224;HLA-A*0226;HLA-A*0233;HLA-A*0266;HLA-A*0289;HLA-A*0268;HLA-A*0265;HLA-A*0277;HLA-A*0260;HLA-A*9232;HLA-A*9234;HLA-A*9235;HLA-A*0201;HLA-A*0209;HLA-A*0266;HLA-A*0289;HLA-A*9232;HLA-A*9234 |
| 5GR7 | YYSIIPHSI | 2;9 | HLA-C*1203;HLA-C*1604;HLA-C*1202;HLA-C*1206;HLA-C*1208;HLA-C*1601;HLA-C*0813;HLA-C*1606;HLA-C*1214;HLA-C*1215;HLA-C*0318;HLA-C*1210;HLA-C*1211;HLA-C*1607;HLA-C*1213;HLA-C*1219;HLA-C*1218 |
| 5GRD | SSCSSCPLSK | 2;10 | HLA-A*1101;HLA-A*1103;HLA-A*1104;HLA-A*1105;HLA-A*1107;HLA-A*1125;HLA-A*1101 |
| 5GSB | YYSIIPHSI | 2;9 | MH1-K1*02;MH1-K1*02 |
| 5GSD | SSCPLSK | 1;7 | HLA-A*1101;HLA-A*1103;HLA-A*1104;HLA-A*1105;HLA-A*1107;HLA-A*1125;HLA-A*1101 |
| 5GSR | YYSIAPHSI | 2;9 | MH1-K1*02;MH1-K1*02 |
| 5GSX | FYAPEPITSL | 2;10 | HLA-C*1203;HLA-C*1604;HLA-C*1202;HLA-C*1206;HLA-C*1208;HLA-C*1601;HLA-C*0813;HLA-C*1606;HLA-C*1214;HLA-C*1215;HLA-C*0318;HLA-C*1210;HLA-C*1211;HLA-C*1607;HLA-C*1213;HLA-C*1219;HLA-C*1218 |
| 5H5Z | FANFCLMMI | 2;9 | MH1-A*01;MH1-A*01 |
| 5H94 | KMNTQFTAV | 2;9 | MH1*0401;MH1*0401 |
| 5HGA | RFPLTFGW | 2;8 | HLA-A*2402;HLA-A*2402 |
| 5HGD | RFPLTFGWCF | 2;10 | HLA-A*2402;HLA-A*2402 |
| 5HGH | RYPLTFGWCF | 2;10 | HLA-A*2402;HLA-A*2402 |
| 5HHM | GILGLVFTL | 2;9 | HLA-A*0201;HLA-A*0201 |
| 5HHN | GILGLVFTL | 2;9 | HLA-A*0201;HLA-A*0201 |
| 5HHO | GILEFVFTL | 2;9 | HLA-A*0201;HLA-A*0201 |
| 5HHP | GILEFVFTL | 2;9 | HLA-A*0201;HLA-A*0201 |
| 5HHQ | GIWGFVFTL | 2;9 | HLA-A*0201;HLA-A*0201 |
| 5HYJ | AQWGPDPAAA | 2;10 | HLA-A*0201;HLA-A*0201 |
| 5IB1 | RRKWRRWHL | 2;9 | HLA-B*2705;HLA-B*2707;HLA-B*2709;HLA-B*2713;HLA-B*2714;HLA-B*2732;HLA-B*2735;HLA-B*2738;HLA-B*2747;HLA-B*2705;HLA-B*2713 |
| 5IB2 | RRKWRRWHL | 2;9 | HLA-B*2705;HLA-B*2707;HLA-B*2709;HLA-B*2713;HLA-B*2714;HLA-B*2732;HLA-B*2735;HLA-B*2738;HLA-B*2747;HLA-B*2705;HLA-B*2713 |
| 5IB3 | RRKWRRWHL | 2;9 | HLA-B*2705;HLA-B*2707;HLA-B*2709;HLA-B*2713;HLA-B*2714;HLA-B*2732;HLA-B*2735;HLA-B*2738;HLA-B*2747;HLA-B*2705;HLA-B*2713 |
| 5IB4 | RRKWRRWHL | 2;9 | HLA-B*2705;HLA-B*2707;HLA-B*2709;HLA-B*2713;HLA-B*2714;HLA-B*2732;HLA-B*2735;HLA-B*2738;HLA-B*2747;HLA-B*2705;HLA-B*2713 |
| 5IB5 | RRKWRRWHL | 2;9 | HLA-B*2705;HLA-B*2707;HLA-B*2709;HLA-B*2713;HLA-B*2714;HLA-B*2732;HLA-B*2735;HLA-B*2738;HLA-B*2747;HLA-B*2709 |
| 5IEH | REFSKEPEL | 2;9 | HLA-B*4002;HLA-B*4003;HLA-B*4004;HLA-B*4005;HLA-B*4006;HLA-B*4009;HLA-B*4018;HLA-B*4020;HLA-B*4024;HLA-B*4026;HLA-B*4027;HLA-B*4028;HLA-B*4035;HLA-B*4039;HLA-B*4056;HLA-B*4044;HLA-B*4064;HLA-B*4074;HLA-B*4071;HLA-B*4070;HLA-B*4078;HLA-B*4082;HLA-B*4086;HLA-B*4085;HLA-B*4091;HLA-B*4089;HLA-B*4090;HLA-B*4097;HLA-B*4095;HLA-B*4098;HLA-B*4099;HLA-B*4002;HLA-B*4056;HLA-B*4097 |
| 5IEK | REFSKEPEL | 2;9 | HLA-B*4002;HLA-B*4003;HLA-B*4004;HLA-B*4005;HLA-B*4006;HLA-B*4020;HLA-B*4039;HLA-B*4070;HLA-B*4089;HLA-B*4097;HLA-B*4002;HLA-B*4097 |
| 5IM7 | QASQEVKNW | 2;9 | HLA-C*1510 |
| 5INC | QATQEVANW | 2;9 | HLA-C*1510 |
| 5IND | QASQDVKNW | 2;9 | HLA-C*1510 |
| 5IRO | LLFGYPVYV | 2;9 | HLA-A*0201;HLA-A*0201 |
| 5ISZ | GILGFVFTL | 2;9 | HLA-A*0201;HLA-A*0203;HLA-A*0204;HLA-A*0207;HLA-A*0209;HLA-A*0212;HLA-A*0213;HLA-A*0216;HLA-A*0217;HLA-A*0218;HLA-A*0222;HLA-A*0224;HLA-A*0226;HLA-A*0233;HLA-A*0266;HLA-A*0289;HLA-A*0268;HLA-A*0265;HLA-A*0277;HLA-A*0260;HLA-A*9232;HLA-A*9234;HLA-A*9235;HLA-A*0201;HLA-A*0209;HLA-A*0266;HLA-A*0289;HLA-A*9232;HLA-A*9234 |
| 5IUE | ILRWEQD | 3;6 | HLA-F*0101;HLA-F*0102;HLA-F*0103;HLA-F*0101;HLA-F*0102;HLA-F*0103 |
| 5IVX | RGPGRAFVTI | 2;10 | H2-D1*02;H2-D1*02 |
| 5J6G | VGITNVDL | 2;8 | H2-Q10*02;H2-Q10*02 |
| 5J6H | VGITNVDL | 2;8 | H2-Q10*02;H2-Q10*02 |
| 5JHD | GILGFVFTL | 2;9 | HLA-A*0201;HLA-A*0203;HLA-A*0204;HLA-A*0207;HLA-A*0209;HLA-A*0212;HLA-A*0213;HLA-A*0216;HLA-A*0217;HLA-A*0218;HLA-A*0222;HLA-A*0224;HLA-A*0226;HLA-A*0233;HLA-A*0266;HLA-A*0289;HLA-A*0268;HLA-A*0265;HLA-A*0277;HLA-A*0260;HLA-A*9232;HLA-A*9234;HLA-A*9235;HLA-A*0201;HLA-A*0209;HLA-A*0266;HLA-A*0289;HLA-A*9232;HLA-A*9234 |
| 5JWD | WLVTNGSYL | 2;9 | H2-D1b;H2-D1b |
| 5JWE | CSANNSHHYI | 2;10 | H2-D1b;H2-D1b |
| 5JZI | KLVALGINAV | 2;10 | HLA-A*0201;HLA-A*0203;HLA-A*0204;HLA-A*0207;HLA-A*0209;HLA-A*0212;HLA-A*0213;HLA-A*0216;HLA-A*0217;HLA-A*0218;HLA-A*0222;HLA-A*0224;HLA-A*0226;HLA-A*0233;HLA-A*0266;HLA-A*0289;HLA-A*0268;HLA-A*0265;HLA-A*0277;HLA-A*0260;HLA-A*9232;HLA-A*9234;HLA-A*9235;HLA-A*0201;HLA-A*0209;HLA-A*0266;HLA-A*0289;HLA-A*9232;HLA-A*9234 |
| 5KD4 | IGPGRAFYVI | 2;10 | H2-D1*02;H2-D1*02 |
| 5KD7 | IGPGRAFYV | 2;9 | H2-D1*02;H2-D1*02 |
| 5M00 | KAVANFATM | 2;9 | H2-D1b;H2-D1b |
| 5M01 | KAPANFATM | 2;9 | H2-D1b;H2-D1b |
| 5M02 | KAPFNFATM | 2;9 | H2-D1b;H2-D1b |
| 5MEO | ILGKFLHRL | 2;9 | HLA-A*0201;HLA-A*0203;HLA-A*0204;HLA-A*0207;HLA-A*0209;HLA-A*0212;HLA-A*0213;HLA-A*0216;HLA-A*0217;HLA-A*0218;HLA-A*0222;HLA-A*0224;HLA-A*0226;HLA-A*0233;HLA-A*0266;HLA-A*0289;HLA-A*0268;HLA-A*0265;HLA-A*0277;HLA-A*0260;HLA-A*9232;HLA-A*9234;HLA-A*9235;HLA-A*0201;HLA-A*0209;HLA-A*0266;HLA-A*0289;HLA-A*9232;HLA-A*9234 |
| 5MEP | ILGKFLHWL | 2;9 | HLA-A*0201;HLA-A*0203;HLA-A*0204;HLA-A*0207;HLA-A*0209;HLA-A*0212;HLA-A*0213;HLA-A*0216;HLA-A*0217;HLA-A*0218;HLA-A*0222;HLA-A*0224;HLA-A*0226;HLA-A*0233;HLA-A*0266;HLA-A*0289;HLA-A*0268;HLA-A*0265;HLA-A*0277;HLA-A*0260;HLA-A*9232;HLA-A*9234;HLA-A*9235;HLA-A*0201;HLA-A*0209;HLA-A*0266;HLA-A*0289;HLA-A*9232;HLA-A*9234 |
| 5MER | ILAKFLHEL | 2;9 | HLA-A*0201;HLA-A*0203;HLA-A*0204;HLA-A*0207;HLA-A*0209;HLA-A*0212;HLA-A*0213;HLA-A*0216;HLA-A*0217;HLA-A*0218;HLA-A*0222;HLA-A*0224;HLA-A*0226;HLA-A*0233;HLA-A*0266;HLA-A*0289;HLA-A*0268;HLA-A*0265;HLA-A*0277;HLA-A*0260;HLA-A*9232;HLA-A*9234;HLA-A*9235;HLA-A*0201;HLA-A*0209;HLA-A*0266;HLA-A*0289;HLA-A*9232;HLA-A*9234 |
| 5MZM | MCPRMTAVM | 2;9 | H2-D1b;H2-D1b |
| 5N1Y | MVWGPDPLYV | 2;10 | HLA-A*0201;HLA-A*0203;HLA-A*0204;HLA-A*0207;HLA-A*0209;HLA-A*0212;HLA-A*0213;HLA-A*0216;HLA-A*0217;HLA-A*0218;HLA-A*0222;HLA-A*0224;HLA-A*0226;HLA-A*0233;HLA-A*0266;HLA-A*0289;HLA-A*0268;HLA-A*0265;HLA-A*0277;HLA-A*0260;HLA-A*9232;HLA-A*9234;HLA-A*9235;HLA-A*0201;HLA-A*0209;HLA-A*0266;HLA-A*0289;HLA-A*9232;HLA-A*9234 |
| 5N6B | LLWNGPMAV | 2;9 | HLA-A*0201;HLA-A*0203;HLA-A*0204;HLA-A*0207;HLA-A*0209;HLA-A*0212;HLA-A*0213;HLA-A*0216;HLA-A*0217;HLA-A*0218;HLA-A*0222;HLA-A*0224;HLA-A*0226;HLA-A*0233;HLA-A*0266;HLA-A*0289;HLA-A*0268;HLA-A*0265;HLA-A*0277;HLA-A*0260;HLA-A*9232;HLA-A*9234;HLA-A*9235;HLA-A*0201;HLA-A*0209;HLA-A*0266;HLA-A*0289;HLA-A*9232;HLA-A*9234 |
| 5NHT | ELAGIGILTV | 2;10 | HLA-A*0201;HLA-A*0203;HLA-A*0204;HLA-A*0207;HLA-A*0209;HLA-A*0212;HLA-A*0213;HLA-A*0216;HLA-A*0217;HLA-A*0218;HLA-A*0222;HLA-A*0224;HLA-A*0226;HLA-A*0233;HLA-A*0266;HLA-A*0289;HLA-A*0268;HLA-A*0265;HLA-A*0277;HLA-A*0260;HLA-A*9232;HLA-A*9234;HLA-A*9235;HLA-A*0201;HLA-A*0209;HLA-A*0266;HLA-A*0289;HLA-A*9232;HLA-A*9234 |
| 5NME | SLYNTVATL | 2;9 | HLA-A*0201;HLA-A*0203;HLA-A*0204;HLA-A*0207;HLA-A*0209;HLA-A*0212;HLA-A*0213;HLA-A*0216;HLA-A*0217;HLA-A*0218;HLA-A*0222;HLA-A*0224;HLA-A*0226;HLA-A*0233;HLA-A*0266;HLA-A*0289;HLA-A*0268;HLA-A*0265;HLA-A*0277;HLA-A*0260;HLA-A*9232;HLA-A*9234;HLA-A*9235;HLA-A*0201;HLA-A*0209;HLA-A*0266;HLA-A*0289;HLA-A*9232;HLA-A*9234 |
| 5NMF | SLYNTIATL | 2;9 | HLA-A*0201;HLA-A*0203;HLA-A*0204;HLA-A*0207;HLA-A*0209;HLA-A*0212;HLA-A*0213;HLA-A*0216;HLA-A*0217;HLA-A*0218;HLA-A*0222;HLA-A*0224;HLA-A*0226;HLA-A*0233;HLA-A*0266;HLA-A*0289;HLA-A*0268;HLA-A*0265;HLA-A*0277;HLA-A*0260;HLA-A*9232;HLA-A*9234;HLA-A*9235;HLA-A*0201;HLA-A*0209;HLA-A*0266;HLA-A*0289;HLA-A*9232;HLA-A*9234 |
| 5NMG | SLFNTIAVL | 2;9 | HLA-A*0201;HLA-A*0203;HLA-A*0204;HLA-A*0207;HLA-A*0209;HLA-A*0212;HLA-A*0213;HLA-A*0216;HLA-A*0217;HLA-A*0218;HLA-A*0222;HLA-A*0224;HLA-A*0226;HLA-A*0233;HLA-A*0266;HLA-A*0289;HLA-A*0268;HLA-A*0265;HLA-A*0277;HLA-A*0260;HLA-A*9232;HLA-A*9234;HLA-A*9235;HLA-A*0201;HLA-A*0209;HLA-A*0266;HLA-A*0289;HLA-A*9232;HLA-A*9234 |
| 5NMH | SLYNTIATL | 2;9 | HLA-A*0201;HLA-A*0203;HLA-A*0204;HLA-A*0207;HLA-A*0209;HLA-A*0212;HLA-A*0213;HLA-A*0216;HLA-A*0217;HLA-A*0218;HLA-A*0222;HLA-A*0224;HLA-A*0226;HLA-A*0233;HLA-A*0266;HLA-A*0289;HLA-A*0268;HLA-A*0265;HLA-A*0277;HLA-A*0260;HLA-A*9232;HLA-A*9234;HLA-A*9235;HLA-A*0201;HLA-A*0209;HLA-A*0266;HLA-A*0289;HLA-A*9232;HLA-A*9234 |
| 5NMK | SLFNTIAVL | 2;9 | HLA-A*0201;HLA-A*0203;HLA-A*0204;HLA-A*0207;HLA-A*0209;HLA-A*0212;HLA-A*0213;HLA-A*0216;HLA-A*0217;HLA-A*0218;HLA-A*0222;HLA-A*0224;HLA-A*0226;HLA-A*0233;HLA-A*0266;HLA-A*0289;HLA-A*0268;HLA-A*0265;HLA-A*0277;HLA-A*0260;HLA-A*9232;HLA-A*9234;HLA-A*9235;HLA-A*0201;HLA-A*0209;HLA-A*0266;HLA-A*0289;HLA-A*9232;HLA-A*9234 |
| 5NPZ | EFEDLTFLA | 2;9 | MH1*0401;MH1*0401 |
| 5NQ0 | DFEREGYSL | 2;9 | MH1*0401;MH1*0401 |
| 5NQ1 | DFEREGYSL | 2;9 | MH1*0401;MH1*0401 |
| 5NQ2 | IAYERMCNI | 2;9 | MH1*0401;MH1*0401 |
| 5NQ3 | EFEDLTFLA | 2;9 | MH1*0401;MH1*0401 |
| 5NQK | ELAGIGILTV | 2;10 | HLA-A*0201;HLA-A*0203;HLA-A*0204;HLA-A*0207;HLA-A*0209;HLA-A*0212;HLA-A*0213;HLA-A*0216;HLA-A*0217;HLA-A*0218;HLA-A*0222;HLA-A*0224;HLA-A*0226;HLA-A*0233;HLA-A*0266;HLA-A*0289;HLA-A*0268;HLA-A*0265;HLA-A*0277;HLA-A*0260;HLA-A*9232;HLA-A*9234;HLA-A*9235;HLA-A*0201;HLA-A*0209;HLA-A*0266;HLA-A*0289;HLA-A*9232;HLA-A*9234 |
| 5SWQ | CVNGSCFTV | 2;9 | HLA-A*0201;HLA-A*0203;HLA-A*0204;HLA-A*0207;HLA-A*0209;HLA-A*0212;HLA-A*0213;HLA-A*0216;HLA-A*0217;HLA-A*0218;HLA-A*0222;HLA-A*0224;HLA-A*0226;HLA-A*0233;HLA-A*0266;HLA-A*0289;HLA-A*0268;HLA-A*0265;HLA-A*0277;HLA-A*0260;HLA-A*9232;HLA-A*9234;HLA-A*9235;HLA-A*0201;HLA-A*0209;HLA-A*0266;HLA-A*0289;HLA-A*9232;HLA-A*9234 |
| 5SWS | ASNENMETM | 2;9 | H2-D1b;H2-D1b |
| 5SWZ | ASNENMETM | 2;9 | H2-D1b;H2-D1b |
| 5T6W | SSTRGISQLW | 3;10 | HLA-C*1510 |
| 5T6X | TSTTSVASSW | 3;10 | HLA-C*1510 |
| 5T6Z | TSTLQEQIGW | 3;10 | HLA-C*1510 |
| 5T70 | TSNLQEQIGW | 2;10 | HLA-C*1510 |
| 5T7G | IGPGRAFYT | 2;9 | H2-D1*02;H2-D1*02 |
| 5TEZ | GILGFVFTL | 2;9 | HLA-A*0201;HLA-A*0203;HLA-A*0204;HLA-A*0207;HLA-A*0209;HLA-A*0212;HLA-A*0213;HLA-A*0216;HLA-A*0217;HLA-A*0218;HLA-A*0222;HLA-A*0224;HLA-A*0226;HLA-A*0233;HLA-A*0266;HLA-A*0289;HLA-A*0268;HLA-A*0265;HLA-A*0277;HLA-A*0260;HLA-A*9232;HLA-A*9234;HLA-A*9235;HLA-A*0201;HLA-A*0209;HLA-A*0266;HLA-A*0289;HLA-A*9232;HLA-A*9234 |
| 5TIL | KAPYNFATM | 2;9 | H2-D1b;H2-D1b |
| 5TJE | KAVYNFATM | 2;9 | H2-D1b;H2-D1b |
| 5TRZ | YQSGLSIVM | 1;8 | MH1-K1*02;MH1-K1*02 |
| 5TXS | AQDIYRASY | 2;9 | HLA-B*1501;HLA-B*1504;HLA-B*1512;HLA-B*1514;HLA-B*1519;HLA-B*1520;HLA-B*1525;HLA-B*1527;HLA-B*1532;HLA-B*1542;HLA-B*1558;HLA-B*1573;HLA-B*1577;HLA-B*1583;HLA-B*9528;HLA-B*9541;HLA-B*9546;HLA-B*1501;HLA-B*9546 |
| 5V5L | TSTLQEQIGW | 3;10 | HLA-C*1510 |
| 5V5M | TSTLQEQIGW | 3;10 | HLA-C*1510 |
| 5VCL | AMAPRTLLL | 2;9 | H2-T23*02;H2-T23*02 |
| 5VGD | SAEPVPLQL | 2;9 | HLA-C*0501;HLA-C*0503;HLA-C*0504;HLA-C*0511;HLA-C*0510;HLA-C*0505;HLA-C*0512;HLA-C*0810;HLA-C*0509;HLA-C*0508;HLA-C*0514;HLA-C*0516;HLA-C*1221;HLA-C*0517;HLA-C*0523;HLA-C*0518;HLA-C*0522;HLA-C*0519;HLA-C*0527;HLA-C*0537;HLA-C*0531;HLA-C*1233;HLA-C*0541;HLA-C*0534;HLA-C*0538;HLA-C*0539;HLA-C*0501;HLA-C*0503;HLA-C*0537 |
| 5VGE | RYRPGTVAL | 2;9 | HLA-C*0702;HLA-C*0705;HLA-C*0708;HLA-C*0710;HLA-C*0711;HLA-C*0712;HLA-C*0713;HLA-C*0704;HLA-C*0714;HLA-C*0715;HLA-C*0723;HLA-C*0727;HLA-C*0611;HLA-C*0731;HLA-C*0725;HLA-C*0729;HLA-C*0717;HLA-C*0738;HLA-C*0737;HLA-C*0745;HLA-C*0743;HLA-C*0748;HLA-C*0747;HLA-C*0741;HLA-C*0742;HLA-C*0750;HLA-C*0756;HLA-C*0754;HLA-C*0763;HLA-C*0768;HLA-C*0766;HLA-C*0767;HLA-C*0774;HLA-C*0787;HLA-C*0799;HLA-C*0702;HLA-C*0750;HLA-C*0766;HLA-C*0774 |
| 5VUD | LSSPVTKSW | 2;9 | HLA-C*1510 |
| 5VUE | LTVQVARVW | 2;9 | HLA-C*1510 |
| 5VUF | LTVQVARVY | 2;9 | HLA-C*1510 |
| 5VVP | LSSPVTKSW | 2;9 | HLA-C*1510 |
| 5VWD | LTVQVARVW | 2;9 | HLA-C*1510 |
| 5VWF | LTVQVARVY | 2;9 | HLA-C*1510 |
| 5VWH | LSSPVTKSW | 2;9 | HLA-C*1510 |
| 5VWJ | LTVQVARVW | 2;9 | HLA-C*1510 |
| 5VZ5 | AQDIYRASYY | 2;10 | HLA-C*1604;HLA-C*1601;HLA-C*0813;HLA-C*1606;HLA-C*1212;HLA-C*0318;HLA-C*1607 |
| 5W1V | VMAPRTLIL | 2;9 | HLA-C*0102;HLA-C*0103;HLA-C*0104;HLA-C*0105;HLA-C*0110;HLA-C*0108;HLA-C*0106;HLA-C*0111;HLA-C*0109;HLA-C*0107;HLA-C*0112;HLA-C*0113 |
| 5W69 | ARFNDLRFV | 2;9 | HLA-C*0602;HLA-C*0606;HLA-C*1801;HLA-C*1802;HLA-C*0612;HLA-C*0617;HLA-C*0624;HLA-C*0602;HLA-C*0617 |
| 5WEU | RGPGCAFVTI | 2;10 | H2-D1*02;H2-D1*02 |
| 5WJL | GTSGSPIVNR | 2;10 | HLA-C*1510 |
| 5WJN | GTSGSPIINR | 2;10 | HLA-C*1510 |
| 5WKF | GTSGSPIVNR | 2;10 | HLA-C*1510 |
| 5WKH | GTSGSPIINR | 2;10 | HLA-C*1510 |
| 5WLG | SQLLNAKYL | 2;9 | H2-D1b;H2-D1b |
| 5WLI | SQLLNAKYL | 2;9 | H2-D1b;H2-D1b |
| 5WMN | SPIVPSFDM | 2;9 | HLA-B*0702;HLA-B*0704;HLA-B*0705;HLA-B*0706;HLA-B*0707;HLA-B*0709;HLA-B*0714;HLA-B*0718;HLA-B*4201;HLA-B*6701;HLA-B*8101;HLA-B*0742;HLA-B*0744;HLA-B*4205;HLA-B*0733;HLA-B*4208;HLA-B*8102;HLA-B*0759;HLA-B*0761;HLA-B*0777;HLA-B*0702;HLA-B*0744;HLA-B*0759;HLA-B*0761 |
| 5WMO | RPPIFIRRL | 2;9 | HLA-B*0702;HLA-B*0704;HLA-B*0705;HLA-B*0706;HLA-B*0707;HLA-B*0709;HLA-B*0714;HLA-B*0718;HLA-B*4201;HLA-B*6701;HLA-B*8101;HLA-B*0742;HLA-B*0744;HLA-B*4205;HLA-B*0733;HLA-B*4208;HLA-B*8102;HLA-B*0759;HLA-B*0761;HLA-B*0777;HLA-B*0702;HLA-B*0744;HLA-B*0759;HLA-B*0761 |
| 5WMP | TPRVTGGGAM | 2;10 | HLA-B*0702;HLA-B*0704;HLA-B*0705;HLA-B*0706;HLA-B*0707;HLA-B*0709;HLA-B*0714;HLA-B*0718;HLA-B*4201;HLA-B*6701;HLA-B*8101;HLA-B*0742;HLA-B*0744;HLA-B*4205;HLA-B*0733;HLA-B*4208;HLA-B*8102;HLA-B*0759;HLA-B*0761;HLA-B*0777;HLA-B*0702;HLA-B*0744;HLA-B*0759;HLA-B*0761 |
| 5WMQ | ELRSRYWAI | 2;9 | HLA-B*0801;HLA-B*0807;HLA-B*0809;HLA-B*0811;HLA-B*0812;HLA-B*0813;HLA-B*0814;HLA-B*0827;HLA-B*0822;HLA-B*0820;HLA-B*0825;HLA-B*0821;HLA-B*0818;HLA-B*0828;HLA-B*0832;HLA-B*0831;HLA-B*0833;HLA-B*0835;HLA-B*0837;HLA-B*0840;HLA-B*0839;HLA-B*0842;HLA-B*0843;HLA-B*0853;HLA-B*0858;HLA-B*0848;HLA-B*0849;HLA-B*0860;HLA-B*0855;HLA-B*9580;HLA-B*0801;HLA-B*0818;HLA-B*0832 |
| 5WMR | QIKVRVDMV | 2;9 | HLA-B*0801;HLA-B*0820;HLA-B*0825;HLA-B*0833;HLA-B*9580;HLA-B*0801 |
| 5WSH | GVWIRTPTA | 2;9 | HLA-A*0201;HLA-A*0203;HLA-A*0204;HLA-A*0207;HLA-A*0209;HLA-A*0212;HLA-A*0213;HLA-A*0216;HLA-A*0217;HLA-A*0218;HLA-A*0222;HLA-A*0224;HLA-A*0226;HLA-A*0233;HLA-A*0266;HLA-A*0289;HLA-A*0268;HLA-A*0265;HLA-A*0277;HLA-A*0260;HLA-A*9232;HLA-A*9234;HLA-A*9235;HLA-A*0201;HLA-A*0209;HLA-A*0266;HLA-A*0289;HLA-A*9232;HLA-A*9234 |
| 5WWI | LYKKLKREMTF | 2;11 | HLA-A*2301;HLA-A*2402;HLA-A*2403;HLA-A*2410;HLA-A*2422;HLA-A*2426;HLA-A*2427;HLA-A*2454;HLA-A*2453;HLA-A*2451;HLA-A*2479;HLA-A*2317;HLA-A*2402;HLA-A*2479 |
| 5WWU | LYKKLKREITF | 2;11 | HLA-A*2301;HLA-A*2402;HLA-A*2403;HLA-A*2410;HLA-A*2422;HLA-A*2426;HLA-A*2427;HLA-A*2454;HLA-A*2453;HLA-A*2451;HLA-A*2479;HLA-A*2317;HLA-A*2402;HLA-A*2479 |
| 5WXD | LYKKLKREMTF | 2;11 | HLA-A*2301;HLA-A*2402;HLA-A*2403;HLA-A*2410;HLA-A*2422;HLA-A*2426;HLA-A*2427;HLA-A*2454;HLA-A*2453;HLA-A*2451;HLA-A*2479;HLA-A*2317;HLA-A*2402;HLA-A*2479 |
| 5XMF | RMANVSTGR | 2;9 | MH1-N*01802 |
| 5XMM | DMANVSTGR | 2;9 | MH1-N*01802 |
| 5XOS | IPLTEEAEL | 2;9 | HLA-C*1604;HLA-C*1601;HLA-C*0813;HLA-C*1606;HLA-C*1212;HLA-C*0318;HLA-C*1607 |
| 5XOT | IPLTEEAEL | 2;9 | HLA-C*1604;HLA-C*1601;HLA-C*0813;HLA-C*1606;HLA-C*1212;HLA-C*0318;HLA-C*1607 |
| 5XOV | RYPLTFGWCF | 2;10 | HLA-A*2301;HLA-A*2402;HLA-A*2403;HLA-A*2410;HLA-A*2422;HLA-A*2426;HLA-A*2427;HLA-A*2454;HLA-A*2453;HLA-A*2451;HLA-A*2479;HLA-A*2317;HLA-A*2402;HLA-A*2479 |
| 5XS3 | VRSRRCLRL | 2;9 | HLA-C*0602;HLA-C*0604;HLA-C*0606;HLA-C*1801;HLA-C*1802;HLA-C*0608;HLA-C*0613;HLA-C*0609;HLA-C*0612;HLA-C*0615;HLA-C*1803;HLA-C*0614;HLA-C*0617;HLA-C*0622;HLA-C*0623;HLA-C*0624;HLA-C*0620;HLA-C*0776;HLA-C*0627;HLA-C*0621;HLA-C*0637;HLA-C*0631;HLA-C*0636;HLA-C*0635;HLA-C*0634;HLA-C*0602;HLA-C*0617 |
| 5Y91 | FANFCLMMI | 2;9 | MH1-A*01;MH1-A*01 |
| 5YLX | TMPPGFELY | 2;9 | MH1*0401;MH1*0401 |
| 5YMV | AVKGVGTMV | 2;8 | MH1-B*2401;MH1-B*2401 |
| 5YMW | LPACVLEV | 2;8 | MH1-B*2401;MH1-B*2401 |
| 5YXN | KLVALGINAV | 2;10 | HLA-A*0201;HLA-A*0203;HLA-A*0204;HLA-A*0207;HLA-A*0209;HLA-A*0212;HLA-A*0213;HLA-A*0216;HLA-A*0217;HLA-A*0218;HLA-A*0222;HLA-A*0224;HLA-A*0226;HLA-A*0233;HLA-A*0266;HLA-A*0289;HLA-A*0268;HLA-A*0265;HLA-A*0277;HLA-A*0260;HLA-A*9232;HLA-A*9234;HLA-A*9235;HLA-A*0201;HLA-A*0209;HLA-A*0266;HLA-A*0289;HLA-A*9232;HLA-A*9234 |
| 5YXU | KLVALGINAV | 2;10 | HLA-A*0201;HLA-A*0203;HLA-A*0204;HLA-A*0207;HLA-A*0209;HLA-A*0212;HLA-A*0213;HLA-A*0216;HLA-A*0217;HLA-A*0218;HLA-A*0222;HLA-A*0224;HLA-A*0226;HLA-A*0233;HLA-A*0266;HLA-A*0289;HLA-A*0268;HLA-A*0265;HLA-A*0277;HLA-A*0260;HLA-A*9232;HLA-A*9234;HLA-A*9235;HLA-A*0201;HLA-A*0209;HLA-A*0266;HLA-A*0289;HLA-A*9232;HLA-A*9234 |
| 6A6H | MTAHIVVPY | 2;9 | MH1*0401;MH1*0401 |
| 6AEE | RIIPRHLQL | 2;9 | HLA-G*0101;HLA-G*0104;HLA-G*0106;HLA-G*0108;HLA-G*0109;HLA-G*0114;HLA-G*0115;HLA-G*0116;HLA-G*0117;HLA-G*0104 |
| 6AMT | MMWDRGLGMM | 2;10 | HLA-A*0201;HLA-A*0203;HLA-A*0204;HLA-A*0207;HLA-A*0209;HLA-A*0212;HLA-A*0213;HLA-A*0216;HLA-A*0217;HLA-A*0218;HLA-A*0222;HLA-A*0224;HLA-A*0226;HLA-A*0233;HLA-A*0266;HLA-A*0289;HLA-A*0268;HLA-A*0265;HLA-A*0277;HLA-A*0260;HLA-A*9232;HLA-A*9234;HLA-A*9235;HLA-A*0201;HLA-A*0209;HLA-A*0266;HLA-A*0289;HLA-A*9232;HLA-A*9234 |
| 6AMU | MMWDRGLGMM | 2;9 | HLA-A*0201;HLA-A*0203;HLA-A*0204;HLA-A*0207;HLA-A*0209;HLA-A*0212;HLA-A*0213;HLA-A*0216;HLA-A*0217;HLA-A*0218;HLA-A*0219;HLA-A*0222;HLA-A*0224;HLA-A*0225;HLA-A*0226;HLA-A*0227;HLA-A*0233;HLA-A*0236;HLA-A*0237;HLA-A*0238;HLA-A*0239;HLA-A*0240;HLA-A*0249;HLA-A*0252;HLA-A*0266;HLA-A*0289;HLA-A*0271;HLA-A*0268;HLA-A*0274;HLA-A*0258;HLA-A*0280;HLA-A*0265;HLA-A*0286;HLA-A*0277;HLA-A*0260;HLA-A*0273;HLA-A*0285;HLA-A*0264;HLA-A*0275;HLA-A*0267;HLA-A*0295;HLA-A*0297;HLA-A*9201;HLA-A*0296;HLA-A*0293;HLA-A*9221;HLA-A*9231;HLA-A*9232;HLA-A*9214;HLA-A*9202;HLA-A*9210;HLA-A*9205;HLA-A*9217;HLA-A*9211;HLA-A*9204;HLA-A*9230;HLA-A*9234;HLA-A*9218;HLA-A*9247;HLA-A*9249;HLA-A*9251;HLA-A*9248;HLA-A*9256;HLA-A*9235;HLA-A*9252;HLA-A*9240;HLA-A*9253;HLA-A*9245;HLA-A*9277;HLA-A*9257;HLA-A*9271;HLA-A*9294;HLA-A*9264;HLA-A*9290;HLA-A*9266;HLA-A*9291;HLA-A*9289;HLA-A*9274;HLA-A*9262;HLA-A*9259;HLA-A*9263;HLA-A*9268;HLA-A*9288;HLA-A*9293;HLA-A*9287;HLA-A*9283;HLA-A*9267;HLA-A*9265;HLA-A*9282;HLA-A*9297;HLA-A*9298;HLA-A*0201;HLA-A*0209;HLA-A*0266;HLA-A*0289;HLA-A*0275;HLA-A*0297;HLA-A*9232;HLA-A*9234;HLA-A*9240 |
| 6AT5 | APRGPHGGAASGL | 2;13 | HLA-B*0702;HLA-B*0704;HLA-B*0705;HLA-B*0706;HLA-B*0707;HLA-B*0709;HLA-B*0714;HLA-B*0718;HLA-B*4201;HLA-B*6701;HLA-B*0742;HLA-B*0744;HLA-B*4205;HLA-B*0733;HLA-B*4208;HLA-B*8102;HLA-B*0759;HLA-B*0761;HLA-B*0777;HLA-B*0702;HLA-B*0744;HLA-B*0759;HLA-B*0761 |
| 6AT9 | AQDIYRASYY | 2;10 | HLA-C*1510 |
| 6AVF | APRGPHGGAASGL | 2;13 | HLA-B*0702;HLA-B*0704;HLA-B*0705;HLA-B*0706;HLA-B*0707;HLA-B*0709;HLA-B*0714;HLA-B*0718;HLA-B*4201;HLA-B*6701;HLA-B*0742;HLA-B*0744;HLA-B*4205;HLA-B*0733;HLA-B*4208;HLA-B*8102;HLA-B*0759;HLA-B*0761;HLA-B*0777;HLA-B*0702;HLA-B*0744;HLA-B*0759;HLA-B*0761 |
| 6AVG | APRGPHGGAASGL | 2;13 | HLA-B*0702;HLA-B*0704;HLA-B*0705;HLA-B*0706;HLA-B*0707;HLA-B*0709;HLA-B*0714;HLA-B*0718;HLA-B*4201;HLA-B*6701;HLA-B*0742;HLA-B*0744;HLA-B*4205;HLA-B*0733;HLA-B*4208;HLA-B*8102;HLA-B*0759;HLA-B*0761;HLA-B*0777;HLA-B*0702;HLA-B*0744;HLA-B*0759;HLA-B*0761 |
| 6D29 | TSMSFVPRPW | 2;10 | HLA-B*5701;HLA-B*5702;HLA-B*5703;HLA-B*5706;HLA-B*5711;HLA-B*5701 |
| 6D2R | GSFDYSGVHLW | 2;11 | HLA-B*5701;HLA-B*5702;HLA-B*5703;HLA-B*5706;HLA-B*5711;HLA-B*5701 |
| 6D2T | LALLTGVRW | 2;9 | HLA-B*5701;HLA-B*5702;HLA-B*5703;HLA-B*5706;HLA-B*5711;HLA-B*5701 |
| 6D78 | AAGIGILTV | 1;9 | H2-Q10*02 |
| 6DKP | ELAGIGILTV | 2;10 | HLA-A*0201;HLA-A*0203;HLA-A*0204;HLA-A*0207;HLA-A*0209;HLA-A*0212;HLA-A*0213;HLA-A*0216;HLA-A*0217;HLA-A*0218;HLA-A*0222;HLA-A*0224;HLA-A*0226;HLA-A*0233;HLA-A*0266;HLA-A*0289;HLA-A*0268;HLA-A*0265;HLA-A*0277;HLA-A*0260;HLA-A*9232;HLA-A*9234;HLA-A*9235;HLA-A*0201;HLA-A*0209;HLA-A*0266;HLA-A*0289;HLA-A*9232;HLA-A*9234 |
| 6EQA | AAGIGILTV | 1;9 | HLA-A*0201;HLA-A*0203;HLA-A*0204;HLA-A*0207;HLA-A*0209;HLA-A*0212;HLA-A*0213;HLA-A*0216;HLA-A*0217;HLA-A*0218;HLA-A*0222;HLA-A*0224;HLA-A*0226;HLA-A*0233;HLA-A*0266;HLA-A*0289;HLA-A*0268;HLA-A*0265;HLA-A*0277;HLA-A*0260;HLA-A*9232;HLA-A*9234;HLA-A*9235;HLA-A*0201;HLA-A*0209;HLA-A*0266;HLA-A*0289;HLA-A*9232;HLA-A*9234 |
| 6EQB | AAGIGILTV | 1;9 | HLA-A*0201;HLA-A*0203;HLA-A*0204;HLA-A*0207;HLA-A*0209;HLA-A*0212;HLA-A*0213;HLA-A*0216;HLA-A*0217;HLA-A*0218;HLA-A*0222;HLA-A*0224;HLA-A*0226;HLA-A*0233;HLA-A*0266;HLA-A*0289;HLA-A*0268;HLA-A*0265;HLA-A*0277;HLA-A*0260;HLA-A*9232;HLA-A*9234;HLA-A*9235;HLA-A*0201;HLA-A*0209;HLA-A*0266;HLA-A*0289;HLA-A*9232;HLA-A*9234 |
| 6EWA | ILKEPVHGV | 2;9 | HLA-A*0201;HLA-A*0203;HLA-A*0204;HLA-A*0207;HLA-A*0209;HLA-A*0212;HLA-A*0213;HLA-A*0216;HLA-A*0217;HLA-A*0218;HLA-A*0222;HLA-A*0224;HLA-A*0226;HLA-A*0233;HLA-A*0266;HLA-A*0289;HLA-A*0268;HLA-A*0265;HLA-A*0277;HLA-A*0260;HLA-A*9232;HLA-A*9234;HLA-A*9235;HLA-A*0201;HLA-A*0209;HLA-A*0266;HLA-A*0289;HLA-A*9232;HLA-A*9234 |
| 6EWC | RLSSPLHFV | 2;9 | HLA-A*0201;HLA-A*0203;HLA-A*0204;HLA-A*0207;HLA-A*0209;HLA-A*0212;HLA-A*0213;HLA-A*0216;HLA-A*0217;HLA-A*0218;HLA-A*0222;HLA-A*0224;HLA-A*0226;HLA-A*0233;HLA-A*0266;HLA-A*0289;HLA-A*0268;HLA-A*0265;HLA-A*0277;HLA-A*0260;HLA-A*9232;HLA-A*9234;HLA-A*9235;HLA-A*0201;HLA-A*0209;HLA-A*0266;HLA-A*0289;HLA-A*9232;HLA-A*9234 |
| 6EWO | RTFSPTYGL | 2;9 | HLA-A*0201;HLA-A*0203;HLA-A*0204;HLA-A*0207;HLA-A*0209;HLA-A*0212;HLA-A*0213;HLA-A*0216;HLA-A*0217;HLA-A*0218;HLA-A*0222;HLA-A*0224;HLA-A*0226;HLA-A*0233;HLA-A*0266;HLA-A*0289;HLA-A*0268;HLA-A*0265;HLA-A*0277;HLA-A*0260;HLA-A*9232;HLA-A*9234;HLA-A*9235;HLA-A*0201;HLA-A*0209;HLA-A*0266;HLA-A*0289;HLA-A*9232;HLA-A*9234 |
| 6G3J | MTSAIGILPV | 2;10 | HLA-A*0201;HLA-A*0203;HLA-A*0204;HLA-A*0207;HLA-A*0209;HLA-A*0212;HLA-A*0213;HLA-A*0216;HLA-A*0217;HLA-A*0218;HLA-A*0222;HLA-A*0224;HLA-A*0226;HLA-A*0233;HLA-A*0266;HLA-A*0289;HLA-A*0268;HLA-A*0265;HLA-A*0277;HLA-A*0260;HLA-A*9232;HLA-A*9234;HLA-A*9235;HLA-A*0201;HLA-A*0209;HLA-A*0266;HLA-A*0289;HLA-A*9232;HLA-A*9234 |
| 6G3K | ITSGIGVLPV | 2;10 | HLA-A*0201;HLA-A*0203;HLA-A*0204;HLA-A*0207;HLA-A*0209;HLA-A*0212;HLA-A*0213;HLA-A*0216;HLA-A*0217;HLA-A*0218;HLA-A*0222;HLA-A*0224;HLA-A*0226;HLA-A*0233;HLA-A*0266;HLA-A*0289;HLA-A*0268;HLA-A*0265;HLA-A*0277;HLA-A*0260;HLA-A*9232;HLA-A*9234;HLA-A*9235;HLA-A*0201;HLA-A*0209;HLA-A*0266;HLA-A*0289;HLA-A*9232;HLA-A*9234 |
| 6G9Q | KAPYDYAPI | 2;9 | H2-D1b;H2-D1b |
| 6G9R | KAPYDYAPI | 2;9 | H2-D1b;H2-D1b |
| 6GB5 | FAPGNYP | 2;5 | H2-D1b;H2-D1b |
| 6GB7 | FAPGNYP | 2;5 | H2-D1b;H2-D1b |
| 6GGM | RFPAKAPLL | 2;9 | HLA-C*0102;HLA-C*0103;HLA-C*0104;HLA-C*0105;HLA-C*0110;HLA-C*0108;HLA-C*0106;HLA-C*0111;HLA-C*0109;HLA-C*0107;HLA-C*0112;HLA-C*0113 |
| 6GH1 | RLPAKAPLL | 2;9 | HLA-C*0102;HLA-C*0103;HLA-C*0104;HLA-C*0105;HLA-C*0110;HLA-C*0108;HLA-C*0106;HLA-C*0111;HLA-C*0109;HLA-C*0107;HLA-C*0112;HLA-C*0113 |
| 6GH4 | RQPAKAPLL | 2;9 | HLA-C*0102;HLA-C*0103;HLA-C*0104;HLA-C*0105;HLA-C*0110;HLA-C*0108;HLA-C*0106;HLA-C*0111;HLA-C*0109;HLA-C*0107;HLA-C*0112;HLA-C*0113 |
| 6GHN | RLPAKAPLF | 2;9 | HLA-C*0102;HLA-C*0103;HLA-C*0104;HLA-C*0105;HLA-C*0110;HLA-C*0108;HLA-C*0106;HLA-C*0111;HLA-C*0109;HLA-C*0107;HLA-C*0112;HLA-C*0113 |
| 6GL1 | RMYSPTSIL | 2;9 | HLA-C*0102;HLA-C*0103;HLA-C*0104;HLA-C*0105;HLA-C*0110;HLA-C*0108;HLA-C*0106;HLA-C*0111;HLA-C*0109;HLA-C*0107;HLA-C*0112;HLA-C*0113 |
| 6H6D | SGPSNTPPEI | 2;10 | H2-D1b;H2-D1b |
| 6H6H | SGPSNTPPEI | 2;10 | H2-D1b;H2-D1b |
| 6ID4 | AIFQSSMTK | 2;9 | HLA-C*1510;HLA-A*1101;HLA-A*1102;HLA-A*1106;HLA-A*1110 |
| 6IEX | GETALALLLL | 2;10 | HLA-B*4001;HLA-B*4016;HLA-B*4023;HLA-B*4101;HLA-B*4102;HLA-B*4501;HLA-B*4504;HLA-B*5001;HLA-B*5002;HLA-B*4051;HLA-B*3563;HLA-B*4060;HLA-B*4059;HLA-B*4077;HLA-B*4072;HLA-B*4001 |
| 6IRL | RREVHTYY | 2;8 | MH1-B*2101;MH1-B*2101 |
| 6J1V | AIFQSSMTK | 2;9 | HLA-A*3003;HLA-A*3003 |
| 6J1W | AIFQSSMTK | 2;9 | HLA-A*3001;HLA-A*3001 |
| 6J29 | QIMYNYPAM | 2;9 | HLA-A*3003;HLA-A*3003 |
| 6J2A | CTELKLSDY | 2;9 | HLA-A*3003;HLA-A*3003 |
| 6JOZ | ATIGTAMYK | 2;9 | HLA-C*1510;HLA-A*1101;HLA-A*1102;HLA-A*1106;HLA-A*1110;HLA-A*1111 |
| 6JQ3 | SIIVFNLL | 2;8 | MH1-K1b;MH1-K1b |
| 6JTN | GADGVGKSAL | 2;10 | HLA-C*0801;HLA-C*0802;HLA-C*0803;HLA-C*0804;HLA-C*0806;HLA-C*0807;HLA-C*0808;HLA-C*0809;HLA-C*0811;HLA-C*0812;HLA-C*0815;HLA-C*0819;HLA-C*0824;HLA-C*0822;HLA-C*0827;HLA-C*0823;HLA-C*0820;HLA-C*0829;HLA-C*0831;HLA-C*0830;HLA-C*0802 |
| 6JTO | GADGVGKSAL | 2;10 | HLA-C*0501;HLA-C*0503;HLA-C*0504;HLA-C*0511;HLA-C*0510;HLA-C*0505;HLA-C*0512;HLA-C*0810;HLA-C*0509;HLA-C*0508;HLA-C*0514;HLA-C*0516;HLA-C*1221;HLA-C*0517;HLA-C*0523;HLA-C*0518;HLA-C*0522;HLA-C*0519;HLA-C*0527;HLA-C*0537;HLA-C*0531;HLA-C*1233;HLA-C*0541;HLA-C*0534;HLA-C*0538;HLA-C*0539;HLA-C*0501;HLA-C*0503;HLA-C*0537 |
| 6JTP | GADGVGKSA | 2;9 | HLA-C*0801;HLA-C*0802;HLA-C*0803;HLA-C*0804;HLA-C*0806;HLA-C*0807;HLA-C*0808;HLA-C*0809;HLA-C*0811;HLA-C*0812;HLA-C*0815;HLA-C*0819;HLA-C*0824;HLA-C*0822;HLA-C*0827;HLA-C*0823;HLA-C*0820;HLA-C*0829;HLA-C*0831;HLA-C*0830;HLA-C*0802 |
| 6K60 | RIIPRHLQL | 2;9 | HLA-G*0101;HLA-G*0104;HLA-G*0106;HLA-G*0108;HLA-G*0109;HLA-G*0114;HLA-G*0115;HLA-G*0116;HLA-G*0117;HLA-G*0101;HLA-G*0106;HLA-G*0108 |
| 6KWK | MTAHITVPY | 2;9 | MH1*0401;MH1*0401 |
| 6KWL | MTAHITVPY | 2;9 | MH1*0401;MH1*0401 |
| 6KWN | NSDTVGWSW | 2;9 | MH1*0401;MH1*0401 |
| 6KWO | ESDTVGWSW | 2;9 | MH1*0401;MH1*0401 |
| 6LBE | FANFCLMMI | 2;9 | MH1-A*01;MH1-A*01 |
| 6LHF | RRREQTDY | 2;8 | MH1-B*2101;MH1-B*2101 |
| 6LHG | IDWFDGKE | 2;8 | MH1-B*2401;MH1-B*2401 |
| 6LHH | RRREQTDY | 2;8 | MH1-B*2101;MH1-B*2101 |
| 6MPP | ILDTAGKEEY | 2;10 | HLA-C*1510 |
| 6MT3 | FEDLRVLSF | 2;9 | HLA-B*1801;HLA-B*1802;HLA-B*1820;HLA-B*1826;HLA-B*1801 |
| 6MT4 | FEDLRVSSF | 2;9 | HLA-B*3701;HLA-B*3702;HLA-B*3704;HLA-B*3709;HLA-B*3701 |
| 6MT5 | FEDLRLLSF | 2;9 | HLA-B*3701;HLA-B*3702;HLA-B*3704;HLA-B*3709;HLA-B*3701 |
| 6MT6 | FEDLRVLSF | 2;9 | HLA-B*3701;HLA-B*3702;HLA-B*3704;HLA-B*3709;HLA-B*3701 |
| 6MTL | FEDLRVLSF | 2;9 | HLA-C*1510;HLA-C*1205;HLA-C*1602;HLA-C*1209 |
| 6MTM | FEDLRVLSF | 2;9 | HLA-B*3701;HLA-B*3702;HLA-B*3704;HLA-B*3709;HLA-B*3701 |
| 6NCA | YVLDHLIVV | 2;9 | HLA-A*0201;HLA-A*0203;HLA-A*0204;HLA-A*0207;HLA-A*0209;HLA-A*0212;HLA-A*0213;HLA-A*0216;HLA-A*0217;HLA-A*0218;HLA-A*0222;HLA-A*0224;HLA-A*0226;HLA-A*0233;HLA-A*0266;HLA-A*0289;HLA-A*0268;HLA-A*0265;HLA-A*0277;HLA-A*0260;HLA-A*9232;HLA-A*9234;HLA-A*9235;HLA-A*0201;HLA-A*0209;HLA-A*0266;HLA-A*0289;HLA-A*9232;HLA-A*9234 |
| 6NF7 | YLRYDSDVGEYR | 2;12 | RT1-A;RT1-A |
| 6NPR | RGPGRAFVTI | 2;10 | H2-D1*02;H2-D1*02 |
| 6O4Y | KLVVGAVGV | 2;9 | HLA-A*0201;HLA-A*0203;HLA-A*0204;HLA-A*0207;HLA-A*0209;HLA-A*0212;HLA-A*0213;HLA-A*0216;HLA-A*0217;HLA-A*0218;HLA-A*0222;HLA-A*0224;HLA-A*0226;HLA-A*0233;HLA-A*0266;HLA-A*0289;HLA-A*0268;HLA-A*0265;HLA-A*0277;HLA-A*0260;HLA-A*9232;HLA-A*9234;HLA-A*9235;HLA-A*0201;HLA-A*0209;HLA-A*0266;HLA-A*0289;HLA-A*9232;HLA-A*9234 |
| 6O4Z | KLVVVAVGV | 2;9 | HLA-A*0201;HLA-A*0203;HLA-A*0204;HLA-A*0207;HLA-A*0209;HLA-A*0212;HLA-A*0213;HLA-A*0216;HLA-A*0217;HLA-A*0218;HLA-A*0222;HLA-A*0224;HLA-A*0226;HLA-A*0233;HLA-A*0266;HLA-A*0289;HLA-A*0268;HLA-A*0265;HLA-A*0277;HLA-A*0260;HLA-A*9232;HLA-A*9234;HLA-A*9235;HLA-A*0201;HLA-A*0209;HLA-A*0266;HLA-A*0289;HLA-A*9232;HLA-A*9234 |
| 6O51 | YLVVVGAVGV | 2;10 | HLA-A*0201;HLA-A*0203;HLA-A*0204;HLA-A*0207;HLA-A*0209;HLA-A*0212;HLA-A*0213;HLA-A*0216;HLA-A*0217;HLA-A*0218;HLA-A*0222;HLA-A*0224;HLA-A*0226;HLA-A*0233;HLA-A*0266;HLA-A*0289;HLA-A*0268;HLA-A*0265;HLA-A*0277;HLA-A*0260;HLA-A*9232;HLA-A*9234;HLA-A*9235;HLA-A*0201;HLA-A*0209;HLA-A*0266;HLA-A*0289;HLA-A*9232;HLA-A*9234 |
| 6O53 | KLVVVGAVGV | 2;10 | HLA-A*0201;HLA-A*0203;HLA-A*0204;HLA-A*0207;HLA-A*0209;HLA-A*0212;HLA-A*0213;HLA-A*0216;HLA-A*0217;HLA-A*0218;HLA-A*0222;HLA-A*0224;HLA-A*0226;HLA-A*0233;HLA-A*0266;HLA-A*0289;HLA-A*0268;HLA-A*0265;HLA-A*0277;HLA-A*0260;HLA-A*9232;HLA-A*9234;HLA-A*9235;HLA-A*0201;HLA-A*0209;HLA-A*0266;HLA-A*0289;HLA-A*9232;HLA-A*9234 |
| 6O9B | TTAPSLSGK | 2;9 | HLA-A*0306;HLA-A*0307;HLA-A*0310;HLA-A*0319;HLA-A*0316;HLA-A*0313;HLA-A*0320;HLA-A*0322;HLA-A*0315;HLA-A*0327;HLA-A*0338;HLA-A*0331;HLA-A*0328;HLA-A*0335;HLA-A*0344;HLA-A*7413;HLA-A*0340;HLA-A*0343;HLA-A*0346;HLA-A*0342;HLA-A*0345;HLA-A*0351;HLA-A*0350;HLA-A*0356;HLA-A*0352;HLA-A*0361;HLA-A*0360;HLA-A*0353;HLA-A*0362;HLA-A*0354;HLA-A*0349;HLA-A*0364;HLA-A*0365;HLA-A*0373;HLA-A*0374;HLA-A*0375;HLA-A*0366;HLA-A*0320;HLA-A*0345 |
| 6O9C | TTAPFLSGK | 2;9 | HLA-A*0306;HLA-A*0307;HLA-A*0310;HLA-A*0319;HLA-A*0316;HLA-A*0313;HLA-A*0320;HLA-A*0322;HLA-A*0315;HLA-A*0327;HLA-A*0338;HLA-A*0331;HLA-A*0328;HLA-A*0335;HLA-A*0344;HLA-A*7413;HLA-A*0340;HLA-A*0343;HLA-A*0346;HLA-A*0342;HLA-A*0345;HLA-A*0351;HLA-A*0350;HLA-A*0356;HLA-A*0352;HLA-A*0361;HLA-A*0360;HLA-A*0353;HLA-A*0362;HLA-A*0354;HLA-A*0349;HLA-A*0364;HLA-A*0365;HLA-A*0373;HLA-A*0374;HLA-A*0375;HLA-A*0366;HLA-A*0320;HLA-A*0345 |
| 6OPD | ILNAMIVKI | 2;9 | HLA-A*0201;HLA-A*0203;HLA-A*0204;HLA-A*0207;HLA-A*0209;HLA-A*0212;HLA-A*0213;HLA-A*0216;HLA-A*0217;HLA-A*0218;HLA-A*0222;HLA-A*0224;HLA-A*0226;HLA-A*0233;HLA-A*0266;HLA-A*0289;HLA-A*0268;HLA-A*0265;HLA-A*0277;HLA-A*0260;HLA-A*9232;HLA-A*9234;HLA-A*9235;HLA-A*0201;HLA-A*0209;HLA-A*0266;HLA-A*0289;HLA-A*9232;HLA-A*9234 |
| 6P23 | AAAKKKYCL | 3;9 | HLA-B*0801;HLA-B*0807;HLA-B*0809;HLA-B*0811;HLA-B*0812;HLA-B*0813;HLA-B*0814;HLA-B*0815;HLA-B*0827;HLA-B*0822;HLA-B*0820;HLA-B*0825;HLA-B*0821;HLA-B*0818;HLA-B*0828;HLA-B*0831;HLA-B*0833;HLA-B*0835;HLA-B*0837;HLA-B*0840;HLA-B*0839;HLA-B*0842;HLA-B*0843;HLA-B*0853;HLA-B*0858;HLA-B*0848;HLA-B*0849;HLA-B*0860;HLA-B*0855;HLA-B*9580;HLA-B*0801;HLA-B*0815;HLA-B*0818 |
| 6P27 | AAAKKKYCL | 3;9 | HLA-B*0801;HLA-B*0807;HLA-B*0809;HLA-B*0811;HLA-B*0812;HLA-B*0813;HLA-B*0814;HLA-B*0815;HLA-B*0827;HLA-B*0822;HLA-B*0820;HLA-B*0825;HLA-B*0821;HLA-B*0818;HLA-B*0828;HLA-B*0831;HLA-B*0833;HLA-B*0835;HLA-B*0837;HLA-B*0840;HLA-B*0839;HLA-B*0842;HLA-B*0843;HLA-B*0853;HLA-B*0858;HLA-B*0848;HLA-B*0849;HLA-B*0860;HLA-B*0855;HLA-B*9580;HLA-B*0801;HLA-B*0815;HLA-B*0818 |
| 6P2C | AAAKKKYCL | 3;9 | HLA-B*0801;HLA-B*0807;HLA-B*0809;HLA-B*0811;HLA-B*0812;HLA-B*0813;HLA-B*0814;HLA-B*0815;HLA-B*0827;HLA-B*0822;HLA-B*0820;HLA-B*0825;HLA-B*0821;HLA-B*0818;HLA-B*0828;HLA-B*0831;HLA-B*0833;HLA-B*0835;HLA-B*0837;HLA-B*0840;HLA-B*0839;HLA-B*0842;HLA-B*0843;HLA-B*0853;HLA-B*0858;HLA-B*0848;HLA-B*0849;HLA-B*0860;HLA-B*0855;HLA-B*9580;HLA-B*0801;HLA-B*0815;HLA-B*0818 |
| 6P2F | AAAKKGYCL | 3;9 | HLA-B*0801;HLA-B*0807;HLA-B*0809;HLA-B*0811;HLA-B*0812;HLA-B*0813;HLA-B*0814;HLA-B*0815;HLA-B*0827;HLA-B*0822;HLA-B*0820;HLA-B*0825;HLA-B*0821;HLA-B*0818;HLA-B*0828;HLA-B*0831;HLA-B*0833;HLA-B*0835;HLA-B*0837;HLA-B*0840;HLA-B*0839;HLA-B*0842;HLA-B*0843;HLA-B*0853;HLA-B*0858;HLA-B*0848;HLA-B*0849;HLA-B*0860;HLA-B*0855;HLA-B*9580;HLA-B*0801;HLA-B*0815;HLA-B*0818 |
| 6P2S | AAAKKKYCL | 3;9 | HLA-B*0801;HLA-B*0807;HLA-B*0809;HLA-B*0811;HLA-B*0812;HLA-B*0813;HLA-B*0814;HLA-B*0815;HLA-B*0827;HLA-B*0822;HLA-B*0820;HLA-B*0825;HLA-B*0821;HLA-B*0818;HLA-B*0828;HLA-B*0831;HLA-B*0833;HLA-B*0835;HLA-B*0837;HLA-B*0840;HLA-B*0839;HLA-B*0842;HLA-B*0843;HLA-B*0853;HLA-B*0858;HLA-B*0848;HLA-B*0849;HLA-B*0860;HLA-B*0855;HLA-B*9580;HLA-B*0801;HLA-B*0815;HLA-B*0818 |
| 6P64 | KQWLVWLFL | 2;9 | HLA-A*0206;HLA-A*0210;HLA-A*0251;HLA-A*0257;HLA-A*0279;HLA-A*0206 |
| 6PA1 | RYRPGTVAL | 2;9 | HLA-C*0702;HLA-C*0711;HLA-C*0704;HLA-C*0729;HLA-C*0741;HLA-C*0742;HLA-C*0750;HLA-C*0756;HLA-C*0766;HLA-C*0767;HLA-C*0702;HLA-C*0750;HLA-C*0766 |
| 6PAG | RYRPGTVAL | 2;9 | HLA-C*0702;HLA-C*0711;HLA-C*0704;HLA-C*0729;HLA-C*0741;HLA-C*0742;HLA-C*0750;HLA-C*0756;HLA-C*0766;HLA-C*0767;HLA-C*0702;HLA-C*0750;HLA-C*0766 |
| 6PTB | ILNAMIAKI | 2;9 | HLA-A*0201;HLA-A*0203;HLA-A*0204;HLA-A*0207;HLA-A*0209;HLA-A*0212;HLA-A*0213;HLA-A*0216;HLA-A*0217;HLA-A*0218;HLA-A*0222;HLA-A*0224;HLA-A*0226;HLA-A*0233;HLA-A*0266;HLA-A*0289;HLA-A*0268;HLA-A*0265;HLA-A*0277;HLA-A*0260;HLA-A*9232;HLA-A*9234;HLA-A*9235;HLA-A*0201;HLA-A*0209;HLA-A*0266;HLA-A*0289;HLA-A*9232;HLA-A*9234 |
| 6PTE | ILNAMITKI | 2;9 | HLA-A*0201;HLA-A*0203;HLA-A*0204;HLA-A*0207;HLA-A*0209;HLA-A*0212;HLA-A*0213;HLA-A*0216;HLA-A*0217;HLA-A*0218;HLA-A*0222;HLA-A*0224;HLA-A*0226;HLA-A*0233;HLA-A*0266;HLA-A*0289;HLA-A*0268;HLA-A*0265;HLA-A*0277;HLA-A*0260;HLA-A*9232;HLA-A*9234;HLA-A*9235;HLA-A*0201;HLA-A*0209;HLA-A*0266;HLA-A*0289;HLA-A*9232;HLA-A*9234 |
| 6PYJ | LRNQSVFNF | 2;9 | HLA-B*2705;HLA-B*2707;HLA-B*2709;HLA-B*2713;HLA-B*2714;HLA-B*2732;HLA-B*2735;HLA-B*2738;HLA-B*2747;HLA-B*2705;HLA-B*2713 |
| 6PYL | KRWIILGLNK | 2;10 | HLA-B*2703;HLA-B*2703 |
| 6PYV | LRNQSVFNF | 2;9 | HLA-B*2703;HLA-B*2703 |
| 6PYW | LRNQSVFNF | 2;9 | HLA-B*2705;HLA-B*2707;HLA-B*2709;HLA-B*2710;HLA-B*2713;HLA-B*2714;HLA-B*2719;HLA-B*2728;HLA-B*2727;HLA-B*2732;HLA-B*2735;HLA-B*2734;HLA-B*2738;HLA-B*2741;HLA-B*2745;HLA-B*2743;HLA-B*2750;HLA-B*2746;HLA-B*2755;HLA-B*2756;HLA-B*2747;HLA-B*2754;HLA-B*2758;HLA-B*2760;HLA-B*2705;HLA-B*2713 |
| 6PZ5 | LRNQSVFNF | 2;9 | HLA-B*2703;HLA-B*2703 |
| 6Q3K | NLVPMVATV | 2;9 | HLA-A*0201;HLA-A*0203;HLA-A*0204;HLA-A*0207;HLA-A*0209;HLA-A*0212;HLA-A*0213;HLA-A*0216;HLA-A*0217;HLA-A*0218;HLA-A*0219;HLA-A*0222;HLA-A*0224;HLA-A*9256;HLA-A*9253;HLA-A*9257;HLA-A*9266;HLA-A*9262;HLA-A*9259;HLA-A*9268;HLA-A*9267;HLA-A*9297;HLA-A*9298;HLA-A*9299;HLA-A*0201;HLA-A*0209;HLA-A*0266;HLA-A*0289;HLA-A*0275;HLA-A*0297;HLA-A*0296;HLA-A*9221;HLA-A*9232;HLA-A*9234;HLA-A*9240;HLA-A*9299 |
| 6Q3S | SLLMWITQV | 2;9 | HLA-A*0201;HLA-A*0203;HLA-A*0204;HLA-A*0207;HLA-A*0209;HLA-A*0212;HLA-A*0213;HLA-A*0216;HLA-A*0217;HLA-A*0218;HLA-A*0219;HLA-A*0222;HLA-A*0224;HLA-A*0225;HLA-A*0226;HLA-A*0227;HLA-A*0233;HLA-A*0236;HLA-A*0237;HLA-A*0238;HLA-A*0239;HLA-A*0240;HLA-A*0249;HLA-A*0252;HLA-A*0266;HLA-A*0289;HLA-A*0271;HLA-A*0268;HLA-A*0274;HLA-A*0258;HLA-A*0280;HLA-A*0265;HLA-A*0286;HLA-A*0277;HLA-A*0260;HLA-A*0273;HLA-A*0285;HLA-A*0264;HLA-A*0275;HLA-A*0267;HLA-A*0295;HLA-A*0297;HLA-A*9201;HLA-A*0296;HLA-A*0293;HLA-A*9221;HLA-A*9231;HLA-A*9232;HLA-A*9214;HLA-A*9202;HLA-A*9210;HLA-A*9205;HLA-A*9217;HLA-A*9211;HLA-A*9204;HLA-A*9230;HLA-A*9234;HLA-A*9218;HLA-A*9247;HLA-A*9249;HLA-A*9251;HLA-A*9248;HLA-A*9256;HLA-A*9235;HLA-A*9252;HLA-A*9240;HLA-A*9253;HLA-A*9245;HLA-A*9277;HLA-A*9257;HLA-A*9271;HLA-A*9294;HLA-A*9264;HLA-A*9290;HLA-A*9266;HLA-A*9291;HLA-A*9289;HLA-A*9274;HLA-A*9262;HLA-A*9259;HLA-A*9263;HLA-A*9268;HLA-A*9288;HLA-A*9293;HLA-A*9287;HLA-A*9283;HLA-A*9267;HLA-A*9265;HLA-A*9282;HLA-A*9297;HLA-A*9298;HLA-A*9299;HLA-A*0201;HLA-A*0209;HLA-A*0266;HLA-A*0289;HLA-A*0275;HLA-A*0297;HLA-A*0296;HLA-A*9221;HLA-A*9232;HLA-A*9234;HLA-A*9240;HLA-A*9299 |
| 6R2L | SLSKILDTV | 2;9 | HLA-A*0201;HLA-A*0203;HLA-A*0204;HLA-A*0207;HLA-A*0209;HLA-A*0212;HLA-A*0213;HLA-A*0216;HLA-A*0217;HLA-A*0218;HLA-A*0222;HLA-A*0224;HLA-A*0226;HLA-A*0233;HLA-A*0266;HLA-A*0289;HLA-A*0268;HLA-A*0265;HLA-A*0277;HLA-A*0260;HLA-A*9232;HLA-A*9234;HLA-A*9235;HLA-A*0201;HLA-A*0209;HLA-A*0266;HLA-A*0289;HLA-A*9232;HLA-A*9234 |
| 6RP9 | SLLMWITQV | 2;9 | HLA-A*0201;HLA-A*0203;HLA-A*0204;HLA-A*0207;HLA-A*0209;HLA-A*0212;HLA-A*0213;HLA-A*0216;HLA-A*0217;HLA-A*0218;HLA-A*0222;HLA-A*0224;HLA-A*0226;HLA-A*0233;HLA-A*0266;HLA-A*0289;HLA-A*0268;HLA-A*0265;HLA-A*0277;HLA-A*0260;HLA-A*9232;HLA-A*9234;HLA-A*9235;HLA-A*0201;HLA-A*0209;HLA-A*0266;HLA-A*0289;HLA-A*9232;HLA-A*9234 |
| 6RPA | SLLMWITQV | 2;9 | HLA-A*0201;HLA-A*0203;HLA-A*0204;HLA-A*0207;HLA-A*0209;HLA-A*0212;HLA-A*0213;HLA-A*0216;HLA-A*0217;HLA-A*0218;HLA-A*0222;HLA-A*0224;HLA-A*0226;HLA-A*0233;HLA-A*0266;HLA-A*0289;HLA-A*0268;HLA-A*0265;HLA-A*0277;HLA-A*0260;HLA-A*9232;HLA-A*9234;HLA-A*9235;HLA-A*0201;HLA-A*0209;HLA-A*0266;HLA-A*0289;HLA-A*9232;HLA-A*9234 |
| 6RPB | SLLMWITQV | 2;9 | HLA-A*0201;HLA-A*0203;HLA-A*0204;HLA-A*0207;HLA-A*0209;HLA-A*0212;HLA-A*0213;HLA-A*0216;HLA-A*0217;HLA-A*0218;HLA-A*0222;HLA-A*0224;HLA-A*0226;HLA-A*0233;HLA-A*0266;HLA-A*0289;HLA-A*0268;HLA-A*0265;HLA-A*0277;HLA-A*0260;HLA-A*9232;HLA-A*9234;HLA-A*9235;HLA-A*0201;HLA-A*0209;HLA-A*0266;HLA-A*0289;HLA-A*9232;HLA-A*9234 |
| 6RSY | RMFPNAPYL | 2;9 | HLA-A*0201;HLA-A*0203;HLA-A*0204;HLA-A*0207;HLA-A*0209;HLA-A*0212;HLA-A*0213;HLA-A*0216;HLA-A*0217;HLA-A*0218;HLA-A*0222;HLA-A*0224;HLA-A*0226;HLA-A*0233;HLA-A*0266;HLA-A*0289;HLA-A*0268;HLA-A*0265;HLA-A*0277;HLA-A*0260;HLA-A*9232;HLA-A*9234;HLA-A*9235;HLA-A*0201;HLA-A*0209;HLA-A*0266;HLA-A*0289;HLA-A*9232;HLA-A*9234 |
| 6SS7 | LLWAGPMAV | 2;9 | HLA-A*0201;HLA-A*0203;HLA-A*0204;HLA-A*0207;HLA-A*0209;HLA-A*0212;HLA-A*0213;HLA-A*0216;HLA-A*0217;HLA-A*0218;HLA-A*0222;HLA-A*0224;HLA-A*0226;HLA-A*0233;HLA-A*0266;HLA-A*0289;HLA-A*0268;HLA-A*0265;HLA-A*0277;HLA-A*0260;HLA-A*9232;HLA-A*9234;HLA-A*9235;HLA-A*0201;HLA-A*0209;HLA-A*0266;HLA-A*0289;HLA-A*9232;HLA-A*9234 |
| 6SS8 | LLWNGPIAV | 2;9 | HLA-A*0201;HLA-A*0203;HLA-A*0204;HLA-A*0207;HLA-A*0209;HLA-A*0212;HLA-A*0213;HLA-A*0216;HLA-A*0217;HLA-A*0218;HLA-A*0222;HLA-A*0224;HLA-A*0226;HLA-A*0233;HLA-A*0266;HLA-A*0289;HLA-A*0268;HLA-A*0265;HLA-A*0277;HLA-A*0260;HLA-A*9232;HLA-A*9234;HLA-A*9235;HLA-A*0201;HLA-A*0209;HLA-A*0266;HLA-A*0289;HLA-A*9232;HLA-A*9234 |
| 6SS9 | LLWNGPMHV | 2;9 | HLA-A*0201;HLA-A*0203;HLA-A*0204;HLA-A*0207;HLA-A*0209;HLA-A*0212;HLA-A*0213;HLA-A*0216;HLA-A*0217;HLA-A*0218;HLA-A*0222;HLA-A*0224;HLA-A*0226;HLA-A*0233;HLA-A*0266;HLA-A*0289;HLA-A*0268;HLA-A*0265;HLA-A*0277;HLA-A*0260;HLA-A*9232;HLA-A*9234;HLA-A*9235;HLA-A*0201;HLA-A*0209;HLA-A*0266;HLA-A*0289;HLA-A*9232;HLA-A*9234 |
| 6SSA | LLWNGPMQV | 2;9 | HLA-A*0201;HLA-A*0203;HLA-A*0204;HLA-A*0207;HLA-A*0209;HLA-A*0212;HLA-A*0213;HLA-A*0216;HLA-A*0217;HLA-A*0218;HLA-A*0222;HLA-A*0224;HLA-A*0226;HLA-A*0233;HLA-A*0266;HLA-A*0289;HLA-A*0268;HLA-A*0265;HLA-A*0277;HLA-A*0260;HLA-A*9232;HLA-A*9234;HLA-A*9235;HLA-A*0201;HLA-A*0209;HLA-A*0266;HLA-A*0289;HLA-A*9232;HLA-A*9234 |
| 6TMO | EAAGIGILTV | 2;10 | HLA-A*0201;HLA-A*0203;HLA-A*0204;HLA-A*0207;HLA-A*0209;HLA-A*0212;HLA-A*0213;HLA-A*0216;HLA-A*0217;HLA-A*0218;HLA-A*0222;HLA-A*0224;HLA-A*0226;HLA-A*0233;HLA-A*0266;HLA-A*0289;HLA-A*0268;HLA-A*0265;HLA-A*0277;HLA-A*0260;HLA-A*9232;HLA-A*9234;HLA-A*9235;HLA-A*0201;HLA-A*0209;HLA-A*0266;HLA-A*0289;HLA-A*9232;HLA-A*9234 |
| 6TRN | AVYDGREHTV | 2;10 | HLA-A*0201;HLA-A*0203;HLA-A*0204;HLA-A*0207;HLA-A*0209;HLA-A*0212;HLA-A*0213;HLA-A*0216;HLA-A*0217;HLA-A*0218;HLA-A*0222;HLA-A*0224;HLA-A*0226;HLA-A*0233;HLA-A*0266;HLA-A*0289;HLA-A*0268;HLA-A*0265;HLA-A*0277;HLA-A*0260;HLA-A*9232;HLA-A*9234;HLA-A*9235;HLA-A*0201;HLA-A*0209;HLA-A*0266;HLA-A*0289;HLA-A*9232;HLA-A*9234 |
| 6TRO | GVYDGREHTV | 2;10 | HLA-A*0201;HLA-A*0203;HLA-A*0204;HLA-A*0207;HLA-A*0209;HLA-A*0212;HLA-A*0213;HLA-A*0216;HLA-A*0217;HLA-A*0218;HLA-A*0222;HLA-A*0224;HLA-A*0226;HLA-A*0233;HLA-A*0266;HLA-A*0289;HLA-A*0268;HLA-A*0265;HLA-A*0277;HLA-A*0260;HLA-A*9232;HLA-A*9234;HLA-A*9235;HLA-A*0201;HLA-A*0209;HLA-A*0266;HLA-A*0289;HLA-A*9232;HLA-A*9234 |
| 6UJO | KQWLVWLFL | 2;9 | HLA-A*0206;HLA-A*0210;HLA-A*0251;HLA-A*0257;HLA-A*0279;HLA-A*0206 |
| 6UJQ | KQWLVWLLL | 2;9 | HLA-A*0206;HLA-A*0210;HLA-A*0251;HLA-A*0257;HLA-A*0279;HLA-A*0206 |
| 6UK2 | KQWLVWLLL | 2;9 | HLA-A*0206;HLA-A*0210;HLA-A*0251;HLA-A*0257;HLA-A*0279;HLA-A*0206 |
| 6UK4 | KQWLVWLFL | 2;9 | HLA-A*0206;HLA-A*0210;HLA-A*0251;HLA-A*0257;HLA-A*0279;HLA-A*0206 |
| 6ULI | GADGVGKSA | 2;9 | HLA-C*0801;HLA-C*0802;HLA-C*0803;HLA-C*0809;HLA-C*0815;HLA-C*0824;HLA-C*0822;HLA-C*0827;HLA-C*0820;HLA-C*0831;HLA-C*0802 |
| 6ULK | GADGVGKSAL | 2;10 | HLA-C*0801;HLA-C*0802;HLA-C*0803;HLA-C*0809;HLA-C*0815;HLA-C*0824;HLA-C*0822;HLA-C*0827;HLA-C*0820;HLA-C*0831;HLA-C*0802 |
| 6ULN | GADGVGKSA | 2;9 | HLA-C*0801;HLA-C*0802;HLA-C*0803;HLA-C*0809;HLA-C*0815;HLA-C*0824;HLA-C*0822;HLA-C*0827;HLA-C*0820;HLA-C*0831;HLA-C*0802 |
| 6ULR | GADGVGKSA | 2;9 | HLA-C*0801;HLA-C*0802;HLA-C*0803;HLA-C*0809;HLA-C*0815;HLA-C*0824;HLA-C*0822;HLA-C*0827;HLA-C*0820;HLA-C*0831;HLA-C*0802 |
| 6UON | GADGVGKSAL | 2;10 | HLA-C*0801;HLA-C*0802;HLA-C*0803;HLA-C*0809;HLA-C*0815;HLA-C*0824;HLA-C*0822;HLA-C*0827;HLA-C*0820;HLA-C*0831;HLA-C*0802 |
| 6UZ1 | LLFGYPVYV | 2;9 | HLA-A*0201;HLA-A*0203;HLA-A*0204;HLA-A*0207;HLA-A*0209;HLA-A*0212;HLA-A*0213;HLA-A*0216;HLA-A*0217;HLA-A*0218;HLA-A*0222;HLA-A*0224;HLA-A*0226;HLA-A*0233;HLA-A*0266;HLA-A*0289;HLA-A*0268;HLA-A*0265;HLA-A*0277;HLA-A*0260;HLA-A*9232;HLA-A*9234;HLA-A*9235;HLA-A*0201;HLA-A*0209;HLA-A*0266;HLA-A*0289;HLA-A*9232;HLA-A*9234 |
| 6V2O | ASLNLPAVSW | 2;10 | HLA-C*1510 |
| 6V2P | ASLNLPAVSW | 2;10 | HLA-C*1510 |
| 6V2Q | LSSPVTKSF | 2;9 | HLA-C*1510 |
| 6V3J | LSSPVTKSF | 2;9 | HLA-C*1510 |
| 6VMX | RPPIFIRRL | 2;9 | HLA-B*0702;HLA-B*0704;HLA-B*0705;HLA-B*0706;HLA-B*0707;HLA-B*0709;HLA-B*0714;HLA-B*0718;HLA-B*4201;HLA-B*6701;HLA-B*8101;HLA-B*0742;HLA-B*0744;HLA-B*4205;HLA-B*0733;HLA-B*4208;HLA-B*8102;HLA-B*0759;HLA-B*0761;HLA-B*0777;HLA-B*0702;HLA-B*0744;HLA-B*0759;HLA-B*0761 |
| 6VR1 | HMTEVVRRC | 2;9 | HLA-A*0201;HLA-A*0203;HLA-A*0204;HLA-A*0207;HLA-A*0209;HLA-A*0212;HLA-A*0213;HLA-A*0216;HLA-A*0217;HLA-A*0218;HLA-A*0222;HLA-A*0224;HLA-A*0226;HLA-A*0233;HLA-A*0266;HLA-A*0289;HLA-A*0268;HLA-A*0265;HLA-A*0277;HLA-A*0260;HLA-A*9232;HLA-A*9234;HLA-A*9235;HLA-A*0201;HLA-A*0209;HLA-A*0266;HLA-A*0289;HLA-A*9232;HLA-A*9234 |
| 6VR5 | HMTEVVRHC | 2;9 | HLA-A*0201;HLA-A*0203;HLA-A*0204;HLA-A*0207;HLA-A*0209;HLA-A*0212;HLA-A*0213;HLA-A*0216;HLA-A*0217;HLA-A*0218;HLA-A*0222;HLA-A*0224;HLA-A*0226;HLA-A*0233;HLA-A*0266;HLA-A*0289;HLA-A*0268;HLA-A*0265;HLA-A*0277;HLA-A*0260;HLA-A*9232;HLA-A*9234;HLA-A*9235;HLA-A*0201;HLA-A*0209;HLA-A*0266;HLA-A*0289;HLA-A*9232;HLA-A*9234 |
| 6VRN | HMTEVVRHC | 2;9 | HLA-A*0201;HLA-A*0203;HLA-A*0204;HLA-A*0207;HLA-A*0209;HLA-A*0212;HLA-A*0213;HLA-A*0216;HLA-A*0217;HLA-A*0218;HLA-A*0222;HLA-A*0224;HLA-A*0226;HLA-A*0233;HLA-A*0266;HLA-A*0289;HLA-A*0268;HLA-A*0265;HLA-A*0277;HLA-A*0260;HLA-A*9232;HLA-A*9234;HLA-A*9235;HLA-A*0201;HLA-A*0209;HLA-A*0266;HLA-A*0289;HLA-A*9232;HLA-A*9234 |
| 6WZY | SGPDNGAVAV | 2;10 | H2-D1b;H2-D1b |
| 6X00 | SGPDNGAVAVL | 2;11 | H2-D1b;H2-D1b |
| 6Y26 | GRLNAPIKV | 2;9 | HLA-B*2705;HLA-B*2707;HLA-B*2709;HLA-B*2713;HLA-B*2714;HLA-B*2732;HLA-B*2735;HLA-B*2738;HLA-B*2747;HLA-B*2705;HLA-B*2713 |
| 6Y28 | GRLNEPIKV | 2;9 | HLA-B*2705;HLA-B*2707;HLA-B*2709;HLA-B*2713;HLA-B*2714;HLA-B*2732;HLA-B*2735;HLA-B*2738;HLA-B*2747;HLA-B*2705;HLA-B*2713 |
| 6Y29 | GRLNEPIKV | 2;9 | HLA-B*2705;HLA-B*2707;HLA-B*2709;HLA-B*2713;HLA-B*2714;HLA-B*2732;HLA-B*2735;HLA-B*2738;HLA-B*2747;HLA-B*2709 |
| 6Y2A | GRLNQPIKV | 2;9 | HLA-B*2705;HLA-B*2707;HLA-B*2709;HLA-B*2713;HLA-B*2714;HLA-B*2732;HLA-B*2735;HLA-B*2738;HLA-B*2747;HLA-B*2705;HLA-B*2713 |
| 6Y2B | GRLNQPIKV | 2;9 | HLA-B*2705;HLA-B*2707;HLA-B*2709;HLA-B*2713;HLA-B*2714;HLA-B*2732;HLA-B*2735;HLA-B*2738;HLA-B*2747;HLA-B*2709 |
| 7K80 | RYPLTFGW | 2;8 | HLA-A*2301;HLA-A*2402;HLA-A*2403;HLA-A*2410;HLA-A*2422;HLA-A*2426;HLA-A*2427;HLA-A*2454;HLA-A*2453;HLA-A*2451;HLA-A*2479;HLA-A*2317;HLA-A*2402;HLA-A*2479 |
| 7K81 | RYPLTFGW | 2;8 | HLA-A*2301;HLA-A*2402;HLA-A*2403;HLA-A*2410;HLA-A*2422;HLA-A*2426;HLA-A*2427;HLA-A*2454;HLA-A*2453;HLA-A*2451;HLA-A*2479;HLA-A*2317;HLA-A*2402;HLA-A*2479 |
