# Supplementary material for: PANDORA: A Fast, Anchor-Restrained Modelling Protocol for Peptide: MHC Complexes
Source: Front Immunol. 2022 May 10;13:878762. doi: 10.3389/fimmu.2022.878762 (PMC9127323; doi:10.3389/fimmu.2022.878762)
Supplement: Supplementary file 3 [file Table_2.docx]

**Supplementary Table 2. L-RMSD values of PANDORA's experments.** Values used for Figures 2, 3, 4 and Supplementary Figures 3, 7 and 8 are listed.

| PDB_ID | Best CA L-RMSD | Best Backbone L-RMSD | Best Full-Atom L-RMSD | Top molpdf CA L-RMSD | Top molpdf Backbone L-RMSD | Top molpdf Backbone + CB L-RMSD | Top molpdf Full-Atom L-RMSD | Top molpdf Backbone L-RMSD canonical ancors | Best BackBone L-RMSD increased sampling |
| --- | --- | --- | --- | --- | --- | --- | --- | --- | --- |
| 1A1N | 1.185 | 1.126 | 2.639 | 1.185 | 1.126 | 1.174 | 2.707 | 1.018 | 1.097 |
| 1A1M | 1.176 | 1.454 | 2.627 | 2.449 | 2.419 | 2.8 | 4.335 | 2.505 | 1.454 |
| 1AGE | 0.223 | 0.253 | 1.043 | 0.232 | 0.322 | 0.351 | 1.78 | 0.248 | 0.253 |
| 1A9B | 0.573 | 0.605 | 1.152 | 0.573 | 0.605 | 0.625 | 1.152 | 0.596 | 0.605 |
| 1A1O | 0.854 | 0.876 | 2.466 | 1.157 | 1.284 | 1.537 | 3.443 | 1.356 | 0.876 |
| 1AGD | 0.497 | 0.501 | 1.189 | 0.504 | 0.522 | 0.565 | 1.699 | 0.515 | 0.485 |
| 1AGB | 0.445 | 0.509 | 1.01 | 0.615 | 0.644 | 0.685 | 1.328 | 0.75 | 0.509 |
| 1A9E | 0.55 | 0.545 | 0.902 | 0.55 | 0.545 | 0.563 | 0.902 | 0.549 | 0.542 |
| 1AGC | 0.18 | 0.284 | 0.744 | 0.18 | 0.284 | 0.287 | 0.744 | 0.359 | 0.284 |
| 1AKJ | 0.297 | 0.333 | 1.048 | 0.297 | 0.333 | 0.336 | 1.048 | 0.315 | 0.333 |
| 1CG9 | 0.613 | 0.602 | 1.048 | 0.784 | 0.77 | 0.795 | 1.37 | 0.784 | 0.602 |
| 1AGF | 0.447 | 0.467 | 0.5 | 0.526 | 0.526 | 0.626 | 0.553 | 0.44 | 0.324 |
| 1AO7 | 0.392 | 0.804 | 1.007 | 0.392 | 0.804 | 0.774 | 1.007 | 0.443 | 0.804 |
| 1B0G | 0.292 | 0.326 | 0.516 | 0.292 | 0.326 | 0.353 | 0.516 | 0.321 | 0.326 |
| 1BII | 0.744 | 0.906 | 1.54 | 0.744 | 0.906 | 0.936 | 1.54 | 0.909 | 0.906 |
| 1CE6 | 0.537 | 0.61 | 0.969 | 0.578 | 0.633 | 0.731 | 0.972 | 0.581 | 0.574 |
| 1BD2 | 0.352 | 0.364 | 0.741 | 0.352 | 0.364 | 0.396 | 0.741 | 0.329 | 0.364 |
| 1DUZ | 0.542 | 0.729 | 1.747 | 1.197 | 1.169 | 1.38 | 1.747 | 1.181 | 0.638 |
| 1DDH | 0.675 | 0.888 | 1.395 | 0.675 | 0.888 | 0.87 | 1.395 | 0.89 | 0.888 |
| 1BQH | 0.748 | 0.745 | 1.179 | 0.754 | 0.782 | 0.761 | 1.179 | 0.784 | 0.745 |
| 1EEZ | 0.69 | 0.735 | 1.535 | 1.239 | 1.181 | 1.245 | 1.689 | 1.726 | 0.664 |
| 1DUY | 0.617 | 0.78 | 1.832 | 1.293 | 1.297 | 1.642 | 3.237 | 2.368 | 0.719 |
| 1E27 | 1.589 | 1.542 | 2.914 | 2.118 | 2.104 | 2.359 | 3.562 | 1.723 | 1.47 |
| 1E28 | 0.88 | 1.132 | 1.929 | 0.88 | 1.132 | 1.207 | 1.929 | 1.132 | 1.132 |
| 1EFX | 0.872 | 0.819 | 1.588 | 0.998 | 0.957 | 1.096 | 1.637 | 1.478 | 0.776 |
| 1BZ9 | 0.833 | 0.832 | 1.976 | 1.664 | 1.617 | 1.962 | 3.451 | 1.495 | 0.765 |
| 1ED3 | 3.595 | 3.395 | 4.032 | 4.375 | 4.148 | 4.46 | 6.018 | 2.765 | 2.636 |
| 1EEY | 0.827 | 0.869 | 1.168 | 0.83 | 0.884 | 0.894 | 1.184 | 1.078 | 0.786 |
| 1FFN | 0.388 | 0.447 | 1.997 | 0.388 | 0.447 | 0.476 | 2.429 | 0.478 | 0.447 |
| 1FFP | 0.357 | 0.53 | 1.614 | 0.407 | 0.593 | 0.616 | 2.651 | 0.606 | 0.413 |
| 1FFO | 0.426 | 0.483 | 2.077 | 0.426 | 0.671 | 0.668 | 2.573 | 0.609 | 0.466 |
| 1FG2 | 0.561 | 0.717 | 1.821 | 0.608 | 0.745 | 0.82 | 2.184 | 0.701 | 0.668 |
| 1FO0 | 0.393 | 0.574 | 1.032 | 0.393 | 0.574 | 0.55 | 1.032 | 0.582 | 0.574 |
| 1FZJ | 0.771 | 0.771 | 1.074 | 0.771 | 0.771 | 0.742 | 1.074 | 0.783 | 0.771 |
| 1FZK | 0.535 | 0.752 | 0.871 | 0.716 | 0.755 | 0.758 | 0.871 | 0.751 | 0.752 |
| 1FZM | 0.518 | 0.591 | 0.957 | 0.518 | 0.591 | 0.559 | 0.957 | 0.605 | 0.591 |
| 1FZO | 0.654 | 0.671 | 0.96 | 0.671 | 0.681 | 0.697 | 0.96 | 0.687 | 0.671 |
| 1G6R | 0.765 | 1.022 | 1.869 | 0.804 | 1.028 | 0.99 | 2.065 | 1.047 | 0.998 |
| 1G7P | 0.343 | 0.544 | 1.098 | 0.343 | 0.544 | 0.531 | 1.098 | 0.515 | 0.544 |
| 1G7Q | 0.345 | 0.35 | 0.933 | 0.471 | 0.539 | 0.625 | 2.334 | 0.574 | 0.335 |
| 1HHG | 1.356 | 1.306 | 2.201 | 1.505 | 1.505 | 1.764 | 2.201 | 1.448 | 1.26 |
| 1HHH | 1.01 | 1.225 | 2.268 | 2.492 | 2.415 | 2.619 | 3.973 | 1.786 | 1.052 |
| 1HHI | 0.406 | 0.46 | 0.804 | 0.406 | 0.46 | 0.457 | 0.886 | 0.368 | 0.46 |
| 1HHJ | 0.281 | 0.304 | 0.653 | 0.281 | 0.304 | 0.309 | 0.653 | 0.313 | 0.304 |
| 1HHK | 0.537 | 0.733 | 1.714 | 1.18 | 1.151 | 1.366 | 1.714 | 1.159 | 0.647 |
| 1HOC | 0.451 | 0.537 | 1.383 | 0.539 | 0.537 | 0.618 | 1.383 | 0.523 | 0.402 |
| 1HSA | 0.717 | 0.655 | 0.892 | 0.756 | 0.675 | 0.844 | 0.94 | 1.882 | 0.536 |
| 1I1F | 0.51 | 0.965 | 1.38 | 0.684 | 1.041 | 1.041 | 2.083 | 1.225 | 0.901 |
| 1I1Y | 0.576 | 0.775 | 1.434 | 0.839 | 1.055 | 1.079 | 2.101 | 1.631 | 0.775 |
| 1I4F | 0.421 | 0.466 | 1.151 | 0.421 | 0.466 | 0.488 | 1.151 | 0.476 | 0.466 |
| 1I7R | 0.762 | 0.827 | 2.508 | 1.521 | 1.588 | 1.823 | 3.78 | 1.53 | 0.781 |
| 1I7T | 0.589 | 0.666 | 1.387 | 1.478 | 1.426 | 1.701 | 3.038 | 1.513 | 0.522 |
| 1I7U | 0.463 | 0.554 | 0.886 | 1.184 | 1.237 | 1.407 | 3.081 | 1.329 | 0.524 |
| 1IM3 | 0.539 | 0.745 | 1.794 | 1.308 | 1.254 | 1.468 | 1.794 | 1.263 | 0.703 |
| 1IM9 | 0.418 | 0.541 | 1.14 | 0.418 | 0.541 | 0.526 | 1.14 | 0.533 | 0.509 |
| 1INQ | 0.501 | 0.552 | 2.088 | 0.608 | 0.637 | 0.645 | 2.384 | 0.57 | 0.527 |
| 1JF1 | 0.39 | 0.537 | 0.867 | 0.39 | 0.537 | 0.583 | 0.867 | 0.529 | 0.537 |
| 1JGD | 1.82 | 1.836 | 2.988 | 2.249 | 2.313 | 2.435 | 3.004 | 1.427 | 1.764 |
| 1JGE | 0.525 | 0.522 | 1.233 | 0.634 | 0.865 | 0.926 | 1.395 | 1.719 | 0.522 |
| 1JHT | 0.294 | 0.31 | 0.641 | 0.294 | 0.31 | 0.339 | 0.641 | 0.32 | 0.31 |
| 1JPF | 0.97 | 1.268 | 2.329 | 1.123 | 1.485 | 1.479 | 2.443 | 1.284 | 0.836 |
| 1JPG | 0.983 | 0.947 | 1.753 | 1.025 | 0.947 | 1.046 | 1.753 | 0.849 | 0.903 |
| 1JTR | 0.607 | 0.702 | 2.005 | 0.781 | 0.922 | 0.905 | 2.181 | 0.904 | 0.613 |
| 1JUF | 0.537 | 0.559 | 1.892 | 0.56 | 0.559 | 0.57 | 2.47 | 1.069 | 0.486 |
| 1K5N | 0.563 | 0.518 | 1.356 | 0.723 | 0.898 | 0.954 | 1.396 | 1.841 | 0.518 |
| 1K8D | 0.964 | 1.167 | 2.546 | 1.984 | 1.886 | 2.384 | 4.839 | 2.004 | 1.069 |
| 1KJ2 | 0.276 | 0.351 | 0.726 | 0.276 | 0.351 | 0.359 | 0.78 | 0.333 | 0.351 |
| 1KJ3 | 0.212 | 0.221 | 0.606 | 0.212 | 0.221 | 0.274 | 0.606 | 0.25 | 0.221 |
| 1KJM | 1.227 | 1.266 | 2.093 | 1.451 | 1.417 | 1.605 | 2.371 | 1.754 | 1.22 |
| 1KJV | 1.35 | 1.277 | 2.056 | 1.904 | 1.894 | 2.052 | 3.014 | 1.968 | 1.161 |
| 1KPR | 0.32 | 0.335 | 0.856 | 0.32 | 0.335 | 0.382 | 0.876 | 0.353 | 0.335 |
| 1KPU | 0.642 | 0.67 | 1.013 | 0.642 | 0.67 | 0.639 | 1.013 | 0.687 | 0.67 |
| 1KPV | 0.472 | 0.664 | 0.976 | 0.472 | 0.687 | 0.654 | 0.976 | 0.699 | 0.664 |
| 1KTL | 0.609 | 0.648 | 1.172 | 0.662 | 0.674 | 0.725 | 1.264 | 0.808 | 0.577 |
| 1L6Q | 2.338 | 2.472 | 2.388 | 2.769 | 2.648 | 2.908 | 2.53 | 2.657 | 2.362 |
| 1LEG | 0.47 | 0.539 | 1.326 | 0.731 | 0.883 | 0.891 | 2.307 | 0.875 | 0.482 |
| 1LEK | 0.458 | 0.638 | 1.524 | 0.705 | 0.776 | 0.799 | 1.524 | 0.758 | 0.618 |
| 1LK2 | 0.675 | 0.781 | 1.889 | 0.761 | 0.813 | 0.855 | 1.972 | 0.86 | 0.743 |
| 1LP9 | 0.332 | 0.344 | 0.659 | 0.332 | 0.344 | 0.354 | 0.659 | 0.341 | 0.344 |
| 1M05 | 0.463 | 0.512 | 0.869 | 0.463 | 0.512 | 0.505 | 0.869 | 0.484 | 0.512 |
| 1M6O | 0.848 | 1.137 | 2.085 | 1.365 | 1.42 | 1.724 | 3.761 | 1.335 | 0.838 |
| 1MHE | 0.361 | 0.385 | 0.41 | 0.361 | 0.385 | 0.418 | 0.41 | 0.402 | 0.385 |
| 1MI5 | 0.736 | 0.756 | 1.263 | 0.748 | 0.756 | 0.817 | 1.263 | 0.788 | 0.756 |
| 1MWA | 0.07 | 0.129 | 0.426 | 0.07 | 0.129 | 0.148 | 0.426 | 0.109 | 0.129 |
| 1N2R | 0.976 | 1.065 | 2.094 | 1.225 | 1.263 | 1.519 | 3.641 | 1.153 | 0.704 |
| 1N3N | 0.664 | 0.723 | 1.222 | 0.749 | 0.723 | 0.759 | 1.222 | 0.748 | 0.684 |
| 1N59 | 0.317 | 0.481 | 0.782 | 0.326 | 0.481 | 0.483 | 0.782 | 0.45 | 0.481 |
| 1N5A | 0.471 | 0.559 | 2.002 | 0.471 | 0.559 | 0.576 | 2.347 | 0.555 | 0.559 |
| 1NAM | 0.624 | 0.561 | 0.975 | 0.624 | 0.561 | 0.546 | 0.975 | 0.576 | 0.561 |
| 1NAN | 0.248 | 0.486 | 0.797 | 0.35 | 0.528 | 0.513 | 0.797 | 0.535 | 0.486 |
| 1OF2 | 0.15 | 0.172 | 0.774 | 0.15 | 0.172 | 0.202 | 0.774 | 0.156 | 0.172 |
| 1OGA | 0.358 | 0.353 | 0.778 | 0.358 | 0.353 | 0.382 | 0.778 | 0.349 | 0.353 |
| 1OGT | 0.184 | 0.24 | 0.641 | 0.184 | 0.24 | 0.247 | 0.641 | 0.199 | 0.24 |
| 1OSZ | 0.675 | 0.729 | 1.329 | 0.722 | 0.808 | 0.775 | 1.796 | 0.743 | 0.729 |
| 1P1Z | 0.688 | 0.892 | 1.658 | 0.77 | 0.937 | 1.008 | 1.658 | 0.953 | 0.892 |
| 1P4L | 0.396 | 0.614 | 1.545 | 0.754 | 0.852 | 0.916 | 1.545 | 0.803 | 0.594 |
| 1P7Q | 0.417 | 0.547 | 0.93 | 0.417 | 0.547 | 0.533 | 0.93 | 0.563 | 0.547 |
| 1Q94 | 0.703 | 0.626 | 1.084 | 0.703 | 0.626 | 0.751 | 1.084 | 0.623 | 0.626 |
| 1QEW | 0.711 | 0.743 | 2.855 | 1.136 | 1.05 | 1.337 | 3.523 | 0.885 | 0.479 |
| 1QLF | 0.322 | 0.405 | 1.124 | 0.37 | 0.405 | 0.438 | 1.401 | 0.48 | 0.371 |
| 1QO3 | 0.454 | 0.395 | 0.892 | 0.454 | 0.395 | 0.458 | 0.892 | 0.387 | 0.395 |
| 1QQD | 0.384 | 0.549 | 0.791 | 0.384 | 0.549 | 0.55 | 0.791 | 0.539 | 0.549 |
| 1QR1 | 0.913 | 1.014 | 1.468 | 1.08 | 1.081 | 1.108 | 1.468 | 0.892 | 0.883 |
| 1QRN | 0.838 | 0.819 | 1.835 | 1.951 | 1.899 | 2.171 | 3.423 | 1.985 | 0.728 |
| 1QSE | 0.438 | 0.64 | 2.505 | 1.573 | 1.49 | 1.795 | 3.815 | 1.587 | 0.576 |
| 1QSF | 0.724 | 0.948 | 2.207 | 1.799 | 1.69 | 1.99 | 3.712 | 1.104 | 0.617 |
| 1QVO | 2.311 | 2.231 | 3.793 | 2.692 | 2.559 | 2.943 | 3.845 | 2.284 | 1.959 |
| 1RJY | 0.462 | 0.442 | 1.365 | 0.462 | 0.442 | 0.496 | 1.488 | 0.497 | 0.442 |
| 1RJZ | 0.366 | 0.391 | 1.755 | 0.366 | 0.391 | 0.42 | 1.755 | 0.393 | 0.391 |
| 1RK0 | 0.455 | 0.46 | 1.319 | 0.455 | 0.46 | 0.508 | 1.469 | 0.431 | 0.46 |
| 1RK1 | 0.408 | 0.394 | 1.477 | 0.408 | 0.394 | 0.423 | 1.477 | 0.34 | 0.394 |
| 1S7Q | 0.245 | 0.425 | 0.534 | 0.245 | 0.425 | 0.478 | 0.534 | 0.449 | 0.425 |
| 1S7R | 0.66 | 0.922 | 2.045 | 0.745 | 0.972 | 1.085 | 2.128 | 2.859 | 0.913 |
| 1S7S | 0.402 | 0.505 | 1.223 | 0.41 | 0.512 | 0.578 | 1.254 | 0.535 | 0.482 |
| 1S7T | 0.419 | 0.461 | 0.968 | 0.419 | 0.461 | 0.546 | 1.0 | 0.638 | 0.457 |
| 1S7U | 0.428 | 0.572 | 1.965 | 0.428 | 0.572 | 0.578 | 2.306 | 0.557 | 0.572 |
| 1S7V | 0.643 | 0.69 | 1.38 | 0.665 | 0.72 | 0.816 | 1.639 | 0.769 | 0.69 |
| 1S7W | 0.488 | 0.77 | 1.47 | 0.582 | 0.801 | 0.858 | 2.038 | 0.786 | 0.716 |
| 1S7X | 0.349 | 0.488 | 1.497 | 0.415 | 0.631 | 0.657 | 1.549 | 0.635 | 0.488 |
| 1S8D | 0.577 | 0.626 | 0.937 | 0.633 | 0.727 | 0.731 | 1.042 | 0.605 | 0.524 |
| 1S9W | 0.722 | 0.848 | 1.739 | 0.935 | 0.881 | 0.987 | 1.955 | 0.888 | 0.803 |
| 1S9X | 0.908 | 1.11 | 2.31 | 1.003 | 1.209 | 1.197 | 3.231 | 1.253 | 0.384 |
| 1S9Y | 0.381 | 0.395 | 1.499 | 0.689 | 0.635 | 0.69 | 1.722 | 1.243 | 0.395 |
| 1SYS | 0.758 | 1.111 | 1.269 | 1.541 | 1.515 | 1.636 | 1.983 | 1.339 | 0.959 |
| 1SYV | 0.917 | 1.151 | 2.124 | 1.308 | 1.368 | 1.624 | 3.631 | 1.287 | 0.83 |
| 1T0M | 0.339 | 0.344 | 1.018 | 0.339 | 0.344 | 0.405 | 1.018 | 0.318 | 0.344 |
| 1T0N | 0.197 | 0.22 | 1.177 | 0.197 | 0.22 | 0.287 | 1.177 | 0.174 | 0.22 |
| 1T1W | 0.843 | 0.913 | 1.389 | 0.843 | 0.913 | 0.909 | 1.389 | 0.845 | 0.905 |
| 1T1X | 0.391 | 0.458 | 1.209 | 0.646 | 0.613 | 0.733 | 1.731 | 0.485 | 0.419 |
| 1T1Y | 0.759 | 0.733 | 1.316 | 1.986 | 1.834 | 2.211 | 2.945 | 0.505 | 0.499 |
| 1T1Z | 0.458 | 0.609 | 1.403 | 2.184 | 2.015 | 2.387 | 2.826 | 2.161 | 0.583 |
| 1T20 | 0.559 | 0.587 | 1.363 | 0.69 | 0.843 | 0.818 | 1.364 | 0.842 | 0.561 |
| 1T21 | 0.366 | 0.416 | 0.992 | 0.366 | 0.416 | 0.427 | 0.992 | 0.451 | 0.416 |
| 1T22 | 0.354 | 0.414 | 0.937 | 0.354 | 0.414 | 0.418 | 0.937 | 0.406 | 0.414 |
| 1TVB | 0.546 | 0.491 | 1.81 | 1.97 | 1.726 | 2.087 | 2.77 | 0.564 | 0.461 |
| 1UXS | 0.694 | 0.727 | 2.544 | 2.219 | 2.19 | 2.44 | 4.689 | 1.691 | 0.616 |
| 1VAC | 0.652 | 0.672 | 1.62 | 0.948 | 0.98 | 0.999 | 1.62 | 0.932 | 0.656 |
| 1VAD | 0.289 | 0.476 | 0.983 | 0.289 | 0.476 | 0.453 | 0.983 | 0.48 | 0.476 |
| 1VGK | 0.76 | 0.823 | 1.417 | 0.762 | 0.823 | 0.876 | 1.477 | 0.702 | 0.68 |
| 1W0V | 0.81 | 0.805 | 1.736 | 1.37 | 1.33 | 1.713 | 2.99 | 1.384 | 0.659 |
| 1W0W | 0.866 | 0.89 | 1.752 | 1.463 | 1.431 | 1.793 | 3.016 | 1.483 | 0.685 |
| 1W72 | 0.493 | 0.541 | 0.861 | 0.548 | 0.569 | 0.539 | 0.861 | 0.57 | 0.491 |
| 1WBX | 0.715 | 1.019 | 2.035 | 1.364 | 1.325 | 1.645 | 2.229 | 1.375 | 0.757 |
| 1WBY | 0.226 | 0.259 | 2.127 | 0.226 | 0.259 | 0.325 | 2.247 | 0.244 | 0.259 |
| 1WBZ | 0.931 | 0.997 | 2.313 | 1.068 | 1.107 | 1.163 | 3.245 | 1.176 | 0.997 |
| 1X7Q | 1.15 | 1.226 | 1.557 | 1.797 | 1.715 | 2.049 | 3.07 | 1.761 | 1.101 |
| 1XH3 | 3.382 | 3.349 | 4.276 | 5.264 | 5.158 | 5.303 | 5.764 | 3.84 | 3.294 |
| 1XR9 | 0.773 | 0.964 | 1.072 | 0.91 | 1.04 | 1.025 | 1.285 | 0.915 | 0.895 |
| 1YDP | 0.39 | 0.403 | 1.171 | 0.39 | 0.403 | 0.423 | 1.171 | 0.414 | 0.403 |
| 1YN6 | 0.196 | 0.277 | 1.871 | 0.196 | 0.277 | 0.283 | 1.936 | 0.255 | 0.277 |
| 1YN7 | 0.642 | 0.699 | 1.755 | 1.186 | 1.05 | 1.215 | 2.215 | 1.161 | 0.552 |
| 1ZHB | 0.638 | 0.748 | 1.773 | 0.749 | 0.879 | 0.942 | 2.221 | 0.805 | 0.748 |
| 1ZHK | 0.736 | 0.7 | 1.183 | 0.736 | 0.7 | 0.714 | 1.183 | 0.724 | 0.7 |
| 1ZHL | 0.523 | 0.508 | 1.258 | 0.523 | 0.508 | 0.542 | 1.258 | 0.494 | 0.508 |
| 1ZSD | 1.018 | 1.058 | 1.543 | 2.731 | 2.492 | 2.684 | 3.779 | 2.481 | 0.976 |
| 1ZT1 | 1.12 | 0.998 | 1.512 | 1.265 | 1.182 | 1.257 | 1.653 | 1.125 | 0.998 |
| 1ZT7 | 1.537 | 1.575 | 3.341 | 2.596 | 2.612 | 2.875 | 4.629 | 1.464 | 1.575 |
| 1ZVS | 1.217 | 1.309 | 2.126 | 1.282 | 1.465 | 1.51 | 2.357 | 1.545 | 1.309 |
| 2A83 | 0.332 | 0.416 | 1.766 | 0.55 | 0.596 | 0.656 | 1.766 | 0.613 | 0.316 |
| 2AK4 | 0.823 | 0.773 | 0.95 | 0.823 | 0.773 | 0.82 | 0.95 | 0.736 | 0.773 |
| 2AV1 | 0.582 | 0.719 | 1.791 | 1.235 | 1.19 | 1.415 | 1.791 | 1.197 | 0.661 |
| 2AV7 | 0.62 | 0.753 | 1.787 | 1.233 | 1.193 | 1.421 | 1.787 | 1.2 | 0.681 |
| 2AXF | 1.266 | 1.421 | 1.706 | 1.266 | 1.421 | 1.59 | 1.706 | 1.918 | 1.221 |
| 2AXG | 0.656 | 0.678 | 1.409 | 2.833 | 2.511 | 2.902 | 3.813 | 0.86 | 0.64 |
| 2BCK | 0.89 | 0.9 | 1.996 | 1.573 | 1.633 | 1.724 | 3.045 | 1.545 | 0.9 |
| 2BNQ | 0.45 | 0.466 | 0.915 | 0.45 | 0.466 | 0.473 | 0.915 | 0.44 | 0.466 |
| 2BNR | 0.701 | 0.691 | 1.716 | 0.963 | 0.9 | 1.021 | 1.934 | 0.875 | 0.626 |
| 2BSR | 0.373 | 0.621 | 1.55 | 0.538 | 0.783 | 0.815 | 1.557 | 0.693 | 0.526 |
| 2BSS | 0.785 | 0.847 | 1.276 | 1.107 | 1.16 | 1.109 | 1.276 | 1.165 | 0.746 |
| 2BST | 0.691 | 0.954 | 3.193 | 0.724 | 1.016 | 1.093 | 3.319 | 2.816 | 0.868 |
| 2BVO | 0.797 | 0.767 | 1.586 | 1.208 | 1.181 | 1.302 | 1.997 | 1.184 | 0.65 |
| 2BVP | 0.902 | 0.955 | 1.483 | 1.022 | 1.15 | 1.201 | 1.484 | 1.478 | 0.955 |
| 2BVQ | 1.137 | 1.081 | 1.849 | 1.555 | 1.446 | 1.614 | 2.405 | 2.384 | 1.075 |
| 2C7U | 0.819 | 0.867 | 1.434 | 0.819 | 0.87 | 0.893 | 1.434 | 0.892 | 0.816 |
| 2CIK | 0.115 | 0.153 | 0.605 | 0.115 | 0.153 | 0.157 | 0.605 | 0.134 | 0.153 |
| 2CKB | 0.434 | 0.656 | 0.974 | 0.434 | 0.656 | 0.671 | 0.974 | 0.624 | 0.615 |
| 2CLR | 1.054 | 1.241 | 1.529 | 1.391 | 1.445 | 1.513 | 1.689 | 2.235 | 1.166 |
| 2CLV | 0.434 | 0.605 | 1.191 | 0.435 | 0.605 | 0.612 | 1.191 | 0.609 | 0.605 |
| 2CLZ | 0.33 | 0.482 | 0.91 | 0.388 | 0.515 | 0.516 | 0.945 | 0.509 | 0.462 |
| 2D31 | 0.587 | 0.58 | 1.653 | 0.814 | 0.745 | 0.834 | 2.344 | 0.769 | 0.528 |
| 2DYP | 0.479 | 0.553 | 2.441 | 0.786 | 0.729 | 0.81 | 2.441 | 0.75 | 0.501 |
| 2ESV | 0.782 | 0.887 | 1.035 | 0.796 | 0.904 | 0.878 | 1.349 | 0.965 | 0.855 |
| 2F53 | 0.719 | 0.8 | 1.754 | 1.074 | 1.054 | 1.16 | 2.017 | 1.044 | 0.746 |
| 2F54 | 0.627 | 0.692 | 1.799 | 0.856 | 0.859 | 0.955 | 1.939 | 0.846 | 0.64 |
| 2F74 | 0.463 | 0.646 | 1.743 | 0.463 | 0.646 | 0.642 | 1.982 | 0.629 | 0.646 |
| 2FO4 | 0.307 | 0.388 | 1.264 | 0.405 | 0.47 | 0.544 | 1.297 | 0.468 | 0.356 |
| 2FWO | 0.557 | 0.945 | 1.495 | 1.718 | 1.662 | 1.9 | 3.298 | 1.594 | 0.886 |
| 2FZ3 | 0.989 | 1.052 | 1.394 | 1.662 | 1.774 | 1.799 | 2.061 | 1.787 | 1.031 |
| 2GIT | 0.341 | 0.483 | 1.252 | 1.114 | 1.07 | 1.299 | 1.552 | 1.025 | 0.483 |
| 2GJ6 | 0.932 | 0.947 | 1.685 | 1.044 | 0.999 | 1.213 | 1.73 | 1.026 | 0.918 |
| 2GT9 | 0.371 | 0.416 | 0.573 | 0.371 | 0.416 | 0.409 | 0.573 | 0.385 | 0.416 |
| 2GTW | 1.869 | 1.787 | 1.814 | 1.869 | 1.868 | 1.916 | 2.809 | 2.923 | 1.143 |
| 2GTZ | 0.334 | 0.328 | 0.638 | 0.334 | 0.328 | 0.339 | 0.638 | 0.389 | 0.328 |
| 2GUO | 0.577 | 0.617 | 1.271 | 0.936 | 0.889 | 0.839 | 1.271 | 0.891 | 0.516 |
| 2H6P | 0.13 | 0.152 | 0.651 | 0.13 | 0.152 | 0.173 | 0.651 | 0.176 | 0.152 |
| 2HJK | 1.163 | 1.206 | 2.086 | 4.611 | 4.454 | 4.766 | 5.824 | 1.91 | 0.554 |
| 2HJL | 0.732 | 0.735 | 1.835 | 3.96 | 3.975 | 4.127 | 4.851 | 1.064 | 0.556 |
| 2HN7 | 1.195 | 1.279 | 2.888 | 1.204 | 1.327 | 1.503 | 2.903 | 1.814 | 1.236 |
| 2J8U | 0.359 | 0.379 | 0.621 | 0.359 | 0.379 | 0.392 | 0.621 | 0.374 | 0.379 |
| 2JCC | 0.428 | 0.427 | 0.702 | 0.428 | 0.427 | 0.442 | 0.702 | 0.422 | 0.427 |
| 2MHA | 0.84 | 0.937 | 1.528 | 0.858 | 0.937 | 1.065 | 1.528 | 0.965 | 0.937 |
| 2NW3 | 1.635 | 1.794 | 1.872 | 1.759 | 1.988 | 1.944 | 2.036 | 1.67 | 1.513 |
| 2NX5 | 1.461 | 1.724 | 2.09 | 2.763 | 2.491 | 2.664 | 3.982 | 2.517 | 1.43 |
| 2OL3 | 0.46 | 0.645 | 1.356 | 0.46 | 0.646 | 0.648 | 1.356 | 0.639 | 0.619 |
| 2P5E | 0.652 | 0.751 | 1.672 | 0.819 | 0.855 | 0.918 | 1.881 | 0.844 | 0.614 |
| 2P5W | 0.716 | 0.805 | 1.729 | 0.838 | 0.875 | 0.937 | 1.959 | 0.87 | 0.644 |
| 2PYE | 0.736 | 0.828 | 1.763 | 0.87 | 0.893 | 0.959 | 1.984 | 0.884 | 0.655 |
| 2RFX | 0.273 | 0.299 | 0.781 | 0.273 | 0.299 | 0.306 | 0.781 | 0.295 | 0.299 |
| 2UWE | 0.323 | 0.341 | 0.651 | 0.323 | 0.341 | 0.337 | 0.651 | 0.338 | 0.341 |
| 2V2W | 0.621 | 0.698 | 1.235 | 0.621 | 0.698 | 0.737 | 1.285 | 0.744 | 0.583 |
| 2V2X | 1.217 | 1.26 | 2.009 | 1.796 | 1.801 | 2.063 | 2.603 | 1.473 | 1.01 |
| 2VAA | 0.565 | 0.623 | 0.962 | 0.565 | 0.623 | 0.596 | 0.962 | 0.642 | 0.623 |
| 2VAB | 0.683 | 0.669 | 1.131 | 0.683 | 0.813 | 0.779 | 1.131 | 0.819 | 0.669 |
| 2VLJ | 0.519 | 0.509 | 0.826 | 0.519 | 0.509 | 0.523 | 0.826 | 0.51 | 0.509 |
| 2VLK | 0.469 | 0.484 | 0.864 | 0.469 | 0.484 | 0.499 | 0.864 | 0.475 | 0.484 |
| 2VLL | 0.22 | 0.264 | 0.677 | 0.22 | 0.264 | 0.28 | 0.677 | 0.264 | 0.264 |
| 2VLR | 0.533 | 0.548 | 0.891 | 0.533 | 0.548 | 0.57 | 0.891 | 0.547 | 0.548 |
| 2X4O | 0.883 | 0.833 | 1.509 | 1.423 | 1.469 | 1.771 | 2.395 | 1.459 | 0.815 |
| 2X4R | 0.507 | 0.593 | 0.927 | 0.524 | 0.593 | 0.652 | 0.942 | 0.599 | 0.593 |
| 2X4S | 0.532 | 0.82 | 1.424 | 0.997 | 0.965 | 1.134 | 1.764 | 0.978 | 0.813 |
| 2X4U | 0.461 | 0.543 | 0.901 | 0.461 | 0.543 | 0.543 | 0.901 | 0.56 | 0.543 |
| 2XFX | 2.252 | 2.131 | 4.016 | 2.701 | 2.623 | 2.95 | 4.953 | 3.237 | 1.877 |
| 2XPG | 0.702 | 0.722 | 2.189 | 0.892 | 0.896 | 1.001 | 2.52 | 0.656 | 0.722 |
| 2YEZ | 2.273 | 2.472 | 3.292 | 3.383 | 3.563 | 3.691 | 4.222 | 3.059 | 1.555 |
| 2YF5 | 0.42 | 0.543 | 0.857 | 0.42 | 0.543 | 0.585 | 0.857 | 0.568 | 0.543 |
| 2YF6 | 0.264 | 0.29 | 0.913 | 0.264 | 0.29 | 0.403 | 0.913 | 0.271 | 0.29 |
| 2YPK | 0.849 | 0.86 | 1.62 | 1.236 | 1.215 | 1.339 | 1.881 | 1.219 | 0.722 |
| 2YPL | 1.066 | 1.135 | 1.771 | 1.307 | 1.262 | 1.392 | 1.771 | 1.219 | 0.865 |
| 3AM8 | 0.79 | 0.867 | 1.226 | 0.851 | 0.909 | 0.922 | 1.249 | 0.928 | 0.804 |
| 3BEV | 3.485 | 3.552 | 4.405 | 4.788 | 4.935 | 5.013 | 4.885 | 5.073 | 2.686 |
| 3BEW | 2.011 | 2.18 | 3.231 | 3.055 | 3.032 | 3.282 | 4.331 | 1.041 | 1.155 |
| 3BGM | 0.334 | 0.389 | 0.932 | 0.334 | 0.389 | 0.45 | 0.932 | 0.381 | 0.389 |
| 3BH8 | 1.394 | 1.417 | 1.923 | 1.433 | 1.418 | 1.565 | 2.484 | 1.643 | 1.159 |
| 3BH9 | 1.094 | 1.121 | 2.003 | 1.094 | 1.121 | 1.114 | 2.112 | 1.171 | 1.027 |
| 3BHB | 1.448 | 1.471 | 2.77 | 1.984 | 1.926 | 2.321 | 4.201 | 1.568 | 1.426 |
| 3BO8 | 0.505 | 0.522 | 0.8 | 0.505 | 0.522 | 0.511 | 0.8 | 0.501 | 0.522 |
| 3BP4 | 0.649 | 0.602 | 1.104 | 0.649 | 0.602 | 0.656 | 1.104 | 0.506 | 0.516 |
| 3BP7 | 0.657 | 0.741 | 1.11 | 0.8 | 0.741 | 0.806 | 1.168 | 0.504 | 0.518 |
| 3BUY | 0.663 | 0.655 | 1.797 | 0.726 | 0.707 | 0.773 | 2.322 | 0.73 | 0.643 |
| 3BVN | 1.089 | 1.17 | 3.122 | 1.566 | 1.615 | 1.793 | 3.788 | 1.009 | 0.937 |
| 3BW9 | 2.757 | 2.709 | 3.689 | 3.533 | 3.587 | 3.708 | 4.764 | 3.471 | 2.309 |
| 3BWA | 0.992 | 1.136 | 1.671 | 0.992 | 1.192 | 1.242 | 1.832 | 1.012 | 1.114 |
| 3BXN | 0.61 | 0.707 | 1.019 | 0.708 | 0.807 | 0.826 | 1.172 | 1.038 | 0.706 |
| 3BZE | 0.443 | 0.462 | 0.82 | 0.525 | 0.54 | 0.578 | 0.867 | 0.482 | 0.462 |
| 3BZF | 0.394 | 0.476 | 0.869 | 0.394 | 0.476 | 0.478 | 1.118 | 0.449 | 0.43 |
| 3C8K | 0.437 | 0.584 | 1.476 | 0.697 | 0.744 | 0.826 | 1.476 | 0.717 | 0.566 |
| 3C9N | 0.406 | 0.564 | 1.568 | 0.702 | 0.808 | 0.823 | 2.082 | 0.862 | 0.564 |
| 3CC5 | 0.427 | 0.414 | 1.807 | 0.427 | 0.414 | 0.485 | 1.807 | 0.46 | 0.386 |
| 3CCH | 0.456 | 0.478 | 1.369 | 0.585 | 0.639 | 0.689 | 1.369 | 0.666 | 0.408 |
| 3CDG | 0.416 | 0.51 | 0.795 | 0.476 | 0.523 | 0.565 | 0.795 | 0.495 | 0.472 |
| 3CH1 | 0.421 | 0.589 | 1.174 | 0.421 | 0.589 | 0.573 | 1.174 | 0.595 | 0.589 |
| 3CII | 0.372 | 0.41 | 0.714 | 0.4 | 0.436 | 0.442 | 0.714 | 0.406 | 0.351 |
| 3CPL | 0.51 | 0.673 | 1.733 | 0.563 | 0.721 | 0.76 | 2.059 | 0.628 | 0.458 |
| 3CVH | 0.526 | 0.563 | 1.53 | 0.856 | 0.898 | 1.003 | 1.611 | 0.874 | 0.522 |
| 3CZF | 0.422 | 0.51 | 1.486 | 0.578 | 0.643 | 0.686 | 1.486 | 0.61 | 0.487 |
| 3D25 | 0.332 | 0.387 | 1.124 | 0.332 | 0.387 | 0.384 | 1.124 | 0.381 | 0.387 |
| 3D3V | 0.258 | 0.32 | 0.818 | 0.258 | 0.32 | 0.328 | 0.818 | 0.303 | 0.32 |
| 3DMM | 0.281 | 0.292 | 0.8 | 0.281 | 0.292 | 0.326 | 0.8 | 0.285 | 0.292 |
| 3DX6 | 1.05 | 1.177 | 2.587 | 1.05 | 1.177 | 1.283 | 2.587 | 1.16 | 1.089 |
| 3DX7 | 0.995 | 1.279 | 2.236 | 0.995 | 1.279 | 1.331 | 2.236 | 1.205 | 1.2 |
| 3DX8 | 1.15 | 1.315 | 2.75 | 1.15 | 1.315 | 1.483 | 2.75 | 1.27 | 1.19 |
| 3DXA | 1.032 | 1.21 | 2.435 | 1.032 | 1.218 | 1.295 | 2.435 | 1.214 | 1.21 |
| 3E6F | 0.592 | 0.82 | 1.377 | 0.592 | 0.82 | 0.847 | 1.809 | 1.193 | 0.675 |
| 3E6H | 0.985 | 1.41 | 2.273 | 2.462 | 2.473 | 2.726 | 5.475 | 1.865 | 1.231 |
| 3ECB | 0.296 | 0.424 | 0.963 | 0.296 | 0.424 | 0.423 | 0.963 | 0.43 | 0.424 |
| 3FFC | 0.676 | 0.672 | 1.206 | 0.676 | 0.672 | 0.727 | 1.206 | 0.707 | 0.672 |
| 3FOL | 0.421 | 0.457 | 1.553 | 0.576 | 0.653 | 0.661 | 2.474 | 0.467 | 0.442 |
| 3FOM | 0.43 | 0.572 | 2.226 | 0.559 | 0.679 | 0.737 | 2.765 | 0.48 | 0.521 |
| 3FON | 0.345 | 0.451 | 1.348 | 0.352 | 0.461 | 0.478 | 1.348 | 0.462 | 0.435 |
| 3FQR | 1.574 | 1.566 | 1.437 | 1.775 | 1.566 | 1.799 | 1.437 | 1.636 | 1.504 |
| 3FQT | 0.728 | 0.816 | 1.115 | 0.728 | 0.816 | 0.85 | 1.115 | 0.791 | 0.816 |
| 3FQU | 0.436 | 0.812 | 0.812 | 0.785 | 0.812 | 0.874 | 0.812 | 0.814 | 0.655 |
| 3FQW | 0.249 | 0.358 | 0.455 | 0.249 | 0.623 | 0.569 | 0.6 | 0.856 | 0.353 |
| 3FQX | 0.267 | 0.307 | 0.307 | 0.267 | 0.874 | 0.805 | 1.153 | 0.608 | 0.307 |
| 3FT3 | 0.296 | 0.364 | 0.81 | 0.296 | 0.364 | 0.37 | 0.81 | 0.367 | 0.364 |
| 3FT4 | 0.606 | 0.737 | 1.96 | 1.156 | 1.112 | 1.406 | 3.241 | 0.559 | 0.553 |
| 3FTG | 0.62 | 0.647 | 1.776 | 0.696 | 0.734 | 0.805 | 1.858 | 0.572 | 0.647 |
| 3GIV | 1.105 | 1.696 | 2.286 | 2.549 | 2.669 | 2.975 | 3.793 | 3.139 | 1.696 |
| 3GJF | 0.436 | 0.479 | 1.204 | 0.436 | 0.479 | 0.527 | 1.204 | 0.536 | 0.479 |
| 3GJG | 0.458 | 0.512 | 1.034 | 0.458 | 0.512 | 0.535 | 1.034 | 0.554 | 0.512 |
| 3GSN | 0.406 | 0.587 | 0.825 | 0.489 | 0.62 | 0.639 | 0.825 | 0.614 | 0.568 |
| 3GSO | 0.282 | 0.513 | 0.864 | 0.366 | 0.561 | 0.551 | 0.864 | 0.544 | 0.506 |
| 3GSQ | 0.277 | 0.385 | 0.683 | 0.277 | 0.431 | 0.436 | 0.683 | 0.371 | 0.353 |
| 3GSR | 0.424 | 0.619 | 0.901 | 0.522 | 0.698 | 0.699 | 1.172 | 1.162 | 0.619 |
| 3GSU | 0.313 | 0.368 | 0.426 | 0.342 | 0.379 | 0.382 | 0.426 | 0.955 | 0.263 |
| 3GSV | 0.442 | 0.584 | 0.787 | 0.619 | 0.756 | 0.736 | 0.787 | 0.811 | 0.521 |
| 3GSW | 0.475 | 0.622 | 1.347 | 1.088 | 1.334 | 1.396 | 1.508 | 1.312 | 0.544 |
| 3H7B | 0.374 | 0.477 | 0.768 | 0.374 | 0.477 | 0.482 | 0.768 | 0.455 | 0.477 |
| 3H9H | 0.297 | 0.361 | 0.876 | 0.297 | 0.361 | 0.339 | 0.876 | 0.354 | 0.361 |
| 3H9S | 0.422 | 0.622 | 2.216 | 0.422 | 0.622 | 0.608 | 2.216 | 0.632 | 0.622 |
| 3HAE | 0.456 | 0.511 | 1.034 | 0.456 | 0.511 | 0.535 | 1.034 | 0.554 | 0.511 |
| 3HG1 | 0.394 | 0.591 | 0.764 | 0.394 | 0.591 | 0.633 | 0.764 | 0.571 | 0.591 |
| 3HPJ | 0.416 | 0.464 | 1.338 | 0.416 | 0.464 | 0.49 | 1.338 | 0.4 | 0.464 |
| 3I6G | 0.664 | 0.68 | 2.255 | 1.062 | 1.185 | 1.371 | 3.859 | 1.121 | 0.576 |
| 3I6K | 1.012 | 1.086 | 1.256 | 1.149 | 1.221 | 1.216 | 2.074 | 0.987 | 0.985 |
| 3I6L | 0.914 | 1.076 | 1.77 | 1.062 | 1.182 | 1.226 | 2.295 | 1.026 | 0.98 |
| 3IXA | 0.594 | 0.641 | 1.243 | 0.594 | 0.641 | 0.645 | 1.243 | 0.639 | 0.641 |
| 3JTS | 1.418 | 1.46 | 2.284 | 1.951 | 1.823 | 2.143 | 3.671 | 1.801 | 1.188 |
| 3JTT | 0.916 | 1.098 | 2.014 | 1.167 | 1.271 | 1.308 | 2.298 | 1.156 | 0.802 |
| 3KLA | 0.602 | 0.679 | 1.984 | 1.088 | 1.035 | 1.212 | 2.34 | 1.187 | 0.679 |
| 3KPL | 0.36 | 0.571 | 1.183 | 0.36 | 0.571 | 0.574 | 1.183 | 0.576 | 0.571 |
| 3KPM | 0.813 | 0.888 | 1.639 | 0.813 | 0.888 | 0.922 | 1.825 | 1.563 | 0.725 |
| 3KPN | 0.429 | 0.616 | 1.232 | 0.429 | 0.616 | 0.62 | 1.232 | 0.62 | 0.556 |
| 3KPO | 0.812 | 0.916 | 2.04 | 0.812 | 0.916 | 0.929 | 2.04 | 0.475 | 0.626 |
| 3KPP | 0.413 | 0.432 | 1.137 | 0.413 | 0.432 | 0.481 | 1.137 | 0.436 | 0.399 |
| 3KPQ | 0.857 | 0.873 | 1.854 | 1.614 | 1.662 | 1.922 | 3.6 | 1.508 | 0.816 |
| 3KPS | 0.443 | 0.617 | 1.182 | 0.443 | 0.617 | 0.577 | 1.182 | 0.608 | 0.617 |
| 3KXF | 1.4 | 1.282 | 2.132 | 1.577 | 1.397 | 1.671 | 2.979 | 2.342 | 0.906 |
| 3KYN | 0.518 | 0.539 | 0.923 | 0.518 | 0.539 | 0.572 | 1.02 | 0.477 | 0.434 |
| 3KYO | 0.558 | 0.618 | 1.357 | 1.912 | 1.856 | 2.136 | 4.014 | 0.513 | 0.56 |
| 3L3D | 0.415 | 0.478 | 1.381 | 0.965 | 0.958 | 1.204 | 2.189 | 1.133 | 0.478 |
| 3L3G | 0.937 | 1.052 | 1.984 | 1.855 | 1.868 | 2.097 | 1.984 | 1.144 | 0.851 |
| 3L3H | 0.595 | 0.81 | 1.804 | 1.033 | 1.087 | 1.214 | 2.339 | 0.649 | 0.57 |
| 3L3J | 0.603 | 0.84 | 1.124 | 2.124 | 1.851 | 2.134 | 1.922 | 0.351 | 0.358 |
| 3L3K | 0.855 | 0.886 | 1.269 | 0.92 | 0.886 | 1.024 | 1.269 | 1.894 | 0.708 |
| 3LKN | 0.452 | 0.568 | 1.854 | 2.539 | 2.284 | 2.641 | 4.411 | 0.65 | 0.392 |
| 3LKO | 0.366 | 0.487 | 1.303 | 0.477 | 0.487 | 0.559 | 1.303 | 0.987 | 0.433 |
| 3LKP | 0.335 | 0.381 | 0.969 | 0.37 | 0.418 | 0.483 | 0.992 | 0.561 | 0.369 |
| 3LKQ | 0.489 | 0.571 | 1.369 | 1.51 | 1.503 | 1.786 | 3.027 | 0.537 | 0.527 |
| 3LKR | 0.364 | 0.467 | 1.864 | 2.004 | 1.969 | 2.188 | 4.14 | 0.607 | 0.385 |
| 3LKS | 0.495 | 0.436 | 1.12 | 0.495 | 0.488 | 0.6 | 1.12 | 0.712 | 0.372 |
| 3LN5 | 2.561 | 2.586 | 3.273 | 2.869 | 2.842 | 2.997 | 3.671 | 2.57 | 1.003 |
| 3LV3 | 0.556 | 0.557 | 2.569 | 0.95 | 0.915 | 0.984 | 3.493 | 1.259 | 0.509 |
| 3MGO | 0.802 | 0.919 | 1.783 | 1.213 | 1.268 | 1.306 | 1.965 | 1.199 | 0.641 |
| 3MGT | 0.83 | 1.039 | 2.241 | 1.963 | 2.142 | 2.262 | 2.943 | 2.276 | 0.949 |
| 3MR9 | 0.302 | 0.374 | 0.691 | 0.487 | 0.602 | 0.609 | 0.888 | 1.111 | 0.354 |
| 3MRB | 0.607 | 0.719 | 1.722 | 0.673 | 1.065 | 1.069 | 2.081 | 0.853 | 0.651 |
| 3MRC | 0.883 | 1.088 | 1.379 | 1.706 | 1.778 | 1.999 | 2.86 | 1.377 | 1.083 |
| 3MRD | 0.708 | 1.144 | 1.515 | 1.444 | 1.53 | 1.506 | 1.614 | 1.183 | 1.144 |
| 3MRE | 0.373 | 0.377 | 0.948 | 0.373 | 0.377 | 0.407 | 0.948 | 0.352 | 0.377 |
| 3MRF | 0.387 | 0.725 | 1.269 | 1.837 | 1.618 | 1.988 | 2.785 | 1.311 | 0.703 |
| 3MRG | 0.671 | 0.725 | 1.252 | 0.704 | 0.725 | 0.768 | 1.581 | 0.826 | 0.675 |
| 3MRH | 0.778 | 0.88 | 1.424 | 0.871 | 0.947 | 1.002 | 1.424 | 1.362 | 0.817 |
| 3MRI | 0.662 | 0.762 | 2.002 | 1.402 | 1.385 | 1.605 | 3.583 | 1.256 | 0.701 |
| 3MRJ | 0.853 | 1.039 | 2.003 | 1.121 | 1.217 | 1.269 | 2.44 | 1.229 | 0.851 |
| 3MRK | 0.745 | 0.815 | 1.662 | 0.99 | 1.039 | 1.142 | 2.401 | 0.875 | 0.62 |
| 3MRL | 0.656 | 0.696 | 1.453 | 0.885 | 0.824 | 1.075 | 3.575 | 0.548 | 0.655 |
| 3MRM | 0.759 | 0.805 | 1.061 | 0.759 | 0.805 | 0.777 | 1.061 | 0.772 | 0.663 |
| 3MRN | 0.774 | 0.896 | 1.601 | 1.756 | 1.753 | 1.883 | 2.392 | 0.975 | 0.868 |
| 3MRO | 1.628 | 1.747 | 1.872 | 2.723 | 2.786 | 2.895 | 3.839 | 2.71 | 1.573 |
| 3MRP | 0.275 | 0.279 | 0.585 | 0.275 | 0.279 | 0.28 | 0.585 | 0.27 | 0.279 |
| 3MRQ | 0.28 | 0.301 | 0.579 | 0.28 | 0.301 | 0.313 | 0.579 | 0.304 | 0.301 |
| 3MRR | 1.301 | 1.206 | 1.577 | 1.706 | 1.718 | 1.699 | 2.138 | 2.247 | 0.828 |
| 3MV7 | 0.883 | 1.258 | 2.328 | 1.636 | 1.738 | 1.753 | 2.658 | 1.68 | 1.164 |
| 3MV8 | 0.872 | 1.203 | 2.348 | 1.612 | 1.673 | 1.697 | 2.671 | 1.625 | 1.108 |
| 3MV9 | 0.92 | 1.272 | 2.37 | 1.696 | 1.791 | 1.824 | 2.732 | 1.73 | 1.198 |
| 3MYJ | 0.978 | 1.068 | 1.206 | 0.978 | 1.068 | 1.136 | 1.206 | 1.457 | 1.068 |
| 3NFJ | 0.945 | 1.102 | 1.602 | 0.945 | 1.102 | 1.126 | 1.759 | 1.12 | 1.102 |
| 3NFN | 0.839 | 1.089 | 1.628 | 0.839 | 1.089 | 1.062 | 1.628 | 1.082 | 1.089 |
| 3O4L | 0.264 | 0.292 | 0.897 | 0.264 | 0.292 | 0.3 | 0.897 | 0.311 | 0.292 |
| 3OX8 | 0.884 | 1.221 | 2.435 | 1.49 | 1.46 | 1.587 | 2.435 | 1.238 | 0.9 |
| 3OXR | 1.316 | 1.267 | 2.652 | 1.476 | 1.455 | 1.585 | 2.688 | 2.711 | 1.267 |
| 3OXS | 1.654 | 1.817 | 2.855 | 2.328 | 2.222 | 2.378 | 3.213 | 1.932 | 1.26 |
| 3P4M | 0.41 | 0.741 | 0.979 | 0.525 | 0.868 | 0.844 | 1.532 | 0.78 | 0.563 |
| 3P4N | 0.406 | 0.766 | 1.042 | 0.486 | 0.819 | 0.795 | 1.235 | 0.734 | 0.612 |
| 3P4O | 0.48 | 0.597 | 0.98 | 0.723 | 0.738 | 0.772 | 1.096 | 0.901 | 0.56 |
| 3P9L | 0.541 | 0.593 | 1.674 | 0.819 | 0.854 | 0.942 | 1.674 | 0.829 | 0.577 |
| 3P9M | 0.553 | 0.573 | 1.201 | 0.595 | 0.604 | 0.711 | 2.08 | 0.607 | 0.529 |
| 3PAB | 0.57 | 0.576 | 1.493 | 0.597 | 0.612 | 0.695 | 2.123 | 0.681 | 0.557 |
| 3PQY | 0.606 | 0.658 | 2.031 | 0.606 | 0.658 | 0.697 | 2.031 | 0.655 | 0.658 |
| 3PWJ | 0.679 | 0.609 | 0.97 | 1.035 | 0.984 | 1.013 | 1.343 | 0.48 | 0.565 |
| 3PWL | 0.566 | 0.758 | 1.325 | 0.889 | 1.054 | 1.154 | 2.336 | 1.127 | 0.758 |
| 3PWN | 0.496 | 0.587 | 1.624 | 0.76 | 0.708 | 0.808 | 1.694 | 1.376 | 0.541 |
| 3PWP | 0.779 | 1.002 | 2.165 | 0.779 | 1.002 | 1.091 | 2.363 | 1.028 | 1.002 |
| 3PWU | 1.142 | 1.382 | 1.686 | 1.397 | 1.549 | 1.762 | 2.542 | 1.51 | 1.23 |
| 3PWV | 1.345 | 1.38 | 2.009 | 1.345 | 1.38 | 1.626 | 2.367 | 1.38 | 1.231 |
| 3QDG | 0.258 | 0.291 | 1.007 | 0.258 | 0.291 | 0.334 | 1.007 | 0.283 | 0.291 |
| 3QDJ | 0.964 | 0.976 | 1.182 | 0.964 | 0.976 | 0.908 | 1.182 | 0.985 | 0.976 |
| 3QDM | 0.502 | 0.542 | 1.06 | 0.502 | 0.542 | 0.566 | 1.06 | 0.511 | 0.542 |
| 3QEQ | 1.168 | 1.198 | 1.276 | 1.168 | 1.198 | 1.127 | 1.55 | 1.203 | 1.198 |
| 3QFD | 0.302 | 0.285 | 0.963 | 0.302 | 0.285 | 0.309 | 1.005 | 0.299 | 0.285 |
| 3QFJ | 0.258 | 0.267 | 0.834 | 0.258 | 0.267 | 0.311 | 0.834 | 0.27 | 0.267 |
| 3QQ3 | 0.81 | 0.766 | 1.481 | 1.411 | 1.31 | 1.418 | 1.602 | 1.304 | 0.766 |
| 3QQ4 | 1.102 | 1.21 | 1.648 | 1.968 | 1.803 | 2.153 | 3.039 | 1.132 | 0.861 |
| 3QUK | 0.266 | 0.282 | 1.123 | 0.266 | 0.282 | 0.331 | 1.76 | 0.296 | 0.282 |
| 3QUL | 0.504 | 0.688 | 1.323 | 0.563 | 0.688 | 0.733 | 1.676 | 0.679 | 0.615 |
| 3QZW | 0.818 | 1.044 | 1.613 | 0.818 | 1.044 | 1.03 | 1.613 | 1.057 | 1.044 |
| 3REW | 0.977 | 0.94 | 1.411 | 2.27 | 2.084 | 2.313 | 3.38 | 2.027 | 0.698 |
| 3RL1 | 0.875 | 0.928 | 1.964 | 0.983 | 1.102 | 1.183 | 2.163 | 1.941 | 0.879 |
| 3RL2 | 1.972 | 1.879 | 3.066 | 2.978 | 2.787 | 3.098 | 4.482 | 2.477 | 1.709 |
| 3ROO | 0.354 | 0.457 | 0.913 | 0.357 | 0.457 | 0.546 | 0.913 | 0.469 | 0.457 |
| 3RWC | 1.002 | 1.069 | 2.562 | 2.083 | 1.96 | 2.312 | 3.297 | 1.353 | 0.955 |
| 3RWD | 1.919 | 1.923 | 3.343 | 2.237 | 2.243 | 2.389 | 4.089 | 2.01 | 1.923 |
| 3RWE | 0.728 | 0.787 | 1.927 | 0.893 | 0.941 | 0.968 | 2.17 | 1.01 | 0.721 |
| 3RWF | 0.693 | 0.698 | 1.578 | 1.417 | 1.331 | 1.546 | 2.575 | 1.54 | 0.521 |
| 3RWG | 1.158 | 1.102 | 2.078 | 1.505 | 1.498 | 1.679 | 2.361 | 1.121 | 0.974 |
| 3RWH | 1.34 | 1.273 | 2.51 | 2.09 | 2.001 | 2.382 | 3.539 | 1.689 | 1.273 |
| 3RWI | 0.917 | 0.809 | 1.098 | 0.917 | 0.809 | 0.781 | 1.18 | 2.81 | 0.794 |
| 3RWJ | 0.55 | 0.542 | 0.983 | 0.55 | 0.542 | 0.609 | 1.292 | 2.416 | 0.391 |
| 3SJV | 0.655 | 0.677 | 1.224 | 0.686 | 0.677 | 0.734 | 1.224 | 0.682 | 0.677 |
| 3SKM | 1.101 | 1.163 | 1.925 | 1.473 | 1.41 | 1.596 | 2.35 | 1.322 | 0.77 |
| 3SKO | 1.119 | 1.074 | 2.026 | 1.12 | 1.081 | 1.137 | 2.026 | 1.1 | 1.074 |
| 3SPV | 0.355 | 0.523 | 1.529 | 0.377 | 0.543 | 0.563 | 1.676 | 0.557 | 0.508 |
| 3TBS | 0.197 | 0.213 | 1.108 | 0.197 | 0.213 | 0.265 | 1.668 | 0.215 | 0.213 |
| 3TBT | 0.434 | 0.642 | 1.112 | 0.495 | 0.679 | 0.704 | 1.198 | 0.624 | 0.642 |
| 3TBV | 0.44 | 0.635 | 1.106 | 0.502 | 0.645 | 0.656 | 1.126 | 0.655 | 0.478 |
| 3TBW | 0.338 | 0.626 | 1.234 | 0.459 | 0.683 | 0.713 | 1.244 | 0.823 | 0.406 |
| 3TBY | 0.558 | 0.54 | 1.234 | 0.558 | 0.54 | 0.603 | 1.234 | 0.56 | 0.54 |
| 3TID | 0.489 | 0.558 | 1.034 | 0.503 | 0.569 | 0.706 | 1.034 | 0.66 | 0.558 |
| 3TIE | 0.36 | 0.551 | 0.725 | 0.448 | 0.607 | 0.684 | 1.116 | 0.564 | 0.519 |
| 3TO2 | 0.915 | 0.934 | 2.479 | 1.002 | 1.04 | 1.173 | 2.578 | 1.081 | 0.888 |
| 3UTQ | 0.549 | 0.595 | 0.986 | 0.549 | 0.595 | 0.624 | 0.986 | 0.562 | 0.595 |
| 3UTS | 0.512 | 0.572 | 0.971 | 0.512 | 0.572 | 0.602 | 0.971 | 0.571 | 0.572 |
| 3UTT | 0.679 | 0.702 | 1.014 | 0.679 | 0.702 | 0.817 | 1.014 | 0.706 | 0.702 |
| 3V5D | 0.7 | 0.928 | 1.923 | 0.951 | 1.14 | 1.227 | 2.009 | 1.046 | 0.928 |
| 3V5H | 0.451 | 0.5 | 1.529 | 0.568 | 0.552 | 0.586 | 1.529 | 0.383 | 0.496 |
| 3V5K | 0.511 | 0.53 | 1.684 | 0.61 | 0.647 | 0.693 | 2.064 | 0.407 | 0.486 |
| 3VCL | 2.636 | 2.567 | 3.167 | 3.105 | 3.111 | 3.345 | 5.497 | 5.11 | 2.179 |
| 3VFM | 0.467 | 0.473 | 0.968 | 0.467 | 0.473 | 0.482 | 0.968 | 0.43 | 0.473 |
| 3VFN | 0.149 | 0.164 | 0.477 | 0.149 | 0.164 | 0.169 | 0.477 | 0.151 | 0.164 |
| 3VFO | 0.158 | 0.182 | 0.6 | 0.158 | 0.182 | 0.212 | 0.6 | 0.174 | 0.182 |
| 3VFP | 0.185 | 0.209 | 0.652 | 0.185 | 0.209 | 0.226 | 0.652 | 0.193 | 0.209 |
| 3VFR | 1.052 | 1.292 | 1.967 | 1.969 | 1.694 | 2.055 | 3.556 | 2.2 | 1.069 |
| 3VFS | 1.559 | 1.513 | 2.074 | 1.613 | 1.543 | 1.739 | 2.733 | 2.72 | 1.33 |
| 3VFT | 0.572 | 0.788 | 1.163 | 2.442 | 2.471 | 2.659 | 3.968 | 3.459 | 0.784 |
| 3VFU | 0.7 | 0.812 | 1.135 | 1.091 | 1.131 | 1.142 | 1.431 | 1.289 | 0.529 |
| 3VFV | 0.628 | 0.681 | 1.311 | 0.861 | 1.119 | 1.141 | 1.657 | 1.284 | 0.581 |
| 3VFW | 1.056 | 1.069 | 1.509 | 3.061 | 2.852 | 2.926 | 3.45 | 2.522 | 1.028 |
| 3VH8 | 0.255 | 0.245 | 0.582 | 0.255 | 0.245 | 0.275 | 0.582 | 0.222 | 0.245 |
| 3VJ6 | 0.272 | 0.29 | 0.811 | 0.272 | 0.29 | 0.297 | 0.811 | 0.287 | 0.29 |
| 3VXM | 0.876 | 0.866 | 1.293 | 0.876 | 0.866 | 0.921 | 1.293 | 0.869 | 0.866 |
| 3VXN | 0.828 | 1.082 | 1.708 | 0.828 | 1.082 | 1.059 | 1.708 | 1.094 | 1.082 |
| 3VXO | 0.874 | 0.881 | 1.552 | 0.874 | 0.881 | 0.933 | 1.552 | 0.872 | 0.881 |
| 3VXP | 1.282 | 1.428 | 1.619 | 1.282 | 1.428 | 1.452 | 1.619 | 1.43 | 1.428 |
| 3VXR | 1.187 | 1.157 | 1.6 | 1.187 | 1.157 | 1.178 | 1.6 | 1.154 | 1.157 |
| 3VXS | 1.381 | 1.544 | 1.824 | 1.381 | 1.544 | 1.575 | 1.824 | 1.516 | 1.231 |
| 3VXU | 1.516 | 1.501 | 2.019 | 1.53 | 1.501 | 1.485 | 2.506 | 1.495 | 1.498 |
| 3W0W | 1.294 | 1.219 | 2.195 | 1.294 | 1.219 | 1.196 | 2.465 | 1.208 | 1.219 |
| 3W39 | 1.39 | 1.321 | 1.615 | 1.576 | 1.545 | 1.547 | 1.717 | 1.504 | 1.321 |
| 3WL9 | 1.01 | 0.923 | 1.352 | 1.192 | 1.025 | 1.097 | 1.53 | 1.158 | 0.923 |
| 3WLB | 0.776 | 0.826 | 1.391 | 1.25 | 1.187 | 1.297 | 2.135 | 1.15 | 0.819 |
| 3WS3 | 1.007 | 1.063 | 2.504 | 1.054 | 1.108 | 1.164 | 2.584 | 0.898 | 1.008 |
| 3WS6 | 0.596 | 0.708 | 1.368 | 0.629 | 0.731 | 0.737 | 1.368 | 0.653 | 0.708 |
| 3WUW | 0.334 | 0.318 | 0.591 | 0.334 | 0.318 | 0.342 | 0.591 | 0.293 | 0.318 |
| 3X11 | 0.65 | 0.635 | 1.122 | 0.809 | 0.767 | 0.904 | 1.32 | 0.842 | 0.507 |
| 3X12 | 0.398 | 0.38 | 0.593 | 0.398 | 0.38 | 0.398 | 0.593 | 0.351 | 0.38 |
| 3X13 | 0.725 | 0.709 | 1.366 | 0.725 | 0.709 | 0.768 | 1.366 | 0.736 | 0.709 |
| 3X14 | 1.134 | 1.069 | 1.645 | 1.419 | 1.582 | 1.692 | 2.763 | 1.153 | 1.069 |
| 4CVX | 1.544 | 1.616 | 2.61 | 1.863 | 1.928 | 2.17 | 2.819 | 1.419 | 1.377 |
| 4CVZ | 1.657 | 2.095 | 3.181 | 3.668 | 3.433 | 3.813 | 6.079 | 3.35 | 2.023 |
| 4CW1 | 0.583 | 0.709 | 1.783 | 0.749 | 0.818 | 0.865 | 1.783 | 0.739 | 0.666 |
| 4D0B | 0.272 | 0.279 | 0.779 | 0.272 | 0.279 | 0.396 | 0.779 | 0.278 | 0.279 |
| 4D0C | 0.417 | 0.526 | 0.852 | 0.417 | 0.526 | 0.584 | 0.852 | 0.511 | 0.526 |
| 4D0D | 0.785 | 0.856 | 1.392 | 0.915 | 0.943 | 1.038 | 1.392 | 1.153 | 0.856 |
| 4E0R | 0.367 | 0.37 | 1.244 | 0.427 | 0.412 | 0.508 | 1.426 | 0.441 | 0.35 |
| 4E5X | 0.548 | 0.687 | 1.531 | 1.066 | 1.035 | 1.234 | 1.972 | 1.033 | 0.645 |
| 4EUP | 1.092 | 1.046 | 1.399 | 1.092 | 1.046 | 1.069 | 1.414 | 1.084 | 1.046 |
| 4EUQ | 0.383 | 0.417 | 0.594 | 0.383 | 0.417 | 0.435 | 0.594 | 0.402 | 0.417 |
| 4F7M | 1.139 | 1.237 | 2.474 | 1.745 | 1.656 | 1.864 | 3.359 | 1.77 | 1.237 |
| 4F7T | 0.789 | 0.927 | 1.784 | 0.82 | 0.949 | 1.004 | 1.784 | 0.957 | 0.863 |
| 4FTV | 0.352 | 0.397 | 0.977 | 0.352 | 0.397 | 0.416 | 0.977 | 0.348 | 0.397 |
| 4G42 | 0.672 | 0.711 | 1.403 | 0.678 | 0.737 | 0.748 | 1.862 | 0.778 | 0.698 |
| 4G43 | 0.419 | 0.525 | 1.612 | 0.431 | 0.537 | 0.529 | 1.66 | 0.749 | 0.484 |
| 4G8G | 1.116 | 1.156 | 1.204 | 1.116 | 1.156 | 1.131 | 1.204 | 1.173 | 0.978 |
| 4G8I | 0.852 | 0.83 | 1.448 | 1.266 | 1.272 | 1.228 | 1.448 | 1.308 | 0.585 |
| 4G9D | 0.491 | 0.55 | 1.066 | 0.715 | 0.689 | 0.817 | 1.256 | 0.696 | 0.315 |
| 4G9F | 1.179 | 1.175 | 1.55 | 1.33 | 1.35 | 1.319 | 1.55 | 1.319 | 1.175 |
| 4GKN | 1.867 | 1.834 | 1.849 | 3.518 | 3.521 | 3.636 | 4.308 | 3.03 | 1.088 |
| 4GKS | 1.83 | 1.704 | 1.792 | 2.699 | 2.693 | 2.686 | 3.025 | 2.905 | 1.604 |
| 4HKJ | 0.498 | 0.574 | 1.533 | 0.832 | 0.931 | 0.983 | 1.533 | 0.91 | 0.54 |
| 4HS3 | 0.403 | 0.535 | 1.035 | 0.409 | 0.535 | 0.603 | 1.035 | 0.643 | 0.518 |
| 4HUU | 0.163 | 0.223 | 1.21 | 0.163 | 0.223 | 0.218 | 1.21 | 0.23 | 0.223 |
| 4HUV | 0.6 | 0.722 | 1.78 | 0.764 | 0.795 | 0.913 | 2.268 | 0.761 | 0.614 |
| 4HUW | 0.545 | 0.551 | 1.853 | 0.643 | 0.608 | 0.764 | 2.138 | 0.506 | 0.488 |
| 4HUX | 0.594 | 0.616 | 1.508 | 0.594 | 0.616 | 0.645 | 1.508 | 0.58 | 0.559 |
| 4HV8 | 0.199 | 0.309 | 1.32 | 0.199 | 0.309 | 0.306 | 1.32 | 0.211 | 0.309 |
| 4HWZ | 0.71 | 0.92 | 1.817 | 0.775 | 0.997 | 1.011 | 1.833 | 0.834 | 0.84 |
| 4HX1 | 1.016 | 1.063 | 2.756 | 1.325 | 1.337 | 1.585 | 3.966 | 1.426 | 0.829 |
| 4I48 | 1.185 | 1.306 | 1.738 | 1.734 | 1.737 | 1.947 | 2.811 | 1.543 | 1.122 |
| 4I4W | 0.544 | 0.916 | 2.212 | 1.305 | 1.452 | 1.635 | 3.646 | 1.241 | 0.828 |
| 4IHO | 0.434 | 0.576 | 1.065 | 0.434 | 0.576 | 0.562 | 1.065 | 0.598 | 0.576 |
| 4JFD | 0.253 | 0.291 | 0.82 | 0.253 | 0.291 | 0.393 | 0.82 | 0.295 | 0.291 |
| 4JFE | 1.952 | 2.044 | 1.953 | 2.224 | 2.102 | 1.98 | 1.953 | 2.256 | 1.261 |
| 4JFF | 0.28 | 0.495 | 0.735 | 0.28 | 0.495 | 0.53 | 0.735 | 0.479 | 0.495 |
| 4JFO | 0.746 | 0.984 | 1.18 | 0.746 | 0.984 | 0.959 | 1.18 | 1.245 | 0.984 |
| 4JFP | 0.211 | 0.268 | 0.783 | 0.211 | 0.268 | 0.325 | 0.783 | 0.28 | 0.268 |
| 4JFQ | 1.179 | 1.354 | 1.542 | 1.727 | 1.782 | 1.658 | 1.617 | 1.193 | 1.354 |
| 4JQV | 0.286 | 0.266 | 1.13 | 0.452 | 0.469 | 0.522 | 1.756 | 0.506 | 0.266 |
| 4JQX | 1.825 | 1.828 | 2.732 | 3.954 | 3.802 | 4.107 | 5.725 | 2.401 | 1.689 |
| 4JRX | 0.925 | 0.873 | 1.102 | 0.925 | 0.873 | 0.895 | 1.102 | 0.848 | 0.783 |
| 4JRY | 0.605 | 0.627 | 1.103 | 0.605 | 0.627 | 0.667 | 1.103 | 0.611 | 0.539 |
| 4K7F | 0.551 | 0.692 | 1.823 | 1.024 | 1.087 | 1.239 | 2.338 | 1.339 | 0.692 |
| 4L29 | 0.265 | 0.308 | 0.821 | 0.265 | 0.308 | 0.309 | 0.821 | 0.27 | 0.308 |
| 4L3C | 0.218 | 0.262 | 0.714 | 0.218 | 0.262 | 0.304 | 0.714 | 0.312 | 0.262 |
| 4L3E | 0.298 | 0.329 | 1.012 | 0.298 | 0.329 | 0.378 | 1.012 | 0.312 | 0.329 |
| 4L8B | 0.548 | 0.722 | 2.068 | 1.452 | 1.253 | 1.657 | 3.866 | 1.331 | 0.53 |
| 4L8C | 0.669 | 0.651 | 1.999 | 0.748 | 1.046 | 1.092 | 2.934 | 0.645 | 0.541 |
| 4L8D | 0.476 | 0.565 | 1.694 | 0.59 | 0.743 | 0.826 | 1.743 | 0.424 | 0.479 |
| 4LCY | 0.571 | 0.701 | 2.091 | 0.794 | 0.759 | 0.892 | 2.158 | 2.032 | 0.683 |
| 4LNR | 1.654 | 1.867 | 2.686 | 1.939 | 2.183 | 2.235 | 2.952 | 1.848 | 1.529 |
| 4MJ5 | 3.194 | 3.021 | 4.61 | 4.177 | 4.04 | 4.358 | 5.875 | 3.818 | 2.177 |
| 4MJ6 | 2.618 | 2.627 | 4.054 | 4.559 | 4.452 | 4.662 | 5.502 | 3.23 | 2.096 |
| 4MJI | 0.781 | 1.069 | 1.752 | 0.827 | 1.109 | 1.18 | 1.951 | 1.126 | 1.034 |
| 4MNQ | 0.395 | 0.52 | 1.641 | 0.942 | 0.867 | 1.119 | 3.057 | 0.866 | 0.444 |
| 4N8V | 0.62 | 0.674 | 1.934 | 1.564 | 1.664 | 1.96 | 3.961 | 0.871 | 0.564 |
| 4NNX | 0.343 | 0.391 | 0.84 | 0.343 | 0.391 | 0.421 | 0.84 | 0.364 | 0.391 |
| 4NNY | 0.831 | 1.045 | 1.567 | 1.849 | 1.95 | 2.045 | 2.525 | 1.9 | 0.746 |
| 4NO0 | 1.688 | 1.834 | 2.131 | 2.544 | 2.614 | 2.746 | 3.296 | 2.601 | 1.709 |
| 4NO2 | 1.934 | 1.992 | 2.378 | 2.443 | 2.524 | 2.664 | 3.253 | 2.529 | 1.608 |
| 4NO3 | 0.554 | 0.804 | 1.22 | 0.554 | 0.804 | 0.82 | 1.22 | 0.773 | 0.478 |
| 4NO5 | 0.489 | 0.726 | 1.309 | 0.489 | 0.833 | 0.807 | 1.309 | 0.825 | 0.701 |
| 4NQV | 0.528 | 0.448 | 1.225 | 0.528 | 0.448 | 0.598 | 1.43 | 1.079 | 0.39 |
| 4NQX | 0.802 | 0.839 | 1.72 | 0.874 | 1.043 | 1.109 | 1.891 | 0.628 | 0.758 |
| 4NSK | 0.487 | 0.525 | 1.184 | 0.526 | 0.525 | 0.555 | 1.184 | 0.565 | 0.525 |
| 4NT6 | 0.992 | 0.986 | 1.755 | 1.218 | 1.087 | 1.246 | 2.011 | 1.104 | 0.768 |
| 4O2E | 0.711 | 0.718 | 1.289 | 1.322 | 1.279 | 1.633 | 2.972 | 1.493 | 0.718 |
| 4O2F | 0.69 | 0.757 | 1.743 | 1.355 | 1.266 | 1.523 | 2.771 | 2.283 | 0.655 |
| 4PG2 | 0.883 | 0.918 | 2.539 | 0.886 | 0.947 | 0.977 | 2.539 | 0.979 | 0.776 |
| 4PG9 | 0.413 | 0.439 | 0.696 | 0.413 | 0.439 | 0.448 | 0.696 | 0.413 | 0.439 |
| 4PGB | 0.778 | 0.972 | 1.28 | 0.938 | 0.972 | 1.126 | 1.829 | 1.322 | 0.948 |
| 4PGD | 0.789 | 0.741 | 1.364 | 0.826 | 0.952 | 1.036 | 1.648 | 0.869 | 0.735 |
| 4PGE | 0.663 | 0.67 | 1.373 | 0.689 | 0.698 | 0.789 | 1.398 | 0.569 | 0.569 |
| 4PR5 | 2.348 | 2.315 | 3.423 | 2.734 | 2.634 | 2.928 | 4.316 | 2.684 | 1.909 |
| 4PRA | 0.759 | 0.74 | 2.296 | 0.759 | 0.74 | 0.764 | 2.296 | 0.732 | 0.74 |
| 4PRB | 1.427 | 1.301 | 2.859 | 2.039 | 2.096 | 2.133 | 3.325 | 2.083 | 1.301 |
| 4PRD | 1.641 | 1.561 | 3.025 | 2.908 | 2.762 | 3.029 | 4.484 | 2.748 | 1.464 |
| 4PRE | 0.798 | 0.804 | 2.195 | 0.798 | 0.804 | 0.848 | 2.195 | 0.828 | 0.804 |
| 4PRH | 1.527 | 1.628 | 2.579 | 2.338 | 2.164 | 2.452 | 4.089 | 2.139 | 1.586 |
| 4PRI | 0.874 | 1.136 | 2.304 | 1.613 | 1.605 | 1.646 | 2.622 | 1.544 | 1.087 |
| 4PRN | 1.923 | 1.93 | 3.327 | 1.967 | 2.021 | 2.028 | 3.327 | 2.04 | 1.069 |
| 4PRP | 1.931 | 1.92 | 3.704 | 3.555 | 3.35 | 3.705 | 5.41 | 3.359 | 1.339 |
| 4QOK | 0.361 | 0.556 | 1.058 | 0.361 | 0.556 | 0.574 | 1.058 | 0.521 | 0.556 |
| 4QRP | 0.53 | 0.552 | 1.193 | 0.53 | 0.552 | 0.617 | 1.193 | 0.659 | 0.552 |
| 4QRQ | 0.375 | 0.493 | 1.244 | 0.375 | 0.493 | 0.528 | 1.244 | 0.525 | 0.463 |
| 4QRR | 1.231 | 1.257 | 2.17 | 1.613 | 1.496 | 1.793 | 2.816 | 1.484 | 1.005 |
| 4QRS | 0.422 | 0.583 | 1.884 | 1.567 | 1.544 | 1.812 | 3.439 | 1.181 | 0.583 |
| 4QRT | 0.493 | 0.648 | 2.296 | 1.398 | 1.477 | 1.684 | 3.068 | 1.643 | 0.648 |
| 4QRU | 0.523 | 0.593 | 1.905 | 0.619 | 0.628 | 0.661 | 1.912 | 1.494 | 0.593 |
| 4U1H | 0.736 | 0.741 | 1.789 | 1.429 | 1.472 | 1.73 | 2.78 | 1.457 | 0.686 |
| 4U1I | 1.013 | 0.908 | 2.26 | 1.415 | 1.432 | 1.718 | 2.739 | 1.408 | 0.823 |
| 4U1J | 0.454 | 0.426 | 1.069 | 0.454 | 0.426 | 0.47 | 1.069 | 0.408 | 0.426 |
| 4U1K | 0.348 | 0.638 | 0.983 | 0.348 | 0.638 | 0.611 | 0.983 | 0.322 | 0.638 |
| 4U1L | 0.314 | 0.332 | 1.429 | 0.314 | 0.332 | 0.39 | 1.429 | 0.322 | 0.332 |
| 4U1N | 0.443 | 0.539 | 1.561 | 1.134 | 1.082 | 1.304 | 2.335 | 1.349 | 0.539 |
| 4U1S | 1.502 | 1.544 | 3.14 | 1.668 | 1.59 | 1.834 | 3.846 | 1.662 | 1.429 |
| 4U6Y | 3.304 | 3.225 | 4.507 | 6.093 | 6.029 | 6.139 | 6.879 | 5.134 | 3.225 |
| 4WDI | 1.695 | 1.744 | 2.396 | 2.849 | 2.692 | 2.96 | 3.911 | 2.684 | 1.735 |
| 4WU7 | 0.323 | 0.468 | 1.134 | 0.349 | 0.468 | 0.467 | 1.189 | 0.481 | 0.468 |
| 4WUU | 0.371 | 0.344 | 0.351 | 0.371 | 0.344 | 0.41 | 0.351 | 0.353 | 0.344 |
| 4XXC | 0.231 | 0.295 | 0.877 | 0.509 | 0.521 | 0.595 | 1.301 | 0.418 | 0.258 |
| 4Z76 | 0.702 | 0.619 | 1.158 | 0.702 | 0.619 | 0.741 | 1.158 | 0.606 | 0.619 |
| 4Z77 | 0.721 | 0.654 | 1.308 | 0.729 | 0.893 | 0.95 | 1.308 | 0.877 | 0.64 |
| 4Z78 | 1.04 | 1.247 | 2.705 | 2.18 | 2.08 | 2.28 | 4.102 | 1.619 | 1.247 |
| 4ZEZ | 1.149 | 1.311 | 2.011 | 1.811 | 1.709 | 1.66 | 2.052 | 1.615 | 1.247 |
| 4ZUS | 1.45 | 1.7 | 2.463 | 2.172 | 1.965 | 2.315 | 3.623 | 2.495 | 1.521 |
| 4ZUT | 2.744 | 2.664 | 2.94 | 3.093 | 3.084 | 3.377 | 3.708 | 3.095 | 2.437 |
| 4ZUU | 1.877 | 1.901 | 2.891 | 2.287 | 2.363 | 2.522 | 2.891 | 1.546 | 1.496 |
| 4ZUV | 2.434 | 2.491 | 3.391 | 4.153 | 4.078 | 4.189 | 4.529 | 4.338 | 2.451 |
| 4ZUW | 1.404 | 1.547 | 2.415 | 3.162 | 3.159 | 3.429 | 3.969 | 3.135 | 1.547 |
| 5B38 | 0.224 | 0.209 | 0.602 | 0.224 | 0.209 | 0.248 | 0.602 | 0.186 | 0.209 |
| 5B39 | 0.224 | 0.268 | 0.59 | 0.224 | 0.268 | 0.282 | 0.59 | 0.246 | 0.268 |
| 5BRZ | 0.573 | 0.626 | 1.015 | 0.721 | 0.74 | 0.788 | 1.085 | 0.652 | 0.558 |
| 5BS0 | 0.687 | 0.66 | 1.412 | 1.622 | 1.611 | 1.886 | 2.473 | 0.927 | 0.616 |
| 5C07 | 0.378 | 0.381 | 0.732 | 0.378 | 0.381 | 0.4 | 0.732 | 0.369 | 0.381 |
| 5C08 | 0.336 | 0.354 | 1.118 | 0.336 | 0.354 | 0.373 | 1.118 | 0.339 | 0.354 |
| 5C09 | 0.771 | 0.772 | 1.148 | 0.771 | 0.772 | 0.915 | 1.496 | 0.776 | 0.772 |
| 5C0A | 0.345 | 0.401 | 1.339 | 0.345 | 0.401 | 0.441 | 1.339 | 0.409 | 0.401 |
| 5C0B | 0.276 | 0.459 | 1.355 | 0.276 | 0.459 | 0.454 | 1.355 | 0.463 | 0.459 |
| 5C0C | 1.045 | 1.022 | 1.536 | 1.045 | 1.022 | 1.188 | 1.708 | 1.028 | 1.022 |
| 5C0D | 0.488 | 0.563 | 0.878 | 0.488 | 0.563 | 0.599 | 0.878 | 0.586 | 0.563 |
| 5C0E | 0.38 | 0.421 | 1.062 | 0.38 | 0.421 | 0.429 | 1.062 | 0.395 | 0.421 |
| 5C0F | 0.323 | 0.342 | 1.06 | 0.323 | 0.342 | 0.377 | 1.06 | 0.332 | 0.342 |
| 5C0G | 0.756 | 0.826 | 1.713 | 0.756 | 0.826 | 0.935 | 1.883 | 0.823 | 0.826 |
| 5C0H | 0.756 | 0.826 | 1.713 | 0.756 | 0.826 | 0.935 | 1.883 | 0.823 | 0.826 |
| 5C0I | 0.356 | 0.501 | 1.373 | 0.356 | 0.501 | 0.504 | 1.373 | 0.461 | 0.501 |
| 5C0J | 0.883 | 1.014 | 1.591 | 1.011 | 1.014 | 1.174 | 1.633 | 1.025 | 0.968 |
| 5CNZ | 0.097 | 0.108 | 0.642 | 0.097 | 0.108 | 0.119 | 0.642 | 0.109 | 0.108 |
| 5D2L | 0.77 | 0.809 | 1.14 | 0.858 | 0.881 | 0.973 | 1.14 | 0.892 | 0.764 |
| 5D2N | 0.509 | 0.65 | 1.007 | 0.696 | 0.759 | 0.814 | 1.007 | 0.756 | 0.648 |
| 5D9S | 0.659 | 0.91 | 1.137 | 0.723 | 0.949 | 0.918 | 1.137 | 1.776 | 0.82 |
| 5DDH | 0.83 | 1.317 | 2.22 | 0.83 | 1.317 | 1.354 | 2.234 | 3.536 | 1.317 |
| 5DEG | 0.715 | 0.72 | 2.379 | 0.76 | 0.72 | 0.809 | 2.379 | 0.933 | 0.592 |
| 5E00 | 0.854 | 0.766 | 2.185 | 0.986 | 1.008 | 1.113 | 2.281 | 0.525 | 0.628 |
| 5E8N | 0.44 | 0.432 | 1.055 | 0.534 | 0.567 | 0.665 | 2.742 | 0.569 | 0.432 |
| 5E9D | 0.318 | 0.329 | 0.832 | 0.318 | 0.329 | 0.369 | 0.832 | 0.336 | 0.329 |
| 5ENW | 0.464 | 0.547 | 1.1 | 0.603 | 0.66 | 0.705 | 1.432 | 0.58 | 0.544 |
| 5EO0 | 0.542 | 0.58 | 1.668 | 1.963 | 1.765 | 2.147 | 4.622 | 1.731 | 0.562 |
| 5EO1 | 0.803 | 1.03 | 1.966 | 1.03 | 1.248 | 1.33 | 2.457 | 1.451 | 1.007 |
| 5EOT | 1.886 | 1.728 | 1.457 | 1.913 | 1.751 | 1.641 | 1.529 | 4.336 | 1.724 |
| 5EU3 | 0.588 | 0.699 | 1.115 | 0.928 | 0.988 | 0.969 | 1.335 | 0.964 | 0.549 |
| 5EU4 | 0.726 | 0.826 | 0.95 | 0.979 | 1.187 | 1.132 | 1.178 | 0.402 | 0.588 |
| 5EU5 | 0.606 | 0.732 | 1.407 | 0.916 | 1.012 | 1.17 | 1.638 | 0.965 | 0.7 |
| 5EU6 | 0.557 | 0.533 | 1.442 | 0.713 | 0.801 | 0.831 | 1.55 | 0.527 | 0.533 |
| 5EUO | 0.37 | 0.474 | 0.966 | 0.37 | 0.474 | 0.479 | 1.031 | 0.483 | 0.474 |
| 5F1I | 1.123 | 1.183 | 1.514 | 1.165 | 1.183 | 1.251 | 1.772 | 1.432 | 0.788 |
| 5F7D | 0.773 | 0.974 | 1.308 | 1.09 | 1.232 | 1.188 | 1.719 | 2.984 | 0.948 |
| 5F9J | 0.394 | 0.585 | 0.918 | 1.404 | 1.528 | 1.714 | 1.999 | 1.404 | 0.554 |
| 5FA3 | 0.299 | 0.381 | 0.463 | 0.342 | 0.386 | 0.404 | 0.463 | 1.013 | 0.311 |
| 5FA4 | 0.81 | 0.904 | 1.323 | 1.285 | 1.443 | 1.608 | 1.901 | 4.908 | 0.899 |
| 5FDW | 1.146 | 1.318 | 1.603 | 3.247 | 3.181 | 3.335 | 3.583 | 1.271 | 1.318 |
| 5GR7 | 1.625 | 1.726 | 2.577 | 1.841 | 1.86 | 2.146 | 2.881 | 1.619 | 1.694 |
| 5GRD | 2.562 | 2.463 | 3.055 | 3.551 | 3.428 | 3.656 | 3.886 | 2.982 | 2.306 |
| 5GSB | 0.708 | 0.794 | 1.345 | 0.887 | 0.925 | 0.97 | 1.427 | 0.946 | 0.794 |
| 5GSD | 1.545 | 1.424 | 1.754 | 1.695 | 1.547 | 1.775 | 2.34 | 2.071 | 1.394 |
| 5GSR | 0.507 | 0.612 | 1.034 | 0.805 | 0.987 | 1.011 | 1.191 | 0.618 | 0.611 |
| 5GSX | 1.569 | 1.412 | 1.957 | 2.381 | 2.186 | 2.548 | 3.572 | 1.913 | 1.322 |
| 5H5Z | 0.097 | 0.108 | 0.642 | 0.097 | 0.108 | 0.119 | 0.642 | 0.109 | 0.108 |
| 5H94 | 1.632 | 1.884 | 2.865 | 2.319 | 2.464 | 2.632 | 4.166 | 1.823 | 1.439 |
| 5HGA | 0.313 | 0.497 | 0.885 | 0.319 | 0.497 | 0.476 | 1.09 | 0.483 | 0.497 |
| 5HGD | 0.782 | 0.832 | 1.592 | 0.782 | 0.832 | 0.879 | 1.592 | 0.822 | 0.832 |
| 5HGH | 0.839 | 1.089 | 1.628 | 0.839 | 1.089 | 1.062 | 1.628 | 1.082 | 1.089 |
| 5HHM | 0.955 | 1.097 | 1.584 | 0.955 | 1.097 | 1.103 | 1.762 | 1.042 | 1.024 |
| 5HHN | 0.833 | 0.92 | 1.826 | 0.875 | 0.969 | 1.011 | 1.826 | 0.979 | 0.678 |
| 5HHO | 0.631 | 0.734 | 1.599 | 0.631 | 0.734 | 0.782 | 2.055 | 0.734 | 0.645 |
| 5HHP | 0.569 | 0.695 | 1.203 | 0.654 | 0.731 | 0.77 | 2.194 | 0.761 | 0.669 |
| 5HHQ | 0.511 | 0.724 | 2.18 | 0.72 | 0.853 | 0.909 | 2.696 | 1.149 | 0.68 |
| 5HYJ | 0.466 | 0.599 | 0.92 | 0.466 | 0.599 | 0.62 | 0.92 | 0.615 | 0.599 |
| 5IB1 | 1.019 | 1.063 | 2.569 | 1.69 | 1.659 | 1.922 | 4.141 | 1.669 | 0.703 |
| 5IB2 | 1.032 | 1.11 | 2.381 | 1.703 | 1.657 | 1.935 | 4.089 | 1.669 | 0.738 |
| 5IB3 | 0.22 | 0.257 | 0.712 | 0.22 | 0.257 | 0.266 | 0.712 | 0.192 | 0.257 |
| 5IB4 | 0.165 | 0.191 | 0.558 | 0.165 | 0.191 | 0.209 | 0.558 | 0.16 | 0.191 |
| 5IB5 | 0.29 | 0.318 | 0.826 | 0.29 | 0.318 | 0.352 | 0.826 | 0.317 | 0.318 |
| 5IEH | 0.163 | 0.219 | 0.262 | 0.163 | 0.219 | 0.288 | 0.262 | 0.239 | 0.219 |
| 5IEK | 0.236 | 0.288 | 0.337 | 0.236 | 0.288 | 0.329 | 0.337 | 0.259 | 0.288 |
| 5IM7 | 0.748 | 0.75 | 1.39 | 1.481 | 1.628 | 1.805 | 2.836 | 2.085 | 0.614 |
| 5INC | 0.887 | 1.01 | 1.695 | 1.17 | 1.197 | 1.405 | 2.104 | 1.145 | 0.979 |
| 5IND | 0.591 | 0.695 | 1.313 | 1.414 | 1.379 | 1.557 | 2.086 | 0.559 | 0.597 |
| 5IRO | 0.803 | 0.881 | 2.258 | 1.471 | 1.338 | 1.582 | 3.306 | 1.361 | 0.788 |
| 5ISZ | 0.329 | 0.477 | 0.583 | 0.329 | 0.477 | 0.474 | 0.583 | 0.484 | 0.477 |
| 5IUE | 6.353 | 5.807 | 6.371 | 6.361 | 5.812 | 5.905 | 6.637 | 5.084 | 5.797 |
| 5IVX | 0.349 | 0.346 | 1.002 | 0.349 | 0.346 | 0.367 | 1.002 | 0.34 | 0.346 |
| 5J6G | 0.344 | 0.389 | 0.487 | 0.363 | 0.389 | 0.401 | 0.487 | 0.351 | 0.389 |
| 5J6H | 0.359 | 0.41 | 0.773 | 0.359 | 0.41 | 0.391 | 0.773 | 0.329 | 0.41 |
| 5JHD | 0.505 | 0.556 | 0.641 | 0.505 | 0.556 | 0.618 | 0.641 | 0.565 | 0.556 |
| 5JWD | 0.635 | 0.59 | 1.873 | 0.652 | 0.59 | 0.696 | 1.877 | 0.691 | 0.59 |
| 5JWE | 0.749 | 1.055 | 1.572 | 0.846 | 1.055 | 1.114 | 2.208 | 1.167 | 0.995 |
| 5JZI | 0.809 | 0.874 | 0.948 | 0.838 | 0.931 | 0.924 | 1.013 | 0.955 | 0.85 |
| 5KD4 | 0.905 | 1.318 | 2.306 | 1.908 | 1.728 | 1.989 | 4.14 | 1.623 | 1.159 |
| 5KD7 | 0.576 | 0.705 | 2.401 | 1.288 | 1.215 | 1.505 | 2.546 | 1.18 | 0.631 |
| 5M00 | 0.294 | 0.308 | 1.026 | 0.294 | 0.308 | 0.332 | 1.726 | 0.291 | 0.308 |
| 5M01 | 0.25 | 0.283 | 0.944 | 0.25 | 0.283 | 0.293 | 1.635 | 0.217 | 0.283 |
| 5M02 | 0.532 | 0.516 | 0.948 | 0.532 | 0.516 | 0.53 | 0.985 | 0.514 | 0.502 |
| 5MEO | 0.621 | 0.888 | 2.486 | 1.339 | 1.44 | 1.778 | 3.68 | 1.459 | 0.742 |
| 5MEP | 0.693 | 0.822 | 1.45 | 0.999 | 0.976 | 1.098 | 2.157 | 1.451 | 0.763 |
| 5MER | 0.843 | 0.953 | 2.472 | 1.672 | 1.688 | 1.953 | 3.678 | 1.913 | 0.944 |
| 5MZM | 0.58 | 0.688 | 1.27 | 0.585 | 0.725 | 0.708 | 1.895 | 0.859 | 0.611 |
| 5N1Y | 0.392 | 0.429 | 1.558 | 0.392 | 0.429 | 0.491 | 1.558 | 0.399 | 0.429 |
| 5N6B | 0.829 | 0.73 | 1.391 | 0.829 | 0.73 | 0.861 | 1.391 | 0.679 | 0.657 |
| 5NHT | 0.653 | 0.843 | 1.216 | 0.653 | 0.843 | 0.825 | 1.216 | 0.813 | 0.843 |
| 5NME | 0.547 | 0.698 | 1.361 | 0.718 | 0.893 | 0.891 | 1.4 | 0.904 | 0.698 |
| 5NMF | 0.594 | 0.627 | 1.106 | 0.668 | 0.759 | 0.758 | 1.348 | 0.738 | 0.528 |
| 5NMG | 0.351 | 0.455 | 0.933 | 0.566 | 0.65 | 0.667 | 1.342 | 0.618 | 0.455 |
| 5NMH | 0.572 | 0.861 | 1.215 | 0.572 | 0.945 | 0.904 | 1.232 | 0.957 | 0.861 |
| 5NMK | 0.386 | 0.458 | 1.019 | 0.386 | 0.458 | 0.487 | 1.019 | 0.474 | 0.458 |
| 5NPZ | 0.377 | 0.495 | 0.949 | 0.377 | 0.495 | 0.503 | 0.949 | 0.481 | 0.495 |
| 5NQ0 | 0.805 | 0.86 | 1.744 | 0.805 | 0.86 | 0.854 | 1.744 | 0.809 | 0.86 |
| 5NQ1 | 0.828 | 0.868 | 1.571 | 0.828 | 0.868 | 0.881 | 1.571 | 0.904 | 0.868 |
| 5NQ2 | 0.989 | 0.99 | 3.329 | 1.828 | 1.819 | 2.132 | 3.923 | 1.856 | 0.672 |
| 5NQ3 | 0.274 | 0.37 | 0.759 | 0.274 | 0.37 | 0.404 | 0.759 | 0.388 | 0.357 |
| 5NQK | 0.557 | 0.695 | 1.108 | 0.557 | 0.695 | 0.678 | 1.108 | 0.649 | 0.695 |
| 5SWQ | 0.616 | 0.718 | 1.377 | 1.469 | 1.479 | 1.74 | 3.149 | 1.369 | 0.532 |
| 5SWS | 0.59 | 0.647 | 1.36 | 0.59 | 0.647 | 0.692 | 1.36 | 0.614 | 0.529 |
| 5SWZ | 0.524 | 0.554 | 1.285 | 0.524 | 0.556 | 0.59 | 1.285 | 0.535 | 0.487 |
| 5T6W | 0.759 | 0.925 | 1.572 | 0.937 | 1.055 | 1.159 | 2.102 | 2.49 | 0.799 |
| 5T6X | 0.694 | 0.735 | 1.124 | 1.451 | 1.417 | 1.716 | 2.119 | 2.971 | 0.735 |
| 5T6Z | 0.721 | 0.75 | 1.535 | 0.784 | 0.75 | 0.853 | 1.535 | 2.927 | 0.61 |
| 5T70 | 1.297 | 1.248 | 2.435 | 2.108 | 2.154 | 2.252 | 2.929 | 2.101 | 1.206 |
| 5T7G | 0.615 | 0.746 | 2.179 | 1.218 | 1.149 | 1.43 | 2.445 | 1.116 | 0.548 |
| 5TEZ | 0.411 | 0.439 | 1.16 | 0.411 | 0.439 | 0.474 | 1.177 | 0.462 | 0.439 |
| 5TIL | 0.492 | 0.509 | 1.307 | 0.492 | 0.509 | 0.544 | 1.318 | 0.503 | 0.47 |
| 5TJE | 0.568 | 0.768 | 1.304 | 0.568 | 0.768 | 0.758 | 2.341 | 0.75 | 0.768 |
| 5TRZ | 0.642 | 1.001 | 1.721 | 0.842 | 1.104 | 1.127 | 1.721 | 3.949 | 1.001 |
| 5TXS | 0.731 | 0.883 | 0.97 | 1.012 | 1.068 | 1.253 | 1.175 | 1.162 | 0.813 |
| 5V5L | 0.841 | 0.802 | 1.712 | 0.841 | 0.802 | 0.909 | 1.712 | 2.696 | 0.743 |
| 5V5M | 0.479 | 0.48 | 0.771 | 0.479 | 0.48 | 0.492 | 0.771 | 2.783 | 0.48 |
| 5VCL | 0.293 | 0.291 | 0.755 | 0.293 | 0.291 | 0.31 | 0.755 | 0.285 | 0.291 |
| 5VGD | 1.146 | 1.261 | 2.288 | 2.393 | 2.334 | 2.747 | 4.196 | 2.216 | 1.089 |
| 5VGE | 0.567 | 0.618 | 1.114 | 0.797 | 0.865 | 0.833 | 1.174 | 0.861 | 0.618 |
| 5VUD | 0.615 | 0.626 | 1.191 | 0.984 | 0.926 | 1.199 | 2.224 | 0.913 | 0.564 |
| 5VUE | 0.472 | 0.523 | 1.168 | 1.454 | 1.449 | 1.695 | 2.37 | 1.456 | 0.381 |
| 5VUF | 0.717 | 0.729 | 1.545 | 1.447 | 1.558 | 1.707 | 2.615 | 1.564 | 0.692 |
| 5VVP | 0.887 | 0.84 | 1.332 | 0.971 | 0.942 | 1.183 | 2.29 | 0.919 | 0.813 |
| 5VWD | 0.638 | 0.616 | 1.521 | 1.457 | 1.428 | 1.683 | 2.384 | 1.424 | 0.616 |
| 5VWF | 0.712 | 0.75 | 2.147 | 1.435 | 1.548 | 1.697 | 2.5 | 1.534 | 0.64 |
| 5VWH | 0.279 | 0.319 | 0.512 | 0.279 | 0.319 | 0.32 | 0.512 | 0.305 | 0.319 |
| 5VWJ | 0.485 | 0.644 | 0.886 | 0.485 | 0.667 | 0.659 | 0.886 | 0.647 | 0.491 |
| 5VZ5 | 2.387 | 2.33 | 3.882 | 3.483 | 3.462 | 3.749 | 5.583 | 3.499 | 2.028 |
| 5W1V | 1.044 | 1.06 | 1.729 | 2.629 | 2.439 | 2.775 | 4.091 | 2.491 | 1.028 |
| 5W69 | 0.736 | 0.84 | 2.048 | 1.645 | 1.496 | 1.826 | 3.602 | 1.671 | 0.82 |
| 5WEU | 1.07 | 1.359 | 1.557 | 1.367 | 1.437 | 1.5 | 1.998 | 1.293 | 1.15 |
| 5WJL | 0.97 | 1.238 | 1.424 | 0.97 | 1.238 | 1.257 | 1.424 | 1.244 | 1.238 |
| 5WJN | 0.779 | 1.013 | 1.403 | 0.779 | 1.013 | 0.993 | 1.403 | 0.957 | 1.013 |
| 5WKF | 0.968 | 1.212 | 1.435 | 0.968 | 1.212 | 1.264 | 1.435 | 1.226 | 1.212 |
| 5WKH | 0.709 | 0.988 | 1.236 | 0.709 | 0.988 | 0.972 | 1.236 | 1.014 | 0.988 |
| 5WLG | 0.659 | 0.643 | 0.851 | 0.774 | 0.744 | 0.78 | 0.851 | 0.731 | 0.643 |
| 5WLI | 0.676 | 0.762 | 0.9 | 0.756 | 0.762 | 0.797 | 0.9 | 0.767 | 0.696 |
| 5WMN | 0.838 | 0.771 | 1.319 | 1.095 | 0.999 | 1.12 | 1.623 | 0.851 | 0.628 |
| 5WMO | 0.714 | 0.643 | 1.41 | 0.777 | 0.752 | 0.862 | 1.41 | 0.719 | 0.643 |
| 5WMP | 1.637 | 1.789 | 1.941 | 2.715 | 2.727 | 2.712 | 4.065 | 2.625 | 1.406 |
| 5WMQ | 0.497 | 0.519 | 2.597 | 1.274 | 1.217 | 1.386 | 3.901 | 1.106 | 0.519 |
| 5WMR | 0.8 | 0.744 | 2.091 | 0.814 | 0.861 | 0.944 | 2.37 | 0.703 | 0.636 |
| 5WSH | 0.414 | 0.488 | 1.449 | 0.488 | 0.5 | 0.598 | 1.449 | 0.826 | 0.44 |
| 5WWI | 0.483 | 0.625 | 1.251 | 0.483 | 0.625 | 0.644 | 1.251 | 0.627 | 0.625 |
| 5WWU | 0.793 | 0.759 | 2.362 | 1.49 | 1.477 | 1.611 | 3.639 | 0.754 | 0.759 |
| 5WXD | 0.5 | 0.897 | 1.354 | 0.5 | 0.905 | 0.848 | 1.354 | 0.896 | 0.89 |
| 5XMF | 0.54 | 0.679 | 1.314 | 1.699 | 1.445 | 1.813 | 2.425 | 0.939 | 0.607 |
| 5XMM | 0.502 | 0.502 | 1.028 | 0.601 | 0.532 | 0.618 | 1.539 | 0.86 | 0.465 |
| 5XOS | 0.635 | 0.954 | 1.367 | 0.895 | 1.123 | 1.13 | 1.367 | 1.094 | 0.582 |
| 5XOT | 0.404 | 0.451 | 1.392 | 0.903 | 1.08 | 1.092 | 1.504 | 1.083 | 0.421 |
| 5XOV | 0.148 | 0.184 | 0.764 | 0.148 | 0.184 | 0.217 | 0.764 | 0.178 | 0.184 |
| 5XS3 | 1.368 | 1.323 | 2.416 | 2.195 | 2.155 | 2.542 | 4.612 | 2.976 | 1.159 |
| 5Y91 | 0.171 | 0.222 | 1.128 | 0.171 | 0.222 | 0.235 | 1.692 | 0.212 | 0.222 |
| 5YLX | 0.53 | 0.586 | 1.649 | 1.081 | 1.222 | 1.418 | 3.111 | 1.164 | 0.536 |
| 5YMV | 1.273 | 1.755 | 2.353 | 1.302 | 1.776 | 1.799 | 2.426 | 2.719 | 1.715 |
| 5YMW | 0.555 | 0.521 | 1.359 | 0.629 | 0.633 | 0.726 | 1.465 | 0.627 | 0.503 |
| 5YXN | 0.445 | 0.618 | 1.048 | 0.445 | 0.618 | 0.626 | 1.048 | 0.649 | 0.602 |
| 5YXU | 0.536 | 0.636 | 0.963 | 0.536 | 0.636 | 0.647 | 0.963 | 0.677 | 0.61 |
| 6A6H | 1.757 | 2.12 | 2.747 | 1.987 | 2.33 | 2.383 | 2.887 | 2.553 | 2.083 |
| 6AEE | 0.362 | 0.437 | 1.228 | 0.415 | 0.437 | 0.45 | 1.228 | 0.441 | 0.437 |
| 6AMT | 1.29 | 1.32 | 2.196 | 2.266 | 2.335 | 2.217 | 2.711 | 2.412 | 1.32 |
| 6AMU | 1.847 | 1.934 | 2.586 | 2.015 | 2.252 | 2.166 | 2.949 | 2.673 | 1.645 |
| 6AT5 | 1.907 | 2.015 | 2.247 | 4.242 | 4.318 | 4.35 | 3.971 | 4.339 | 1.823 |
| 6AT9 | 2.121 | 2.058 | 4.041 | 2.551 | 2.467 | 2.793 | 5.151 | 2.446 | 1.735 |
| 6AVF | 3.059 | 2.986 | 2.944 | 4.24 | 4.224 | 4.29 | 3.95 | 4.257 | 2.986 |
| 6AVG | 1.762 | 1.823 | 2.039 | 2.731 | 2.83 | 2.881 | 2.901 | 2.875 | 1.456 |
| 6D29 | 1.507 | 1.435 | 2.35 | 1.507 | 1.435 | 1.709 | 3.207 | 1.406 | 1.395 |
| 6D2R | 1.168 | 1.237 | 2.428 | 1.383 | 1.452 | 1.584 | 2.496 | 1.452 | 0.972 |
| 6D2T | 1.137 | 1.099 | 1.862 | 1.906 | 1.913 | 2.064 | 2.587 | 1.872 | 1.093 |
| 6D78 | 2.714 | 2.738 | 2.862 | 3.394 | 3.293 | 3.296 | 3.82 | 3.276 | 2.738 |
| 6DKP | 0.403 | 0.405 | 1.043 | 0.403 | 0.405 | 0.44 | 1.043 | 0.356 | 0.405 |
| 6EQA | 0.424 | 0.486 | 0.775 | 0.424 | 0.486 | 0.524 | 0.775 | 2.455 | 0.486 |
| 6EQB | 0.499 | 0.563 | 0.85 | 0.499 | 0.563 | 0.582 | 0.85 | 2.534 | 0.563 |
| 6EWA | 0.343 | 0.312 | 0.705 | 0.343 | 0.312 | 0.351 | 0.705 | 0.283 | 0.312 |
| 6EWC | 0.814 | 0.82 | 1.542 | 0.969 | 0.926 | 1.017 | 1.888 | 0.538 | 0.593 |
| 6EWO | 1.296 | 1.257 | 2.486 | 1.963 | 1.863 | 2.16 | 2.991 | 1.366 | 0.964 |
| 6G3J | 0.727 | 0.84 | 1.419 | 1.745 | 1.626 | 1.548 | 1.687 | 1.669 | 0.519 |
| 6G3K | 1.583 | 1.598 | 1.568 | 3.098 | 2.913 | 2.906 | 3.276 | 1.087 | 1.103 |
| 6G9Q | 0.577 | 0.552 | 1.132 | 0.577 | 0.552 | 0.615 | 1.132 | 0.542 | 0.552 |
| 6G9R | 0.602 | 0.585 | 1.242 | 0.602 | 0.585 | 0.666 | 1.242 | 0.614 | 0.529 |
| 6GB5 | 0.162 | 0.242 | 0.626 | 0.167 | 0.242 | 0.319 | 0.626 | 3.85 | 0.242 |
| 6GB7 | 0.213 | 0.235 | 0.444 | 0.213 | 0.235 | 0.249 | 0.444 | 3.864 | 0.235 |
| 6GGM | 0.601 | 0.793 | 1.456 | 0.857 | 0.879 | 0.937 | 1.491 | 0.68 | 0.669 |
| 6GH1 | 0.518 | 0.537 | 1.453 | 0.52 | 0.537 | 0.59 | 1.453 | 0.784 | 0.518 |
| 6GH4 | 0.477 | 0.507 | 1.331 | 0.649 | 0.686 | 0.728 | 1.391 | 0.471 | 0.493 |
| 6GHN | 0.999 | 1.112 | 1.816 | 1.175 | 1.112 | 1.197 | 1.936 | 1.014 | 0.894 |
| 6GL1 | 0.877 | 0.912 | 1.263 | 2.035 | 2.118 | 2.322 | 2.62 | 1.199 | 0.912 |
| 6H6D | 0.269 | 0.267 | 0.437 | 0.269 | 0.267 | 0.293 | 0.437 | 0.249 | 0.267 |
| 6H6H | 0.263 | 0.284 | 0.525 | 0.263 | 0.284 | 0.318 | 0.525 | 0.268 | 0.284 |
| 6ID4 | 0.595 | 0.784 | 1.071 | 0.648 | 0.784 | 0.843 | 1.071 | 0.764 | 0.721 |
| 6IEX | 1.266 | 1.214 | 1.676 | 1.893 | 1.864 | 2.162 | 3.291 | 1.917 | 1.214 |
| 6IRL | 0.402 | 0.449 | 1.335 | 0.489 | 0.499 | 0.51 | 1.414 | 0.561 | 0.449 |
| 6J1V | 0.745 | 0.809 | 1.84 | 0.771 | 0.809 | 0.858 | 1.996 | 0.858 | 0.767 |
| 6J1W | 0.674 | 0.744 | 1.446 | 0.814 | 0.806 | 0.85 | 1.446 | 0.699 | 0.695 |
| 6J29 | 0.937 | 0.887 | 2.41 | 0.963 | 0.903 | 0.998 | 2.627 | 0.772 | 0.789 |
| 6J2A | 0.722 | 0.898 | 1.572 | 1.6 | 1.703 | 1.778 | 2.61 | 1.122 | 0.512 |
| 6JOZ | 0.559 | 0.709 | 1.037 | 1.408 | 1.425 | 1.606 | 1.742 | 1.547 | 0.69 |
| 6JQ3 | 0.354 | 0.5 | 1.003 | 0.43 | 0.55 | 0.567 | 1.068 | 0.628 | 0.5 |
| 6JTN | 0.826 | 0.904 | 1.387 | 2.682 | 2.385 | 2.787 | 4.238 | 2.378 | 0.904 |
| 6JTO | 1.007 | 1.258 | 1.91 | 1.214 | 1.398 | 1.603 | 2.734 | 1.424 | 1.123 |
| 6JTP | 0.327 | 0.557 | 1.111 | 0.327 | 0.75 | 0.715 | 1.111 | 0.751 | 0.546 |
| 6K60 | 0.347 | 0.395 | 1.745 | 0.347 | 0.395 | 0.387 | 1.745 | 0.403 | 0.395 |
| 6KWK | 1.071 | 1.265 | 1.872 | 1.393 | 1.645 | 1.741 | 2.077 | 1.527 | 1.265 |
| 6KWL | 0.638 | 0.721 | 1.388 | 1.457 | 1.442 | 1.633 | 2.151 | 1.443 | 0.721 |
| 6KWN | 0.813 | 0.862 | 1.529 | 1.389 | 1.318 | 1.413 | 1.591 | 1.305 | 0.632 |
| 6KWO | 0.972 | 0.991 | 1.714 | 1.437 | 1.377 | 1.399 | 1.831 | 1.596 | 0.953 |
| 6LBE | 1.669 | 1.485 | 2.799 | 1.819 | 1.803 | 2.102 | 2.799 | 1.79 | 1.022 |
| 6LHF | 0.409 | 0.462 | 1.859 | 0.409 | 0.462 | 0.477 | 1.859 | 0.437 | 0.462 |
| 6LHG | 0.353 | 0.372 | 1.488 | 0.395 | 0.411 | 0.48 | 1.681 | 0.384 | 0.359 |
| 6LHH | 0.355 | 0.396 | 1.659 | 0.355 | 0.396 | 0.426 | 1.659 | 0.41 | 0.396 |
| 6MPP | 1.504 | 1.379 | 1.292 | 2.26 | 2.104 | 2.425 | 1.991 | 2.049 | 1.297 |
| 6MT3 | 1.189 | 1.224 | 2.521 | 1.189 | 1.224 | 1.326 | 2.521 | 0.858 | 1.141 |
| 6MT4 | 0.328 | 0.385 | 0.928 | 0.328 | 0.385 | 0.433 | 0.928 | 0.372 | 0.385 |
| 6MT5 | 0.768 | 0.818 | 1.611 | 1.824 | 1.689 | 2.017 | 3.597 | 0.783 | 0.377 |
| 6MT6 | 0.283 | 0.28 | 0.957 | 0.283 | 0.28 | 0.324 | 0.957 | 0.279 | 0.28 |
| 6MTL | 1.168 | 1.135 | 2.577 | 2.07 | 1.987 | 2.244 | 4.271 | 1.887 | 1.101 |
| 6MTM | 0.283 | 0.278 | 0.805 | 0.283 | 0.278 | 0.33 | 0.805 | 0.291 | 0.278 |
| 6NCA | 0.634 | 0.759 | 1.531 | 0.843 | 0.928 | 0.96 | 1.745 | 0.804 | 0.759 |
| 6NF7 | 4.693 | 4.766 | 6.489 | 5.67 | 5.573 | 5.842 | 7.221 | 7.127 | 4.594 |
| 6NPR | 0.336 | 0.343 | 1.208 | 0.336 | 0.343 | 0.384 | 1.208 | 0.337 | 0.343 |
| 6O4Y | 0.503 | 0.63 | 1.044 | 0.788 | 1.158 | 1.076 | 1.264 | 0.643 | 0.491 |
| 6O4Z | 0.676 | 0.753 | 1.158 | 0.858 | 0.833 | 1.022 | 1.343 | 0.401 | 0.718 |
| 6O51 | 0.629 | 0.752 | 0.905 | 1.897 | 1.709 | 1.975 | 2.165 | 1.096 | 0.705 |
| 6O53 | 0.552 | 0.605 | 0.991 | 1.392 | 1.295 | 1.569 | 2.175 | 1.187 | 0.519 |
| 6O9B | 0.447 | 0.79 | 0.806 | 1.272 | 1.292 | 1.484 | 2.286 | 0.469 | 0.413 |
| 6O9C | 0.552 | 0.764 | 0.96 | 0.951 | 0.955 | 1.007 | 1.417 | 0.723 | 0.513 |
| 6OPD | 0.755 | 0.808 | 1.634 | 1.079 | 1.196 | 1.405 | 2.428 | 1.898 | 0.508 |
| 6P23 | 0.172 | 0.172 | 0.685 | 0.172 | 0.172 | 0.198 | 0.685 | 1.798 | 0.172 |
| 6P27 | 0.176 | 0.165 | 0.48 | 0.176 | 0.165 | 0.201 | 0.48 | 1.796 | 0.165 |
| 6P2C | 0.257 | 0.227 | 0.494 | 0.257 | 0.227 | 0.277 | 0.494 | 1.811 | 0.227 |
| 6P2F | 0.391 | 0.396 | 0.971 | 0.451 | 0.425 | 0.48 | 1.157 | 1.949 | 0.388 |
| 6P2S | 0.896 | 0.801 | 0.77 | 0.896 | 0.801 | 0.816 | 0.77 | 1.46 | 0.801 |
| 6P64 | 0.363 | 0.577 | 1.135 | 0.681 | 0.807 | 0.859 | 1.135 | 0.834 | 0.44 |
| 6PA1 | 0.583 | 0.673 | 1.081 | 0.654 | 0.756 | 0.748 | 1.128 | 0.807 | 0.597 |
| 6PAG | 0.288 | 0.324 | 0.809 | 0.288 | 0.324 | 0.355 | 0.809 | 0.32 | 0.324 |
| 6PTB | 0.328 | 0.524 | 1.712 | 0.328 | 0.524 | 0.571 | 1.712 | 0.652 | 0.489 |
| 6PTE | 0.624 | 0.685 | 1.528 | 0.92 | 1.09 | 1.285 | 2.21 | 1.216 | 0.621 |
| 6PYJ | 0.176 | 0.214 | 0.65 | 0.176 | 0.214 | 0.238 | 0.65 | 0.169 | 0.214 |
| 6PYL | 1.771 | 1.751 | 3.261 | 2.834 | 2.684 | 2.815 | 4.2 | 1.567 | 1.495 |
| 6PYV | 0.139 | 0.193 | 0.775 | 0.139 | 0.193 | 0.225 | 0.775 | 0.169 | 0.193 |
| 6PYW | 0.133 | 0.144 | 0.524 | 0.133 | 0.144 | 0.165 | 0.524 | 0.152 | 0.144 |
| 6PZ5 | 0.171 | 0.18 | 0.4 | 0.171 | 0.18 | 0.193 | 0.4 | 0.201 | 0.18 |
| 6Q3K | 0.267 | 0.319 | 0.655 | 0.284 | 0.331 | 0.336 | 0.655 | 0.307 | 0.272 |
| 6Q3S | 0.578 | 0.496 | 1.297 | 0.578 | 0.496 | 0.576 | 1.297 | 0.51 | 0.496 |
| 6R2L | 0.847 | 0.89 | 1.769 | 0.847 | 0.901 | 0.955 | 1.769 | 1.336 | 0.832 |
| 6RP9 | 1.451 | 1.468 | 1.903 | 1.699 | 1.811 | 2.015 | 3.946 | 1.818 | 1.314 |
| 6RPA | 0.363 | 0.528 | 1.103 | 0.363 | 0.528 | 0.516 | 1.103 | 0.534 | 0.528 |
| 6RPB | 0.549 | 0.771 | 1.353 | 0.639 | 0.771 | 0.775 | 1.353 | 0.8 | 0.715 |
| 6RSY | 0.674 | 0.786 | 1.095 | 0.768 | 0.842 | 0.861 | 1.095 | 0.823 | 0.786 |
| 6SS7 | 0.786 | 0.835 | 1.157 | 0.927 | 0.835 | 0.807 | 1.157 | 0.86 | 0.722 |
| 6SS8 | 0.796 | 0.863 | 1.358 | 1.382 | 1.271 | 1.192 | 1.5 | 0.729 | 0.708 |
| 6SS9 | 0.826 | 0.886 | 1.199 | 0.884 | 0.886 | 0.95 | 1.544 | 0.823 | 0.876 |
| 6SSA | 0.888 | 0.918 | 1.552 | 1.064 | 0.985 | 1.043 | 1.552 | 1.491 | 0.909 |
| 6TMO | 0.356 | 0.566 | 1.01 | 0.356 | 0.566 | 0.577 | 1.01 | 0.525 | 0.566 |
| 6TRN | 0.65 | 0.861 | 1.91 | 1.43 | 1.443 | 1.557 | 3.759 | 1.274 | 0.723 |
| 6TRO | 0.462 | 0.427 | 1.294 | 0.462 | 0.427 | 0.482 | 1.294 | 0.403 | 0.427 |
| 6UJO | 0.772 | 0.811 | 1.169 | 0.772 | 0.879 | 0.922 | 1.169 | 0.875 | 0.573 |
| 6UJQ | 0.46 | 0.545 | 1.293 | 0.46 | 0.545 | 0.591 | 1.293 | 0.554 | 0.545 |
| 6UK2 | 0.384 | 0.434 | 1.081 | 0.457 | 0.514 | 0.555 | 1.497 | 0.471 | 0.431 |
| 6UK4 | 0.291 | 0.324 | 0.694 | 0.291 | 0.324 | 0.334 | 0.694 | 0.331 | 0.324 |
| 6ULI | 0.24 | 0.47 | 0.8 | 0.24 | 0.47 | 0.459 | 0.8 | 0.472 | 0.47 |
| 6ULK | 1.613 | 1.43 | 1.933 | 2.704 | 2.408 | 2.807 | 4.278 | 2.421 | 0.908 |
| 6ULN | 0.327 | 0.534 | 0.896 | 0.327 | 0.534 | 0.532 | 0.896 | 0.537 | 0.534 |
| 6ULR | 0.475 | 0.569 | 0.921 | 0.475 | 0.569 | 0.602 | 1.025 | 0.559 | 0.569 |
| 6UON | 0.757 | 0.871 | 1.186 | 0.757 | 0.871 | 0.927 | 1.186 | 0.898 | 0.871 |
| 6UZ1 | 0.66 | 0.777 | 1.336 | 1.135 | 1.062 | 1.287 | 2.155 | 1.062 | 0.703 |
| 6V2O | 0.167 | 0.399 | 0.6 | 0.167 | 0.399 | 0.375 | 0.6 | 0.393 | 0.399 |
| 6V2P | 0.214 | 0.387 | 0.725 | 0.214 | 0.387 | 0.378 | 0.725 | 0.39 | 0.387 |
| 6V2Q | 0.393 | 0.435 | 1.383 | 1.581 | 1.486 | 1.834 | 2.399 | 1.462 | 0.435 |
| 6V3J | 0.741 | 0.753 | 1.741 | 1.555 | 1.605 | 1.89 | 2.604 | 1.479 | 0.73 |
| 6VMX | 0.475 | 0.489 | 1.384 | 0.711 | 0.694 | 0.752 | 1.384 | 0.703 | 0.489 |
| 6VR1 | 0.93 | 1.122 | 2.629 | 1.466 | 1.412 | 1.805 | 3.088 | 1.323 | 1.041 |
| 6VR5 | 0.728 | 0.846 | 1.344 | 0.728 | 0.889 | 0.92 | 1.344 | 0.888 | 0.837 |
| 6VRN | 0.836 | 0.989 | 1.531 | 0.836 | 0.989 | 1.058 | 1.531 | 0.969 | 0.938 |
| 6WZY | 0.849 | 0.966 | 1.265 | 0.849 | 0.966 | 0.986 | 1.265 | 1.332 | 0.929 |
| 6X00 | 1.015 | 1.181 | 1.943 | 2.327 | 2.172 | 2.481 | 2.959 | 1.291 | 1.148 |
| 6Y26 | 0.472 | 0.669 | 0.761 | 0.807 | 0.798 | 0.931 | 0.825 | 0.761 | 0.669 |
| 6Y28 | 0.206 | 0.243 | 0.537 | 0.206 | 0.243 | 0.257 | 0.537 | 0.2 | 0.243 |
| 6Y29 | 0.207 | 0.895 | 1.229 | 0.207 | 0.895 | 0.927 | 1.229 | 0.223 | 0.895 |
| 6Y2A | 0.187 | 0.432 | 0.577 | 0.187 | 0.432 | 0.412 | 0.577 | 0.41 | 0.432 |
| 6Y2B | 0.208 | 0.45 | 1.056 | 0.208 | 0.45 | 0.448 | 1.056 | 0.43 | 0.45 |
| 7K80 | 0.471 | 0.533 | 1.357 | 0.511 | 0.533 | 0.562 | 1.357 | 0.502 | 0.533 |
| 7K81 | 0.435 | 0.464 | 1.442 | 0.501 | 0.518 | 0.547 | 1.442 | 0.495 | 0.464 |
| 1LD9 | 0.921 | 1.105 | 1.89 | 0.972 | 1.155 | 1.208 | 2.024 | 1.155 | 1.048 |
| 1TVH | 0.559 | 0.49 | 1.523 | 0.623 | 0.546 | 0.648 | 1.936 | 0.546 | 0.49 |
| 4WU5 | 0.435 | 0.43 | 1.257 | 0.435 | 0.43 | 0.46 | 1.257 | 0.43 | 0.423 |
