# Supplementary material for: PANDORA: A Fast, Anchor-Restrained Modelling Protocol for Peptide: MHC Complexes
Source: Front Immunol. 2022 May 10;13:878762. doi: 10.3389/fimmu.2022.878762 (PMC9127323; doi:10.3389/fimmu.2022.878762)
Supplement: Supplementary file 4 [file Table_3.docx]

Supplementary Table 3. List of non-canonical amino acids tolerated by PANDORA parsing step.

| PDB ID | PDB url link |
| --- | --- |
| CIR | https://www.rcsb.org/ligand/CIR |
| CSO | https://www.rcsb.org/ligand/CSO |
| F2F | https://www.rcsb.org/ligand/F2F |
| SEP | https://www.rcsb.org/ligand/SEP |
